# The effects of anti-inflammatory agents as host-directed adjunct treatment of tuberculosis in humans: a systematic review and meta-analysis

**DOI:** 10.1186/s12931-020-01488-9

**Published:** 2020-08-26

**Authors:** Frank Ekow Atta Hayford, Robin Claire Dolman, Renee Blaauw, Arista Nienaber, Cornelius Mattheus Smuts, Linda Malan, Cristian Ricci

**Affiliations:** 1 Centre of Excellence for Nutrition, Faculty of Health Sciences, Building G16, North- West University, Potchefstroom Campus, Potchefstroom, South Africa; 2 Department of Nutrition and Dietetics, School of Biomedical and Allied Health Sciences, College of Health Sciences, University of Ghana, Accra, Ghana; 3 Division of Human Nutrition, Stellenbosch University, Cape Town, South Africa; 4 Department of Pediatric Epidemiology, Department of Pediatrics, Medical Faculty , University/Institution: Leipzig University, Leipzig, Germany

**Keywords:** Adjunctive treatment, Anti-inflammatory agents, Host-directed therapy, Sputum conversion rate, Systematic review & meta-analysis, Tuberculosis

## Abstract

**Background:**

The potential role of adjunctive anti-inflammatory therapy to enhance tuberculosis (TB) treatment has recently received increasing interest. There is, therefore, a need to broadly examine current host-directed therapies (HDTs) that could accelerate treatment response and improve TB outcomes.

**Methods:**

This systematic review and meta-analysis included randomised controlled trials of vitamin D and other HDT agents in patients receiving antibiotic treatment for pulmonary TB. Sputum smear conversion rate at 4–8 weeks was the primary outcome. Secondary outcomes included blood indices associated with infectivity and inflammation, chest radiology and incidence of adverse events.

**Results:**

Fifty-five studies were screened for eligibility after the initial search, which yielded more than 1000 records. Of the 2540 participants in the 15 trials included in the meta-analysis, 1898 (74.7%) were male, and the age at entry ranged from 18 to 70 years. There was a 38% significantly (*RR* 1.38, 95% *CI =* 1.03–1.84) increased sputum smear negativity in patients administered with vitamin D in addition to standard TB treatment than those receiving only the TB treatment. Patients treated with other HDT anti-inflammatory agents in addition to TB treatment also had a 29% significantly increased sputum smear conversion rate (*RR* 1.29, 95% *CI =* 1.09–1.563). Lymphocyte to monocyte ratio was significantly higher in the vitamin D treatment groups compared to the controls (3.52 vs 2.70, 95% *CI* for difference 0.16–1.11, *p* = 0.009) and (adjusted mean difference 0.4, 95% CI 0.2 -- 0.6; *p* = 0.001); whilst tumour necrosis factor-alpha (TNF-α) showed a trend towards a reduction in prednisolone (*p* < 0.001) and pentoxifylline (*p* = 0.27) treatment groups. Vitamin D and N-acetylcysteine also accelerated radiographic resolution in treatment compared to placebo at 8 weeks. No differences were observed in the occurrence of adverse events among all HDT treatments.

**Conclusions:**

Vitamin D and other anti-inflammatory HDT medications used as adjunct TB treatment may be well tolerated and effective. They significantly improved sputum smear conversion rate and chest radiological appearance, and also exhibited an inflammation resolution effect.

## Background

According to the World Health Organization, in 2017, mortality from TB and TB-related deaths stood at 1.6 million, making TB the leading cause of death from a single infectious agent worldwide, surpassing HIV/AIDS [1]. Despite the decrease in numbers of TB deaths by 22% between 2000 and 2015 and that the incidence rate continues to fall globally, the cost of TB treatment is still high. About US$ 10.4 billion was required in low- and middle-income countries to successfully continue with the Global Plan to End TB 2016–2020 by The Stop TB Partnership in 2018 [1].

Even though multiple antibiotic therapies administered for 6 months are used to prevent the development of drug resistance, the prolonged treatment duration, coupled with the toxicity of the drugs, contributes to patient non-compliance [2, 3]. This can contribute to the development of drug-resistant *Mycobacteria tuberculosis (Mtb*), multi-drug-resistant (MDR) and extensive drug-resistant (XDR) strains [4]. Host-directed therapy (HDT) is an emerging concept in the treatment of TB, aimed at directly modulating host cell functions, and is administered in addition to antimicrobial treatment. Thus, the development of drug resistance by *Mtb* may be reduced or even avoided and better control of TB may be achieved [5, 6]. Functionally, HDT agents improve the antimicrobial activities of host immune cells and limit inflammation and tissue damage associated with TB [2, 7, 8].

Current drug regimens are mainly directed at various targets on the *Mtb* bacillus [9, 10]; this means that even after cure, TB patients are at risk of residual damage due to inflammatory effects (post TB fibrosis and bronchiectasis) [11]. Thus, supplementing anti-TB drug treatment with host immune modulators may lead to shorter treatment time, reduced lung damage, lower risk of relapse or reinfection and also improvement of other clinical outcomes [2, 10, 12–14].

The role of anti-inflammatory agents such as vitamin D, phenylbutyrate, prednisolone and others have been studied as HDT in human randomised control trials (RCTs) with varied success [15–18]. Potential advantageous functions that were identified, were to accelerate the resolution of the inflammatory responses and to improve other clinical outcomes associated with increased risk of mortality [19]. The addition of these agents to the therapy of pulmonary TB (PTB) was shown to significantly accelerate sputum culture and/or smear conversion rates, as well as clinical and radiographic improvement, even though some yielded conflicting results [14, 20, 21].

In addition to their anti-inflammatory functions, HDT may also provide other immune-modulating advantages. Vitamin D (cholecalciferol-D3) is a known immune modulator in TB, associated with cathelicidin-mediated killing of mycobacterium [22, 23]. It has also been shown to suppress NF-κB signalling pathways, expression of matrix metalloproteinases (MMPs), and pro-inflammatory cytokines and chemokines, thus accelerating the resolution of inflammation [19]. Prednisone is a glucocorticoid receptor antagonist that has the ability to downregulate the production of pro-inflammatory cytokines such as tumour necrosis factor (TNF) [24]. Recombinant human interleukin, used as HDT, also enhances both the bacterial clearance and immune response when administered twice daily for 30 days [25]. N-acetylcysteine, another HDT molecule, effectively increases glutathione (GSH), which is usually low in TB patients due to oxidative stress [26, 27]. Increased levels of GSH have been demonstrated to play a positive role in phagocytosis, T-cell activation responses and balancing in TB [28]. In human monocyte-derived macrophage cell lines, recombinant human granulocyte-macrophage colony-stimulating factor, which has cytokine regulatory abilities, was observed to reduce *Mycobacterium* growth, indicating its potential use as adjunct TB treatment [29]. All this evidence supports the potential use of HDT molecules to enhance TB treatment.

Currently, most meta-analyses conducted in the area of HDT in TB are on vitamin D, and results seem to suggest that it does not influence the time to sputum culture and/or smear conversion [6, 30–32]. However, one meta-analysis indicated that vitamin D accelerated sputum culture conversion rate in patients with MDR pulmonary TB [6]. Other anti-inflammatory agents, such as prednisolone, also accelerated the sputum culture conversion when used as HDT in pulmonary TB [17]. Considering that there are other agents used as HDTs, it would be prudent to consider effects of these agents in addition to vitamin D. Therefore, the present meta-analysis was conducted to determine and estimate the overall efficacy and safety of vitamin D and all other anti-inflammatory agents that have been used as adjunct treatment in patients with TB, namely recombinant human interleukin, prednisolone, pentoxifylline, N-acetylcysteine and recombinant human granulocyte-macrophage colony-stimulating factor. The efficacy and safety of HDT agents were determined in relation to the administered dosages. The analysis focused on smear status as the primary outcome between 4 and 8 weeks of treatment, considering the role of this marker as an indicator of sterilizing activity [33] and as a predictor of relapse risk [34, 35].

## Methods

The review process and findings are reported according to – and are consistent with – the PRISMA guidelines [36].

### Eligibility (inclusion and exclusion criteria)

Eligibility of included studies was based on the following criteria: 1) double-blind placebo-controlled RCTs, 2) trials that were conducted in newly diagnosed PTB patients who were on initial anti-tuberculosis treatment with sputum smear-positive for acid-fast bacilli or culture positive for *Mtb* at baseline, 3) the HDT agent was used alone as an adjunct to standard anti-TB treatment, 4) trials were conducted in patients aged ≥18 years, 5) treatment outcome measured between 4 and 8 weeks, and 6) trials reporting the sputum smear or culture conversion rate (proportion of participants with sputum smear or culture conversion from positive to negative). Furthermore, trials in which a factorial design was used to investigate the effects of other therapies alongside the anti-inflammatory therapy of interest (such that they could be isolated) were also included. Trials conducted in children or adolescents; single-blinded RCTs, non-PTB, controlled experiments that were not randomised (quasi-experimental studies) and open-label trials that were not blinded, were excluded.

### Search strategies

In this meta-analysis, two authors searched Medline (Additional file 2), Cochrane Central Register of Controlled Trials, embase, Web of Science and Google scholar for trials, using the keywords “pulmonary tuberculosis” or “tuberculoses”, “anti-inflammatory agents” and “host-directed-therapeutics”, with no limitations in the publication type, language or period. The detailed search string was as follows: Tuberculosis [MeSH Terms] OR tuberculoses [MeSH Terms] OR *Mycobacterium tuberculosis* [MeSH Terms] AND *Mycobacterium tuberculoses* [MeSH Terms] OR pulmonary tuberculosis [MeSH Terms] OR pulmonary tuberculoses [MeSH Terms] OR adjunctive immunotherapy in tuberculosis [MeSH Terms] OR adjunctive immunotherapy in tuberculoses [MeSH Terms] OR adjunct tuberculosis treatment [MeSH Terms] AND adjunct tuberculoses treatment [MeSH Terms] OR host-directed-therapeutics in tuberculosis [MeSH Terms] OR host-directed-therapeutics in tuberculoses [MeSH Terms] OR anti-tubercular agent [MeSH Terms] OR anti-inflammatory agents [MeSH Terms] AND sputum smear conversion [MeSH Terms] OR sputum culture conversion [MeSH Terms] OR immune inflammatory response [MeSH Terms] OR anti-inflammatory response [MeSH Terms] OR inflammatory response [MeSH Terms] AND immunomodulatory regulators [MeSH Terms] OR immuno modulators [MeSH Terms] OR human [MeSH Terms] OR human subjects [MeSH Terms] OR patients [MeSH Terms] AND adults [MeSH Terms] AND randomised control trials [MeSH Terms]**.** The database search was from inception up to and including November 2019 and was regularly updated. The reference or citation listed in each identified article was also reviewed for potentially relevant studies. These searches were supplemented by searches of review articles and reference lists of trial publications. When full-length articles were not available from the databases, these were requested from the authors. Four authors then determined which studies met the eligibility criteria.

### Data extraction and quality assessment

Four authors were involved in data extraction and quality assessment. Two authors independently performed the initial extraction of data.

Data relating to the following study characteristics were collected: authors, publication year, trial registration number, study setting, mode of anti-TB therapy administration, details of adjunctive therapies being evaluated in each trial (type, dose and route of treatment) and primary study outcome. Baseline characteristics of trial participant data were extracted for the following variables: age, sex ratio, body mass index (BMI), mid-upper arm circumference (MUAC), number of participants randomised and those included, intervention treatment duration and follow-up period, HIV status, proportion with MDR-TB, extent of disease on baseline chest radiograph (as measured by the proportion of zones involved and the presence or absence of cavitation), TB score, C-reactive protein (CRP), erythrocyte sedimentation rate (ESR), other blood indices, adverse effects and mortality in a standard form as recommended by Cochrane [37, 38]. Follow-up data after 4–8 weeks of initiating antimicrobial and adjunct treatment were also extracted, namely sputum smear and culture status to estimate conversion rates, time from initiation of antibiotic treatment to stable sputum smear/culture conversion (the date of stable smear/culture conversion being estimated as the mid-point between the date of the last positive sputum smear/culture and the date of the first negative sputum smear/culture thereafter); TB score, chest radiography, weight gain, BMI, MUAC, CRP, ESR and other blood indices, as well as adverse effects and mortality. For any missing information, corresponding authors were contacted by email. Three items were used to assess the methodological quality of each included study, based on the Jadad scale [39], which includes criteria of randomisation, blinding, and addressing the problem of incomplete outcome data (withdrawals and dropouts). The other two authors were consulted to solve any disagreement on study selection, data extraction, or quality assessment.

### Definition of outcome measures

The primary and secondary outcome measures of the meta-analysis comprised of efficacy assessment and safety evaluation, respectively. The primary outcome was sputum smear conversion rate, estimated as the percentage of smear-positive PTB (PTB+) cases registered in a specified period that converted to smear-negative status [40]. Secondary outcomes included sputum smear and culture time to conversion, sputum culture conversion rate, TB score, chest radiography, weight gain, BMI, MUAC, CRP, ESR, other blood indices associated with infectivity and inflammation (total white blood cells, lymphocytes, neutrophils, monocytes, interferon gamma (IFN-γ), and TNF-α), incidence of potential adverse reactions attributable to the study intervention agents and all-cause mortality. These outcome measures are considered very relevant in TB disease progression and treatment success.

### Risk of bias across and within individual studies

Risk of bias assessment was conducted using the Cochrane Collaboration Risk of Bias tool [37], considering the following parameters: sequence generation, allocation concealment, blinding of participants, personnel and outcome assessors, completeness of outcome data, evidence of selective outcome reporting, and other potential threats to validity. The Cochrane risk of bias tool was used for the assessment of risk of bias in estimating the study outcomes. The selective reporting within studies was assessed by answering whether the results were fully reported, as the study was pre-specified (for example, if all the results were reported at all follow-up time points). For the primary analysis, the likelihood of publication bias was investigated through the construction of a funnel plot and Egger’s test [41, 42]. Study quality, in this case, was assessed independently by three authors. Discrepancies were resolved by a fourth author.

### Statistical methods

Meta-analysis of sputum smear conversion rate was conducted using both random and fixed effect methods, resulting in conversion hazard of the treatment group with respect to the control group. Study weights reported on the forest plots were derived from the random effect analysis and were computed as w_i_ = 1/(s_i_^2^ + t^2^) with s_i_^2^ being the variance estimate from the i-th study and t^2^ representing the overall variance. Standard errors were computed as di/1.96 where di = max [(log (upper 95% confidence interval bound)-log (RRi); (log (RRi)-log (lower 95% bound)].

Heterogeneity between studies was reported by means of Cochrane Q test and I^2^ statistic [43]. When relevant heterogeneity was detected (significant Cochrane Q test and/or I^2^ > 50%), stratification and study exclusion were conducted to identify its source. Publication bias was assessed by means of funnel plot visual inspection and Egger’s test [42]. Influence analysis, excluding one study at a time, was conducted to assess robustness of results.

Statistical analysis was conducted using STATA, vers 15. Meta-analysis was performed using the metan function. Funnel plots, Egger’s test and influence analyses were performed using the metafunnel, metabias and the metainf functions, respectively. *P* value less than 0.05 was considered statistically significant.

## Results

### Description and quality assessment of included studies

**Fig. 1:**
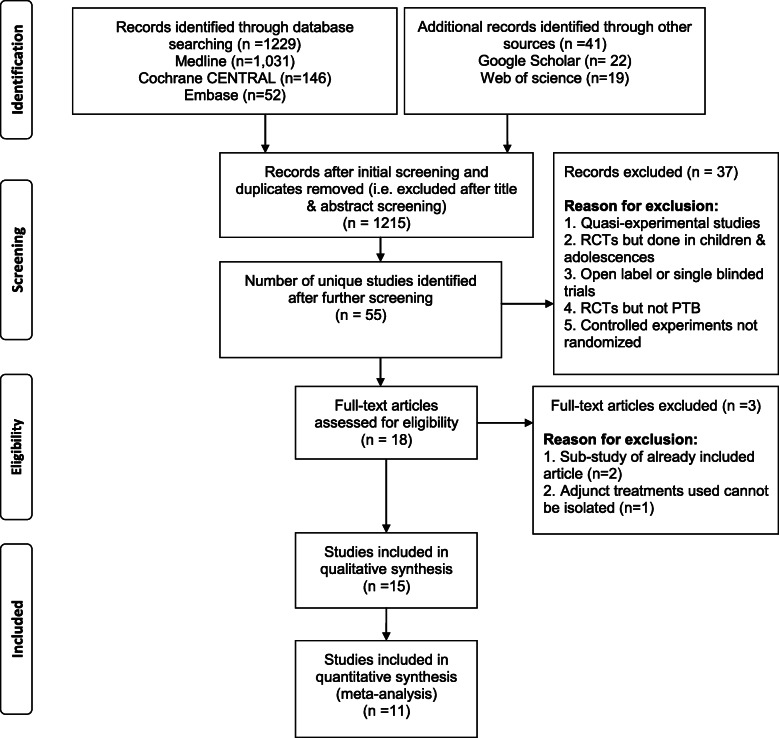
PRISMA flow diagram of study selection and inclusion process. CENTRAL: Central Register of Controlled Trials; RCT: randomised controlled trial; PTB: pulmonary tuberculosis

The initial search yielded more than 1000 records (Additional file 1). After the initial screening and duplicates were removed, the remaining 55 unique studies identified were further screened for eligibility. Out of the 18 full-text articles identified and assessed for eligibility, 15 studies met the inclusion criteria of this meta-analysis. Some reasons for excluding articles, were that the adjunct treatments used could not be isolated and some of the articles were sub-studies of an article that was already included. Fifteen articles were included in the qualitative, whereas only 11 in the final quantitative synthesis due to the unavailability of data from authors. The detailed literature search and selection process is shown in Fig. 1. The clinical trials were conducted in 12 countries and most of them were registered as described in Table 1. Ten studies investigated the effect of vitamin D [14–16, 18, 20, 21, 23, 44–46] as adjunct HDT agent, while five studies used either recombinant human interleukin [25], prednisolone [47], pentoxifylline [48], N-acetylcysteine [49] or recombinant human granulocyte-macrophage colony-stimulating factor [50]. Out of the fifteen studies, thirteen studies used two-arm parallel designs to investigate the effects of only one HDT treatment [14–16, 21, 23, 25, 44–50]. Two studies included L-arginine [20] and phenylbutyrate [18] concurrently with vitamin D as HDT in a factorial design such that they could be isolated. The treatments were administered either by oral [15, 16, 18, 20, 21, 44–49], intramuscular [14, 23], intradermal [25] or subcutaneous [50] routes. Apart from three studies [15, 16, 21], all others followed the standard anti-TB therapy regime of 2 months of isoniazid, rifampicin, pyrazinamide, ethambutol (HRZE), then 4 months of rifampicin and isoniazid (RH) (Table 1). All studies included sputum smear and/or culture conversion as a primary (or co-primary) or secondary outcome measure. The following studies reported these as primary outcomes: sputum culture conversion rate [15, 16, 18, 20, 21, 25, 46], sputum smear conversion rate [23, 44, 49], TB clinical scores [18, 20, 23, 45], chest radiograph resolution [14], weight gain [14], treatment safety [47], treatment tolerance [50] and treatment effect [48] (Table 2). According to the Jadad scale, all the trials included in the meta-analysis were considered high-quality studies as shown in supplementary Table 1 (Jadad score 3–5). All trials achieved the maximum quality score of 5, except two studies which scored 3 [44] and 4 [50] on the scale. Details of the quality assessment are provided in supplementary Table 1 (Additional file 2).

**Table 1:** Characteristics of each trial included in meta-analysis

Author (year)	Study setting (s)	Trial registration	Anti-TB therapy	Type, dose and route of treatment (intervention)
Martineau 2011 [21]	UK	NCT00419068	2 months HRZE	Vit D_3_, 2.5 mg,oral
Daley 2015 [16]	India	NCT00366470	2 months HRZE	Vit D_3,_ 2.5 mg,oral
Farazi 2017 [36]	Iran	IRCT201407029855N5	2 months HRZE then 4 months RH	Vit D_3_,11.2 mg,intramuscular
Mily 2015 [18]	Bangladesh	NCT01580007	2 months HRZE then 4 months RH	Vit D_3_,0.13 mg and PBA,500 mg,oral
Nursyam 2006 [37]	Indonesia	NM	2 months HRZE then 4 months RH	Vit D_3_, 0.25 mg,oral
Ralph 2013 [20]	Indonesia	NCT00677339	2 months HRZE then 4 months RH	Vit D_3_,1.25 mg, and L-arginine,6 g,oral
Salahuddin 2013 [14]	Pakistan	NCT01130311	2 months HRZE, then6 months HE	Vit D_3_,15 mg, intramuscular
Tukvadze 2015 [39]	Georgia	NCT00918086	2 months HRZE then 4 months RH	Vit D_3_,1.25 mg, oral
Wejse 2009 [38]	Guinea-Bissau	ISRCTN35212132	2 months HRZE, then 6 months HE	Vita D_3_, 2.5 mg,oral
Johnson 2003 [40]	Uganda	NM	2 months HRZE then 4 months RH	rIL-2,11.2 mg,intradermal
Mayanja-Kizza 2005 [41]	Uganda	NM	2 months HRZE then 4 months RH	Prednisolone, 2.75 mg,oral
Pedral-Sampaio 2003 [44]	Brazil	NM	2 months HRZ then 4 months RH	Rhu-GM-CSF,125 μg, subcutaneous
Willis 1996 [42]	Ugandan	NM	2 months HRZ then 4 months RH	Pentoxifylline,1800 mg,oral
Mahakalkar 2017 [43]	India	not registered	2 months HRZ then 4 months RH	N-acetylcysteine,600 mg,oral
Ganmaa 2017 [15]	Mongolia	NCT01657656	2 months HRZE	vit D_3_, 3.5 mg,oral

*NM* not mentioned, *Vit* Vitamins, *HRZE* isoniazid,rifampicin, pyrazinamide, ethambutol, *RH* rifampicin & isoniazid, *HE* isoniazid & ethambutol, *mg* milligram, *μg* microgram, *g* gram, *PBA* phenylbutyrate, *Rhu-GM-CSF* recombinant human granulocyte-macrophage colony-stimulating factor, *rIL* recombinant human Interleukin, *UK* United kingdom, *Anti-TB* Anti-tuberculosis, *vit D*_*3*_ vitamin D_3_

**Table 2:** Baseline characteristics of enrolled participants for each trial included in meta-analysis: I

Author (year)	Primary outcome	Chest X-ray cavities present, number (I/C) ^b^	^a^Chest X-ray Zones affected (mean, SD)(I/C)	BMI (mean, SD)(kg/m^2^)(I/C)	TB score (mean, SD)(I/C)	CRP (mean, SD) (mg/L)(I/C)	ESR (mean, SD) (mm/h)(I/C)
Martineau 2011 [21]	sputum culture conversion	36 (58)/36 (56)	2.8 (1.3)/ 2.8 (1.3)	20.1 (3.1)/20.2 (2.7)	ND	71.4 (49.5)/60.5 (45.0)	62.1 (23.1) /60.9 (17.4)
Daley 2015 [16]	sputum culture conversion	ND	ND	18.0 (2.9)/17.8 (3.0)	ND	ND	ND
Farazi 2017 [36]	TB clinical score sputum conversion (co-primary)	ND	ND	20.75 (3.8) /21.2 (4.1)	6.3 (2.4)/ 6.1 (2.2)	26 (86.7%) /25 (83.3%)	25 (83.3%)/27 (90%)
Mily 2015 [18]	sputum culture conversion, TB clinical score (co-primary)	ND	ND	NM	7.9 (5.6)/ 8.0 (5.0)	26.2 (0.4)/32.1 (0.4)	55.9 (48.4)/60.2 (34.9)
Nursyam 2006 [37]	sputum conversion	ND	ND	16.87 (2.06)/ 17.68 (2.54)	ND	ND	ND
Ralph 2013 [20]	sputum culture conversion, TB clinical score (co-primary)	42 (25–73)/ 36.5 (20.5–57.5)	70 (36–94)/ 66.5 (29.5–91)	19.1 (13.3–32.5)/ 19.3 (12.0–26.3)	6.9 (2.0)/ 6.8 (1.9)	ND	ND
Salahuddin 2013 [14]	Weight gain, chest radiograph resolution (Co-primary).	3.61 (1.40)/3.64 (1.48)	ND	17.2 (11–25)/17.3 (11–27)	6.68 (2.04)/ 6.85 (2.50)	ND	ND
Tukvadze 2015 [39]	sputum culture conversion	15 (15.0)/21 (21.2)	ND	ND	ND	ND	ND
Wejse 2009 [38]	TB clinical score	ND	ND	18.8 (12–33)/18.5 (12–27)	6.7 (6.4–7.0)/ 6.8 (6.5–7.1)	ND	ND
Johnson 2003 [40]	sputum culture conversion	54 (98)/52 (95)	ND	NM	ND	ND	ND
Mayanja-Kizza 2005 [41]	Treatment safety	ND	ND	19 (2.8)/19 (2.6)	ND	ND	ND
Pedral-Sampaio 2003 [44]	Treatment tolerance	ND	ND	ND	ND	ND	ND
Willis 1996 [42]	Effect on treatment	ND	ND	ND	ND	ND	ND
Mahakalkar 2017 [43]	sputum conversion	11.01 ± 2/9.27 ± 1.93^c^	ND	ND	ND	ND	ND
Ganmaa 2017 [15]	Sputum culture conversion	95 (50.0)/100 (50.0)	7.4 (4.4)/7.3 (4.4)	19.7 (2.8)/20.1 (3.1)	ND	62.7 (46.1)/ 63.0 (46.7)	17.2 (10.7)/15.8 (11.3)

*ND* not determined, *I/C* intervention & control groups, *SD* standard deviation, *mm/h* millimetres/hour, *mg/L* milligram/litre, *kg/m* kilogram/metre, *BMI* body mass index, *TB* tuberculosis, *CRP* C-reactive protein, *ESR* erythrocyte sedimentation rate

^a^:Chest radiograph; b:in percentage(%); ^c^: mean value

### Study participants characteristics

A total of 2728 randomised participants from 15 studies fulfilled the eligibility criteria, of which 2540 were included in the final analysis. One thousand two hundred and sixty-nine patients received treatment, whilst 1271 were on identical placebo in addition to TB treatment. Two thousand and fifty-seven participants contributed data for the vitamin D treatment group whilst 483 participants contributed data for the other anti-inflammatory agents group, for analysis of various study outcomes. The treatment outcome endpoint measured, ranged from 4 to 8 weeks, with follow-up duration up to 32 weeks. The age range of the 2540 participants included in the meta-analysis was from 18 to 70 years in both the intervention and control groups, and 1898 (74.7%) were male. Administration of treatment to participants in the intervention arm was as follows: treatment was administered at baseline only [23], daily [18, 25, 44, 47–49], twice a week [50], weekly then 2-weekly [46], 2-weekly [15, 16, 21], 4-weekly [20], 3 times over a period of 32 weeks (at baseline, 20 weeks and 32 weeks) [45] and at baseline and 4 weeks [14]. Four hundred and three (28.6%) of the 1411 participants in 11 studies [16, 20, 21, 23, 25, 44–48, 50] tested positive for HIV status at baseline. The proportion of participants with MDR-TB (i.e. their *Mtb* complex isolate was resistant to at least isoniazid and rifampicin) was 3.9%, which is 57 out of 1460 reported in 7 studies [15, 16, 18, 20, 21, 25, 46] (Table 3).

**Table 3:** Baseline characteristics of enrolled participants for each trial included in meta-analysis: II

Author (year)	Age (yrs) (mean or median) (I/C)	Sex ratio Male/Female	Participants randomised/included^d^, Treatment administered^c^	Proportion HIV infected at baseline^a^	Proportion with MDR-TB^a^
Martineau 2011 [21]	30.7 (24.5–41.5) /30.5 (24.8–38.4)^b^	98/28	146/126, at 0, 2, 4 & 6 weeks	5/93 (5.4)	1/135 (0.7)
Daley 2015 [16]	41·6 ± 15·1/ 43·7 ± 14·3″	189/58	247/211, at 0, 2, 4, & 6 weeks	0/209 (0)	1/209 (0.5)
Farazi 2017 [36]	52.4 ± 17.6/ 51.8 ± 18.2″	31/29	68/60 at week 0 only	0/60 (0)	ND
Mily 2015 [18]	27.2 ± 8.0 / 26.7 ± 8.1″	177/111	288/260 daily for 8 weeks	ND	7/260 (2.7)
Nursyam 2006 [37]	29.85 ± 11.08 / 32.55 ± 11.6″	39/28	67/67 daily for 6 weeks	0/67 (0)	ND
Ralph 2013 [20]	29 (15–65)/ 26 (15–73) ^b^	131/69	200/164 at 0 & 4 weeks	19/123 (15.4)	2/164 (1.2)
Salahuddin 2013 [14]	27.8 ± 13.2 / 28.3 ± 14.1″	141/118	259/259 at 0 & 4 weeks	ND	ND
Tukvadze 2015 [39]	32.4 6 ± 10.6 / 34.1 6 ± 12.4″	127/72	199/192 weekly for 8 weeks,then fortnightly for 8 weeks	3/184 (1.6)	23/192 (12.0)
Wejse 2009 [38]	37 ± 13/ 38 ± 14”	222/145	367/241 at 0,20 & 32 weeks	82/240 (34.2)	ND
Johnson 2003 [40]	26.7 ± 7 / 27.4 ± 7″	75/35	110/110 daily for 4 ^1/2^ weeks	0/110 (0)	2/110 (1.8)
Mayanja-Kizza 2005 [41]	31.0 ± 7.1/ 31 ± 7.2″	113/74	187/187 daily for 8 weeks	187/187 (100)	ND
Pedral-Sampaio 2003 [44]	31.6 ± 10.9/ 28.0 ± 6.2″	16/15	31/31twice weekly for 4 weeks	0/31 (0)	ND
Willis 1996 [42]	28.1 ± 0.7 / 29.9 ± 1.0″	NM	107/107 daily for 16 weeks	107/107 (100)	ND
Mahakalkar 2017 [43]	31.96 ± 13.14 / 29.79 ± 11.12″	27/21	62/48 daily for 8 week	ND	NM
Ganmaa 2017 [15]	31 (23–44) / 35 (25–47) ^b^	256/134	390/390 at 0, 2, 4 & 6 weeks	ND	21/390 (5.4)

*I/C* Intervention & control groups, *Anti-TB* Anti-tuberculosis, *ND* not determined, *NM* not mentioned, *MDR-TB* multi-drug resistance tuberculosis, *yrs.* years, *HIV* human immuno deficiency virus

^a^: where denominators are less than total number of included participants, data are missing; “:mean age; ^b^:median age; ^c^: frequency at which adjunct treatment was administered; ^d^:number of participants included in the final analysis

### Description of outcomes of included studies

Twelve studies reported the sputum smear conversion rate [14–16, 18, 20, 21, 23, 44–50], whilst ten studies reported the sputum culture conversion rate [15, 16, 18, 20, 21, 25, 46–48, 50]. Eleven reported the time to sputum smear conversion [15, 18, 20, 21, 23, 25, 45, 47–50] and eight provided the time to sputum culture conversion [15, 16, 21, 25, 46–48, 50]. Six reported changes in BMI [14–16, 21, 23, 44], four presented changes in mean MUAC [14, 15, 23, 45], ten provided the data on chest radiograph change [14, 15, 18, 20, 21, 25, 44, 46–49] and five presented results on TB clinical score changes [14, 18, 20, 23, 45]. Eleven presented the incidence of adverse events [15, 16, 18, 20, 21, 25, 45–48, 50], nine indicated the changes in weight gain [14, 18, 20, 25, 45, 47–50], eight provided the incidence of all-cause mortality [15, 16, 20, 21, 45–47, 50], four exhibited changes in CRP and ESR [15, 18, 21, 23], and eight studies showed changes in other blood parameters such as total white cells, lymphocytes, neutrophils, monocytes and TNF-α [15, 18, 21, 23, 25, 44, 47, 48, 50]. Details on study outcome assessments are summarised in supplementary Tables 3 and 4 (Additional file 2).

### Risk of bias and influence analysis across and within studies

**Fig. 2:**
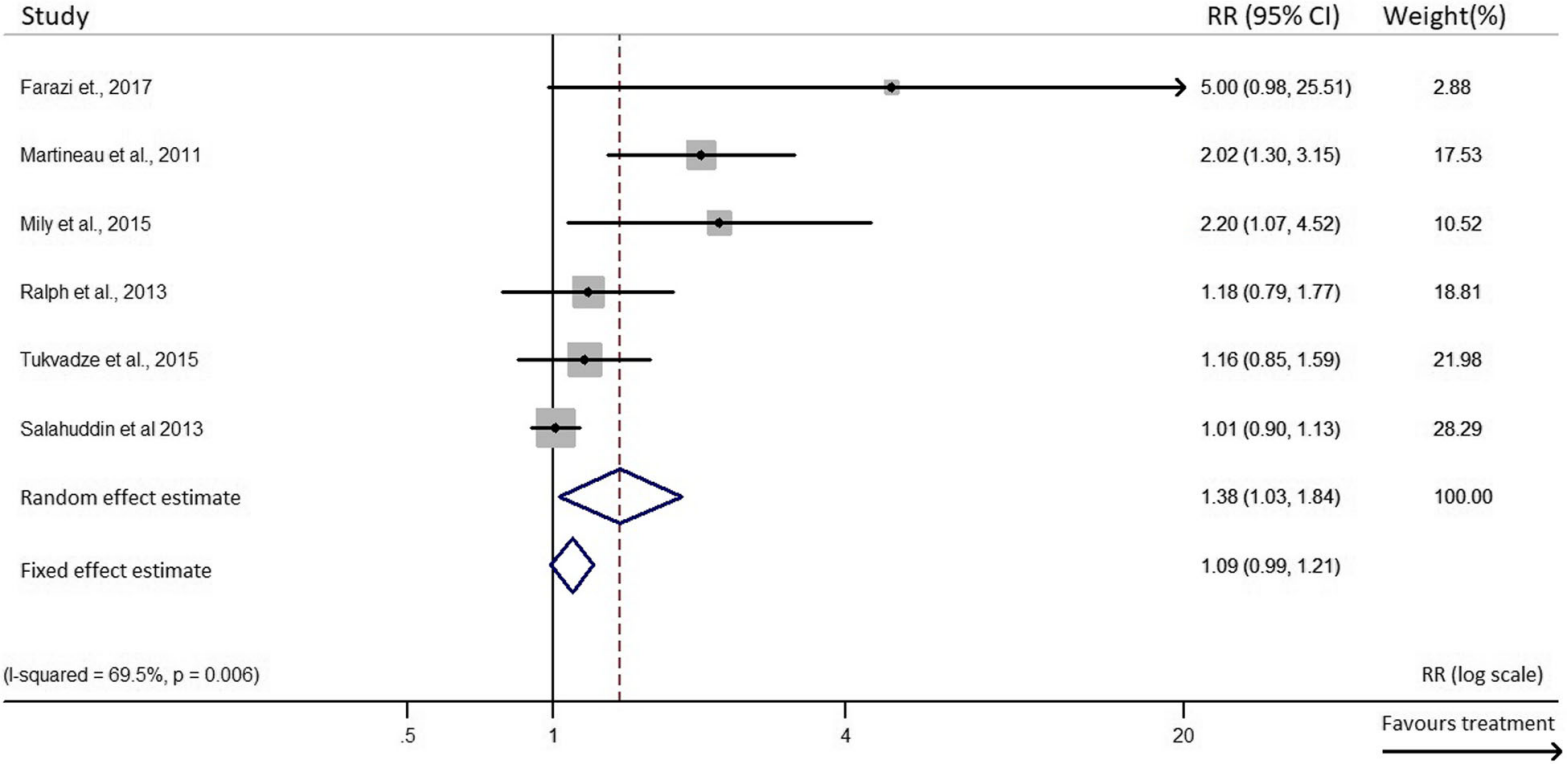
Forest plot of the random and fixed effect estimates of sputum smear conversion rate in vitamin D supplemented randomized controlled trials. *CI*: confidence interval; RR: relative risk NB: Weights are from random effects analysis.

**Fig. 3:**
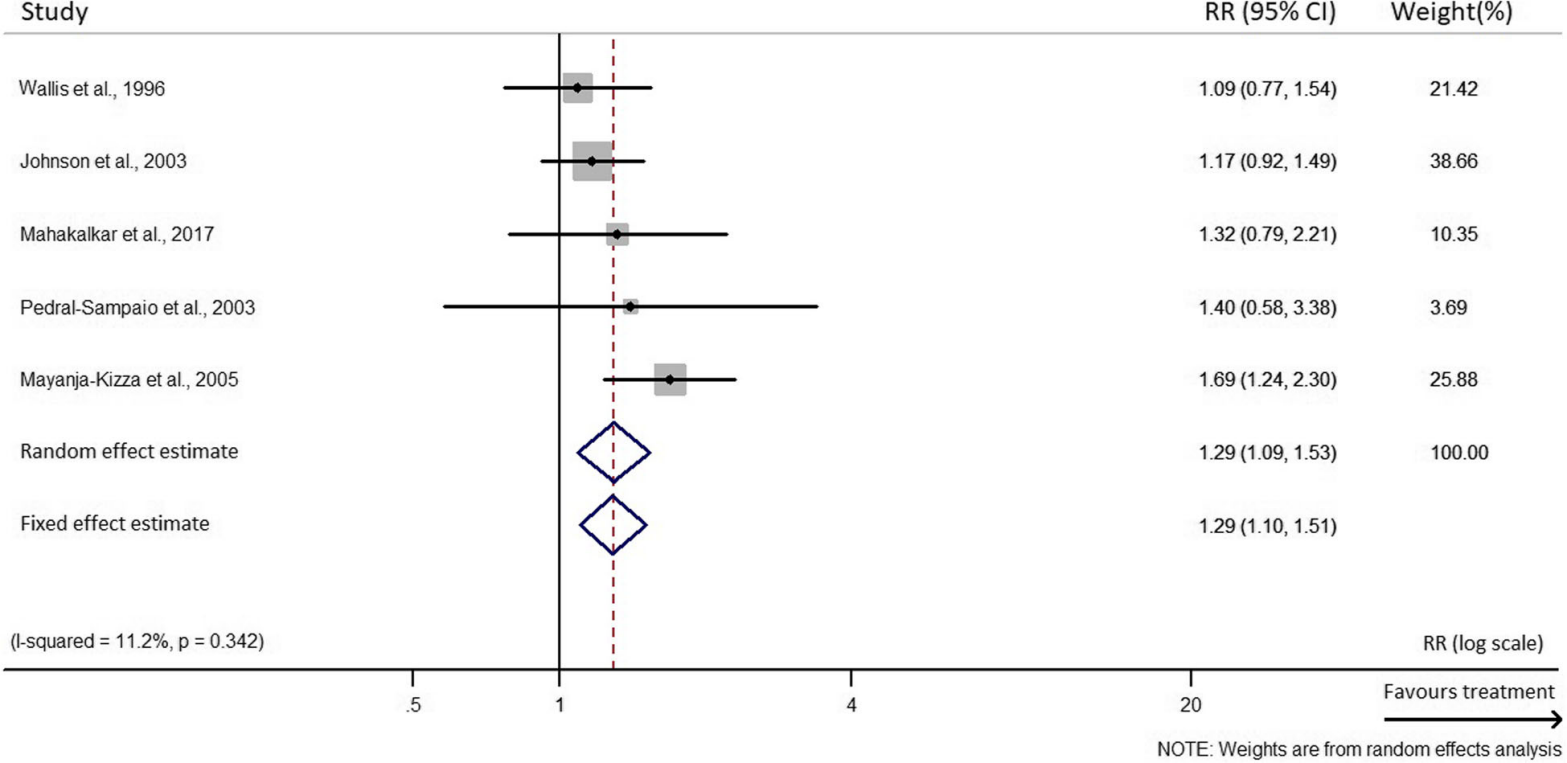
Forest plot of the random and fixed effect estimates of sputum smear conversion rate in other anti-inflammatory agents supplemented randomized controlled trials. *CI*: confidence interval; RR: relative risk

A funnel plot and Egger’s test for the outcome of rate of sputum smear conversion across studies did suggest a publication bias in trials involving vitamin D but not for those involving other anti-inflammatory treatments, in relation to this outcome (Additional file 2:Supplementary Figs. 1 & 2). An Egger’s test revealed an uneven spread of results of RCTs skewed at one side of the overall adjusted hazard ratio as shown in Supplementary Fig. 1. Seven RCTs [14, 15, 21, 25, 46–48] were at low risk of bias for all aspects analysed according to the Cochrane Collaboration Risk of Bias tool. The study led by Nursyam and colleagues [44] had an unclear risk bias due to selection bias (adequate sequence generation) and detection bias (blinding). Methods used in random sequence generation, blinding of participants, personnel and outcome assessments were not clearly or adequately described. Even though this could have affected the outcome, no evidence was found that it did. Selection bias was also observed in the trial by Pedral-Sampaio and colleagues, as it did not adequately describe a clear method used in random sequence generation [50]. RCTs conducted by Daley, Mahakalkar, Mily, Wejse and Ralph had an unclear risk of attrition bias due to incomplete outcome data [16, 18, 20, 45, 49] as a result of relatively high rates of loss to follow-up (LFU) of participants (> 20%). In addition, no evidence was found that it affected the overall final outcome of these studies, since the LFU was similar amongst groups. Some reasons for the LFU common to these studies included defaulting, death or transfer of patients to other locations of residence or for treatment. Details on the risk of bias assessment are provided in supplementary Table 2 (Additional file 2). There was a low statistical heterogeneity observed between/within treatments using other anti-inflammatory agents (I^2^ = 11.2%, *p* = 0.342), compared to the vitamin D supplementation group (I^2^ = 69.5%, *p* = 0.006) in the proportion of the rate of sputum smear conversion (Fig. 2 & Fig. 3). The high heterogeneity observed in the vitamin D supplementation group could be as a result of the different adjunct treatment doses administered, timing (Table 1) or duration of treatment (Table 3). The exclusion of the study from Salahuddin et al. [14] reduced the between-study heterogeneity to 53.3%. Overall, influence analysis did not detect any relevant difference in the relative risk (RR) estimate when excluding single studies. Likewise, no study was excluded due to dubious decisions as a result of the sensitivity analysis conducted to exclude any study**.**

### Primary outcome

The results represent the sputum smear conversion rate upon HDT treatment measured after a period of 4–8 weeks. It was observed that there was a 38% increased rate of sputum smear negativity among patients administered with vitamin D, compared to those receiving only the TB treatment (*RR* 1.38, 95% *CI* 1.03 to 1.84). There was moderate to high between-study heterogeneity observed in this group (*p* = 0.006 and *I*^2^ = 69.5%) (Fig. 2). Similarly, patients treated with other anti-inflammatory HDT agents had a 29% increased sputum smear conversion rate (*RR* 1.29, 95% *CI* 1.09 to 1.53). No heterogeneity was observed between studies in this group (*p* = 0.342 and *I*^2^ = 11.2%) (Fig. 3).

### Secondary outcomes

Sputum smear and culture time to conversion tended to be faster in the treatment group than in placebo in both vitamin D and other anti-inflammatory adjunct therapies in general. However, the sputum culture conversion rate at 8 weeks did not differ significantly and was similar between groups in the majority of trials. Administration of vitamin D did not influence the proportion of participants with sputum culture conversion at 8 weeks (treatment vs placebo, 150 of 190 vs. 148 of 200, respectively; adjusted odds ratio, 1.47; 95% CI, 0.88–2.45; *P* = 0.14). However, vitamin D did significantly accelerate sputum smear conversion (adjusted HR, 1.47; 95% CI,1.09–1.98) more in the treatment arm according to Ganmaa et al. [15] (Supplementary Table 4a).

Similarly, higher sputum clearance in the treatment group were reported by Farazi et al. (OR 0.20, 95% CI 0.04–1.02; *p* = 0.037) [23], Nursyam et al. (*p* = 0.002) [44] and Mayanja-Kizza et al. [47]. The proportion of participants with negative sputum culture at 4 weeks was significantly different for those randomised to vitamin D versus placebo (62% vs 37%, *p* = 0.001) but not at week 8 [47]. Mily et al. reported similar observations (OR 3.37, 95% CI 1.0–10.8; *p* = 0.041) [18]. According to Mahakalkar et al., there was a significant clearing of infiltration and reduction in cavity size in treatment compared to placebo (87.5% vs 33.33%, *p* < 0.05) and (*p* < 0.01) at 8 weeks, respectively [49]. Ganmaa et al. also reported vitamin D accelerated radiographic resolution (mean number of zones affected on chest radiograph at 8 weeks, treatment vs placebo, 5.48 vs 5.69 respectively; 95% *CI* for difference, − 0.06–0.77 zones; *p* = 0.02) [15]. There was also a 50% reduction in cavity size observed in the vitamin D arm compared to the placebo arm (*p* = 0.035) [14]. Most trials, however, did not observe significant clearing of infiltration or reduction in cavity size in treatment compared to placebo groups (Supplementary Table 4a).

There was no clinically significant difference in the change in MUAC between the vitamin D treatment and placebo group according to Salahuddin et al. [14]. However, Ganmaa et al. observed changes in MAUC after 8 weeks of treatment (adjusted mean difference,0.25,95% CI 0.08–0.43; p = 0.04) [15]. No significant effect was observed on concentrations of CRP and ESR at 8 weeks in three reported vitamin D supplemented trials [15, 18, 21]. However, an increase in CRP was seen in the placebo group at the end of 4 weeks (OR 2.98, 95% *CI* 1.04–8.52; *p* = 0.038) and 8 weeks (OR 3.33, 95% *CI* 1–11.14; *p* = 0.044) of treatment [23]. In addition, the ESR was observed to be higher in the placebo group compared to the Vitamin D group though not significantly (Vitamin D vs placebo, 33.5 ± 22.0 vs 36.3 ± 22.2 respectively) [18]. Similarly, there were no major significant effects of supplementary treatments observed with regards to weight gain and BMI for some of the vitamin D and other anti-inflammatory treatment groups. However, Salahuddin et al., Daley et al., and Mahakalkar et al. [14, 16, 49] reported highly significant weight changes in the vitamin D and N-acetylcysteine treatment groups compared to the placebo groups (3.75 vs 2.61; *p* < 0.009, 3·1; p < 0·0001, and 2.66 ± 0.18 vs − 0.02 ± 0.03; *p* < 0.001, respectively). Farazi et al. [23] also found marginal significant changes in BMI in favour of the vitamin D treatment group (24.66 ± 4.3 vs 22.31 ± 3.9, 95% *CI* 0.23–4.47) in comparison to Daley et al. [16], who observed increased BMI changes in favour of placebo group (0·087 kg/m^2^, p = 0·597) (Supplemetary Table 4b).

Changes in time to clinical improvement of the TB score were also similar in the two groups in three reported vitamin D supplemented trials included in this review. Farazi and colleagues [23], however, showed that the vitamin D group had a low TB score and better health related quality of life in comparison to placebo after 8 weeks of treatment (2.4 ± 1.5 vs 3.7 ± 1.7 95% CI -2.13 -- 0.47; *p* = 0.003 and 52.3 ± 9.6 vs 46.2 ± 10.1; *p* = 0.019, respectively). Adverse effects (AEs) were reported in the majority of the studies and there were no differences in the occurrence of most of the AEs between the study arms. However, in the study by Johnson et al., hyperpigmentation (recombinant human Interleukin vs placebo, 55 vs 1; *p* < 0.0001) and other adverse effects were observed to be higher in the treatment group [25]. No study reported mortality due to study intervention except for the study by Mayanja-Kizza et al. (prednisolone vs placebo, 17 vs 14 respectively; *p* = 0.28) [47]. Two studies reported that vitamin D administration was associated with reduced total white blood cell counts, neutrophil counts and monocyte counts [15, 21]. The lymphocyte to monocyte ratio was significantly higher in the intervention than in the control group at 8 weeks (3.52 vs 2.70, 95% *CI* for difference 0.16–1.11; *p* = 0.009) [21] and adjusted mean difference 0.4, 95% CI 0.2–0.6; *p* = 0.001 [15]. The median levels of TNF-α decreased by at least 50% from baseline values in the treatment arm compared to the placebo at 4 weeks (*p* < 0.001) [47]. There was a similar trend toward reduced TNF-α production in the pentoxifylline arm, although the difference between groups was not statistically significant (pentoxifylline vs placebo,0.4 vs 0.6 respectively; *p* = 0.27) [48] (Supplementary Table 4c)..

## Discussion

Sputum smear or culture conversion rate is a key biomarker used to predict TB cure. This biomarker has been found to be directly associated with relapse risk when regimens of equal duration were compared, and patients were on a modern short-course TB regimen [33, 51, 52]. The results of this systematic review and meta-analysis revealed that there was a faster sputum smear conversation rate among patients on adjunct therapy, compared to those on placebo between 4 and 8 weeks of anti-TB treatment. This finding was consistent with a meta-analysis published by Jingyan et al. in 2014 [31], which indicated that patients on vitamin D supplementation were at a reduced risk of remaining sputum positive after 6 weeks of anti-TB treatment compared to those in the control group, though not statistically significant. The non-significant effect observed in Jingyan’s study could be as a result of the limited number of trials included in the meta-analysis. Similar trends of non-significant sputum smear conversion rate changes were also observed from the results of other existing systematic reviews and aggregate data meta-analyses [30, 53, 54]. However, results from a meta-analysis of individual participant data from eight RCTs of adjunctive vitamin D in patients with PTB by Jolliffe et al. [6], which indicated a modestly accelerated sputum smear conversion rate in the treatment group, was consistent with our results.

The proportion of sputum smear or culture negativity did not differ significantly between groups across most of the trials, even though sputum smear or culture tended to convert to negative faster in the treatment group than in the placebo group. A similar trend was observed in the meta-analysis by Jolliffe et al. [6] and Riaz et al. [32], but not by Wu et al. [30] in the vitamin D supplemented group. Even though Wu at el [30]. reported significant differences in the overall effect in the proportion of sputum smear or culture conversion among the two study arms, no difference was observed between 4 and 8 weeks of treatment. This could be as a result of fewer studies included in the meta-analysis. Administration of daily prednisolone, given together with standard TB drug therapy, also reduced the proportion of positive cultures at 8 weeks from 15 to 2% according to Wallis [17].

Adverse effects observed in all the trials were deemed ‘unlikely to be related’ to study medications, as there were no differences in their occurrence between the study arms. There was no difference in adverse effect and mortality reported between the study arms for all adjunct treatments administered in the included studies, demonstrating the safety of their use. This finding was in accordance with previously reported systematic reviews and meta-analysis previously mentioned [30, 31]. Though there were some adverse effects such as hyperpigmentation, pain at injection site and erythema as a result of recombinant human interlukin administration, they were mild to moderate in severity and the duration was short. None of the subjects had to discontinue or required dose reduction as a result of these side effects, as the interlukin was generally well-tolerated. Also, even though Mayanja-Kizza et al. [47] reported mortality in the prednisolone arm of the study, only 1 was classified as probably related and 2 were classified as possibly related to the study intervention. There was no difference in short-term survival between the intervention and placebo arms. Notwithstanding, Critchley et al. indicated that corticosteroids could be effective in reducing mortality in persons with pulmonary tuberculosis, though more evidence is needed [55].

There was also limited evidence observed for the difference in changes in time to clinical improvement with regards to chest radiograph, MUAC, weight gain or BMI changes, and the overall TB score between the treatment and control groups. However, significant improvement of chest radiographs was observed between study arms in favour of the treatment group, where vitamin D and *N-acetylcysteine* were administered [15, 49], which was consistent with the findings of Wu et al. [30]. However, considering the limited number of studies that reported this outcome, more studies are required to either confirm this observation or not. Results on weight gain between the two study arms were consistent with other reported studies [30, 31].

Although no significant effect of improvement was observed in either group in the concentrations of CRP and ESR, which are biomarkers of inflammation [56, 57], the lymphocyte to monocyte ratio was shown to be significantly higher in the intervention administered with vitamin D than in the control group [15, 21]. This marker is a recognised biomarker of resolution of pulmonary inflammation in human and animal TB models [19, 58]. A trend toward reduced TNF-α production was also observed in participants treated with adjunctive prednisolone and pentoxifylline treatments. Pentoxifylline has been shown to suppress the synthesis of TNF—α from lipopolysaccharide-stimulated (LPS) human monocytes in cell cultures [59], inhibits LPS-induced TNF—α production from peripheral blood monocytes and alveolar macrophages, and can also inhibit the spontaneous TNF-α production [60]. Tumour necrosis factor-α is essential for immune control of infection with *Mtb* but has the potential to cause severe tissue damage [61, 62]. The positive effect of these adjunct treatments may yet play a critical role in improving lung function by the end of the TB treatment. It is possible that, by reducing lung inflammation, these adjunct treatments may improve drug penetration into affected tissue; thereby accelerating the response to standard TB drug therapy [19]. This may warrant additional studies to test this hypothesis.

This meta-analysis is different from most previously reported in that we included the most RCTs in the field of HDT adjunct treatment of TB, which were double-blinded and placebo-controlled. Secondly, it also included other adjunct treatments such as pentoxifylline, N-acetylcysteine and prednisolone, instead of vitamin D alone, which has been the focus of previously reported meta-analysis. Besides reporting the sputum conversion, which is an important indicator of treatment efficacy and success [63, 64], this meta-analysis also focused on the anti-inflammatory property of these treatment agents. This immunomodulatory property (effect) may have the potential of reducing residual lung damage, which is common after TB antimicrobial therapy and needs to be further investigated in a phase 2 or 3 clinical trial.

There are some limitations of this study: firstly, immunological characteristics were only determined in a minority of the studies, therefore,their immunomodulatory effect in TB should be further investigated to include more TB sensitive inflammatory biomarkers to confirm these potential properties. Secondly, the dose, timing and duration of treatment in both the vitamin D group and the other anti-inflammatory agents were different and not standardised. This could have influenced the results to some extent, such as the increased heterogeneity observed among the vitamin adjunct treatment. Thus, the need to standardise the dosing and also optimise the schedule of administration is vital for future clinical trials. Interpretation of the funnel plot in the vitamin D supplemented group indicated publication bias. This could be attributed to the number of studies included in the group, which may have influenced the results.

## Conclusion

In summary, this meta-analysis found that adjunctive treatment with vitamin D, pentoxifylline, N-acetylcysteine, prednisolone, recombinant human Interleukin and Rhu-GM-CSF may be a well-tolerated and effective addition to the treatment of TB patients. They were shown to significantly improve sputum conversion, chest radiographs appearance and also exhibit inflammation resolution properties. Thus, supplementing pulmonary TB patients on standard drug treatment with these HDT adjunct therapies may be justified. Also, considering the major economic burden associated with PTB, the use of low-cost adjunctive therapy such as vitamin D and pentoxifylline for such intervention could be regarded as cost-effective. However, future RCTs need to standardise the doses of these adjunct treatments and also optimise the schedule of administration. Secondly, more studies need to include more TB sensitive inflammatory biomarkers as an outcome measure, since resolution of inflammatory responses during TB therapy is associated with reducing high mortality in TB.

## Supplementary information

**Additional file 1.** Database search results from Medline.

**Additional file 2: Supplementary Table 1.** Quality assessment of included studies according to the Jadad scale**. Supplementary Table 2.** Risk of bias assessment of individual studies included. **Supplementary Table 3.** Frequency of outcome assessment and follow-up duration. **Supplementary Table 4a**. Outcome measurement at end point by allocation. **Supplementary Table 4b.** Outcome measurement at end point by allocation. **Supplementary Table 4c.** Outcome measurement at end point by allocation. **Supplementary Figure 1.** Funnel plot for aggregate patient data meta-analysis of sputum smear conversion rate conversion in vitamin D supplemented randomized controlled trials. **Supplementary Figure 2.** Funnel plot for aggregate patient data meta-analysis of sputum smear conversion rate in other anti-inflammatory HDT agents supplemented randomized controlled trials.

## Data Availability

All data generated or analysed during this study are included in this published article (and its supplementary information files).

## Abbreviations

TB: Tuberculosis
HDT: Host-directed therapy
RR: Relative risk
TNF-α: Tumour necrosis factor alpha
IFN-γ: interferon gamma
Mtb: Mycobacteria tuberculosis
MDR: Multi drug-resistant
XDR: Extensive drug-resistant
RCT: Randomized control trial
BMI: Body mass index
MUAC: Mid-upper arm circumference
CRP: C-reactive protein
ESR: Erythrocyte sedimentation rate
PTB: Pulmonary tuberculosis
MeSH: Medical subject heading
PRISMA: Preferred reporting items for systematic reviews and meta-analysis
HRZE: Isoniazid, rifampicin, pyrazinamide, ethambutol
RH: Rifampicin and isoniazid
LFU: Loss to follow-up
OR: Odds ratio
SD: Standard deviation
95% CI: 95% Confidnce interval

## References

[1] WHO (World Health Organization). Global tuberculosis report 2018. 2018 [cited 2019 18/02/2019]; Available from: https://www.who.int/tb/publications/global_report/en/.

[2] Kolloli A, Subbian S (2017). Host-directed therapeutic strategies for tuberculosis. Frontiers in medicine.

[3] Hawn TR (2013). Host-directed therapeutics for tuberculosis: can we harness the host?. Microbiol Mol Biol Rev.

[4] Manjelievskaia J (2016). Drug-resistant TB: deadly, costly and in need of a vaccine. Trans R Soc Trop Med Hyg.

[5] Wallis RS, Hafner R (2015). Advancing host-directed therapy for tuberculosis. Nat Rev Immunol.

[6] Jolliffe DA (2019). Adjunctive vitamin D in tuberculosis treatment: meta-analysis of individual participant data. Eur Respir J.

[7] Afzal A (2018). Efficacy of vitamin D supplementation in achieving an early sputum conversion in smear positive pulmonary tuberculosis. Pakistan J Med Sci.

[8] Fullerton JN, O’Brien AJ, Gilroy DW (2014). Lipid mediators in immune dysfunction after severe inflammation. Trends Immunol.

[9] WHO (World Health Organization), Treatment of tuberculosis: guidelines. 4th edition ed, ed. WHO/HTM/TB/2009.420. 2010, Geneva: World Health Organization.

[10] Kroesen VM (2017). Non-steroidal anti-inflammatory drugs as host-directed therapy for tuberculosis: a systematic review. Front Immunol.

[11] Stek C, et al. The immune mechanisms of lung parenchymal damage in tuberculosis and the role of host-directed therapy. Front Microbiol. 2018;9. 10.3389/fmicb.2018.02603 PMC6218626 30425706

[12] Ivanyi, J. and A. Zumla, Nonsteroidal antiinflammatory drugs for adjunctive tuberculosis treatment*.* J Infectious Diseases, 2013: p. jit153. 10.1093/infdis/jit153 23564637

[13] Bekele A (2018). Daily adjunctive therapy with vitamin D 3 and phenylbutyrate supports clinical recovery from pulmonary tuberculosis: a randomized controlled trial in Ethiopia. J Intern Med.

[14] Salahuddin N (2013). Vitamin D accelerates clinical recovery from tuberculosis: results of the SUCCINCT Study [Supplementary Cholecalciferol in recovery from tuberculosis]. A randomized, placebo-controlled, clinical trial of vitamin D supplementation in patients with pulmonary tuberculosis’. BMC Infectious Dis.

[15] Ganmaa D (2017). High-dose vitamin D3 during tuberculosis treatment in Mongolia. A randomized controlled trial. Am J Respir Crit Care Med.

[16] Daley P (2015). Adjunctive vitamin D for treatment of active tuberculosis in India: a randomised, double-blind, placebo-controlled trial. Lancet Infect Dis.

[17] Wallis, R.S. Corticosteroid effects on sputum culture in pulmonary tuberculosis: a meta-regression analysis. In Open forum infectious diseases. 2014. Oxford University Press. 10.1093/ofid/ofu020 PMC4324181 25734093

[18] Mily A (2015). Significant effects of oral phenylbutyrate and vitamin D3 adjunctive therapy in pulmonary tuberculosis: a randomized controlled trial. PLoS One.

[19] Coussens AK (2012). Vitamin D accelerates resolution of inflammatory responses during tuberculosis treatment. Proc Natl Acad Sci.

[20] Ralph AP (2013). L-arginine and vitamin D adjunctive therapies in pulmonary tuberculosis: a randomised, double-blind, placebo-controlled trial. PLoS One.

[21] Martineau AR (2011). High-dose vitamin D 3 during intensive-phase antimicrobial treatment of pulmonary tuberculosis: a double-blind randomised controlled trial. Lancet.

[22] Liu PT (2007). Cutting edge: vitamin D-mediated human antimicrobial activity against Mycobacterium tuberculosis is dependent on the induction of cathelicidin. J Immunol.

[23] Farazi A, Didgar F, Sarafraz A (2017). The effect of vitamin D on clinical outcomes in tuberculosis. Egypt J Chest Dis Tuberculosis.

[24] Bilaçeroğlu S (1999). Prednisolone: a beneficial and safe adjunct to antituberculosis treatment? A randomized controlled trial. Int J Tuberculosis Lung Disease.

[25] Johnson JL (2003). Randomized trial of adjunctive interleukin-2 in adults with pulmonary tuberculosis. Am J Respir Crit Care Med.

[26] Teskey G (2018). The synergistic effects of the glutathione precursor*,* NAC and first-line antibiotics in the granulomatous response against *Mycobacterium tuberculosis*. Front Immunol.

[27] De Rosa S (2000). N-acetylcysteine replenishes glutathione in HIV infection. Eur J Clin Investig.

[28] Peterson JD (1998). Glutathione levels in antigen-presenting cells modulate Th1 versus Th2 response patterns. Proc Natl Acad Sci.

[29] Denis M, Ghadirian E (1990). Granulocyte-macrophage colony-stimulating factor restricts growth of tubercle bacilli in human macrophages. Immunol Lett.

[30] Wu H-X (2018). Effects of vitamin D supplementation on the outcomes of patients with pulmonary tuberculosis: a systematic review and meta-analysis. BMC Pulmonary Med.

[31] Jingyan, X., et al., Impact of vitamin D supplementation on the outcome of tuberculosis treatment: a systematic review and meta-analysis of randomized controlled trials. 2014, LWW. 25189958

[32] Riaz H, et al. Vitamin D as a supplementary agent in the treatment of pulmonary tuberculosis: a systematic review and meta-analysis of randomized controlled trials. 2013, Eur Respiratory Soc.

[33] Frette C (1993). Assessment of new sterilizing drugs for treating pulmonary tuberculosis by culture at 2 months.[correspondence]. Am Rev Respir Dis.

[34] Wallis RS (2010). Biomarkers for tuberculosis disease activity, cure, and relapse. Lancet Infect Dis.

[35] Wallis RS (2013). Month 2 culture status and treatment duration as predictors of tuberculosis relapse risk in a meta-regression model. PLoS One.

[36] Stewart LA (2015). Preferred reporting items for a systematic review and meta-analysis of individual participant data: the PRISMA-IPD statement. Jama.

[37] Higgins JP (2011). The Cochrane Collaboration’s tool for assessing risk of bias in randomised trials. Bmj.

[38] Higgins JP, G.S., Cochrane Handbook for Systematic Reviews of Interventions 5.3.0. Ed. the Cochrane collaboration, 2014. 2014, Oxford England: Oxford.

[39] Jadad AR (1996). Assessing the quality of reports of randomized clinical trials: is blinding necessary?. Control Clin Trials.

[40] Kayigamba FR (2012). Sputum completion and conversion rates after intensive phase of tuberculosis treatment: an assessment of the Rwandan control program. BMC Research Notes.

[41] Peters JL (2008). Contour-enhanced meta-analysis funnel plots help distinguish publication bias from other causes of asymmetry. J Clin Epidemiol.

[42] Egger M (1997). Bias in meta-analysis detected by a simple, graphical test. Bmj.

[43] Higgins J, Thompson S (2002). Quantifying heterogeneity in a meta-analysis. Stat Med.

[44] Nursyam EW, Amin Z, Rumende CM (2006). The effect of vitamin D as supplementary treatment in patients with moderately advanced pulmonary tuberculous lesion. Hemoglobin.

[45] Wejse C (2009). Vitamin D as supplementary treatment for tuberculosis: a double-blind, randomized, placebo-controlled trial. Am J Respir Crit Care Med.

[46] Tukvadze N (2015). High-dose vitamin D3 in adults with pulmonary tuberculosis: a double-blind randomized controlled trial. Am J Clin Nutr.

[47] Mayanja-Kizza H (2005). Immunoadjuvant prednisolone therapy for HIV-associated tuberculosis: a phase 2 clinical trial in Uganda. J Infect Dis.

[48] Wallis R (1996). Pentoxifylline therapy in human immunodeficiency virus—seropositive persons with tuberculosis: a randomized*,* Controlled Trial. J Infectious Diseases.

[49] Mahakalkar SM (2017). N-acetylcysteine as an add-on to directly observed therapy short-I therapy in fresh pulmonary tuberculosis patients: a randomized, placebo-controlled, double-blinded study. Perspectives Clin Res.

[50] Pedral-Sampaio DB (2003). Use of Rhu-GM-CSF in pulmonary tuberculosis patients: results of a randomized clinical trial. Braz J Infect Dis.

[51] Phillips PP, Fielding K, Nunn AJ (2013). An evaluation of culture results during treatment for tuberculosis as surrogate endpoints for treatment failure and relapse. PLoS One.

[52] Wallis RS (2009). Biomarkers for tuberculosis disease activity, cure, and relapse. Lancet Infect Dis.

[53] Grobler L, Nagpal S, Sudarsanam TD, Sinclair D. Nutritional supplements for people being treated for active tuberculosis. Cochrane Database Syst Rev. 2016;(6):CD006086. 10.1002/14651858.CD006086.pub4. 10.1002/14651858.CD006086.pub4 PMC4981643 27355911

[54] Wallis, R.S. and A. Zumla. Vitamin D as adjunctive host-directed therapy in tuberculosis: a systematic review. In Open forum infectious diseases. 2016. Oxford University Press. 10.1093/ofid/ofw151 PMC5084719 27800526

[55] Critchley JA (2013). Corticosteroids for prevention of mortality in people with tuberculosis: a systematic review and meta-analysis. Lancet Infect Dis.

[56] Sands BE (2015). Biomarkers of inflammation in inflammatory bowel disease. Gastroenterology.

[57] Sepúlveda-Delgado J (2017). Inflammatory biomarkers, disease activity index, and self-reported disability may be predictors of chronic arthritis after chikungunya infection: brief report. Clin Rheumatol.

[58] Sabin, F.R., C.A. Doan, and R.S. Cunningham. Studies of the blood in experimental tuberculosis: the monocyte-lymphocyte ratio; the anemia-leucopenia phase. in Transactions of the 22nd Annual Meeting of the National Tuberculosis Association. 1926.

[59] Spatafora M (1994). Theophylline suppresses the release of tumour necrosis factor-alpha by blood monocytes and alveolar macrophages. Eur Respir J.

[60] Marques LJ (1999). Pentoxifylline inhibits TNF-α production from human alveolar macrophages. Am J Respir Crit Care Med.

[61] Mootoo A (2009). TNF-α in tuberculosis: a cytokine with a Split personality. Inflammation Allergy-Drug Targets (Formerly Current Drug Targets-Inflammation & Allergy).

[62] Zumla A (2015). Inflammation and tuberculosis: host-directed therapies. J Intern Med.

[63] Su W (2011). Role of 2-month sputum smears in predicting culture conversion in pulmonary tuberculosis. Eur Respir J.

[64] Shibabaw A (2018). Time to sputum smear and culture conversions in multidrug resistant tuberculosis at University of Gondar Hospital, Northwest Ethiopia. PloS one.

