# Supplementary material for: The effects of anti-inflammatory agents as host-directed adjunct treatment of tuberculosis in humans: a systematic review and meta-analysis
Source: Respir Res. 2020 Aug 26;21:223. doi: 10.1186/s12931-020-01488-9 (PMC7448999; doi:10.1186/s12931-020-01488-9)
Supplement: Supplementary file 1 — Additional file 1. Database search results from Medline. [file 12931_2020_1488_MOESM1_ESM.pdf]

1: Singh B, Cocker D, Ryan H, Sloan DJ. Linezolid for drug-resistant pulmonary
tuberculosis. Cochrane Database Syst Rev. 2019 Mar 20;3:CD012836. doi:
10.1002/14651858.CD012836.pub2. Review. PubMed PMID: 30893466; PubMed Central
PMCID: PMC6426281.

2: Lyakurwa D, Lyimo J, Mleoh L, Riziki K, Lupinda M, Mpondo BC. Successful
treatment of XDR-TB patient in Tanzania: report of the first XDR-TB patient. Trop
Doct. 2019 Mar 18:49475519833554. doi: 10.1177/0049475519833554. [Epub ahead of
print] PubMed PMID: 30885056.

3: Sarin R, Singla N, Vohra V, Singla R, Puri MM, Munjal S, Khalid UK, Myneedu
VP, Kumar Verma A, Mathuria KK. Initial experience of bedaquiline implementation
under the National TB Programme at NITRD, Delhi, India. Indian J Tuberc. 2019
Jan;66(1):209-213. doi: 10.1016/j.ijtb.2019.02.009. Epub 2019 Feb 27. PubMed
PMID: 30878071.

4: Sarin R, Vohra V, Singla N, Singla R, Puri MM, Munjal SK, Khalid UK, Myneedu
VP, Verma A, Mathuria KK. Early efficacy and safety of Bedaquiline and Delamanid
given together in a "Salvage Regimen" for treatment of drug-resistant
tuberculosis. Indian J Tuberc. 2019 Jan;66(1):184-188. doi:
10.1016/j.ijtb.2019.02.006. Epub 2019 Feb 27. PubMed PMID: 30878066.

5: Shirley M. Amikacin Liposome Inhalation Suspension: A Review in Mycobacterium
avium Complex Lung Disease. Drugs. 2019 Apr;79(5):555-562. doi:
10.1007/s40265-019-01095-z. Erratum in: Drugs. 2019 Apr 3;:. PubMed PMID:
30877642.

6: Savioli MTG, Morrone N, Santoro I. Primary bacillary resistance in
multidrug-resistant tuberculosis and predictive factors associated with cure at a
referral center in SÃ£o Paulo, Brazil. J Bras Pneumol. 2019 Mar
11;45(2):e20180075. doi: 10.1590/1806-3713/e20180075. English, Portuguese. PubMed
PMID: 30864608.

7: Moon SM, Choe J, Jhun BW, Jeon K, Kwon OJ, Huh HJ, Lee NY, Daley CL, Koh WJ.
Treatment with a macrolide-containing regimen for Mycobacterium kansasii
pulmonary disease. Respir Med. 2019 Mar;148:37-42. doi:
10.1016/j.rmed.2019.01.012. Epub 2019 Jan 30. PubMed PMID: 30827472.

8: Padmapriyadarsini C, Bhavani PK, Natrajan M, Ponnuraja C, Kumar H, Gomathy SN,
Guleria R, Jawahar SM, Singh M, Balganesh T, Swaminathan S. Evaluation of
metformin in combination with rifampicin containing antituberculosis therapy in
patients with new, smear-positive pulmonary tuberculosis (METRIF): study protocol
for a randomised clinical trial. BMJ Open. 2019 Mar 1;9(3):e024363. doi:
10.1136/bmjopen-2018-024363. PubMed PMID: 30826761; PubMed Central PMCID:
PMC6429929.

9: Lai JML, Yang SL, Avoi R. Treating More with Less: Effectiveness and Event
Outcomes of Antituberculosis Fixed-dose Combination Drug versus Separate-drug
Formulation (Ethambutol, Isoniazid, Rifampicin and Pyrazinamide) for Pulmonary
Tuberculosis Patients in Real-world Clinical Practice. J Glob Infect Dis. 2019
Jan-Mar;11(1):2-6. doi: 10.4103/jgid.jgid_50_18. PubMed PMID: 30814828; PubMed
Central PMCID: PMC6380106.

10: Perumal R, Padayatchi N, Yende-Zuma N, Naidoo A, Govender D, Naidoo K. A
moxifloxacin-based regimen for the treatment of recurrent drug-sensitive
pulmonary tuberculosis: An open-label randomised controlled trial. Clin Infect
Dis. 2019 Feb 27. pii: ciz152. doi: 10.1093/cid/ciz152. [Epub ahead of print]
PubMed PMID: 30809633.

11: Nandasena S, Senavirathna C, Munasinghe C, Wijesena C, Sucharitharathna R.
Characteristics and sputum conversion of tuberculosis (TB) patients in Kalutara,
Sri Lanka. Indian J Tuberc. 2019 Jan;66(1):76-80. doi:
10.1016/j.ijtb.2018.04.008. Epub 2018 Apr 13. PubMed PMID: 30797288.

12: Soedarsono S, Subiantoro MC. Changes of CRP serum levels in pulmonary TB
patients with AFB smear-positive sputum before and two months after receiving
anti-tuberculosis drug treatment. Indian J Tuberc. 2019 Jan;66(1):134-138. doi:
10.1016/j.ijtb.2018.07.007. Epub 2018 Oct 12. PubMed PMID: 30797271.

13: Bhatt R, Chopra K, Vashisht R. Impact of integrated psycho-socio-economic
support on treatment outcome in drug resistant tuberculosis - A retrospective
cohort study. Indian J Tuberc. 2019 Jan;66(1):105-110. doi:
10.1016/j.ijtb.2018.05.020. Epub 2018 Jun 13. PubMed PMID: 30797265.

14: Cruz AT, Starke JR. Window Period Prophylaxis for Children Exposed to
Tuberculosis, Houston, Texas, USA, 2007-2017. Emerg Infect Dis. 2019
Mar;25(3):523-528. doi: 10.3201/eid2503.181596. PubMed PMID: 30789136; PubMed
Central PMCID: PMC6390765.

15: Ma Y, Che NY, Liu YH, Shu W, Du J, Xie SH, Li L. The joint impact of smoking
plus alcohol drinking on treatment of pulmonary tuberculosis. Eur J Clin
Microbiol Infect Dis. 2019 Apr;38(4):651-657. doi: 10.1007/s10096-019-03489-z.
Epub 2019 Feb 15. PubMed PMID: 30771122.

16: Tsuyuguchi K, Sasaki Y, Mitarai S, Kurosawa K, Saito Y, Koh T. Safety,
efficacy, and pharmacokinetics of bedaquiline in Japanese patients with pulmonary
multidrug-resistant tuberculosis: An interim analysis of an open-label, phase 2
study. Respir Investig. 2019 Feb 7. pii: S2212-5345(18)30240-5. doi:
10.1016/j.resinv.2019.01.001. [Epub ahead of print] PubMed PMID: 30745177.

17: Tokairin Y, Nakajima Y, Kawada K, Hoshino A, Okada T, Ryotokuji T, Ogo T,
Okuda M, Kume Y, Kawamura Y, Yamaguchi K, Nagai K, Kawano T, Kinugasa Y. A
feasibility study of mediastinoscopic radical esophagectomy for thoracic
esophageal cancer from the viewpoint of the dissected mediastinal lymph nodes
validated with thoracoscopic procedure: a prospective clinical trial. Esophagus.

2019 Apr;16(2):214-219. doi: 10.1007/s10388-018-00656-7. Epub 2019 Feb 8. PubMed
PMID: 30737707.

18: Jolliffe DA, Ganmaa D, Wejse C, Raqib R, Haq MA, Salahuddin N, Daley PK,
Ralph AP, Ziegler TR, Martineau AR. Adjunctive vitamin D in tuberculosis
treatment: meta-analysis of individual participant data. Eur Respir J. 2019 Mar
7;53(3). pii: 1802003. doi: 10.1183/13993003.02003-2018. Print 2019 Mar. PubMed
PMID: 30728208.

19: Bongiolatti S, Gonfiotti A, Viggiano D, Borgianni S, Politi L, Crisci R,
Curcio C, Voltolini L; Italian VATS Group. Risk factors and impact of conversion
from VATS to open lobectomy: analysis from a national database. Surg Endosc. 2019
Jan 31. doi: 10.1007/s00464-019-06682-5. [Epub ahead of print] PubMed PMID:
30706153.

20: Kreisel CF, Passannante MR, Lardizabal AA. The Negative Clinical Impact of
Diabetes on Tuberculosis: A Cross-Sectional Study in New Jersey. J Endocr Soc.
2018 Nov 14;3(1):62-68. doi: 10.1210/js.2018-00203. eCollection 2019 Jan 1.
PubMed PMID: 30697601; PubMed Central PMCID: PMC6344343.

21: McCurtain JL, Gilbertsen AJ, Evert C, Williams BJ, Hunter RC. Agmatine
accumulation by Pseudomonas aeruginosa clinical isolates confers antibiotic
tolerance and dampens host inflammation. J Med Microbiol. 2019 Mar;68(3):446-455.
doi: 10.1099/jmm.0.000928. Epub 2019 Jan 22. PubMed PMID: 30688634.

22: de Melo MGM, Mesquita EDD, Oliveira MM, da Silva-Monteiro C, Silveira AKA,
Malaquias TS, Dutra TCP, Galliez RM, Kritski AL, Silva EC; Rede-TB Study Group.
Imbalance of NET and Alpha-1-Antitrypsin in Tuberculosis Patients Is Related With
Hyper Inflammation and Severe Lung Tissue Damage. Front Immunol. 2019 Jan
10;9:3147. doi: 10.3389/fimmu.2018.03147. eCollection 2018. PubMed PMID:
30687336; PubMed Central PMCID: PMC6335334.

23: Reimann M, Schaub D, Kalsdorf B, Runge C, Carballo PS, Terhalle E, Salzer
HJF, Lange C, Heyckendorf J. Cigarette smoking and culture conversion in patients
with susceptible and M/XDR-TB. Int J Tuberc Lung Dis. 2019 Jan 1;23(1):93-98.
doi: 10.5588/ijtld.18.0354. PubMed PMID: 30674380.

24: Lee M, Mok J, Kim DK, Shim TS, Koh WJ, Jeon D, Lee T, Lee SH, Kim JS, Park
JS, Lee JY, Kim SY, Lee JH, Jo KW, Jhun BW, Kang YA, Ahn JH, Kim CK, Shin S, Song
T, Shin SJ, Kim YR, Ahn H, Hahn S, Won HJ, Jang JY, Cho SN, Yim JJ. Delamanid,
linezolid, levofloxacin, and pyrazinamide for the treatment of patients with
fluoroquinolone-sensitive multidrug-resistant tuberculosis (Treatment Shortening
of MDR-TB Using Existing and New Drugs, MDR-END): study protocol for a phase
II/III, multicenter, randomized, open-label clinical trial. Trials. 2019 Jan
16;20(1):57. doi: 10.1186/s13063-018-3053-1. PubMed PMID: 30651149; PubMed
Central PMCID: PMC6335682.

25: Warsinske HC, Rao AM, Moreira FMF, Santos PCP, Liu AB, Scott M, Malherbe ST,
Ronacher K, Walzl G, Winter J, Sweeney TE, Croda J, Andrews JR, Khatri P.
Assessment of Validity of a Blood-Based 3-Gene Signature Score for Progression
and Diagnosis of Tuberculosis, Disease Severity, and Treatment Response. JAMA
Netw Open. 2018 Oct 5;1(6):e183779. doi: 10.1001/jamanetworkopen.2018.3779.
PubMed PMID: 30646264; PubMed Central PMCID: PMC6324428.

26: Dousa KM, Hamad A, Albirair M, Al Soub H, Elzouki AN, Alwakeel MI, Thiel BA,
Johnson JL. Impact of Diabetes Mellitus on the Presentation and Response to
Treatment of Adults With Pulmonary Tuberculosis in Qatar. Open Forum Infect Dis.
2018 Dec 19;6(1):ofy335. doi: 10.1093/ofid/ofy335. eCollection 2019 Jan. PubMed
PMID: 30631793; PubMed Central PMCID: PMC6324545.

27: von Groote-Bidlingmaier F, Patientia R, Sanchez E, Balanag V Jr, Ticona E,

Segura P, Cadena E, Yu C, Cirule A, Lizarbe V, Davidaviciene E, Domente L,
Variava E, Caoili J, Danilovits M, Bielskiene V, Staples S, Hittel N, Petersen C,
Wells C, Hafkin J, Geiter LJ, Gupta R. Efficacy and safety of delamanid in
combination with an optimised background regimen for treatment of
multidrug-resistant tuberculosis: a multicentre, randomised, double-blind,
placebo-controlled, parallel group phase 3 trial. Lancet Respir Med. 2019
Mar;7(3):249-259. doi: 10.1016/S2213-2600(18)30426-0. Epub 2019 Jan 7. PubMed
PMID: 30630778.

28: Nkurunziza J, Karstaedt AS, Louw R, Padanilam X. Treatment outcomes of pre-
and extensively drug-resistant tuberculosis in Johannesburg, South Africa. Int J
Tuberc Lung Dis. 2018 Dec 1;22(12):1469-1474. doi: 10.5588/ijtld.18.0205. PubMed
PMID: 30606319.

29: Mukisa J, Kawooya I, Nangendo J, Nalutaaya A, Nyamwiza J, Sam A, Ssenyonga R,
Worodria W, Mupere E. Male gender and duration of anti-tuberculosis treatment are
associated with hypocholesterolemia in adult pulmonary tuberculosis patients in
Kampala, Uganda. Afr Health Sci. 2018 Sep;18(3):479-487. doi:
10.4314/ahs.v18i3.3. PubMed PMID: 30602978; PubMed Central PMCID: PMC6307033.

30: Htun YM, Khaing TMM, Aung NM, Yin Y, Myint Z, Aung ST, Soonthornworasiri N,
Silachamroon U, Kasetjaroen Y, Kaewkungwal J. Delay in treatment initiation and
treatment outcomes among adult patients with multidrug-resistant tuberculosis at
Yangon Regional Tuberculosis Centre, Myanmar: A retrospective study. PLoS One.
2018 Dec 31;13(12):e0209932. doi: 10.1371/journal.pone.0209932. eCollection 2018.
PubMed PMID: 30596734; PubMed Central PMCID: PMC6312206.

31: Kwon YS, Koh WJ, Daley CL. Treatment of Mycobacterium avium Complex Pulmonary
Disease. Tuberc Respir Dis (Seoul). 2019 Jan;82(1):15-26. doi:
10.4046/trd.2018.0060. Review. PubMed PMID: 30574687; PubMed Central PMCID:
PMC6304322.

32: Choi JY, Jhun BW, Hyun SH, Chung MJ, Koh WJ. (18)F-Fluorodeoxyglucose
Positron Emission Tomography/Computed Tomography for Assessing Treatment Response
of Pulmonary Multidrug-Resistant Tuberculosis. J Clin Med. 2018 Dec 17;7(12).
pii: E559. doi: 10.3390/jcm7120559. PubMed PMID: 30562940; PubMed Central PMCID:
PMC6307081.

33: Davies Forsman L, Jonsson J, Wagrell C, Werngren J, MansjÃ¶ M, Wijkander M,
Groenheit R, Hammar U, Giske CG, SchÃ¶n T, Bruchfeld J. Minimum inhibitory
concentrations of fluoroquinolones and pyrazinamide susceptibility correlate to
clinical improvement in MDR-TB patients - a nationwide Swedish cohort study over
two decades. Clin Infect Dis. 2018 Dec 18. doi: 10.1093/cid/ciy1068. [Epub ahead
of print] PubMed PMID: 30561569.

34: Ige OM, Oladokun RE. Time to sputum culture conversion and treatment outcome
among the first cohort of multidrug resistant tuberculosis patients in a high
burden country. Indian J Tuberc. 2018 Oct;65(4):322-328. doi:
10.1016/j.ijtb.2018.07.006. Epub 2018 Aug 9. PubMed PMID: 30522620.

35: Diarra B, Kone M, Togo ACG, Sarro YDS, Cisse AB, Somboro A, Degoga B,
Tolofoudie M, Kone B, Sanogo M, Baya B, Kodio O, Maiga M, Belson M, Orsega S,
Krit M, Dao S, Maiga II, Murphy RL, Rigouts L, Doumbia S, Diallo S, de Jong BC.
Mycobacterium africanum (Lineage 6) shows slower sputum smear conversion on
tuberculosis treatment than Mycobacterium tuberculosis (Lineage 4) in Bamako,
Mali. PLoS One. 2018 Dec 12;13(12):e0208603. doi: 10.1371/journal.pone.0208603.
eCollection 2018. PubMed PMID: 30540823; PubMed Central PMCID: PMC6291124.

36: Akinsola OJ, Yusuf OB, Ige OM, Okonji PE. Models for Predicting Time to
Sputum Conversion Among Multi-Drug Resistant Tuberculosis Patients in Lagos,
South-West Nigeria. Front Public Health. 2018 Nov 27;6:347. doi:
10.3389/fpubh.2018.00347. eCollection 2018. PubMed PMID: 30538978; PubMed Central

PMCID: PMC6277524.

37: Deshpande D, Pasipanodya JG, Mpagama SG, Bendet P, Srivastava S, Koeuth T,
Lee PS, Bhavnani SM, Ambrose PG, Thwaites G, Heysell SK, Gumbo T. Levofloxacin
Pharmacokinetics/Pharmacodynamics, Dosing, Susceptibility Breakpoints, and
Artificial Intelligence in the Treatment of Multidrug-resistant Tuberculosis.
Clin Infect Dis. 2018 Nov 28;67(suppl_3):S293-S302. doi: 10.1093/cid/ciy611.
PubMed PMID: 30496461; PubMed Central PMCID: PMC6260169.

38: Pasipanodya JG, Smythe W, Merle CS, Olliaro PL, Deshpande D, Magombedze G,
McIlleron H, Gumbo T. Artificial intelligence-derived 3-Way
Concentration-dependent Antagonism of Gatifloxacin, Pyrazinamide, and Rifampicin
During Treatment of Pulmonary Tuberculosis. Clin Infect Dis. 2018 Nov
28;67(suppl_3):S284-S292. doi: 10.1093/cid/ciy610. PubMed PMID: 30496458.

39: Deshpande D, Pasipanodya JG, Mpagama SG, Srivastava S, Bendet P, Koeuth T,
Lee PS, Heysell SK, Gumbo T. Ethionamide
Pharmacokinetics/Pharmacodynamics-derived Dose, the Role of MICs in Clinical
Outcome, and the Resistance Arrow of Time in Multidrug-resistant Tuberculosis.
Clin Infect Dis. 2018 Nov 28;67(suppl_3):S317-S326. doi: 10.1093/cid/ciy609.
PubMed PMID: 30496457; PubMed Central PMCID: PMC6260165.

40: Visser J, McLachlan MH, Maayan N, Garner P. Community-based supplementary
feeding for food insecure, vulnerable and malnourished populations - an overview
of systematic reviews. Cochrane Database Syst Rev. 2018 Nov 9;11:CD010578. doi:
10.1002/14651858.CD010578.pub2. PubMed PMID: 30480324.

41: Lee JK, Lee JY, Kim DK, Yoon HI, Jeong I, Heo EY, Park YS, Jo YS, Lee JH,
Park SS, Park JS, Kim J, Lee SM, Joh JS, Lee CH, Lee J, Choi SM, Park JH, Lee SH,
Cho YJ, Lee YJ, Kim SJ, Kwak N, Hwang YR, Kim H, Ki J, Lim JN, Choi HS, Lee M,
Song T, Kim HS, Han J, Ahn H, Hahn S, Yim JJ. Substitution of ethambutol with

linezolid during the intensive phase of treatment of pulmonary tuberculosis: a
prospective, multicentre, randomised, open-label, phase 2 trial. Lancet Infect
Dis. 2019 Jan;19(1):46-55. doi: 10.1016/S1473-3099(18)30480-8. Epub 2018 Nov 23.
PubMed PMID: 30477961.

42: Meintjes G, Stek C, Blumenthal L, Thienemann F, Schutz C, Buyze J, Ravinetto
R, van Loen H, Nair A, Jackson A, Colebunders R, Maartens G, Wilkinson RJ, Lynen
L; PredART Trial Team. Prednisone for the Prevention of Paradoxical
Tuberculosis-Associated IRIS. N Engl J Med. 2018 Nov 15;379(20):1915-1925. doi:
10.1056/NEJMoa1800762. PubMed PMID: 30428290.

43: Ferrian S, Ross M, Conradie F, Vally Omar S, Ismail N, Little F, Kaplan G,
Fallows D, Gray CM. Frequency of Circulating CD4(+)Ki67(+)HLA-DR(-) T Regulatory
Cells Prior to Treatment for Multidrug Resistant Tuberculosis Can Differentiate
the Severity of Disease and Predict Time to Culture Conversion. Front Immunol.
2018 Oct 25;9:2438. doi: 10.3389/fimmu.2018.02438. eCollection 2018. PubMed PMID:
30410488; PubMed Central PMCID: PMC6209685.

44: Parvaresh L, Bag SK, Cho JG, Heron N, Assareh H, Norton S, Corbett S, Marais
BJ. Monitoring tuberculosis contact tracing outcomes in Western Sydney,
Australia. BMJ Open Respir Res. 2018 Oct 25;5(1):e000341. doi:
10.1136/bmjresp-2018-000341. eCollection 2018. PubMed PMID: 30397487; PubMed
Central PMCID: PMC6203069.

45: Te Riele JB, Buser V, Calligaro G, Esmail A, Theron G, Lesosky M, Dheda K.
Relationship between chest radiographic characteristics, sputum bacterial load,
and treatment outcomes in patients with extensively drug-resistant tuberculosis.
Int J Infect Dis. 2019 Feb;79:65-71. doi: 10.1016/j.ijid.2018.10.026. Epub 2018
Nov 3. PubMed PMID: 30395979.

46: Yamada K, Seki Y, Nakagawa T, Hayashi Y, Yagi M, Ogawa K. Outcomes and risk

factors after adjuvant surgical treatments for Mycobacterium avium complex lung
disease. Gen Thorac Cardiovasc Surg. 2018 Nov 2. doi: 10.1007/s11748-018-1029-4.
[Epub ahead of print] PubMed PMID: 30390231.

47: Choi JS, Lee SH, Leem AY, Song JH, Kim SY, Chung KS, Jung JY, Kang YA, Kim
YS, Chang J, Park MS. Pneumocystis jirovecii pneumonia (PCP) PCR-negative
conversion predicts prognosis of HIV-negative patients with PCP and acute
respiratory failure. PLoS One. 2018 Oct 25;13(10):e0206231. doi:
10.1371/journal.pone.0206231. eCollection 2018. PubMed PMID: 30359436; PubMed
Central PMCID: PMC6201940.

48: Ahmed MIM, Ntinginya NE, Kibiki G, Mtafya BA, Semvua H, Mpagama S, Mtabho C,
Saathoff E, Held K, Loose R, Kroidl I, Chachage M, von Both U, Haule A, Mekota
AM, Boeree MJ, Gillespie SH, Hoelscher M, Heinrich N, Geldmacher C. Phenotypic
Changes on Mycobacterium Tuberculosis-Specific CD4 T Cells as Surrogate Markers
for Tuberculosis Treatment Efficacy. Front Immunol. 2018 Sep 28;9:2247. doi:
10.3389/fimmu.2018.02247. eCollection 2018. PubMed PMID: 30323818; PubMed Central
PMCID: PMC6172348.

49: Myers B, Bouton TC, Ragan EJ, White LF, McIlleron H, Theron D, Parry CDH,
Horsburgh CR, Warren RM, Jacobson KR. Impact of alcohol consumption on
tuberculosis treatment outcomes: a prospective longitudinal cohort study
protocol. BMC Infect Dis. 2018 Sep 29;18(1):488. doi: 10.1186/s12879-018-3396-y.
PubMed PMID: 30268101; PubMed Central PMCID: PMC6162918.

50: Griffith DE, Eagle G, Thomson R, Aksamit TR, Hasegawa N, Morimoto K,
Addrizzo-Harris DJ, O'Donnell AE, Marras TK, Flume PA, Loebinger MR, Morgan L,
Codecasa LR, Hill AT, Ruoss SJ, Yim JJ, Ringshausen FC, Field SK, Philley JV,
Wallace RJ Jr, van Ingen J, Coulter C, Nezamis J, Winthrop KL; CONVERT Study
Group. Amikacin Liposome Inhalation Suspension for Treatment-Refractory Lung

Disease Caused by Mycobacterium avium Complex (CONVERT): A Prospective,
Open-Label, Randomized Study. Am J Respir Crit Care Med. 2018 Sep 14. doi:
10.1164/rccm.201807-1318OC. [Epub ahead of print] PubMed PMID: 30216086.

51: Ni Y, Ding L, Yu Y, Dai R, Chen H, Shi G. Oscillatory positive expiratory
pressure treatment in lower respiratory tract infection. Exp Ther Med. 2018
Oct;16(4):3241-3248. doi: 10.3892/etm.2018.6552. Epub 2018 Aug 1. PubMed PMID:
30214547; PubMed Central PMCID: PMC6125886.

52: Yuengling KA, Padayatchi N, Wolf A, Mathema B, Brown T, Horsburgh CR,
OÊ¼Donnell MR. Effect of Antiretroviral Therapy on Treatment Outcomes in a
Prospective Study of Extensively Drug-Resistant Tuberculosis (XDR-TB) HIV
Coinfection Treatment in KwaZulu-Natal, South Africa. J Acquir Immune Defic
Syndr. 2018 Dec 1;79(4):474-480. doi: 10.1097/QAI.0000000000001833. PubMed PMID:
30212394; PubMed Central PMCID: PMC6203657.

53: Kedia K, Wendler JP, Baker ES, Burnum-Johnson KE, Jarsberg LG, Stratton KG,
Wright AT, Piehowski PD, Gritsenko MA, Lewinsohn DM, Sigal GB, Weiner MH, Smith
RD, Jacobs JM, Nahid P. Application of multiplexed ion mobility spectrometry
towards the identification of host protein signatures of treatment effect in
pulmonary tuberculosis. Tuberculosis (Edinb). 2018 Sep;112:52-61. doi:
10.1016/j.tube.2018.07.005. Epub 2018 Jul 18. PubMed PMID: 30205969; PubMed
Central PMCID: PMC6181582.

54: Goring SM, Wilson JB, Risebrough NR, Gallagher J, Carroll S, Heap KJ,
Obradovic M, Loebinger MR, Diel R. The cost of Mycobacterium avium complex lung
disease in Canada, France, Germany, and the United Kingdom: a nationally
representative observational study. BMC Health Serv Res. 2018 Sep 10;18(1):700.
doi: 10.1186/s12913-018-3489-8. PubMed PMID: 30200944; PubMed Central PMCID:
PMC6131733.

55: Kibuule D, Verbeeck RK, Nunurai R, Mavhunga F, Ene E, Godman B, Rennie TW.
Predictors of tuberculosis treatment success under the DOTS program in Namibia.
Expert Rev Respir Med. 2018 Nov;12(11):979-987. doi:
10.1080/17476348.2018.1520637. Epub 2018 Oct 4. PubMed PMID:
30198358.

56: Afzal A, Rathore R, Butt NF, Randhawa FA. Efficacy of Vitamin D supplementation in achieving an early Sputum Conversion in Smear positive
Pulmonary Tuberculosis. Pak J Med Sci. 2018 Jul-Aug;34(4):849-854. doi:
10.12669/pjms.344.14397. PubMed PMID: 30190740; PubMed Central PMCID: PMC6115594.

57: Filate M, Mehari Z, Alemu YM. Longitudinal body weight and sputum conversion
in patients with tuberculosis, Southwest Ethiopia: a retrospective follow-up
study. BMJ Open. 2018 Sep 5;8(9):e019076. doi: 10.1136/bmjopen-2017-019076.
PubMed PMID: 30185566; PubMed Central PMCID: PMC6129038.

58: Aznar ML, Zubrinic M, Siemienowicz M, Hashimoto K, Brode SK, Mehrabi M,
Patsios D, Keshavjee S, Marras TK. Adjuvant lung resection in the management of
nontuberculous mycobacterial lung infection: A retrospective matched cohort
study. Respir Med. 2018 Sep;142:1-6. doi:
10.1016/j.rmed.2018.07.003. Epub 2018
Jul 9. PubMed PMID: 30170795.

59: Alfarisi O, Mave V, Gaikwad S, Sahasrabudhe T, Ramachandran G, Kumar H, Gupte
N, Kulkarni V, Deshmukh S, Atre S, Raskar S, Lokhande R, Barthwal M, Kakrani A,
Chon S, Gupta A, Golub JE, Dooley KE. Effect of Diabetes Mellitus on the
Pharmacokinetics and Pharmacodynamics of Tuberculosis Treatment. Antimicrob
Agents Chemother. 2018 Oct 24;62(11). pii: e01383-18. doi:
10.1128/AAC.01383-18.
Print 2018 Nov. PubMed PMID: 30126955; PubMed Central PMCID:
PMC6201087.

60: Tan Q, Ai Q, Xu Q, Li F, Yu J. Polymorphonuclear Leukocytes or Hydrogen

Peroxide Enhance Biofilm Development of Mucoid Pseudomonas aeruginosa. Mediators
Inflamm. 2018 Jul 4;2018:8151362. doi: 10.1155/2018/8151362. eCollection 2018.
PubMed PMID: 30116152; PubMed Central PMCID: PMC6079396.

61: Shin SH, Jhun BW, Kim SY, Choe J, Jeon K, Huh HJ, Ki CS, Lee NY, Shin SJ,
Daley CL, Koh WJ. Nontuberculous Mycobacterial Lung Diseases Caused by Mixed
Infection with Mycobacterium avium Complex and Mycobacterium abscessus Complex.
Antimicrob Agents Chemother. 2018 Sep 24;62(10). pii: e01105-18. doi:
10.1128/AAC.01105-18. Print 2018 Oct. PubMed PMID: 30104265; PubMed Central
PMCID: PMC6153851.

62: Al-Shaer MH, Elewa H, Alkabab Y, Nazer LH, Heysell SK. Fixed-dose combination
associated with faster time to smear conversion compared to separate tablets of
anti-tuberculosis drugs in patients with poorly controlled diabetes and pulmonary
tuberculosis in Qatar. BMC Infect Dis. 2018 Aug 8;18(1):384. doi:
10.1186/s12879-018-3309-0. PubMed PMID: 30089476; PubMed Central PMCID:
PMC6083564.

63: Ghosh R, Roy S, Rashid MK. Assessment of microbiological status after
successful completion of intermittent revised national tuberculosis control
programme directly observed treatment, short course regimen for microbiologically
confirmed pulmonary tuberculosis cases: While new daily regimen going to be
implemented in India. Indian J Med Microbiol. 2018 Apr-Jun;36(2):251-256. doi:
10.4103/ijmm.IJMM_18_65. PubMed PMID: 30084420.

64: Feng JY, Pan SW, Huang SF, Chen YY, Lin YY, Su WJ. Depressed Gamma Interferon
Responses and Treatment Outcomes in Tuberculosis Patients: a Prospective Cohort
Study. J Clin Microbiol. 2018 Sep 25;56(10). pii: e00664-18. doi:
10.1128/JCM.00664-18. Print 2018 Oct. PubMed PMID: 30068533; PubMed Central
PMCID: PMC6156303.

65: Liu Y, Matsumoto M, Ishida H, Ohguro K, Yoshitake M, Gupta R, Geiter L,
Hafkin J. Delamanid: From discovery to its use for pulmonary multidrug-resistant
tuberculosis (MDR-TB). Tuberculosis (Edinb). 2018 Jul;111:20-30. doi:
10.1016/j.tube.2018.04.008. Epub 2018 May 3. Review. PubMed PMID: 30029909.

66: Zhang R, Xi X, Wang C, Pan Y, Ge C, Zhang L, Zhang S, Liu H. Therapeutic
effects of recombinant human interleukin 2 as adjunctive immunotherapy against
tuberculosis: A systematic review and meta-analysis. PLoS One. 2018 Jul
19;13(7):e0201025. doi: 10.1371/journal.pone.0201025. eCollection 2018. PubMed
PMID: 30024982; PubMed Central PMCID: PMC6053227.

67: Meyvisch P, Kambili C, Andries K, Lounis N, Theeuwes M, Dannemann B,
Vandebosch A, Van der Elst W, Molenberghs G, Alonso A. Evaluation of six months
sputum culture conversion as a surrogate endpoint in a multidrug
resistant-tuberculosis trial. PLoS One. 2018 Jul 19;13(7):e0200539. doi:
10.1371/journal.pone.0200539. eCollection 2018. PubMed PMID: 30024924; PubMed
Central PMCID: PMC6053142.

68: Rekha RS, Mily A, Sultana T, Haq A, Ahmed S, Mostafa Kamal SM, van Schadewijk
A, Hiemstra PS, Gudmundsson GH, Agerberth B, Raqib R. Immune responses in the
treatment of drug-sensitive pulmonary tuberculosis with phenylbutyrate and
vitamin D(3) as host directed therapy. BMC Infect Dis. 2018 Jul 4;18(1):303. doi:
10.1186/s12879-018-3203-9. PubMed PMID: 29973153; PubMed Central PMCID:
PMC6033279.

69: Alipanah N, Jarlsberg L, Miller C, Linh NN, Falzon D, Jaramillo E, Nahid P.
Adherence interventions and outcomes of tuberculosis treatment: A systematic
review and meta-analysis of trials and observational studies. PLoS Med. 2018 Jul
3;15(7):e1002595. doi: 10.1371/journal.pmed.1002595. eCollection 2018 Jul. PubMed
PMID: 29969463; PubMed Central PMCID: PMC6029765.

70: do Socorro Nantua Evangelista M, Maia R, Toledo JP, de Abreu RG, Braga JU,
Barreira D, Trajman A. Second month sputum smear as a predictor of tuberculosis
treatment outcomes in Brazil. BMC Res Notes. 2018 Jun 28;11(1):414. doi:
10.1186/s13104-018-3522-3. PubMed PMID: 29954436; PubMed Central PMCID:
PMC6022340.

71: Wu HX, Xiong XF, Zhu M, Wei J, Zhuo KQ, Cheng DY. Effects of vitamin D
supplementation on the outcomes of patients with pulmonary tuberculosis: a
systematic review and meta-analysis. BMC Pulm Med. 2018 Jun 28;18(1):108. doi:
10.1186/s12890-018-0677-6. PubMed PMID: 29954353; PubMed Central PMCID:
PMC6025740.

72: Shibabaw A, Gelaw B, Wang SH, Tessema B. Time to sputum smear and culture
conversions in multidrug resistant tuberculosis at University of Gondar Hospital,
Northwest Ethiopia. PLoS One. 2018 Jun 26;13(6):e0198080. doi:
10.1371/journal.pone.0198080. eCollection 2018. PubMed PMID: 29944658; PubMed
Central PMCID: PMC6019386.

73: Kumar R, Rai J, Kajal NC, Devi P. Comparative study of effect of Withania
somnifera as an adjuvant to DOTS in patients of newly diagnosed sputum smear
positive pulmonary tuberculosis. Indian J Tuberc. 2018 Jul;65(3):246-251. doi:
10.1016/j.ijtb.2017.05.005. Epub 2017 May 21. PubMed PMID: 29933868.

74: Yihunie Akalu T, Muchie KF, Alemu Gelaye K. Time to sputum culture conversion
and its determinants among Multi-drug resistant Tuberculosis patients at public
hospitals of the Amhara Regional State: A multicenter retrospective follow up
study. PLoS One. 2018 Jun 21;13(6):e0199320. doi: 10.1371/journal.pone.0199320.
eCollection 2018. PubMed PMID: 29927980; PubMed Central PMCID: PMC6013102.

75: Diktanas S, Vasiliauskiene E, Polubenko K, Danila E, Celedinaite I,

Boreikaite E, Misiunas K. Factors Associated with Persistent Sputum Positivity at
the End of the Second Month of Tuberculosis Treatment in Lithuania. Tuberc Respir
Dis (Seoul). 2018 Jul;81(3):233-240. doi: 10.4046/trd.2017.0096. Epub 2018 Jun
19. PubMed PMID: 29926543; PubMed Central PMCID: PMC6030656.

76: Svensson EM, Svensson RJ, Te Brake LHM, Boeree MJ, Heinrich N, Konsten S,
Churchyard G, Dawson R, Diacon AH, Kibiki GS, Minja LT, Ntingiya NE, Sanne I,
Gillespie SH, Hoelscher M, Phillips PPJ, Simonsson USH, Aarnoutse R. The
Potential for Treatment Shortening With Higher Rifampicin Doses: Relating Drug
Exposure to Treatment Response in Patients With Pulmonary Tuberculosis. Clin
Infect Dis. 2018 Jun 18;67(1):34-41. doi: 10.1093/cid/ciy026. PubMed PMID:
29917079; PubMed Central PMCID: PMC6005123.

77: Wang J, Feng M, Ying S, Zhou J, Li X. Efficacy and Safety of Vitamin D
Supplementation for Pulmonary Tuberculosis: A Systematic Review and
Meta-analysis. Iran J Public Health. 2018 Apr;47(4):466-472. Review. PubMed PMID:
29900130; PubMed Central PMCID: PMC5996342.

78: Patel SV, Nimavat KB, Patel AB, Mehta KG, Shringarpure K, Shukla LK. Sputum
Smear and Culture Conversion in Multidrug Resistance Tuberculosis Patients in
Seven Districts of Central Gujarat, India: A Longitudinal Study. Indian J
Community Med. 2018 Apr-Jun;43(2):117-119. doi: 10.4103/ijcm.IJCM_152_17. PubMed
PMID: 29899612; PubMed Central PMCID: PMC5974826.

79: Cailleaux-Cezar M, Loredo C, Silva JRLE, Conde MB. Impact of smoking on
sputum culture conversion and pulmonary tuberculosis treatment outcomes in
Brazil: a retrospective cohort study. J Bras Pneumol. 2018 Apr;44(2):99-105. doi:
10.1590/s1806-37562017000000161. Portuguese, English. PubMed PMID: 29791542;
PubMed Central PMCID: PMC6044654.

80: Alene KA, Viney K, Yi H, McBryde ES, Yang K, Bai L, Gray DJ, Xu Z, Clements

ACA. Comparison of the validity of smear and culture conversion as a prognostic
marker of treatment outcome in patients with multidrug-resistant tuberculosis.
PLoS One. 2018 May 23;13(5):e0197880. doi: 10.1371/journal.pone.0197880.
eCollection 2018. PubMed PMID: 29791488; PubMed Central PMCID: PMC5965863.

81: Agodokpessi G, Wachinou AP, Awanou B, Gninafon M. [Factors associated with
non-conversion of the direct smear after the initial phase of anti-tuberculous
treatment. A study undertaken in three tuberculosis management centres in South
Benin]. Rev Mal Respir. 2018 May;35(5):546-551. doi: 10.1016/j.rmr.2017.06.004.
Epub 2018 May 16. French. PubMed PMID: 29778620.

82: Musarurwa C, Zijenah LS, Mhandire DZ, Bandason T, Mhandire K, Chipiti MM,
Munjoma MW, Mujaji WB. Higher serum 25-hydroxyvitamin D concentrations are
associated with active pulmonary tuberculosis in hospitalised HIV infected
patients in a low income tropical setting: a cross sectional study. BMC Pulm Med.
2018 May 8;18(1):67. doi: 10.1186/s12890-018-0640-6. PubMed PMID: 29739378;
PubMed Central PMCID: PMC5941493.

83: Mohr E, Hughes J, Reuter A, Trivino Duran L, Ferlazzo G, Daniels J, De
Azevedo V, Kock Y, Steele SJ, Shroufi A, Ade S, Alikhanova N, Benedetti G,
Edwards J, Cox H, Furin J, Isaakidis P. Delamanid for rifampicin-resistant
tuberculosis: a retrospective study from South Africa. Eur Respir J. 2018 Jun
14;51(6). pii: 1800017. doi: 10.1183/13993003.00017-2018. Print 2018 Jun. PubMed
PMID: 29724920.

84: Asres A, Jerene D, Deressa W. Delays to treatment initiation is associated
with tuberculosis treatment outcomes among patients on directly observed
treatment short course in Southwest Ethiopia: a follow-up study. BMC Pulm Med.
2018 May 2;18(1):64. doi: 10.1186/s12890-018-0628-2. PubMed PMID: 29716569;
PubMed Central PMCID: PMC5930812.

85: Bekele A, Gebreselassie N, Ashenafi S, Kassa E, Aseffa G, Amogne W, Getachew
M, Aseffa A, Worku A, Raqib R, Agerberth B, Hammar U, Bergman P, Aderaye G,
Andersson J, Brighenti S. Daily adjunctive therapy with vitamin D(3) and
phenylbutyrate supports clinical recovery from pulmonary tuberculosis: a
randomized controlled trial in Ethiopia. J Intern Med. 2018 Sep;284(3):292-306.
doi: 10.1111/joim.12767. Epub 2018 May 23. PubMed PMID: 29696707; PubMed Central
PMCID: PMC6202271.

86: Rumende CM. Risk Factors for Multidrug-resistant Tuberculosis. Acta Med
Indones. 2018 Jan;50(1):1-2. PubMed PMID: 29686169.

87: Jhun BW, Yang B, Moon SM, Lee H, Park HY, Jeon K, Kwon OJ, Ahn J, Moon IJ,
Shin SJ, Daley CL, Koh WJ. Amikacin Inhalation as Salvage Therapy for Refractory
Nontuberculous Mycobacterial Lung Disease. Antimicrob Agents Chemother. 2018 Jun
26;62(7). pii: e00011-18. doi: 10.1128/AAC.00011-18. Print 2018 Jul. PubMed PMID:
29661870; PubMed Central PMCID: PMC6021683.

88: Parmar MM, Sachdeva KS, Dewan PK, Rade K, Nair SA, Pant R, Khaparde SD.
Unacceptable treatment outcomes and associated factors among India's initial
cohorts of multidrug-resistant tuberculosis (MDR-TB) patients under the revised
national TB control programme (2007-2011): Evidence leading to policy
enhancement. PLoS One. 2018 Apr 11;13(4):e0193903. doi:
10.1371/journal.pone.0193903. eCollection 2018. PubMed PMID: 29641576; PubMed
Central PMCID: PMC5894982.

89: Rifat D, Prideaux B, Savic RM, Urbanowski ME, Parsons TL, Luna B, Marzinke
MA, Ordonez AA, DeMarco VP, Jain SK, Dartois V, Bishai WR, Dooley KE.
Pharmacokinetics of rifapentine and rifampin in a rabbit model of tuberculosis
and correlation with clinical trial data. Sci Transl Med. 2018 Apr 4;10(435).

pii: eaai7786. doi: 10.1126/scitranslmed.aai7786. PubMed PMID: 29618565; PubMed
Central PMCID: PMC5969904.

90: Javaid A, Ahmad N, Afridi AK, Basit A, Khan AH, Ahmad I, Atif M. Validity of
Time to Sputum Culture Conversion to Predict Cure in Patients with
Multidrug-Resistant Tuberculosis: A Retrospective Single-Center Study. Am J Trop
Med Hyg. 2018 Jun;98(6):1629-1636. doi: 10.4269/ajtmh.17-0936. Epub 2018 Mar 29.
PubMed PMID: 29611497; PubMed Central PMCID: PMC6086179.

91: Forson A, Kwara A, Kudzawu S, Omari M, Otu J, Gehre F, de Jong B, Antonio M.
A cross-sectional study of tuberculosis drug resistance among previously treated
patients in a tertiary hospital in Accra, Ghana: public health implications of
standardized regimens. BMC Infect Dis. 2018 Apr 2;18(1):149. doi:
10.1186/s12879-018-3053-5. PubMed PMID: 29606091; PubMed Central PMCID:
PMC5879759.

92: Heyckendorf J, van Leth F, Avsar K, Glattki G, GÃ¼nther G, Kalsdorf B, MÃ¼ller
M, Olaru ID, Rolling T, Salzer HJF, Schuhmann M, Terhalle E, Lange C. Treatment
responses in multidrug-resistant tuberculosis in Germany. Int J Tuberc Lung Dis.
2018 Apr 1;22(4):399-406. doi: 10.5588/ijtld.17.0741. PubMed PMID: 29562987.

93: ProaÃ±o A, Bui DP, LÃ³pez JW, Vu NM, Bravard MA, Lee GO, Tracey BH, Xu Z,
Comina G, Ticona E, Mollura DJ, Friedland JS, Moore DAJ, Evans CA, Caligiuri P,
Gilman RH; Tuberculosis Working Group in Peru*. Cough Frequency During Treatment
Associated With Baseline Cavitary Volume and Proximity to the Airway in Pulmonary
TB. Chest. 2018 Jun;153(6):1358-1367. doi: 10.1016/j.chest.2018.03.006. Epub 2018
Mar 17. PubMed PMID: 29559307; PubMed Central PMCID: PMC6026292.

94: Kim CT, Kim TO, Shin HJ, Ko YC, Hun Choe Y, Kim HR, Kwon YS. Bedaquiline and
delamanid for the treatment of multidrug-resistant tuberculosis: a multicentre
cohort study in Korea. Eur Respir J. 2018 Mar 22;51(3). pii: 1702467. doi:

10.1183/13993003.02467-2017. Print 2018 Mar. PubMed PMID: 29545276.

95: Lee YJ, Han SK, Park JH, Lee JK, Kim DK, Chung HS, Heo EY. The effect of
metformin on culture conversion in tuberculosis patients with diabetes mellitus.
Korean J Intern Med. 2018 Sep;33(5):933-940. doi: 10.3904/kjim.2017.249. Epub
2018 Mar 16. PubMed PMID: 29540054; PubMed Central PMCID: PMC6129638.

96: Kobayashi T, Tsuyuguchi K, Yoshida S, Kurahara Y, Ikegami N, Naito M, Sonobe
S, Maekura T, Tsuji T, Minomo S, Inoue Y, Suzuki K. Mycobacterium abscessus
subsp. abscessus Lung Disease: Drug Susceptibility Testing in Sputum Culture
Negative Conversion. Int J Mycobacteriol. 2018 Jan-Mar;7(1):69-75. doi:
10.4103/ijmy.ijmy_179_17. PubMed PMID: 29516889.

97: Sekaggya-Wiltshire C, von Braun A, Lamorde M, Ledergerber B, Buzibye A,
Henning L, Musaazi J, Gutteck U, Denti P, de Kock M, Jetter A, Byakika-Kibwika P,
Eberhard N, Matovu J, Joloba M, Muller D, Manabe YC, Kamya MR, Corti N, Kambugu
A, Castelnuovo B, Fehr JS. Delayed Sputum Culture Conversion in
Tuberculosis-Human Immunodeficiency Virus-Coinfected Patients With Low Isoniazid
and Rifampicin Concentrations. Clin Infect Dis. 2018 Aug 16;67(5):708-716. doi:
10.1093/cid/ciy179. PubMed PMID: 29514175; PubMed Central PMCID: PMC6094003.

98: Liu Q, Lu P, Martinez L, Yang H, Lu W, Ding X, Zhu L. Factors affecting time
to sputum culture conversion and treatment outcome of patients with
multidrug-resistant tuberculosis in China. BMC Infect Dis. 2018 Mar 6;18(1):114.
doi: 10.1186/s12879-018-3021-0. PubMed PMID: 29510666; PubMed Central PMCID:
PMC5840772.

99: MusteikienÄ— G, Miliauskas S, ZaveckienÄ— J, Å½emaitis M, VitkauskienÄ— A.
Factors associated with sputum culture conversion in patients with pulmonary
tuberculosis. Medicina (Kaunas). 2017;53(6):386-393. doi:
10.1016/j.medici.2018.01.005. Epub 2018 Feb 9. PubMed PMID: 29496377.

100: Kamariza M, Shieh P, Ealand CS, Peters JS, Chu B, Rodriguez-Rivera FP, Babu
Sait MR, Treuren WV, Martinson N, Kalscheuer R, Kana BD, Bertozzi CR. Rapid
detection of Mycobacterium tuberculosis in sputum with a solvatochromic trehalose
probe. Sci Transl Med. 2018 Feb 28;10(430). pii: eaam6310. doi:
10.1126/scitranslmed.aam6310. PubMed PMID: 29491187; PubMed Central PMCID:
PMC5985656.

101: BariÄeviÄ‡ D, PopoviÄ‡ Grle S, MoroviÄ‡ Vergles J, ÄŒukoviÄ‡ ÄŒavka S, JakopoviÄ‡
M, RedÅ¾epi G, Boras Z, BariÄeviÄ‡ M, SamarÅ¾ija M. QuantiFERON-TB Gold In-Tube Test
in the Diagnosis of Latent Tuberculosis Infection in Arthritis Patients Treated
with Tumor Necrosis Factor Antagonists. Acta Clin Croat. 2017 Jun;56(2):203-209.
doi: 10.20471/acc.2017.56.02.02. PubMed PMID: 29485786.

102: Guo Q, Chu H, Ye M, Zhang Z, Li B, Yang S, Ma W, Yu F. The Clarithromycin
Susceptibility Genotype Affects the Treatment Outcome of Patients with
Mycobacterium abscessus Lung Disease. Antimicrob Agents Chemother. 2018 Apr
26;62(5). pii: e02360-17. doi: 10.1128/AAC.02360-17. Print 2018 May. PubMed PMID:
29483126; PubMed Central PMCID: PMC5923093.

103: Agrawal S, Parkash O, Palaniappan AN, Bhatia AK, Kumar S, Chauhan DS, Madhan
Kumar M. Efficacy of T Regulatory Cells, Th17 Cells and the Associated Markers in
Monitoring Tuberculosis Treatment Response. Front Immunol. 2018 Feb 5;9:157. doi:
10.3389/fimmu.2018.00157. eCollection 2018. PubMed PMID: 29472922; PubMed Central
PMCID: PMC5810270.

104: Adane K, Spigt M, Dinant GJ. Tuberculosis treatment outcome and predictors
in northern Ethiopian prisons: a five-year retrospective analysis. BMC Pulm Med.
2018 Feb 20;18(1):37. doi: 10.1186/s12890-018-0600-1. PubMed PMID: 29463234;
PubMed Central PMCID: PMC5819685.

105: Hong JY, Kim M, Sol IS, Kim KW, Lee CM, Elias JA, Sohn MH, Lee CG.
Chitotriosidase inhibits allergic asthmatic airways via regulation of TGF-Î²
expression and Foxp3(+) Treg cells. Allergy. 2018 Aug;73(8):1686-1699. doi:
10.1111/all.13426. Epub 2018 Mar 5. PubMed PMID: 29420850; PubMed Central PMCID:
PMC6047905.

106: Lv L, Li T, Xu K, Shi P, He B, Kong W, Wang J, Sun J. Sputum bacteriology
conversion and treatment outcome of patients with multidrug-resistant
tuberculosis. Infect Drug Resist. 2018 Jan 23;11:147-154. doi:
10.2147/IDR.S153499. eCollection 2018. PubMed PMID: 29416359; PubMed Central
PMCID: PMC5790105.

107: Wang Q, Pang Y, Jing W, Liu Y, Wang N, Yin H, Zhang Q, Ye Z, Zhu M, Li F,
Liu P, Wu T, Chen W, Wu W, Qin Z, Qiu C, Deng Q, Xu T, Wang J, Guo R, Du Y, Wang
J, Huang H, Chen X, Chu N. Clofazimine for Treatment of Extensively
Drug-Resistant Pulmonary Tuberculosis in China. Antimicrob Agents Chemother. 2018
Mar 27;62(4). pii: e02149-17. doi: 10.1128/AAC.02149-17. Print 2018 Apr. PubMed
PMID: 29378718; PubMed Central PMCID: PMC5913945.

108: Choi H, Jhun BW, Kim SY, Kim DH, Lee H, Jeon K, Kwon OJ, Huh HJ, Ki CS, Lee
NY, Shin SJ, Daley CL, Koh WJ. Treatment outcomes of macrolide-susceptible
Mycobacterium abscessus lung disease. Diagn Microbiol Infect Dis. 2018
Apr;90(4):293-295. doi: 10.1016/j.diagmicrobio.2017.12.008. Epub 2017 Dec 16.
PubMed PMID: 29329759.

109: Degner NR, Wang JY, Golub JE, Karakousis PC. Metformin Use Reverses the
Increased Mortality Associated With Diabetes Mellitus During Tuberculosis
Treatment. Clin Infect Dis. 2018 Jan 6;66(2):198-205. doi: 10.1093/cid/cix819.
PubMed PMID: 29325084; PubMed Central PMCID: PMC5848303.

110: Patel N, Jagannath K, Vora A, Patel M, Patel A. A Randomized, Controlled,

Phase III Clinical Trial to Evaluate the Efficacy and Tolerability of Risorine
with Conventional Rifampicin in the Treatment of Newly Diagnosed Pulmonary
Tuberculosis Patients. J Assoc Physicians India. 2017 Sep;65(9):48-54. PubMed
PMID: 29313577.

111: Tan Q, Min R, Dai GQ, Wang YL, Nan L, Yang Z, Xia J, Pan SY, Mao H, Xie WP,
Wang H. Clinical and Immunological Effects of rhIL-2 Therapy in Eastern Chinese
Patients with Multidrug-resistant Tuberculosis. Sci Rep. 2017 Dec 19;7(1):17854.
doi: 10.1038/s41598-017-18200-5. PubMed PMID: 29259310; PubMed Central PMCID:
PMC5736576.

112: Jhun BW, Moon SM, Kim SY, Park HY, Jeon K, Kwon OJ, Huh HJ, Ki CS, Lee NY,
Chung MJ, Lee KS, Shin SJ, Daley CL, Koh WJ. Intermittent Antibiotic Therapy for
Recurrent Nodular Bronchiectatic Mycobacterium avium Complex Lung Disease.
Antimicrob Agents Chemother. 2018 Jan 25;62(2). pii: e01812-17. doi:
10.1128/AAC.01812-17. Print 2018 Feb. PubMed PMID: 29203483; PubMed Central
PMCID: PMC5786774.

113: Lima GL, Paupitz JA, Aikawa NE, Alvarenga JC, Pereira RMR. A randomized
double-blind placebo-controlled trial of vitamin D supplementation in
juvenile-onset systemic lupus erythematosus: positive effect on trabecular
microarchitecture using HR-pQCT. Osteoporos Int. 2018 Mar;29(3):587-594. doi:
10.1007/s00198-017-4316-5. Epub 2017 Nov 19. PubMed PMID: 29152675.

114: Salindri AD, Sales RF, DiMiceli L, Schechter MC, Kempker RR, Magee MJ.
Isoniazid Monoresistance and Rate of Culture Conversion among Patients in the
State of Georgia with Confirmed Tuberculosis, 2009-2014. Ann Am Thorac Soc. 2018
Mar;15(3):331-340. doi: 10.1513/AnnalsATS.201702-147OC. PubMed PMID: 29131662;
PubMed Central PMCID: PMC5880520.

115: Hashemian SM, Mortaz E, Jamaati H, Bagheri L, Mohajerani SA, Garssen J,

Movassaghi M, Barnes PJ, Hill NS, Adcock IM. Budesonide facilitates weaning from
mechanical ventilation in difficult-to-wean very severe COPD patients:
Association with inflammatory mediators and cells. J Crit Care. 2018
Apr;44:161-167. doi: 10.1016/j.jcrc.2017.10.045. Epub 2017 Oct 31. PubMed PMID:
29127842.

116: Mok J, Kang H, Hwang SH, Park JS, Kang B, Lee T, Koh WJ, Yim JJ, Jeon D.
Interim outcomes of delamanid for the treatment of MDR- and XDR-TB in South
Korea. J Antimicrob Chemother. 2018 Feb 1;73(2):503-508. doi: 10.1093/jac/dkx373.
PubMed PMID: 29069496.

117: Marfina GY, Vladimirov KB, Avetisian AO, Starshinova AA, Kudriashov GG,
Sokolovich EG, Yablonskii PK. Bilateral cavitary multidrug- or extensively
drug-resistant tuberculosis: role of surgery. Eur J Cardiothorac Surg. 2018 Mar
1;53(3):618-624. doi: 10.1093/ejcts/ezx350. PubMed PMID: 29040413.

118: Schechter MC, Bizune D, Kagei M, Machaidze M, Holland DP, Oladele A, Wang
YF, Rebolledo PA, Ray SM, Kempker RR. Time to Sputum Culture Conversion and
Treatment Outcomes Among Patients with Isoniazid-Resistant Tuberculosis in
Atlanta, Georgia. Clin Infect Dis. 2017 Nov 13;65(11):1862-1871. doi:
10.1093/cid/cix686. PubMed PMID: 29020173; PubMed Central PMCID: PMC5850645.

119: Svensson EM, Karlsson MO. Modelling of mycobacterial load reveals
bedaquiline's exposure-response relationship in patients with drug-resistant TB.
J Antimicrob Chemother. 2017 Dec 1;72(12):3398-3405. doi: 10.1093/jac/dkx317.
PubMed PMID: 28961790; PubMed Central PMCID: PMC5890768.

120: Prins HJ, Duijkers R, Lutter R, Daniels JM, van der Valk P, Schoorl M,
Kerstjens HA, van der Werf TS, Boersma WG. Blood eosinophilia as a marker of
early and late treatment failure in severe acute exacerbations of COPD. Respir

Med. 2017 Oct;131:118-124. doi: 10.1016/j.rmed.2017.07.064. Epub 2017 Aug 1.
PubMed PMID: 28947018.

121: ChoreÃ±o Parra JA, MartÃnez ZÃºÃ±iga N, Salinas Lara C. Tuberculosis "the great
imitator": False healing and subclinical activity. Indian J Tuberc. 2017
Oct;64(4):345-348. doi: 10.1016/j.ijtb.2017.05.006. Epub 2017 May 31. PubMed
PMID: 28941864.

122: Pasipanodya JG, Ogbonna D, Deshpande D, Srivastava S, Gumbo T. Meta-analyses
and the evidence base for microbial outcomes in the treatment of pulmonary
Mycobacterium avium-intracellulare complex disease. J Antimicrob Chemother. 2017
Sep 1;72(suppl_2):i3-i19. doi: 10.1093/jac/dkx311. Review. PubMed
PMID: 28922813.

123: Pavord ID, Chanez P, Criner GJ, Kerstjens HAM, Korn S, Lugogo N, Martinot
JB, Sagara H, Albers FC, Bradford ES, Harris SS, Mayer B, Rubin DB, Yancey SW,
Sciurba FC. Mepolizumab for Eosinophilic Chronic Obstructive Pulmonary Disease. N
Engl J Med. 2017 Oct 26;377(17):1613-1629. doi: 10.1056/NEJMoa1708208. Epub 2017
Sep 11. PubMed PMID: 28893134.

124: Sudfeld CR, Manji KP, Duggan CP, Aboud S, Muhihi A, Sando DM, Al-Beity FMA,
Wang M, Fawzi WW. Effect of maternal vitamin D(3) supplementation on maternal
health, birth outcomes, and infant growth among HIV-infected Tanzanian pregnant
women: study protocol for a randomized controlled trial. Trials. 2017 Sep
4;18(1):411. doi: 10.1186/s13063-017-2157-3. PubMed PMID: 28870263; PubMed
Central PMCID: PMC5584035.

125: Banurekha V, Bhatnagar T, Savithri S, Kumar ND, Kangusamy B, Mehendale S.
Sputum Conversion and Treatment Success among Tuberculosis Patients with Diabetes
Treated under the Tuberculosis Control Programme in an Urban Setting in South
India. Indian J Community Med. 2017 Jul-Sep;42(3):180-182. doi:

10.4103/ijcm.IJCM_179_16. PubMed PMID: 28852286; PubMed Central PMCID:
PMC5561700.

126: Sakashita K, Fujita A, Takamori M, Nagai T, Matsumoto T, Saito T, Nakagawa
T, Ogawa K, Shigeto E, Nakatsumi Y, Goto H, Mitarai S. Efficiency of the Lung
Flute for sputum induction in patients with presumed pulmonary tuberculosis. Clin
Respir J. 2018 Apr;12(4):1503-1509. doi: 10.1111/crj.12697. Epub 2017 Sep 13.
PubMed PMID: 28846200.

127: Saffari M, Jolandimi HA, Sehat M, Nejad NV, Hedayati M, Zamani M, Ghasemi A.
Smear grading and the Mantoux skin test can be used to predict sputum smear
conversion in patients suffering from tuberculosis. GMS Hyg Infect Control. 2017
Aug 15;12:Doc12. doi: 10.3205/dgkh000297. eCollection 2017. PubMed PMID:
28840092; PubMed Central PMCID: PMC5564005.

128: Altet N, Latorre I, JimÃ©nez-Fuentes MÃ, Maldonado J, Molina I, GonzÃ¡lez-DÃaz
Y, MilÃ C, GarcÃa-GarcÃa E, Muriel B, Villar-HernÃ¡ndez R, Laabei M, GÃ³mez AC,
Godoy P, de Souza-GalvÃ£o ML, Solano S, JimÃ©nez-Ruiz CA, DomÃnguez J; PII Smoking
SEPAR Working Group. Assessment of the influence of direct tobacco smoke on
infection and active TB management. PLoS One. 2017 Aug 24;12(8):e0182998. doi:
10.1371/journal.pone.0182998. eCollection 2017. PubMed PMID: 28837570; PubMed
Central PMCID: PMC5570217.

129: Mahakalkar SM, Nagrale D, Gaur S, Urade C, Murhar B, Turankar A.
N-acetylcysteine as an add-on to Directly Observed Therapy Short-I therapy in
fresh pulmonary tuberculosis patients: A randomized, placebo-controlled,
double-blinded study. Perspect Clin Res. 2017 Jul-Sep;8(3):132-136. doi:
10.4103/2229-3485.210450. PubMed PMID: 28828308; PubMed Central PMCID:
PMC5543764.

130: Pasipanodya JG, Ogbonna D, Ferro BE, Magombedze G, Srivastava S, Deshpande
D, Gumbo T. Systematic Review and Meta-analyses of the Effect of Chemotherapy on
Pulmonary Mycobacterium abscessus Outcomes and Disease Recurrence. Antimicrob
Agents Chemother. 2017 Oct 24;61(11). pii: e01206-17. doi: 10.1128/AAC.01206-17.
Print 2017 Nov. PubMed PMID: 28807911; PubMed Central PMCID: PMC5655093.

131: Jacobson DL, Stephensen CB, Miller TL, Patel K, Chen JS, Van Dyke RB, Mirza
A, Schuster GU, Hazra R, Ellis A, Brummel SS, Geffner ME, Silio M, Spector SA,
DiMeglio LA; Pediatric HIV/AIDS Cohort Study. Associations of Low Vitamin D and
Elevated Parathyroid Hormone Concentrations With Bone Mineral Density in
Perinatally HIV-Infected Children. J Acquir Immune Defic Syndr. 2017 Sep
1;76(1):33-42. doi: 10.1097/QAI.0000000000001467. PubMed PMID: 28797019; PubMed
Central PMCID: PMC5624211.

132: Yagi K, Ishii M, Namkoong H, Asami T, Iketani O, Asakura T, Suzuki S,
Sugiura H, Yamada Y, Nishimura T, Fujiwara H, Funatsu Y, Uwamino Y, Kamo T,
Tasaka S, Betsuyaku T, Hasegawa N. The efficacy, safety, and feasibility of
inhaled amikacin for the treatment of difficult-to-treat non-tuberculous
mycobacterial lung diseases. BMC Infect Dis. 2017 Aug 9;17(1):558. doi:
10.1186/s12879-017-2665-5. PubMed PMID: 28793869; PubMed Central PMCID:
PMC5550988.

133: Osei FA, Enimil A, Ansong D, Laryea DO, Mensah NK, Amuzu EX, Agyemang EO,
Sarpong PO, Nyanor I, Dekugmen Yar D. Review of Organism Density and
Bacteriologic Conversion of Sputum among Tuberculosis Patients. Int Sch Res
Notices. 2017 Jul 11;2017:7052583. doi: 10.1155/2017/7052583. eCollection 2017.
Review. PubMed PMID: 28781995; PubMed Central PMCID: PMC5525070.

134: Prajapati K, Mishra V, Desai M, Solanki R, Naik P. Treatment outcome of
patients having extensively drug-resistant tuberculosis in Gujarat, India. Int J

Mycobacteriol. 2017 Jul-Sep;6(3):289-295. doi:
10.4103/ijmy.ijmy_59_17. PubMed
PMID: 28776529.

135: Agrawal Y, Goyal V, Singh A, Lal S. Role of Anaemia and Magnesium Levels at
the Initiation of Tuberculosis Therapy with Sputum Conversion among Pulmonary
Tuberculosis Patients. J Clin Diagn Res. 2017 Jun;11(6):BC01-BC04. doi:
10.7860/JCDR/2017/23734.9975. Epub 2017 Jun 1. PubMed PMID: 28764146; PubMed
Central PMCID: PMC5535339.

136: Choi H, Kim SY, Kim DH, Huh HJ, Ki CS, Lee NY, Lee SH, Shin S, Shin SJ,
Daley CL, Koh WJ. Clinical Characteristics and Treatment Outcomes of Patients
with Acquired Macrolide-Resistant Mycobacterium abscessus Lung Disease.
Antimicrob Agents Chemother. 2017 Sep 22;61(10). pii: e01146-17. doi:
10.1128/AAC.01146-17. Print 2017 Oct. PubMed PMID: 28739795; PubMed Central
PMCID: PMC5610486.

137: Nduba V, Van't Hoog AH, Mitchell EMH, Borgdorff M, Laserson KF. Incidence of
Active Tuberculosis and Cohort Retention Among Adolescents in Western Kenya.
Pediatr Infect Dis J. 2018 Jan;37(1):10-15. doi: 10.1097/INF.0000000000001685.
PubMed PMID: 28719498.

138: Magee MJ, Sun YV, Brust JCM, Shah NS, Ning Y, Allana S, Campbell A, Hui Q,
Mlisana K, Moodley P, Gandhi NR. Polymorphisms in the vitamin D receptor gene are
associated with reduced rate of sputum culture conversion in multidrug-resistant
tuberculosis patients in South Africa. PLoS One. 2017 Jul 10;12(7):e0180916. doi:
10.1371/journal.pone.0180916. eCollection 2017. PubMed PMID: 28700743; PubMed
Central PMCID: PMC5507304.

139: Gunda DW, Nkandala I, Kavishe GA, Kilonzo SB, Kabangila R, Mpondo BC.
Prevalence and Risk Factors of Delayed Sputum Conversion among Patients Treated

for Smear Positive PTB in Northwestern Rural Tanzania: A Retrospective Cohort
Study. J Trop Med. 2017;2017:5352906. doi: 10.1155/2017/5352906. Epub 2017 Jun
11. PubMed PMID: 28694828; PubMed Central PMCID: PMC5485336.

140: Ganmaa D, Munkhzul B, Fawzi W, Spiegelman D, Willett WC, Bayasgalan P,
Baasansuren E, Buyankhishig B, Oyun-Erdene S, Jolliffe DA, Xenakis T, Bromage S,
Bloom BR, Martineau AR. High-Dose Vitamin D(3) during Tuberculosis Treatment in
Mongolia. A Randomized Controlled Trial. Am J Respir Crit Care Med. 2017 Sep
1;196(5):628-637. doi: 10.1164/rccm.201705-0936OC. PubMed PMID: 28692301; PubMed
Central PMCID: PMC5620670.

141: D'Souza KA, Zaidi SMA, Jaswal M, Butt S, Khowaja S, Habib SS, Malik AA.
Factors associated with month 2 smear non-conversion among Category 1
tuberculosis patients in Karachi, Pakistan. J Infect Public Health. 2018 Mar -
Apr;11(2):283-285. doi: 10.1016/j.jiph.2017.06.009. Epub 2017 Jun 26. PubMed
PMID: 28662884.

142: Zhu L, Yang YZ, Guan HY, Cheng SM, Jin YY, Tan WG, Wu QF, Liu XL, Zhao MG,
Lu ZH, Jia ZW. Trends in drug-resistant tuberculosis after the implementation of
the DOTS strategy in Shenzhen, China, 2000-2013. Int J Tuberc Lung Dis. 2017 Jul
1;21(7):759-765. doi: 10.5588/ijtld.16.0759. PubMed PMID: 28633700.

143: Sharma SK, Katoch K, Sarin R, Balambal R, Kumar Jain N, Patel N, Murthy KJR,
Singla N, Saha PK, Khanna A, Singh U, Kumar S, Sengupta A, Banavaliker JN,
Chauhan DS, Sachan S, Wasim M, Tripathi S, Dutt N, Jain N, Joshi N, Penmesta SRR,
Gaddam S, Gupta S, Khamar B, Dey B, Mitra DK, Arora SK, Bhaskar S, Rani R.
Efficacy and Safety of Mycobacterium indicus pranii as an adjunct therapy in
Category II pulmonary tuberculosis in a randomized trial. Sci Rep. 2017 Jun
13;7(1):3354. doi: 10.1038/s41598-017-03514-1. PubMed PMID: 28611374; PubMed
Central PMCID: PMC5469738.

144: Hasanain AFA, Zayed AAH, Mahdy RE, Nafee AMA. Cholecalciferol for
prophylaxis against antituberculosis therapy-induced liver disorders among naÃ¯ve
patients with pulmonary tuberculosis: A randomized, comparative study. Int J
Mycobacteriol. 2017 Apr-Jun;6(2):149-155. doi: 10.4103/ijmy.ijmy_19_17. PubMed
PMID: 28559516.

145: Eriksson G, Calverley PM, Jenkins CR, Anzueto AR, Make BJ, Lindberg M,
FagerÃ¥s M, Postma DS. The effect of COPD severity and study duration on
exacerbation outcome in randomized controlled trials. Int J Chron Obstruct Pulmon
Dis. 2017 May 15;12:1457-1468. doi: 10.2147/COPD.S130713. eCollection 2017.
PubMed PMID: 28553098; PubMed Central PMCID: PMC5440002.

146: Pan SW, Shu CC, Feng JY, Wang JY, Chan YJ, Yu CJ, Su WJ. Microbiological
Persistence in Patients With Mycobacterium avium Complex Lung Disease: The
Predictors and the Impact on Radiographic Progression. Clin Infect Dis. 2017 Sep
15;65(6):927-934. doi: 10.1093/cid/cix479. PubMed PMID: 28541556.

147: Borisov SE, Dheda K, Enwerem M, Romero Leyet R, D'Ambrosio L, Centis R,
Sotgiu G, Tiberi S, Alffenaar JW, Maryandyshev A, Belilovski E, Ganatra S,
Skrahina A, Akkerman O, Aleksa A, Amale R, Artsukevich J, Bruchfeld J, Caminero
JA, Carpena Martinez I, Codecasa L, Dalcolmo M, Denholm J, Douglas P, Duarte R,
Esmail A, Fadul M, Filippov A, Davies Forsman L, Gaga M, Garcia-Fuertes JA,
GarcÃa-GarcÃa JM, Gualano G, Jonsson J, Kunst H, Lau JS, Lazaro Mastrapa B, Teran
Troya JL, Manga S, Manika K, GonzÃ¡lez Montaner P, Mullerpattan J, Oelofse S,
Ortelli M, Palmero DJ, Palmieri F, Papalia A, Papavasileiou A, Payen MC, Pontali
E, Robalo Cordeiro C, Saderi L, Sadutshang TD, Sanukevich T, Solodovnikova V,
Spanevello A, Topgyal S, Toscanini F, Tramontana AR, Udwadia ZF, Viggiani P,
White V, Zumla A, Migliori GB. Effectiveness and safety of bedaquiline-containing
regimens in the treatment of MDR- and XDR-TB: a multicentre study. Eur Respir J.

2017 May 21;49(5). pii: 1700387. doi: 10.1183/13993003.00387-2017. Print 2017
May. PubMed PMID: 28529205.

148: Salekzamani S, Bavil AS, Mehralizadeh H, Jafarabadi MA, Ghezel A, Gargari
BP. The effects of vitamin D supplementation on proatherogenic inflammatory
markers and carotid intima media thickness in subjects with metabolic syndrome: a
randomized double-blind placebo-controlled clinical trial. Endocrine. 2017
Jul;57(1):51-59. doi: 10.1007/s12020-017-1317-2. Epub 2017 May 16. PubMed PMID:
28509078.

149: Xu P, Chen H, Xu J, Wu M, Zhu X, Wang F, Chen S, Xu J. Moxifloxacin is an
effective and safe candidate agent for tuberculosis treatment: a meta-analysis.
Int J Infect Dis. 2017 Jul;60:35-41. doi: 10.1016/j.ijid.2017.05.003. Epub 2017
May 8. Review. PubMed PMID: 28495364.

150: Ferrian S, Manca C, Lubbe S, Conradie F, Ismail N, Kaplan G, Gray CM,
Fallows D. A combination of baseline plasma immune markers can predict
therapeutic response in multidrug resistant tuberculosis. PLoS One. 2017 May
2;12(5):e0176660. doi: 10.1371/journal.pone.0176660. eCollection 2017. PubMed
PMID: 28464011; PubMed Central PMCID: PMC5413057.

151: Diel R, Ringshausen F, Richter E, Welker L, Schmitz J, Nienhaus A.
Microbiological and Clinical Outcomes of Treating Non-Mycobacterium Avium Complex
Nontuberculous Mycobacterial Pulmonary Disease: A Systematic Review and
Meta-Analysis. Chest. 2017 Jul;152(1):120-142. doi: 10.1016/j.chest.2017.04.166.
Epub 2017 Apr 28. Review. PubMed PMID: 28461147.

152: Vinhas SA, Jones-LÃ³pez EC, Ribeiro Rodrigues R, Gaeddert M, Peres RL,
Marques-Rodrigues P, de Aguiar PPL, White LF, Alland D, Salgame P, Hom D, Ellner
JJ, Dietze R, Collins LF, Shashkina E, Kreiswirth B, Palaci M. Strains of

Mycobacterium tuberculosis transmitting infection in Brazilian households and
those associated with community transmission of tuberculosis. Tuberculosis
(Edinb). 2017 May;104:79-86. doi: 10.1016/j.tube.2017.03.003. Epub 2017 Mar 16.
PubMed PMID: 28454653; PubMed Central PMCID: PMC5716464.

153: Atwine D, Orikiriza P, Taremwa I, Ayebare A, Logoose S, Mwanga-Amumpaire J,
Jindani A, Bonnet M. Predictors of delayed culture conversion among Ugandan
patients. BMC Infect Dis. 2017 Apr 24;17(1):299. doi: 10.1186/s12879-017-2335-7.
PubMed PMID: 28438118; PubMed Central PMCID: PMC5402635.

154: Crowley J, ThaÃ§i D, Joly P, Peris K, Papp KA, Goncalves J, Day RM, Chen R,
Shah K, FerrÃ¡ndiz C, Cather JC. Long-term safety and tolerability of apremilast
in patients with psoriasis: Pooled safety analysis for â‰¥156Â weeks from 2 phase 3,
randomized, controlled trials (ESTEEM 1 and 2). J Am Acad Dermatol. 2017
Aug;77(2):310-317.e1. doi: 10.1016/j.jaad.2017.01.052. Epub 2017 Apr 14. PubMed
PMID: 28416342.

155: Ukwaja KN, Alobu I, Gidado M, Onazi O, Oshi DC. Economic support
intervention improves tuberculosis treatment outcomes in rural Nigeria. Int J
Tuberc Lung Dis. 2017 May 1;21(5):564-570. doi: 10.5588/ijtld.16.0741. PubMed
PMID: 28399972.

156: Miranda P, Gil-Santana L, Oliveira MG, Mesquita ED, Silva E, Rauwerdink A,
Cobelens F, Oliveira MM, Andrade BB, Kritski A. Sustained elevated levels of
C-reactive protein and ferritin in pulmonary tuberculosis patients remaining
culture positive upon treatment initiation. PLoS One. 2017 Apr 6;12(4):e0175278.
doi: 10.1371/journal.pone.0175278. eCollection 2017. PubMed PMID: 28384354;
PubMed Central PMCID: PMC5383283.

157: Jhun BW, Kim SY, Park HY, Jeon K, Shin SJ, Koh WJ. Changes in Serum IgA

Antibody Levels against the Glycopeptidolipid Core Antigen during Antibiotic
Treatment of Mycobacterium avium Complex Lung Disease. Jpn J Infect Dis. 2017 Sep
25;70(5):582-585. doi: 10.7883/yoken.JJID.2016.523. Epub 2017 Mar 28. PubMed
PMID: 28367886.

158: Mahishale V, Avuthu S, Patil B, Lolly M, Eti A, Khan S. Effect of Poor
Glycemic Control in Newly Diagnosed Patients with Smear-Positive Pulmonary
Tuberculosis and Type-2 Diabetes Mellitus. Iran J Med Sci. 2017
Mar;42(2):144-151. PubMed PMID: 28360440; PubMed Central PMCID: PMC5366362.

159: Yang B, Jhun BW, Moon SM, Lee H, Park HY, Jeon K, Kim DH, Kim SY, Shin SJ,
Daley CL, Koh WJ. Clofazimine-Containing Regimen for the Treatment of
Mycobacterium abscessus Lung Disease. Antimicrob Agents Chemother. 2017 May
24;61(6). pii: e02052-16. doi: 10.1128/AAC.02052-16. Print 2017 Jun. PubMed PMID:
28348153; PubMed Central PMCID: PMC5444135.

160: Valenzuela F, de la Cruz Fernandez C, Galimberti RL, GÃ¼rbÃ¼z S,
McKean-Matthews M, Goncalves L, Romiti R. Comparison of ixekizumab with
etanercept or placebo in moderate-to-severe psoriasis: Subgroup analysis of Latin
American patients in the phase 3 randomized UNCOVER-3 study. Actas
Dermosifiliogr. 2017 Jul - Aug;108(6):550-563. doi: 10.1016/j.ad.2017.02.005.
Epub 2017 Mar 23. English, Spanish. PubMed PMID: 28342534.

161: Lu P, Liu Q, Martinez L, Yang H, Lu W, Ding X, Zhu L. Time to sputum culture
conversion and treatment outcome of patients with multidrug-resistant
tuberculosis: a prospective cohort study from urban China. Eur Respir J. 2017 Mar
22;49(3). pii: 1601558. doi: 10.1183/13993003.01558-2016. Print 2017 Mar. PubMed
PMID: 28331033; PubMed Central PMCID: PMC5380874.

162: Bastos ML, Lan Z, Menzies D. An updated systematic review and meta-analysis
for treatment of multidrug-resistant tuberculosis. Eur Respir J. 2017 Mar

22;49(3). pii: 1600803. doi: 10.1183/13993003.00803-2016. Print 2017 Mar. Review.
PubMed PMID: 28331031.

163: ProaÃ±o A, Bravard MA, LÃ³pez JW, Lee GO, Bui D, Datta S, Comina G, Zimic M,
Coronel J, Caviedes L, Cabrera JL, Salas A, Ticona E, Vu NM, Kirwan DE, Loader
MI, Friedland JS, Moore DAJ, Evans CA, Tracey BH, Gilman RH; Tuberculosis Working
Group in Peru. Dynamics of Cough Frequency in Adults Undergoing Treatment for
Pulmonary Tuberculosis. Clin Infect Dis. 2017 May 1;64(9):1174-1181. doi:
10.1093/cid/cix039. PubMed PMID: 28329268; PubMed Central PMCID: PMC5399950.

164: Mundra A, Deshmukh PR, Dawale A. Magnitude and determinants of adverse
treatment outcomes among tuberculosis patients registered under Revised National
Tuberculosis Control Program in a Tuberculosis Unit, Wardha, Central India: A
record-based cohort study. J Epidemiol Glob Health. 2017 Jun;7(2):111-118. doi:
10.1016/j.jegh.2017.02.002. Epub 2017 Mar 18. PubMed PMID: 28315657.

165: Hwang JA, Kim S, Jo KW, Shim TS. Natural history of Mycobacterium avium
complex lung disease in untreated patients with stable course. Eur Respir J. 2017
Mar 8;49(3). pii: 1600537. doi: 10.1183/13993003.00537-2016. Print 2017 Mar.
PubMed PMID: 28275170.

166: Kadota JI, Kurashima A, Suzuki K. The clinical efficacy of a
clarithromycin-based regimen for Mycobacterium avium complex disease: A
nationwide post-marketing study. J Infect Chemother. 2017 May;23(5):293-300. doi:
10.1016/j.jiac.2017.01.007. Epub 2017 Feb 27. PubMed PMID: 28254517.

167: Rockwood N, Wojno J, Ghebrekristos Y, Nicol MP, Meintjes G, Wilkinson RJ.
Utility of Second-Generation Line Probe Assay (Hain MTBDRplus) Directly on
2-Month Sputum Specimens for Monitoring Tuberculosis Treatment Response. J Clin
Microbiol. 2017 May;55(5):1508-1515. doi: 10.1128/JCM.00025-17. Epub 2017 Mar 1.
PubMed PMID: 28249999; PubMed Central PMCID: PMC5405268.

168: Leung CC, Yew WW, Mok TYW, Lau KS, Wong CF, Chau CH, Chan CK, Chang KC, Tam
G, Tam CM. Effects of diabetes mellitus on the clinical presentation and
treatment response in tuberculosis. Respirology. 2017 Aug;22(6):1225-1232. doi:
10.1111/resp.13017. Epub 2017 Feb 28. PubMed PMID: 28244689.

169: Masjedi MR, Hosseini M, Aryanpur M, Mortaz E, Tabarsi P, Soori H, Emami H,
Heidari G, Dizagie MK, Baikpour M. The effects of smoking on treatment outcome in
patients newly diagnosed with pulmonary tuberculosis. Int J Tuberc Lung Dis. 2017
Mar 1;21(3):351-356. doi: 10.5588/ijtld.16.0513. PubMed PMID: 28225348.

170: Scott C, Cavanaugh JS, Silk BJ, Ershova J, Mazurek GH, LoBue PA, Moonan PK.
Comparison of Sputum-Culture Conversion for Mycobacterium bovis and M.
tuberculosis. Emerg Infect Dis. 2017 Mar;23(3):456-462. doi:
10.3201/eid2303.161916. PubMed PMID: 28221125; PubMed Central PMCID: PMC5382750.

171: Rockwood N, Pasipanodya JG, Denti P, Sirgel F, Lesosky M, Gumbo T, Meintjes
G, McIlleron H, Wilkinson RJ. Concentration-Dependent Antagonism and Culture
Conversion in Pulmonary Tuberculosis. Clin Infect Dis. 2017 May
15;64(10):1350-1359. doi: 10.1093/cid/cix158. PubMed PMID: 28205671; PubMed
Central PMCID: PMC5411399.

172: Lee JY, Kim DK, Lee JK, Yoon HI, Jeong I, Heo E, Park YS, Lee JH, Park SS,
Lee SM, Lee CH, Lee J, Choi SM, Park JS, Joh JS, Cho YJ, Lee YJ, Kim SJ, Hwang
YR, Kim H, Ki J, Choi H, Han J, Ahn H, Hahn S, Yim JJ. Substitution of ethambutol
with linezolid during the intensive phase of treatment of pulmonary tuberculosis:
study protocol for a prospective, multicenter, randomized, open-label, phase II
trial. Trials. 2017 Feb 13;18(1):68. doi: 10.1186/s13063-017-1811-0. PubMed PMID:
28193240; PubMed Central PMCID: PMC5307889.

173: Nouhin J, Pean P, Madec Y, Chevalier MF, Didier C, Borand L, Blanc FX,
Scott-Algara D, Laureillard D, Weiss L. Interleukin-1 receptor antagonist, a
biomarker of response to anti-TB treatment in HIV/TB co-infected patients. J
Infect. 2017 May;74(5):456-465. doi: 10.1016/j.jinf.2017.01.016. Epub 2017 Feb 9.
PubMed PMID: 28189712.

174: Lee EH, Lee JM, Kang YA, Leem AY, Kim EY, Jung JY, Park MS, Kim YS, Kim SK,
Chang J, Kim SY. Prevalence and Impact of Diabetes Mellitus Among Patients with
Active Pulmonary Tuberculosis in South Korea. Lung. 2017 Apr;195(2):209-215. doi:
10.1007/s00408-017-9978-4. Epub 2017 Feb 9. PubMed PMID: 28184994.

175: Sudfeld CR, Mugusi F, Aboud S, Nagu TJ, Wang M, Fawzi WW. Efficacy of
vitamin D(3) supplementation in reducing incidence of pulmonary tuberculosis and
mortality among HIV-infected Tanzanian adults initiating antiretroviral therapy:
study protocol for a randomized controlled trial. Trials. 2017 Feb 10;18(1):66.
doi: 10.1186/s13063-017-1819-5. PubMed PMID: 28183335; PubMed Central PMCID:
PMC5301352.

176: Migliori GB, Pontali E, Sotgiu G, Centis R, D'Ambrosio L, Tiberi S, Tadolini
M, Esposito S. Combined Use of Delamanid and Bedaquiline to Treat
Multidrug-Resistant and Extensively Drug-Resistant Tuberculosis: A Systematic
Review. Int J Mol Sci. 2017 Feb 7;18(2). pii: E341. doi: 10.3390/ijms18020341.
Review. PubMed PMID: 28178199; PubMed Central PMCID: PMC5343876.

177: Tenforde MW, Yadav A, Dowdy DW, Gupte N, Shivakoti R, Yang WT, Mwelase N,
Kanyama C, Pillay S, Samaneka W, Santos B, Poongulali S, Tripathy S, Riviere C,
Berendes S, Lama JR, Cardoso SW, Sugandhavesa P, Christian P, Semba RD, Campbell
TB, Gupta A; NWCS319 and ACTG 5175 study team. Vitamin A and D Deficiencies
Associated With Incident Tuberculosis in HIV-Infected Patients Initiating
Antiretroviral Therapy in Multinational Case-Cohort Study. J Acquir Immune Defic

Syndr. 2017 Jul 1;75(3):e71-e79. doi: 10.1097/QAI.0000000000001308.
PubMed PMID:
28169875; PubMed Central PMCID: PMC5472489.

178: Alkabab Y, Keller S, Dodge D, Houpt E, Staley D, Heysell S.
Early
interventions for diabetes related tuberculosis associate with
hastened sputum
microbiological clearance in Virginia, USA. BMC Infect Dis. 2017 Feb
6;17(1):125.
doi: 10.1186/s12879-017-2226-y. PubMed PMID: 28166721; PubMed
Central PMCID:
PMC5294910.

179: Al-Shaer MH, Mansour H, Elewa H, Salameh P, Iqbal F. Treatment
outcomes of
fixed-dose combination versus separate tablet regimens in pulmonary
tuberculosis
patients with or without diabetes in Qatar. BMC Infect Dis. 2017 Feb
2;17(1):118.
doi: 10.1186/s12879-017-2231-1. PubMed PMID: 28152986; PubMed
Central PMCID:
PMC5290647.

180: Ebers A, Stroup S, Mpagama S, Kisonga R, Lekule I, Liu J,
Heysell S.
Determination of plasma concentrations of levofloxacin by high
performance liquid
chromatography for use at a multidrug-resistant tuberculosis
hospital in
Tanzania. PLoS One. 2017 Jan 31;12(1):e0170663. doi:
10.1371/journal.pone.0170663. eCollection 2017. PubMed PMID:
28141813; PubMed
Central PMCID: PMC5283651.

181: Savic RM, Weiner M, MacKenzie WR, Engle M, Whitworth WC,
Johnson JL, Nsubuga
P, Nahid P, Nguyen NV, Peloquin CA, Dooley KE, Dorman SE;
Tuberculosis Trials
Consortium of the Centers for Disease Control and Prevention.
Defining the
optimal dose of rifapentine for pulmonary tuberculosis: Exposure-
response
relations from two phase II clinical trials. Clin Pharmacol Ther.
2017
Aug;102(2):321-331. doi: 10.1002/cpt.634. Epub 2017 Mar 2. PubMed
PMID: 28124478;
PubMed Central PMCID: PMC5545752.

182: Ko Y, Shin JH, Lee HK, Lee YS, Lee SY, Park SY, Mo EK, Kim C,
Park YB.

Duration of Pulmonary Tuberculosis Infectiousness under Adequate Therapy, as
Assessed Using Induced Sputum Samples. Tuberc Respir Dis (Seoul). 2017
Jan;80(1):27-34. doi: 10.4046/trd.2017.80.1.27. Epub 2016 Dec 30. PubMed PMID:
28119744; PubMed Central PMCID: PMC5256343.

183: SchÃ¶n T, Chryssanthou E. Minimum inhibitory concentration distributions for
Mycobacterium avium complex-towards evidence-based susceptibility breakpoints.
Int J Infect Dis. 2017 Feb;55:122-124. doi: 10.1016/j.ijid.2016.12.027. Epub 2017
Jan 6. PubMed PMID: 28069470.

184: Toujani S, Ben Safta B, Ben Salah N, Mjid M, Ouahchi Y, Louzir B, Daghfous
J, Cherif J, Mehiri N, Beji M. Contribution of fixed-dose combinations in the
treatment of tuberculosis. Tunis Med. 2016 Jul;94(7):401-405. PubMed PMID:
28051229.

185: MuÃ±oz-Torrico M, Caminero Luna J, Migliori GB, D'Ambrosio L,
Carrillo-Alduenda JL, Villareal-Velarde H, Torres-Cruz A, Flores-Ergara H,
MartÃ-nez-Mendoza D, GarcÃ-a-Sancho C, Centis R, Salazar-Lezama MÃ, PÃ©rez-Padilla
R. Comparison of bacteriological conversion and treatment outcomes among MDR-TB
patients with and without diabetes in Mexico: Preliminary data. Rev Port Pneumol
(2006). 2017 Jan - Feb;23(1):27-30. doi: 10.1016/j.rppnen.2016.11.009. Epub 2016
Dec 31. PubMed PMID: 28043788.

186: Skrahina A, Hurevich H, Falzon D, Zhilevich L, Rusovich V, Dara M, Setkina
S. Bedaquiline in the multidrug-resistant tuberculosis treatment: Belarus
experience. Int J Mycobacteriol. 2016 Dec;5 Suppl 1:S62-S63. doi:
10.1016/j.ijmyco.2016.11.014. Epub 2016 Nov 23. PubMed PMID: 28043617.

187: Fennelly KP. What is in a cough? Int J Mycobacteriol. 2016 Dec;5 Suppl
1:S51. doi: 10.1016/j.ijmyco.2016.10.037. Epub 2016 Nov 25. PubMed PMID:
28043607.

188: Diarra B, CissÃ© AB, Kodio O, Sanogo M, Baya B, Togo AC, Somboro A,
TolofoudiÃ© M, Degoga B, Keita ML, Diallo F, Nguiakam N, Coulibaly G, Bane S,
Sarro YD, Doumbia S, Murphy RL, Diallo S, Dejong BC. Screening new tuberculosis
patients in Mali for rifampicin resistance at 2months. Int J Mycobacteriol. 2016
Dec;5 Suppl 1:S42-S43. doi: 10.1016/j.ijmyco.2016.09.052. Epub 2016 Nov 11.
PubMed PMID: 28043602.

189: Hu Y, Zheng X, Ning Z, Li Q, Zhang Z, Hoffner S. Impact of genotypic and
phenotypic resistance to second-line anti-tuberculosis drugs on treatment
outcomes in multidrug-resistant tuberculosis in China. Int J Mycobacteriol. 2016
Dec;5 Suppl 1:S34-S35. doi: 10.1016/j.ijmyco.2016.11.007. Epub 2016 Nov 22.
PubMed PMID: 28043597.

190: Calverley PM, Eriksson G, Jenkins CR, Anzueto AR, Make BJ, Persson A,
FagerÃ¥s M, Postma DS. Early efficacy of budesonide/formoterol in patients with
moderate-to-very-severe COPD. Int J Chron Obstruct Pulmon Dis. 2016 Dec
19;12:13-25. doi: 10.2147/COPD.S114209. eCollection 2017. PubMed PMID: 28031707;
PubMed Central PMCID: PMC5182036.

191: Zheng X, Ning Z, Drobniewski F, Yang J, Li Q, Zhang Z, Hu Y. pncA mutations
are associated with slower sputum conversion during standard treatment of
multidrug-resistant tuberculosis. Int J Antimicrob Agents. 2017
Feb;49(2):183-188. doi: 10.1016/j.ijantimicag.2016.10.012. Epub 2016 Nov 24.
PubMed PMID: 28012685.

192: Park J, Cho J, Lee CH, Han SK, Yim JJ. Progression and Treatment Outcomes of
Lung Disease Caused by Mycobacterium abscessus and Mycobacterium massiliense.
Clin Infect Dis. 2017 Feb 1;64(3):301-308. doi: 10.1093/cid/ciw723. Epub 2016 Nov
10. Erratum in: Clin Infect Dis. 2017 May 15;64(10):1469. PubMed PMID: 28011609.

193: Koh WJ, Jeong BH, Kim SY, Jeon K, Park KU, Jhun BW, Lee H, Park HY, Kim DH,
Huh HJ, Ki CS, Lee NY, Kim HK, Choi YS, Kim J, Lee SH, Kim CK, Shin SJ, Daley CL,
Kim H, Kwon OJ. Mycobacterial Characteristics and Treatment Outcomes in
Mycobacterium abscessus Lung Disease. Clin Infect Dis. 2017 Feb 1;64(3):309-316.
doi: 10.1093/cid/ciw724. Epub 2016 Nov 10. PubMed PMID: 28011608.

194: Vashakidze S, Despuig A, Gogishvili S, Nikolaishvili K, Shubladze N,
Avaliani Z, Tukvadze N, Casals M, CaylÃ JA, Cardona PJ, Vilaplana C.
Retrospective study of clinical and lesion characteristics of patients undergoing
surgical treatment for Pulmonary Tuberculosis in Georgia. Int J Infect Dis. 2017
Mar;56:200-207. doi: 10.1016/j.ijid.2016.12.009. Epub 2016 Dec 19. Review. PubMed
PMID: 28007659; PubMed Central PMCID: PMC5660856.

195: Mbatchou Ngahane BH, Dahirou F, Tchieche C, Wandji A, NgniÃ© C,
Nana-Metchedjin A, NyankiyÃ© E, Endale Mangamba ML, Kuaban C. Clinical
characteristics and outcomes of tuberculosis in Douala, Cameroon: a 7-year
retrospective cohort study. Int J Tuberc Lung Dis. 2016 Dec 1;20(12):1609-1614.
doi: 10.5588/ijtld.15.0731. PubMed PMID: 28000583.

196: Arnold A, Cooke GS, Kon OM, Dedicoat M, Lipman M, Loyse A, Butcher PD, Ster
IC, Harrison TS. Drug resistant TB: UK multicentre study (DRUMS): Treatment,
management and outcomes in London and West Midlands 2008-2014. J Infect. 2017
Mar;74(3):260-271. doi: 10.1016/j.jinf.2016.12.005. Epub 2016 Dec 18. PubMed
PMID: 27998752.

197: Liebenberg JJ, Dold CJ, Olivier LR. A prospective investigation into the
effect of colchicine on tuberculous pericarditis. Cardiovasc J Afr. 2016
Nov/Dec;27(6):350-355. doi: 10.5830/CVJA-2016-035. PubMed PMID: 27965998; PubMed
Central PMCID: PMC5412665.

198: Velayutham B, Nair D, Kannan T, Padmapriyadarsini C, Sachdeva KS, Bency J,

Klinton JS, Haldar S, Khanna A, Jayasankar S, Swaminathan S. Factors associated
with sputum culture conversion in multidrug-resistant pulmonary tuberculosis. Int
J Tuberc Lung Dis. 2016 Dec;20(12):1671-1676. PubMed PMID: 27931345.

199: Park HO, Kim SH, Moon SH, Byun JH, Kim JW, Lee CE, Kim JD, Jang IS, Yang JH.
Association between Body Mass Index and Sputum Culture Conversion among South
Korean Patients with Multidrug Resistant Tuberculosis in a Tuberculosis Referral
Hospital. Infect Chemother. 2016 Dec;48(4):317-323. doi:
10.3947/ic.2016.48.4.317. Epub 2016 Nov 21. PubMed PMID: 27883374; PubMed Central
PMCID: PMC5204011.

200: Batbold U, Butov DO, Kutsyna GA, Damdinpurev N, Grinishina EA, Mijiddorj O,
Kovolev ME, Baasanjav K, Butova TS, Sandagdorj M, Batbold O, Tseveendorj A, Chunt
E, Zaitzeva SI, Stepanenko HL, Makeeva NI, Mospan IV, Pylypchuk VS, Rowe JL,
Nyasulu P, Jirathitikal V, Bain AI, Tarakanovskaya MG, Bourinbaiar AS.
Double-blind, placebo-controlled, 1:1 randomized Phase III clinical trial of
Immunoxel honey lozenges as an adjunct immunotherapy in 269 patients with
pulmonary tuberculosis. Immunotherapy. 2017 Jan;9(1):13-24. doi:
10.2217/imt-2016-0079. Epub 2016 Nov 21. PubMed PMID: 27868466.

201: Padmapriyadarsini C, Shobana M, Lakshmi M, Beena T, Swaminathan S.
Undernutrition & tuberculosis in India: Situation analysis & the way forward.
Indian J Med Res. 2016 Jul;144(1):11-20. doi: 10.4103/0971-5916.193278. Review.
PubMed PMID: 27834321; PubMed Central PMCID: PMC5116882.

202: Vora A, Patel S, Patel K. Role of Risorine in the Treatment of Drug -
Susceptible Pulmonary Tuberculosis: A Pilot Study. J Assoc Physicians India. 2016
Nov;64(11):20-24. PubMed PMID: 27805329.

203: Okumura M, Yoshiyama T. [EXPERIENCE OF USE OF NEW ANTI-TUBERCULOUS DRUG,
DELAMANID IN MULTI- AND EXTENSIVELY DRUG RESISTANT TUBERCULOSIS CASES IN OUR

HOSPITAL]. Kekkaku. 2016 Nov;91(11-12):699-702. Japanese. PubMed
PMID: 30648372.

204: Choi R, Kim K, Kim MJ, Kim SY, Kwon OJ, Jeon K, Park HY, Jeong BH, Shin SJ,
Koh WJ, Lee SY. Serum inflammatory profiles in pulmonary tuberculosis and their
association with treatment response. J Proteomics. 2016 Oct 21;149:23-30. doi:
10.1016/j.jprot.2016.06.016. Epub 2016 Jun 15. PubMed PMID: 27321581.

205: Salekzamani S, Mehralizadeh H, Ghezel A, Salekzamani Y, Jafarabadi MA, Bavil
AS, Gargari BP. Effect of high-dose vitamin D supplementation on cardiometabolic
risk factors in subjects with metabolic syndrome: a randomized controlled
double-blind clinical trial. J Endocrinol Invest. 2016 Nov;39(11):1303-1313. Epub
2016 Jul 11. PubMed PMID: 27400997.

206: Olivier KN, Griffith DE, Eagle G, McGinnis JP 2nd, Micioni L, Liu K, Daley
CL, Winthrop KL, Ruoss S, Addrizzo-Harris DJ, Flume PA, Dorgan D, Salathe M,
Brown-Elliott BA, Gupta R, Wallace RJ Jr. Randomized Trial of Liposomal Amikacin
for Inhalation in Nontuberculous Mycobacterial Lung Disease. Am J Respir Crit
Care Med. 2017 Mar 15;195(6):814-823. doi: 10.1164/rccm.201604-0700OC. PubMed
PMID: 27748623; PubMed Central PMCID: PMC5363966.

207: Fletcher R, Jones JD, Shah NS. Treatment of Active Tuberculosis in Chicago,
2008-2011: The Role of Public Health Departments. PLoS One. 2016 Oct
12;11(10):e0164162. doi: 10.1371/journal.pone.0164162. eCollection 2016. PubMed
PMID: 27732650; PubMed Central PMCID: PMC5061361.

208: PaiÃ£o DS, Lemos EF, Carbone AD, Sgarbi RV, Junior AL, da Silva FM, BrandÃ£o
LM, Dos Santos LS, Martins VS, Simionatto S, Motta-Castro AR, PompÃlio MA, Urrego
J, Ko AI, Andrews JR, Croda J. Impact of mass-screening on tuberculosis incidence
in a prospective cohort of Brazilian prisoners. BMC Infect Dis. 2016 Oct
3;16(1):533. PubMed PMID: 27716170; PubMed Central PMCID: PMC5048439.

209: Sara C, Elsa H, Baijayanti M, Lennartsdotter EM. Clinical Correlates and
Drug Resistance in HIV-Infected and -Uninfected Pulmonary Tuberculosis Patients
in South India. World J AIDS. 2016 Sep;6(3):87-100. Epub 2016 Sep 9. PubMed PMID:
27708985; PubMed Central PMCID: PMC5047007.

210: Yadav S, Rawal G, Baxi M. Bedaquiline: A Novel Antitubercular Agent for the
Treatment of Multidrug-Resistant Tuberculosis. J Clin Diagn Res. 2016
Aug;10(8):FM01-2. doi: 10.7860/JCDR/2016/19052.8286. Epub 2016 Aug 1. PubMed
PMID: 27656462; PubMed Central PMCID: PMC5028565.

211: Narendran G, Kavitha D, Karunaianantham R, Gil-Santana L, Almeida-Junior JL,
Reddy SD, Kumar MM, Hemalatha H, Jayanthi NN, Ravichandran N, Krishnaraja R,
Prabhakar A, Manoharan T, Nithyananthan L, Arjunan G, Natrajan M, Swaminathan S,
Andrade BB. Role of LTA4H Polymorphism in Tuberculosis-Associated Immune
Reconstitution Inflammatory Syndrome Occurrence and Clinical Severity in Patients
Infected with HIV. PLoS One. 2016 Sep 19;11(9):e0163298. doi:
10.1371/journal.pone.0163298. eCollection 2016. PubMed PMID: 27643598; PubMed
Central PMCID: PMC5028072.

212: Siddiqui AN, Khayyam KU, Sharma M. Effect of Diabetes Mellitus on
Tuberculosis Treatment Outcome and Adverse Reactions in Patients Receiving
Directly Observed Treatment Strategy in India: A Prospective Study. Biomed Res
Int. 2016;2016:7273935. doi: 10.1155/2016/7273935. Epub 2016 Aug 24. PubMed PMID:
27642601; PubMed Central PMCID: PMC5013229.

213: Rathee D, Arora P, Meena M, Sarin R, Chakraborty P, Jaiswal A, Goyal M.
Comparative study of clinico-bacterio-radiological profile and treatment outcome
of smokers and nonsmokers suffering from pulmonary tuberculosis. Lung India. 2016
Sep-Oct;33(5):507-11. doi: 10.4103/0970-2113.188970. PubMed PMID: 27625444;
PubMed Central PMCID: PMC5006330.

214: Feng M, Ding Q, Zhong C, Li J, Wang Q, Yuan Z, Dong Y. Adjunctive therapy
with V-5 Immunitor (V5) for the treatment of tuberculosis patients: a
meta-analysis. Pharmazie. 2016 Sep 1;71(9):499-503. doi: 10.1691/ph.2016.6051.
Review. PubMed PMID: 29441844.

215: Yoon YS, Jung JW, Jeon EJ, Seo H, Ryu YJ, Yim JJ, Kim YH, Lee BH, Park YB,
Lee BJ, Kang H, Choi JC. The effect of diabetes control status on treatment
response in pulmonary tuberculosis: a prospective study. Thorax. 2017
Mar;72(3):263-270. doi: 10.1136/thoraxjnl-2015-207686. Epub 2016 Aug 23. PubMed
PMID: 27553224.

216: Nguyen QD, Merrill PT, Jaffe GJ, Dick AD, Kurup SK, Sheppard J, Schlaen A,
Pavesio C, Cimino L, Van Calster J, Camez AA, Kwatra NV, Song AP, Kron M, Tari S,
BrÃ©zin AP. Adalimumab for prevention of uveitic flare in patients with inactive
non-infectious uveitis controlled by corticosteroids (VISUAL II): a multicentre,
double-masked, randomised, placebo-controlled phase 3 trial. Lancet. 2016 Sep
17;388(10050):1183-92. doi: 10.1016/S0140-6736(16)31339-3. Epub 2016 Aug 16.
Erratum in: Lancet. 2016 Sep 17;388(10050):1160. PubMed PMID: 27542302.

217: Shenai S, Ronacher K, Malherbe S, Stanley K, Kriel M, Winter J, Peppard T,
Barry CE, Wang J, Dodd LE, Via LE, Barry CE 3rd, Walzl G, Alland D. Bacterial
Loads Measured by the Xpert MTB/RIF Assay as Markers of Culture Conversion and
Bacteriological Cure in Pulmonary TB. PLoS One. 2016 Aug 10;11(8):e0160062. doi:
10.1371/journal.pone.0160062. eCollection 2016. PubMed PMID: 27508390; PubMed
Central PMCID: PMC4980126.

218: Namkoong H, Morimoto K, Nishimura T, Tanaka H, Sugiura H, Yamada Y, Kurosaki
A, Asakura T, Suzuki S, Fujiwara H, Yagi K, Ishii M, Tasaka S, Betsuyaku T,

Hoshino Y, Kurashima A, Hasegawa N. Clinical efficacy and safety of multidrug
therapy including thrice weekly intravenous amikacin administration for
Mycobacterium abscessus pulmonary disease in outpatient settings: a case series.
BMC Infect Dis. 2016 Aug 9;16:396. doi: 10.1186/s12879-016-1689-6. PubMed PMID:
27506679; PubMed Central PMCID: PMC4977760.

219: Micheletti VC, Kritski AL, Braga JU. Clinical Features and Treatment
Outcomes of Patients with Drug-Resistant and Drug-Sensitive Tuberculosis: A
Historical Cohort Study in Porto Alegre, Brazil. PLoS One. 2016 Aug
9;11(8):e0160109. doi: 10.1371/journal.pone.0160109. eCollection 2016. PubMed
PMID: 27505633; PubMed Central PMCID: PMC4978410.

220: Cai L, Gu J, Zheng J, Zheng M, Wang G, Xi LY, Hao F, Liu XM, Sun QN, Wang Y,
Lai W, Fang H, Tu YT, Sun Q, Chen J, Gao XH, Gu Y, Teixeira HD, Zhang JZ, Okun
MM. Efficacy and safety of adalimumab in Chinese patients with moderate-to-severe
plaque psoriasis: results from a phase 3, randomized, placebo-controlled,
double-blind study. J Eur Acad Dermatol Venereol. 2017 Jan;31(1):89-95. doi:
10.1111/jdv.13746. Epub 2016 Aug 9. PubMed PMID: 27504914; PubMed Central PMCID:
PMC5215651.

221: Mesquita ED, Gil-Santana L, Ramalho D, Tonomura E, Silva EC, Oliveira MM,
Andrade BB, Kritski A; Rede-TB Study group. Associations between systemic
inflammation, mycobacterial loads in sputum and radiological improvement after
treatment initiation in pulmonary TB patients from Brazil: a prospective cohort
study. BMC Infect Dis. 2016 Aug 5;16:368. doi: 10.1186/s12879-016-1736-3. PubMed
PMID: 27494953; PubMed Central PMCID: PMC4974760.

222: Honarvar MR, Eghtesadi S, Gill P, Jazayeri S, Vakili MA, Shamsardekani MR,
Abbasi A. The effect of green tea extract supplementation on sputum smear
conversion and weight changes in pulmonary TB patients: A randomized controlled

trial. Med J Islam Repub Iran. 2016 Jun 1;30:381. eCollection 2016. PubMed PMID:
27493925; PubMed Central PMCID: PMC4972068.

223: Vogelmeier C, Paggiaro PL, Dorca J, Sliwinski P, Mallet M, Kirsten AM, Beier
J, Seoane B, Segarra RM, Leselbaum A. Efficacy and safety of aclidinium/formoterol versus salmeterol/fluticasone: a phase 3 COPD study. Eur
Respir J. 2016 Oct;48(4):1030-1039. doi: 10.1183/13993003.00216-2016. Epub 2016
Aug 4. PubMed PMID: 27492833.

224: Mlotshwa M, Abraham N, Beery M, Williams S, Smit S, Uys M, Reddy C,
Medina-Marino A. Risk factors for tuberculosis smear non-conversion in Eden
district, Western Cape, South Africa, 2007-2013: a retrospective cohort study.
BMC Infect Dis. 2016 Aug 2;16:365. doi: 10.1186/s12879-016-1712-y. PubMed PMID:
27484399; PubMed Central PMCID: PMC4971671.

225: Arbex MA, Bonini EH, Kawakame Pirolla G, D'Ambrosio L, Centis R, Migliori
GB. Effectiveness and safety of imipenem/clavulanate and linezolid to treat
multidrug and extensively drug-resistant tuberculosis at a referral hospital in
Brazil. Rev Port Pneumol (2006). 2016 Nov - Dec;22(6):337-341. doi:
10.1016/j.rppnen.2016.06.006. Epub 2016 Jul 29. PubMed PMID: 27481315.

226: Shadmehr MB, Abbasidezfouli A, Farzanegan R, Pejhan S, Daneshvar Kakhaki A,
Sheikhy K, Saghebi SR, Sadeghbeigee F, Gharedaghi A, Jahanshahi N, Zangi M. The
Role of Systemic Steroids in Postintubation Tracheal Stenosis: A Randomized
Clinical Trial. Ann Thorac Surg. 2017 Jan;103(1):246-253. doi:
10.1016/j.athoracsur.2016.05.063. Epub 2016 Jul 29. PubMed PMID: 27476818.

227: Modongo C, Pasipanodya JG, Magazi BT, Srivastava S, Zetola NM, Williams SM,
Sirugo G, Gumbo T. Artificial Intelligence and Amikacin Exposures Predictive of
Outcomes in Multidrug-Resistant Tuberculosis Patients. Antimicrob Agents
Chemother. 2016 Sep 23;60(10):5928-32. doi: 10.1128/AAC.00962-16. Print 2016 Oct.

PubMed PMID: 27458224; PubMed Central PMCID: PMC5038293.

228: Lee HW, Lee JK, Kim E, Yim JJ, Lee CH. The Effectiveness and Safety of
Fluoroquinolone-Containing Regimen as a First-Line Treatment for Drug-Sensitive
Pulmonary Tuberculosis: A Systematic Review and Meta-Analysis. PLoS One. 2016 Jul
25;11(7):e0159827. doi: 10.1371/journal.pone.0159827. eCollection 2016. Review.
PubMed PMID: 27455053; PubMed Central PMCID: PMC4959712.

229: Bunkar ML, Agnihotri SP, Gupta PR, Arya S. Add-on prednisolone in the
management of cervical lymph node tuberculosis. Indian J Tuberc. 2016
Apr;63(2):96-9. doi: 10.1016/j.ijtb.2015.05.002. Epub 2016 Jun 4. PubMed PMID:
27451818.

230: Salindri AD, Kipiani M, Kempker RR, Gandhi NR, Darchia L, Tukvadze N,
Blumberg HM, Magee MJ. Diabetes Reduces the Rate of Sputum Culture Conversion in
Patients With Newly Diagnosed Multidrug-Resistant Tuberculosis. Open Forum Infect
Dis. 2016 Jun 16;3(3):ofw126. doi: 10.1093/ofid/ofw126. eCollection 2016 Sep.
PubMed PMID: 27419188; PubMed Central PMCID: PMC4942763.

231: Gualano G, Capone S, Matteelli A, Palmieri F. New Antituberculosis Drugs:
From Clinical Trial to Programmatic Use. Infect Dis Rep. 2016 Jun 24;8(2):6569.
doi: 10.4081/idr.2016.6569. eCollection 2016 Jun 24. Review. PubMed PMID:
27403268; PubMed Central PMCID: PMC4927937.

232: Radovic M, Ristic L, Ciric Z, Dinic-Radovic V, Stankovic I, Pejcic T, Rancic
M, Bogdanovic D. Changes in respiratory function impairment following the
treatment of severe pulmonary tuberculosis - limitations for the underlying COPD
detection. Int J Chron Obstruct Pulmon Dis. 2016 Jun 16;11:1307-16. doi:
10.2147/COPD.S106875. eCollection 2016. PubMed PMID: 27366058; PubMed Central
PMCID: PMC4914072.

233: Howard AA, Hirsch-Moverman Y, Frederix K, Daftary A, Saito S, Gross T, Wu Y,
Maama LB. The START Study to evaluate the effectiveness of a combination
intervention package to enhance antiretroviral therapy uptake and retention
during TB treatment among TB/HIV patients in Lesotho: rationale and design of a
mixed-methods, cluster-randomized trial. Glob Health Action. 2016 Jun 27;9:31543.
doi: 10.3402/gha.v9.31543. eCollection 2016. PubMed PMID: 27357074; PubMed
Central PMCID: PMC4926099.

234: Grobler L, Nagpal S, Sudarsanam TD, Sinclair D. Nutritional supplements for
people being treated for active tuberculosis. Cochrane Database Syst Rev. 2016
Jun 29;(6):CD006086. doi: 10.1002/14651858.CD006086.pub4. Review. PubMed PMID:
27355911; PubMed Central PMCID: PMC4981643.

235: Aseffa A, Chukwu JN, Vahedi M, Aguwa EN, Bedru A, Mebrahtu T, Ezechi OC,
Yimer G, Yamuah LK, Medhin G, Connolly C, Rida W, Aderaye G, Zumla AI, Onyebujoh
PC; 4FDC Study Group. Efficacy and Safety of 'Fixed Dose' versus 'Loose' Drug
Regimens for Treatment of Pulmonary Tuberculosis in Two High TB-Burden African
Countries: A Randomized Controlled Trial. PLoS One. 2016 Jun 20;11(6):e0157434.
doi: 10.1371/journal.pone.0157434. eCollection 2016. PubMed PMID: 27322164;
PubMed Central PMCID: PMC4913909.

236: Xu L, Cui G, Jia H, Zhu Y, Ding Y, Chen J, Lu C, Ye P, Gao H, Li L, Ma W,
Lyu J, Diao H. Decreased IL-17 during treatment of sputumÂ smear-positive
pulmonary tuberculosisÂ due to increased regulatory T cells and IL-10. J Transl
Med. 2016 Jun 16;14(1):179. doi: 10.1186/s12967-016-0909-6. PubMed PMID:
27311307; PubMed Central PMCID: PMC4911683.

237: Koethe JR, von Reyn CF. Protein-calorie malnutrition, macronutrient
supplements, and tuberculosis. Int J Tuberc Lung Dis. 2016 Jul;20(7):857-63. doi:
10.5588/ijtld.15.0936. Review. PubMed PMID: 27287634.

238: Simonovska L, Ilievska-Popovska B. Comparison of Results from Inpatient and
Outpatient Treatment of Tuberculosis in Republic of Macedonia. Open Access Maced
J Med Sci. 2015 Jun 15;3(2):337-40. doi: 10.3889/oamjms.2015.050. Epub 2015 Apr
30. PubMed PMID: 27275247; PubMed Central PMCID: PMC4877879.

239: Bidaki R, Zarepur E, Akrami M, Mohammad M. Functional Neurological Symptom
Disorder: Mismanagement, Misdiagnosis, Chronic Cough Following Sexual Abuse: A
Rare Case Report. Iran J Child Neurol. 2016 Spring;10(2):90-2. PubMed PMID:
27247590; PubMed Central PMCID: PMC4885161.

240: MÃ©chaÃ¯ F, Figoni J, Leblanc C, Gousseff M, Vignier N, Bouchaud O. Active
pulmonary tuberculosis: Role for amikacin in early treatment. Med Mal Infect.
2016 Sep;46(6):318-21. doi: 10.1016/j.medmal.2016.04.010. Epub 2016 May 24.
PubMed PMID: 27235009.

241: Hejazi ME, Modarresi-Ghazani F, Hamishehkar H, Mesgari-Abbasi M, Dousti S,
Entezari-Maleki T. The Effect of Treatment of Vitamin D Deficiency on the Level
of P-Selectin and hs-CRP in Patients With Thromboembolism: A Pilot Randomized
Clinical Trial. J Clin Pharmacol. 2017 Jan;57(1):40-47. doi: 10.1002/jcph.774.
Epub 2016 Jun 28. PubMed PMID: 27225617.

242: Tamura A. TUBERCULOSIS AND LUNG CANCER. Kekkaku. 2016 Jan;91(1):17-25.
PubMed PMID: 27192776.

243: Gallardo CR, Rigau Comas D, Valderrama RodrÃguez A, RoquÃ© i Figuls M, Parker
LA, CaylÃ J, Bonfill Cosp X. Fixed-dose combinations of drugs versus single-drug
formulations for treating pulmonary tuberculosis. Cochrane Database Syst Rev.
2016 May 17;(5):CD009913. doi: 10.1002/14651858.CD009913.pub2. Review. PubMed
PMID: 27186634; PubMed Central PMCID: PMC4916937.

244: Koh WJ, Jeong BH, Jeon K, Kim SY, Park KU, Park HY, Huh HJ, Ki CS, Lee NY,
Lee SH, Kim CK, Daley CL, Shin SJ, Kim H, Kwon OJ. Oral Macrolide Therapy
Following Short-term Combination Antibiotic Treatment of Mycobacterium
massiliense Lung Disease. Chest. 2016 Dec;150(6):1211-1221. doi:
10.1016/j.chest.2016.05.003. Epub 2016 May 7. PubMed PMID: 27167209.

245: Conde MB, Mello FC, Duarte RS, Cavalcante SC, Rolla V, Dalcolmo M, Loredo C,
Durovni B, Armstrong DT, Efron A, Barnes GL, Marzinke MA, Savic RM, Dooley KE,
Cohn S, Moulton LH, Chaisson RE, Dorman SE. A Phase 2 Randomized Trial of a
Rifapentine plus Moxifloxacin-Based Regimen for Treatment of Pulmonary
Tuberculosis. PLoS One. 2016 May 9;11(5):e0154778. doi:
10.1371/journal.pone.0154778. eCollection 2016. PubMed PMID: 27159505; PubMed
Central PMCID: PMC4861335.

246: Sgaragli G, Frosini M, Saponara S, Corelli F. Human Tuberculosis. III.
Current and Prospective Approaches in Anti-Tubercular Therapy. Curr Med Chem.
2016;23(21):2245-74. Review. PubMed PMID: 27142291.

247: Kwon YS, Koh WJ. Diagnosis and Treatment of Nontuberculous Mycobacterial
Lung Disease. J Korean Med Sci. 2016 May;31(5):649-59. doi:
10.3346/jkms.2016.31.5.649. Epub 2016 Mar 22. Review. PubMed PMID: 27134484;
PubMed Central PMCID: PMC4835588.

248: Charan J, Reljic T, Kumar A. Bedaquiline versus placebo for management of
multiple drug-resistant tuberculosis: A systematic review. Indian J Pharmacol.
2016 Mar-Apr;48(2):186-91. doi: 10.4103/0253-7613.178839. Review. PubMed PMID:
27127322; PubMed Central PMCID: PMC4825437.

249: Barogui YT, Klis SA, Johnson RC, Phillips RO, van der Veer E, van Diemen C,
van der Werf TS, Stienstra Y. Genetic Susceptibility and Predictors of
Paradoxical Reactions in Buruli Ulcer. PLoS Negl Trop Dis. 2016 Apr
20;10(4):e0004594. doi: 10.1371/journal.pntd.0004594. eCollection 2016 Apr.
PubMed PMID: 27097163; PubMed Central PMCID: PMC4838240.

250: Kulkarni RA, Deshpande AR. Anti-inflammatory and antioxidant effect of
ginger in tuberculosis. J Complement Integr Med. 2016 Jun 1;13(2):201-6. doi:
10.1515/jcim-2015-0032. PubMed PMID: 27089418.

251: Tiberi S, Sotgiu G, D'Ambrosio L, Centis R, Abdo Arbex M, Alarcon Arrascue
E, Alffenaar JW, Caminero JA, Gaga M, Gualano G, Skrahina A, Solovic I, Sulis G,
Tadolini M, Alarcon Guizado V, De Lorenzo S, Roby Arias AJ, Scardigli A, Akkerman
OW, Aleksa A, Artsukevich J, Auchynka V, Bonini EH, Chong MarÃn FA, Collahuazo
LÃ³pez L, de Vries G, Dore S, Kunst H, Matteelli A, Moschos C, Palmieri F,
Papavasileiou A, Payen MC, Piana A, Spanevello A, Vargas Vasquez D, Viggiani P,
White V, Zumla A, Migliori GB. Comparison of effectiveness and safety of
imipenem/clavulanate- versus meropenem/clavulanate-containing regimens in the
treatment of MDR-Â andÂ XDR-TB. Eur Respir J. 2016 Jun;47(6):1758-66. doi:
10.1183/13993003.00214-2016. Epub 2016 Apr 13. PubMed PMID: 27076583.

252: Qin F, Barry PM, Pascopella L. Factors associated with extended treatment
among tuberculosis patients at risk of relapse in California. Int J Tuberc Lung
Dis. 2016 Mar;20(3):363-9. doi: 10.5588/ijtld.15.0469. PubMed PMID: 27046718.

253: Musselwhite LW, Andrade BB, Ellenberg SS, Tierney A, Belaunzaran-Zamudio PF,
Rupert A, Lederman MM, Sanne I, Sierra Madero JG, Sereti I. Vitamin D, D-dimer,
Interferon Î³, and sCD14 Levels are Independently Associated with Immune
Reconstitution Inflammatory Syndrome: A Prospective, International Study.
EBioMedicine. 2016 Jan 14;4:115-23. doi: 10.1016/j.ebiom.2016.01.016. eCollection
2016 Feb. PubMed PMID: 26981576; PubMed Central PMCID: PMC4776072.

254: Swaminathan A, du Cros P, Seddon JA, Quinnell S, Bobokhojaev OI, Dusmatova
Z, Achar J. Treating children for drug-resistant tuberculosis in Tajikistan with

Group 5 medications. Int J Tuberc Lung Dis. 2016 Apr;20(4):474-8. doi:
10.5588/ijtld.15.0666. PubMed PMID: 26970156.

255: Tiberi S, Payen MC, Sotgiu G, D'Ambrosio L, Alarcon Guizado V, Alffenaar JW,
Abdo Arbex M, Caminero JA, Centis R, De Lorenzo S, Gaga M, Gualano G, Roby Arias
AJ, Scardigli A, Skrahina A, Solovic I, Sulis G, Tadolini M, Akkerman OW, Alarcon
Arrascue E, Aleska A, Avchinko V, Bonini EH, Chong MarÃn FA, Collahuazo LÃ³pez L,
de Vries G, Dore S, Kunst H, Matteelli A, Moschos C, Palmieri F, Papavasileiou A,
Spanevello A, Vargas Vasquez D, Viggiani P, White V, Zumla A, Migliori GB.
Effectiveness and safety of meropenem/clavulanate-containing regimens in the
treatment of MDR- and XDR-TB. Eur Respir J. 2016 Apr;47(4):1235-43. doi:
10.1183/13993003.02146-2015. Epub 2016 Mar 10. PubMed PMID: 26965290.

256: Shariff NM, Safian N. Diabetes mellitus and its influence on sputum smear
positivity at the 2nd month of treatment among pulmonary tuberculosis patients in
Kuala Lumpur, Malaysia: A case control study. Int J Mycobacteriol. 2015
Dec;4(4):323-9. doi: 10.1016/j.ijmyco.2015.09.003. Epub 2015 Oct 1. PubMed PMID:
26964816.

257: Calverley PM, Postma DS, Anzueto AR, Make BJ, Eriksson G, Peterson S,
Jenkins CR. Early response to inhaled bronchodilators and corticosteroids as a
predictor of 12-month treatment responder status and COPD exacerbations. Int J
Chron Obstruct Pulmon Dis. 2016 Feb 25;11:381-90. doi: 10.2147/COPD.S93303.
eCollection 2016. PubMed PMID: 26952309; PubMed Central PMCID: PMC4772946.

258: Sun Q, Zhang Q, Gu J, Sun WW, Wang P, Bai C, Xiao HP, Sha W. Prevalence,
risk factors, management, and treatment outcomes of first-line antituberculous
drug-induced liver injury: a prospective cohort study. Pharmacoepidemiol Drug
Saf. 2016 Aug;25(8):908-17. doi: 10.1002/pds.3988. Epub 2016 Mar 2. PubMed PMID:

26935778.

259: Mukerji S, Shahpuri B, Clayton-Smith B, Smith N, Armstrong P, Hardy M,
Marchant G, Marsh E. Intravenous magnesium sulphate as an adjuvant therapy in
acute exacerbations of chronic obstructive pulmonary disease: a single centre,
randomised, double-blinded, parallel group, placebo-controlled trial: a pilot
study. N Z Med J. 2015 Nov 20;128(1425):34-42. PubMed PMID: 26905985.

260: Ruan Q, Liu Q, Sun F, Shao L, Jin J, Yu S, Ai J, Zhang B, Zhang W.
Moxifloxacin and gatifloxacin for initial therapy of tuberculosis: a
meta-analysis of randomized clinical trials. Emerg Microbes Infect. 2016 Feb
24;5:e12. doi: 10.1038/emi.2016.12. PubMed PMID: 26905025; PubMed Central PMCID:
PMC4777926.

261: Colombel JF, Sands BE, Rutgeerts P, Sandborn W, Danese S, D'Haens G,
Panaccione R, Loftus EV Jr, Sankoh S, Fox I, Parikh A, Milch C, Abhyankar B,
Feagan BG. The safety of vedolizumab for ulcerative colitis and Crohn's disease.
Gut. 2017 May;66(5):839-851. doi: 10.1136/gutjnl-2015-311079. Epub 2016 Feb 18.
PubMed PMID: 26893500; PubMed Central PMCID: PMC5531223.

262: Kang YA, Shim TS, Koh WJ, Lee SH, Lee CH, Choi JC, Lee JH, Jang SH, Yoo KH,
Jung KH, Kim KU, Choi SB, Ryu YJ, Kim KC, Um S, Kwon YS, Kim YH, Choi WI, Jeon K,
Hwang YI, Kim SJ, Lee HK, Heo E, Yim JJ. Choice between Levofloxacin and
Moxifloxacin and Multidrug-Resistant Tuberculosis Treatment Outcomes. Ann Am
Thorac Soc. 2016 Mar;13(3):364-70. doi: 10.1513/AnnalsATS.201510-690BC. PubMed
PMID: 26871879.

263: Phillips PP, Mendel CM, Burger DA, Crook AM, Nunn AJ, Dawson R, Diacon AH,
Gillespie SH. Limited role of culture conversion for decision-making in
individual patient care and for advancing novel regimens to confirmatory clinical

trials. BMC Med. 2016 Feb 4;14:19. doi: 10.1186/s12916-016-0565-y. Erratum in:
BMC Med. 2016;14:36. Crook, Angela [corrected to Crook, Angela M]. PubMed PMID:
26847437; PubMed Central PMCID: PMC4743210.

264: Okumura M, Sasaki Y, Yoshiyama T, Matsuda S, Osawa T, Morimoto K, Yanai H,
Kurasima A, Ogata H, Gotoh H. [CLINICAL EFFECTS OF TREATMENT FOR MYCOBACTERIUM
TUBERCULOSIS INFECTION IN PATIENTS AT A SPECIALIZED HOSPITAL IN 2011]. Kekkaku.
2015 Oct;90(10):665-70. Japanese. PubMed PMID: 26821395.

265: Kadota T, Matsui H, Hirose T, Suzuki J, Saito M, Akaba T, Kobayashi K,
Akashi S, Kawashima M, Tamura A, Nagai H, Akagawa S, Kobayashi N, Ohta K.
Analysis of drug treatment outcome in clarithromycin-resistant Mycobacterium
avium complex lung disease. BMC Infect Dis. 2016 Jan 27;16:31. doi:
10.1186/s12879-016-1384-7. PubMed PMID: 26818764; PubMed Central PMCID:
PMC4730784.

266: Chen KY, Chuang KJ, Liu HC, Lee KY, Feng PH, Su CL, Lin CL, Lee CN, Chuang
HC. Particulate matter is associated with sputum culture conversion in patients
with culture-positive tuberculosis. Ther Clin Risk Manag. 2016 Jan 6;12:41-6.
doi: 10.2147/TCRM.S92927. eCollection 2016. PubMed PMID: 26792994; PubMed Central
PMCID: PMC4708199.

267: Biraro IA, Kimuda S, Egesa M, Cose S, Webb EL, Joloba M, Smith SG, Elliott
AM, Dockrell HM, Katamba A. The Use of Interferon Gamma Inducible Protein 10 as a
Potential Biomarker in the Diagnosis of Latent Tuberculosis Infection in Uganda.
PLoS One. 2016 Jan 15;11(1):e0146098. doi: 10.1371/journal.pone.0146098.
eCollection 2016. PubMed PMID: 26771653; PubMed Central PMCID: PMC4714877.

268: Pastells C, Pascual N, Sanchez-Baeza F, Marco MP. Immunochemical
Determination of Pyocyanin and 1-Hydroxyphenazine as Potential Biomarkers of

Pseudomonas aeruginosa Infections. Anal Chem. 2016 Feb 2;88(3):1631-8. doi:
10.1021/acs.analchem.5b03490. Epub 2016 Jan 20. PubMed PMID: 26738983.

269: CarriÃ³n-Torres O, Cazorla-Saravia P, Torres Sales JW, Yhuri Carreazo N, De
La Cruz Armijo FE. [Characteristics of the diagnosis and treatment of pulmonary
tuberculosis in patients with and without diabetes mellitus type 2]. Rev Peru Med
Exp Salud Publica. 2015 Oct;32(4):680-6. Spanish. PubMed PMID: 26732915.

270: Azarkar Z, Sharifzadeh G, Ebrahimzadeh A, Olumi S. Time to Sputum Smear
Conversion in Smear-Positive Pulmonary Tuberculosis Patients and Factors for
Delayed Conversion. Iran J Med Sci. 2016 Jan;41(1):44-7. PubMed PMID: 26722144;
PubMed Central PMCID: PMC4691269.

271: Yuen CM, Kurbatova EV, Tupasi T, Caoili JC, Van Der Walt M, Kvasnovsky C,
Yagui M, Bayona J, Contreras C, Leimane V, Ershova J, Via LE, Kim H, Akksilp S,
Kazennyy BY, Volchenkov GV, Jou R, Kliiman K, Demikhova OV, Vasilyeva IA, Dalton
T, Cegielski JP. Association between Regimen Composition and Treatment Response
in Patients with Multidrug-Resistant Tuberculosis: A Prospective Cohort Study.
PLoS Med. 2015 Dec 29;12(12):e1001932. doi: 10.1371/journal.pmed.1001932.
eCollection 2015 Dec. PubMed PMID: 26714320; PubMed Central PMCID: PMC4700973.

272: Karoli R, Fatima J, Gupta SS, Shukla V, Moidurrehman, Manhar M. Vitamin D
Deficiency in Medical Patients at a Teaching Hospital in North India. J Assoc
Physicians India. 2015 Jun;63(6):35-9. PubMed PMID: 26710398.

273: Gadallah MA, Mokhtar A, Rady M, El-Moghazy E, Fawzy M, Kandil SK. Prognostic
factors of treatment among patients with multidrug-resistant tuberculosis in
Egypt. J Formos Med Assoc. 2016 Nov;115(11):997-1003. doi:
10.1016/j.jfma.2015.10.002. Epub 2015 Dec 13. PubMed PMID: 26696497.

274: Kim J, Kwak N, Lee HY, Kim TS, Kim CK, Han SK, Yim JJ. Effect of drug
resistance on negative conversion of sputum culture in patients with pulmonary
tuberculosis. Int J Infect Dis. 2016 Jan;42:64-68. doi:
10.1016/j.ijid.2015.11.018. Epub 2015 Dec 10. PubMed PMID: 26692454.

275: Li D, Wang T, Shen S, Cheng S, Yu J, Zhang Y, Zhang C, Tang H. Effects of
Fluroquinolones in Newly Diagnosed, Sputum-Positive Tuberculosis Therapy: A
Systematic Review and Network Meta-Analysis. PLoS One. 2015 Dec
15;10(12):e0145066. doi: 10.1371/journal.pone.0145066. eCollection 2015. Review.
PubMed PMID: 26669635; PubMed Central PMCID: PMC4682926.

276: Rieu R, Chang C, Collin SM, Fazekas J, Dassanaike S, Abbara A, Davidson RN.
Time to detection in liquid culture of sputum in pulmonary MDR-TB does not
predict culture conversion for early discharge. J Antimicrob Chemother. 2016
Mar;71(3):803-6. doi: 10.1093/jac/dkv407. Epub 2015 Dec 11. PubMed PMID:
26661394.

277: Masuda M, Sato T, Sakamaki K, Kudo M, Kaneko T, Ishigatsubo Y. The
effectiveness of sputum pH analysis in the prediction of response to therapy in
patients with pulmonary tuberculosis. PeerJ. 2015 Nov 26;3:e1448. doi:
10.7717/peerj.1448. eCollection 2015. PubMed PMID: 26644982; PubMed Central
PMCID: PMC4671190.

278: Si ZL, Kang LL, Shen XB, Zhou YZ. Adjuvant Efficacy of Nutrition Support
During Pulmonary Tuberculosis Treating Course: Systematic Review and
Meta-analysis. Chin Med J (Engl). 2015 Dec 5;128(23):3219-30. doi:
10.4103/0366-6999.170255. Review. PubMed PMID: 26612299; PubMed Central PMCID:
PMC4794866.

279: Zhu Y, Jia H, Chen J, Cui G, Gao H, Wei Y, Lu C, Wang L, Uede T, Diao H.
Decreased Osteopontin Expression as a Reliable Prognostic Indicator of
Improvement in Pulmonary Tuberculosis: Impact of the Level of
Interferon-gamma-Inducible Protein 10. Cell Physiol Biochem.
2015;37(5):1983-96.

doi: 10.1159/000438559. Epub 2015 Nov 20. PubMed PMID: 26584297.

280: Kanda R, Nagao T, Tho NV, Ogawa E, Murakami Y, Osawa M, Saika Y, Doi K,
Nakano Y. Factors Affecting Time to Sputum Culture Conversion in Adults with
Pulmonary Tuberculosis: A Historical Cohort Study without Censored Cases. PLoS
One. 2015 Nov 11;10(11):e0142607. doi: 10.1371/journal.pone.0142607. eCollection
2015. PubMed PMID: 26558764; PubMed Central PMCID: PMC4641703.

281: Miyazaki Y, Azuma A, Inase N, Taniguchi H, Ogura T, Inoue E, Takeuchi M,
Yoshizawa Y, Sugiyama Y, Kudoh S; IPF trial group in Japan. Cyclosporine A
combined with low-dose corticosteroid treatment in patients with idiopathic
pulmonary fibrosis. Respir Investig. 2015 Nov;53(6):288-95. doi:
10.1016/j.resinv.2015.05.002. Epub 2015 Jul 14. PubMed PMID: 26521106.

282: Min J, Park J, Lee YJ, Kim SJ, Park JS, Cho YJ, Yoon HI, Lee CT, Lee JH.
Determinants of recurrence after successful treatment of Mycobacterium avium
complex lung disease. Int J Tuberc Lung Dis. 2015 Oct;19(10):1239-45. doi:
10.5588/ijtld.14.0139. PubMed PMID: 26459540.

283: Kim YW, Seong MW, Kim TS, Yoo CG, Kim YW, Han SK, Yim JJ. Evaluation of
Xpert(Â®) MTB/RIF assay: diagnosis and treatment outcomes in rifampicin-resistant
tuberculosis. Int J Tuberc Lung Dis. 2015 Oct;19(10):1216-21. doi:
10.5588/ijtld.15.0183. PubMed PMID: 26459536.

284: Wallis RS, Peppard T. Early Biomarkers and Regulatory Innovation in
Multidrug-Resistant Tuberculosis. Clin Infect Dis. 2015 Oct 15;61Suppl 3:S160-3.
doi: 10.1093/cid/civ612. PubMed PMID: 26409278.

285: Mahadeo R, Gounder S, Graham SM. Changing from single-drug to fixed-dose
combinations: experience from Fiji. Public Health Action. 2014 Sep
21;4(3):169-73. doi: 10.5588/pha.14.0024. PubMed PMID: 26400805; PubMed Central
PMCID: PMC4533822.

286: Prasad P, Gounder S, Varman S, Viney K. Sputum smear conversion and
treatment outcomes for tuberculosis patients with and without diabetes in Fiji.
Public Health Action. 2014 Sep 21;4(3):159-63. doi: 10.5588/pha.14.0023. PubMed
PMID: 26400803; PubMed Central PMCID: PMC4533811.

287: Tukvadze N, Sanikidze E, Kipiani M, Hebbar G, Easley KA, Shenvi N, Kempker
RR, Frediani JK, Mirtskhulava V, Alvarez JA, Lomtadze N, Vashakidze L, Hao L, Del
Rio C, Tangpricha V, Blumberg HM, Ziegler TR. High-dose vitamin D3 in adults with
pulmonary tuberculosis: a double-blind randomized controlled trial. Am J Clin
Nutr. 2015 Nov;102(5):1059-69. doi: 10.3945/ajcn.115.113886. Epub 2015 Sep 23.
PubMed PMID: 26399865; PubMed Central PMCID: PMC4625591.

288: Jones-LÃ³pez EC, White LF, Kirenga B, Mumbowa F, Ssebidandi M, Moine S,
Mbabazi O, Mboowa G, Ayakaka I, Kim S, Thornton CS, Okwera A, Joloba M, Fennelly
KP. Cough Aerosol Cultures of Mycobacterium tuberculosis: Insights on TST / IGRA
Discordance and Transmission Dynamics. PLoS One. 2015 Sep 22;10(9):e0138358. doi:
10.1371/journal.pone.0138358. eCollection 2015. PubMed PMID: 26394149; PubMed
Central PMCID: PMC4578948.

289: Mily A, Rekha RS, Kamal SM, Arifuzzaman AS, Rahim Z, Khan L, Haq MA, Zaman
K, Bergman P, Brighenti S, Gudmundsson GH, Agerberth B, Raqib R. Significant
Effects of Oral Phenylbutyrate and Vitamin D3 Adjunctive Therapy in Pulmonary
Tuberculosis: A Randomized Controlled Trial. PLoS One. 2015 Sep
22;10(9):e0138340. doi: 10.1371/journal.pone.0138340. eCollection 2015. PubMed
PMID: 26394045; PubMed Central PMCID: PMC4578887.

290: Sawadogo B, Tint KS, Tshimanga M, Kuonza L, Ouedraogo L. Risk factors for
tuberculosis treatment failure among pulmonary tuberculosis patients in four
health regions of Burkina Faso, 2009: case control study. Pan Afr Med J. 2015 Jun
24;21:152. doi: 10.11604/pamj.2015.21.152.4827. eCollection 2015. PubMed PMID:

26327989; PubMed Central PMCID: PMC4546781.

291: Mahishale V, Patil B, Lolly M, Eti A, Khan S. Prevalence of Smoking and Its
Impact on Treatment Outcomes in Newly Diagnosed Pulmonary Tuberculosis Patients:
A Hospital-Based Prospective Study. Chonnam Med J. 2015 Aug;51(2):86-90. doi:
10.4068/cmj.2015.51.2.86. Epub 2015 Aug 17. PubMed PMID: 26306303; PubMed Central
PMCID: PMC4543154.

292: Shi G, Zhang L. Effects on type 2 diabetes complicated with pulmonary
tuberculosis: regiment of insulin, isoniazid, rifampicin, pyrazinamide and
ethambutol versus the regiment plus Qi-boosting and Yin-nourishing decoction of
Traditional Chinese Medicine. J Tradit Chin Med. 2015 Jun;35(3):260-5. PubMed
PMID: 26237828.

293: Nebenzahl-Guimaraes H, Verhagen LM, Borgdorff MW, van Soolingen D.
Transmission and Progression to Disease of Mycobacterium tuberculosis
Phylogenetic Lineages in The Netherlands. J Clin Microbiol. 2015
Oct;53(10):3264-71. doi: 10.1128/JCM.01370-15. Epub 2015 Jul 29. PubMed PMID:
26224845; PubMed Central PMCID: PMC4572527.

294: Junaid K, Rehman A, Saeed T, Jolliffe DA, Wood K, Martineau AR.
Genotype-independent association between profound vitamin D deficiency and
delayed sputum smear conversion in pulmonary tuberculosis. BMC Infect Dis. 2015
Jul 21;15:275. doi: 10.1186/s12879-015-1018-5. PubMed PMID: 26193879; PubMed
Central PMCID: PMC4508807.

295: Lin ZW, Gu J, Xu ST, Ge D, Wang Q. Video-Assisted Thoracoscopic Surgery for
Intralobar Pulmonary Sequestration: Wedge Resection Is Feasible in Limited
Peripheral Lesions. Thorac Cardiovasc Surg. 2016 Aug;64(5):456-60. doi:
10.1055/s-0035-1556820. Epub 2015 Jul 15. PubMed PMID: 26177227.

296: Shen H, Min R, Tan Q, Xie W, Wang H, Pan H, Zhang L, Xu H, Zhang X, Dai J.

The beneficial effects of adjunctive recombinant human interleukin-2 for
multidrug resistant tuberculosis. Arch Med Sci. 2015 Jun 19;11(3):584-90. doi:
10.5114/aoms.2015.52362. PubMed PMID: 26170852; PubMed Central PMCID: PMC4495154.

297: Jung M, Uhl B, Kristiansen G, Dietrich D. Bisulfite Conversion of DNA from
Tissues, Cell Lines, Buffy Coat, FFPE Tissues, Microdissected Cells, Swabs,
Sputum, Aspirates, Lavages, Effusions, Plasma, Serum, and Urine. Methods Mol
Biol. 2017;1589:139-159. doi: 10.1007/7651_2015_260. PubMed PMID: 26138988.

298: Field SK. Bedaquiline for the treatment of multidrug-resistant tuberculosis:
great promise or disappointment? Ther Adv Chronic Dis. 2015 Jul;6(4):170-84. doi:
10.1177/2040622315582325. Review. PubMed PMID: 26137207; PubMed Central PMCID:
PMC4480545.

299: Feng G, Wang D, Chen LI, Xie G, Zhang YU, Wang J, DU XB. Malignant
conversion of a solitary squamous cell papilloma in the trachea treated by
radiotherapy: A case report. Oncol Lett. 2015 May;9(5):2013-2016. Epub 2015 Mar
20. PubMed PMID: 26137004; PubMed Central PMCID: PMC4467276.

300: Ernawati, Gumilar E, Kuntoro, Soeroso J, Dekker G. Expectant management of
preterm preeclampsia in Indonesia and the role of steroids. J Matern Fetal
Neonatal Med. 2016;29(11):1736-40. doi: 10.3109/14767058.2015.1059815. Epub 2015
Jul 27. PubMed PMID: 26135754.

301: Rohini K, Bhat S, Srikumar PS, Mahesh Kumar A. Serum PCT and its Relation to
Body Weight Gain in Pulmonary Tuberculosis. Indian J Clin Biochem. 2015
Jul;30(3):329-33. doi: 10.1007/s12291-014-0432-6. Epub 2014 Apr 18. PubMed PMID:
26089621; PubMed Central PMCID: PMC4469052.

302: Lee SH, Seo KA, Lee YM, Lee HK, Kim JH, Shin C, Ghim JR, Shin JG, Kim DH.

Low Serum Concentrations of Moxifloxacin, Prothionamide, and Cycloserine on
Sputum Conversion in Multi-Drug Resistant TB. Yonsei Med J. 2015 Jul;56(4):961-7.
doi: 10.3349/ymj.2015.56.4.961. PubMed PMID: 26069117; PubMed Central PMCID:
PMC4479863.

303: Griffith DE, Adjemian J, Brown-Elliott BA, Philley JV, Prevots DR, Gaston C,
Olivier KN, Wallace RJ Jr. Semiquantitative Culture Analysis during Therapy for
Mycobacterium avium Complex Lung Disease. Am J Respir Crit Care Med. 2015 Sep
15;192(6):754-60. doi: 10.1164/rccm.201503-0444OC. PubMed PMID: 26068042; PubMed
Central PMCID: PMC4595680.

304: Wallis RS. Sputum culture conversion in new TB regimens. Lancet Respir Med.
2015 Jun;3(6):e18-9. doi: 10.1016/S2213-2600(15)00182-4. PubMed PMID: 26065975.

305: Dawson R, Narunsky K, Carman D, Gupte N, Whitelaw A, Efron A, Barnes GL,
Hoffman J, Chaisson RE, McIlleron H, Dorman SE. Two-stage activity-safety study
of daily rifapentine during intensive phase treatment of pulmonary tuberculosis.
Int J Tuberc Lung Dis. 2015 Jul;19(7):780-6. doi: 10.5588/ijtld.14.0868. PubMed
PMID: 26056101.

306: Mzinza DT, Sloan DJ, Jambo KC, Shani D, Kamdolozi M, Wilkinson KA, Wilkinson
RJ, Davies GR, Heyderman RS, Mwandumba HC. Kinetics of Mycobacterium
tuberculosis-specific IFN-Î³ responses and sputum bacillary clearance in
HIV-infected adults during treatment of pulmonary tuberculosis. Tuberculosis
(Edinb). 2015 Jul;95(4):463-9. doi: 10.1016/j.tube.2015.05.009. Epub 2015 May 28.
PubMed PMID: 26051653; PubMed Central PMCID: PMC4503815.

307: Palmero D, GonzÃ¡lez Montaner P, CufrÃ© M, GarcÃa A, Vescovo M, Poggi S. First
series of patients with XDR and pre-XDR TB treated with regimens that included
meropenen-clavulanate in Argentina. Arch Bronconeumol. 2015 Oct;51(10):e49-52.

doi: 10.1016/j.arbres.2015.03.012. Epub 2015 May 27. English, Spanish. PubMed
PMID: 26026689.

308: RamÃ-rez-Lapausa M, Pascual Pareja JF, Carrillo GÃ³mez R, MartÃ-nez-Prieto M,
GonzÃ¡lez-Ruano PÃ©rez P, Noguerado Asensio A. Retrospective study of tolerability
and efficacy of linezolid in patients with multidrug-resistant tuberculosis
(1998-2014). Enferm Infecc Microbiol Clin. 2016 Feb;34(2):85-90. doi:
10.1016/j.eimc.2015.04.003. Epub 2015 May 23. PubMed PMID: 26015067.

309: Mthiyane T, Rustomjee R, Pym A, Connolly C, Onyebujoh P, Theron G, Dheda K.
Impact of tuberculosis treatment and antiretroviral therapy on serial
RD-1-specific quantitative T-cell readouts (QuantiFERON-TB Gold In-Tube), and
relationship to treatment-related outcomes and bacterial burden. Int J Infect
Dis. 2015 Jul;36:46-53. doi: 10.1016/j.ijid.2015.05.006. Epub 2015 May 21. PubMed
PMID: 26003404.

310: Mohr E, Cox V, Wilkinson L, Moyo S, Hughes J, Daniels J, Muller O, Cox H.
Programmatic treatment outcomes in HIV-infected and uninfected drug-resistant TB
patients in Khayelitsha, South Africa. Trans R Soc Trop Med Hyg. 2015
Jul;109(7):425-32. doi: 10.1093/trstmh/trv037. Epub 2015 May 15. PubMed PMID:
25979526.

311: Amogne W, Aderaye G, Habtewold A, Yimer G, Makonnen E, Worku A, Sonnerborg
A, Aklillu E, Lindquist L. Efficacy and Safety of Antiretroviral Therapy
Initiated One Week after Tuberculosis Therapy in Patients with CD4 Counts < 200
Cells/Î¼L: TB-HAART Study, a Randomized Clinical Trial. PLoS One. 2015 May
12;10(5):e0122587. doi: 10.1371/journal.pone.0122587. eCollection 2015. PubMed
PMID: 25966339; PubMed Central PMCID: PMC4429073.

312: Dobler CC, Korver S, Batbayar O, Oyuntsetseg S, Tsolmon B, Wright C, Solongo

B, Marais BJ. Success of community-based directly observed anti-tuberculosis
treatment in Mongolia. Int J Tuberc Lung Dis. 2015 Jun;19(6):657-62. doi:
10.5588/ijtld.14.0927. PubMed PMID: 25946355.

313: Pietersen E, Peter J, Streicher E, Sirgel F, Rockwood N, Mastrapa B, Te
Riele J, Davids M, van Helden P, Warren R, Dheda K. High frequency of resistance,
lack of clinical benefit, and poor outcomes in capreomycin treated South African
patients with extensively drug-resistant tuberculosis. PLoS One. 2015 Apr
24;10(4):e0123655. doi: 10.1371/journal.pone.0123655. eCollection 2015. PubMed
PMID: 25909847; PubMed Central PMCID: PMC4409172.

314: Liang S, Zhang J, Hu L, Chen J, Wu J, Huang Y, Zeng Y, Zhu Y, Li Z, Wen Y,
Liang W, Zhuo J, He H. USA's expanded overseas tuberculosis screening program: a
retrospective study in China. BMC Public Health. 2015 Mar 7;15:231. doi:
10.1186/s12889-015-1558-z. PubMed PMID: 25886508; PubMed Central PMCID:
PMC4364631.

315: Djouma FN, Noubom M, Ateudjieu J, Donfack H. Delay in sputum smear
conversion and outcomes of smear-positive tuberculosis patients: a retrospective
cohort study in Bafoussam, Cameroon. BMC Infect Dis. 2015 Mar 21;15:139. doi:
10.1186/s12879-015-0876-1. PubMed PMID: 25884844; PubMed Central PMCID:
PMC4381415.

316: Cates J, Trieu L, Proops D, Ahuja SD. Contact Investigations Around
Mycobacterium tuberculosis Patients Without Positive Respiratory Culture. J
Public Health Manag Pract. 2016 May-Jun;22(3):275-82. doi:
10.1097/PHH.0000000000000261. PubMed PMID: 25867495.

317: Daley P, Jagannathan V, John KR, Sarojini J, Latha A, Vieth R, Suzana S,
Jeyaseelan L, Christopher DJ, Smieja M, Mathai D. Adjunctive vitamin D for
treatment of active tuberculosis in India: a randomised, double-blind,

placebo-controlled trial. Lancet Infect Dis. 2015 May;15(5):528-34. doi:
10.1016/S1473-3099(15)70053-8. Epub 2015 Apr 8. PubMed PMID: 25863562.

318: Wang M, Guan X, Chi Y, Robinson N, Liu JP. Chinese herbal medicine as
adjuvant treatment to chemotherapy for multidrug-resistant tuberculosis (MDR-TB):
A systematic review of randomised clinical trials. Tuberculosis (Edinb). 2015
Jul;95(4):364-72. doi: 10.1016/j.tube.2015.03.003. Epub 2015 Mar 18. Review.
PubMed PMID: 25861717.

319: Kibleur Y, Veziris N. French Nationwide Cohort Temporary Utilization
Authorization Survey of GranuPAS(Â®) in MDR-TB patients. Chemotherapy.
2014;60(3):174-9. doi: 10.1159/000371869. Epub 2015 Mar 23. PubMed PMID:
25823752.

320: Adekambi T, Ibegbu CC, Cagle S, Kalokhe AS, Wang YF, Hu Y, Day CL, Ray SM,
Rengarajan J. Biomarkers on patient T cells diagnose active tuberculosis and
monitor treatment response. J Clin Invest. 2015 May;125(5):1827-38. doi:
10.1172/JCI77990. Epub 2015 Mar 30. Erratum in: J Clin Invest. 2015
Sep;125(9):3723. PubMed PMID: 25822019; PubMed Central PMCID: PMC4598074.

321: Ndusilo ND, Heysell SK, Mpagama SG, Gratz J, Segesela FH, Pazia SJ, Wang XQ,
Houpt ER, Kibiki GS. Improvement in plasma drug activity during the early
treatment interval among Tanzanian patients with multidrug-resistant
tuberculosis. PLoS One. 2015 Mar 27;10(3):e0122769. doi:
10.1371/journal.pone.0122769. eCollection 2015. PubMed PMID: 25816161; PubMed
Central PMCID: PMC4376785.

322: Shahraki AH, Heidarieh P, Bostanabad SZ, Khosravi AD, Hashemzadeh M, Khandan
S, Biranvand M, Schraufnagel DE, Mirsaeidi M. "Multidrug-resistant tuberculosis"
may be nontuberculous mycobacteria. Eur J Intern Med. 2015 May;26(4):279-84. doi:
10.1016/j.ejim.2015.03.001. Epub 2015 Mar 14. PubMed PMID: 25784643; PubMed

Central PMCID: PMC4414892.

323: Wang Q, Zhang C, Guo J, Huang J, Xi X, Zhang L, Cui X. Super-compact
treatment with a high dose of moxifloxacin in patients with drug-resistant
tuberculosis and its resistance mechanisms. Exp Ther Med. 2015
Apr;9(4):1314-1318. Epub 2015 Jan 29. PubMed PMID: 25780428; PubMed Central
PMCID: PMC4353789.

324: Kim W, Lee KS, Kim HS, Koh WJ, Jeong BH, Chung MJ, Jang HW. CT and
microbiologic follow-up in primary multidrug-resistant pulmonary tuberculosis.
Acta Radiol. 2016 Feb;57(2):197-204. doi: 10.1177/0284185115575196. Epub 2015 Mar
9. PubMed PMID: 25759483.

325: Frediani JK, Sanikidze E, Kipiani M, Tukvadze N, Hebbar G, Ramakrishnan U,
Jones DP, Easley KA, Shenvi N, Kempker RR, Tangpricha V, Blumberg HM, Ziegler TR.
Macronutrient intake and body composition changes during anti-tuberculosis
therapy in adults. Clin Nutr. 2016 Feb;35(1):205-12. doi:
10.1016/j.clnu.2015.02.007. Epub 2015 Feb 26. PubMed PMID: 25753551; PubMed
Central PMCID: PMC4550569.

326: Selvaraj P, Harishankar M, Afsal K. Vitamin D: Immuno-modulation and
tuberculosis treatment. Can J Physiol Pharmacol. 2015 May;93(5):377-84. doi:
10.1139/cjpp-2014-0386. Epub 2015 Jan 19. Review. PubMed PMID: 25744368.

327: Wallis RS. Corticosteroid effects on sputum culture in pulmonary
tuberculosis: a meta-regression analysis. Open Forum Infect Dis. 2014 Jun
3;1(1):ofu020. doi: 10.1093/ofid/ofu020. eCollection 2014 Mar. PubMed PMID:
25734093; PubMed Central PMCID: PMC4324181.

328: Farazi A, Shafaat O, Sofian M, Kahbazi M. Arginine adjunctive therapy in
active tuberculosis. Tuberc Res Treat. 2015;2015:205016. doi:
10.1155/2015/205016. Epub 2015 Feb 3. PubMed PMID: 25734013; PubMed Central

PMCID: PMC4334935.

329: Wallis RS. Sputum culture conversion as a tuberculosis biomarker: a glass
half empty or half full? Lancet Respir Med. 2015 Mar;3(3):174-5. doi:
10.1016/S2213-2600(15)00058-2. Epub 2015 Feb 26. PubMed PMID: 25726087.

330: Kurbatova EV, Cegielski JP, Lienhardt C, Akksilp R, Bayona J, Becerra MC,
Caoili J, Contreras C, Dalton T, Danilovits M, Demikhova OV, Ershova J, Gammino
VM, Gelmanova I, Heilig CM, Jou R, Kazennyy B, Keshavjee S, Kim HJ, Kliiman K,
Kvasnovsky C, Leimane V, Mitnick CD, Quelapio I, Riekstina V, Smith SE, Tupasi T,
van der Walt M, Vasilyeva IA, Via LE, Viiklepp P, Volchenkov G, Walker AT,
Wolfgang M, Yagui M, Zignol M. Sputum culture conversion as a prognostic marker
for end-of-treatment outcome in patients with multidrug-resistant tuberculosis: a
secondary analysis of data from two observational cohort studies. Lancet Respir
Med. 2015 Mar;3(3):201-9. doi: 10.1016/S2213-2600(15)00036-3. Epub 2015 Feb 26.
PubMed PMID: 25726085; PubMed Central PMCID: PMC4401426.

331: Mroz RM, Lisowski P, Tycinska A, Bierla J, Trzeciak PZ, Minarowski L,
Milewski R, Lisowska A, Boros P, Sobkowicz B, Duszewska AM, Chyczewska E, Musial
WJ, MacNee W. Anti-inflammatory effects of atorvastatin treatment in chronic
obstructive pulmonary disease. A controlled pilot study. J Physiol Pharmacol.
2015 Feb;66(1):111-28. PubMed PMID: 25716971.

332: Kerstjens HA, Casale TB, Bleecker ER, Meltzer EO, Pizzichini E, Schmidt O,
Engel M, Bour L, Verkleij CB, Moroni-Zentgraf P, Bateman ED. Tiotropium or
salmeterol as add-on therapy to inhaled corticosteroids for patients with
moderate symptomatic asthma: two replicate, double-blind, placebo-controlled,
parallel-group, active-comparator, randomised trials. Lancet Respir Med. 2015
May;3(5):367-76. doi: 10.1016/S2213-2600(15)00031-4. Epub 2015 Feb 12. PubMed
PMID: 25682232.

333: Szumowski JD, Lynch JB. Profile of delamanid for the treatment of
multidrug-resistant tuberculosis. Drug Des Devel Ther. 2015 Jan 29;9:677-82. doi:
10.2147/DDDT.S60923. eCollection 2015. Review. PubMed PMID: 25678771; PubMed
Central PMCID: PMC4319680.

334: Behnaz F, Mohammadzadeh M, Mohammadzade G. Five-year assessment of time of
sputum smears conversion and outcome and risk factors of tuberculosis patients in
central iran. Tuberc Res Treat. 2015;2015:609083. doi: 10.1155/2015/609083. Epub
2015 Jan 14. PubMed PMID: 25653874; PubMed Central PMCID: PMC4310312.

335: Diacon AH, Dawson R, von Groote-Bidlingmaier F, Symons G, Venter A, Donald
PR, van Niekerk C, Everitt D, Hutchings J, Burger DA, Schall R, Mendel CM.
Bactericidal activity of pyrazinamide and clofazimine alone and in combinations
with pretomanid and bedaquiline. Am J Respir Crit Care Med. 2015 Apr
15;191(8):943-53. doi: 10.1164/rccm.201410-1801OC. PubMed PMID: 25622149.

336: Tan S, Ding X, Tan Y, Cai X, Li Y. [The therapeutic effect of regimens
containing isoniazid and rifampicin for pulmonary tuberculosis with single
isoniazid or rifampicin resistance]. Zhonghua Jie He He Hu Xi Za Zhi. 2014
Dec;37(12):915-8. Chinese. PubMed PMID: 25609129.

337: Tang S, Yao L, Hao X, Liu Y, Zeng L, Liu G, Li M, Li F, Wu M, Zhu Y, Sun H,
Gu J, Wang X, Zhang Z. Clofazimine for the treatment of multidrug-resistant
tuberculosis: prospective, multicenter, randomized controlled study in China.
Clin Infect Dis. 2015 May 1;60(9):1361-7. doi: 10.1093/cid/civ027. Epub 2015 Jan
20. PubMed PMID: 25605283.

338: Mah A, Kharrat H, Ahmed R, Gao Z, Der E, Hansen E, Long R, Kunimoto D,
Cooper R. Serum drug concentrations of INH and RMP predict 2-month sputum culture

results in tuberculosis patients. Int J Tuberc Lung Dis. 2015
Feb;19(2):210-5.
doi: 10.5588/ijtld.14.0405. PubMed PMID: 25574921.

339: Volkmann T, Moonan PK, Miramontes R, Oeltmann JE. Tuberculosis
and excess
alcohol use in the United States, 1997-2012. Int J Tuberc Lung Dis.
2015
Jan;19(1):111-9. doi: 10.5588/ijtld.14.0516. PubMed PMID: 25519800;
PubMed
Central PMCID: PMC4798229.

340: Brode SK, Varadi R, McNamee J, Malek N, Stewart S, Jamieson FB,
Avendano M.
Multidrug-resistant tuberculosis: Treatment and outcomes of 93
patients. Can
Respir J. 2015 Mar-Apr;22(2):97-102. Epub 2014 Dec 10. PubMed PMID:
25493698;
PubMed Central PMCID: PMC4390019.

341: Khandelwal D, Gupta N, Mukherjee A, Lodha R, Singh V, Grewal
HM, Bhatnagar
S, Singh S, Kabra SK; Delhi Pediatric TB Study Group. Vitamin D
levels in Indian
children with intrathoracic tuberculosis. Indian J Med Res. 2014
Oct;140(4):531-7. PubMed PMID: 25488448; PubMed Central PMCID:
PMC4277140.

342: Jajarm HH, Falaki F, Sanatkhani M, Ahmadzadeh M, Ahrari F,
Shafaee H. A
comparative study of toluidine blue-mediated photodynamic therapy
versus topical
corticosteroids in the treatment of erosive-atrophic oral lichen
planus: a
randomized clinical controlled trial. Lasers Med Sci. 2015
Jul;30(5):1475-80.
doi: 10.1007/s10103-014-1694-1. Epub 2014 Dec 9. PubMed PMID:
25487185.

343: Haridas V, Pean P, Jasenosky LD, Madec Y, Laureillard D, Sok T,
Sath S,
Borand L, Marcy O, Chan S, Tsitsikov E, Delfraissy JF, Blanc FX,
Goldfeld AE;
CAPRI-T (ANRS 12164) Study Team. TB-IRIS, T-cell activation, and
remodeling of
the T-cell compartment in highly immunosuppressed HIV-infected
patients with TB.
AIDS. 2015 Jan 28;29(3):263-73. doi: 10.1097/QAD.0000000000000546.
PubMed PMID:
25486415; PubMed Central PMCID: PMC4649949.

344: Vasudevan K, Jayakumar N, Gnanasekaran D. Smear Conversion, Treatment
Outcomes and the Time of Default in Registered Tuberculosis Patients on RNTCP
DOTS in Puducherry, Southern India. J Clin Diagn Res. 2014 Oct;8(10):JC05-8. doi:
10.7860/JCDR/2014/8421.4984. Epub 2014 Oct 20. PubMed PMID: 25478371; PubMed
Central PMCID: PMC4253189.

345: Tung YJ, Bittaye SO, Tsai JR, Lin CY, Huang CH, Chen TC, Lin WR, Chang K,
Lai CC, Lu PL, Chen YH. Risk factors for microbiologic failure among Taiwanese
adults with Mycobacterium abscessus complex pulmonary disease. J Microbiol
Immunol Infect. 2015 Aug;48(4):437-45. doi: 10.1016/j.jmii.2014.08.005. Epub 2014
Oct 22. PubMed PMID: 25440976.

346: Venkat VL, Shneider BL, Magee JC, Turmelle Y, Arnon R, Bezerra JA, Hertel
PM, Karpen SJ, Kerkar N, Loomes KM, Molleston J, Murray KF, Ng VL, Raghunathan T,
Rosenthal P, Schwartz K, Sherker AH, Sokol RJ, Teckman J, Wang K, Whitington PF,
Heubi JE; Childhood Liver Disease Research and Education Network. Total serum
bilirubin predicts fat-soluble vitamin deficiency better than serum bile acids in
infants with biliary atresia. J Pediatr Gastroenterol Nutr. 2014 Dec;59(6):702-7.
doi: 10.1097/MPG.0000000000000547. PubMed PMID: 25419594; PubMed Central PMCID:
PMC4243585.

347: Blair HA, Scott LJ. Delamanid: a review of its use in patients with
multidrug-resistant tuberculosis. Drugs. 2015 Jan;75(1):91-100. doi:
10.1007/s40265-014-0331-4. Review. PubMed PMID: 25404020.

348: Jeong BH, Jeon K, Park HY, Kim SY, Lee KS, Huh HJ, Ki CS, Lee NY, Shin SJ,
Daley CL, Koh WJ. Intermittent antibiotic therapy for nodular bronchiectatic
Mycobacterium avium complex lung disease. Am J Respir Crit Care Med. 2015 Jan
1;191(1):96-103. doi: 10.1164/rccm.201408-1545OC. PubMed PMID: 25393520.

349: Critchley JA, Orton LC, Pearson F. Adjunctive steroid therapy for managing
pulmonary tuberculosis. Cochrane Database Syst Rev. 2014 Nov 12;(11):CD011370.
doi: 10.1002/14651858.CD011370. Review. PubMed PMID: 25387839.

350: Kim JH, Song WJ, Jun JE, Ryu DH, Lee JE, Jeong HJ, Jeong SH, Kang HK, Kim
JS, Lee H, Chon HR, Jeon K, Kim D, Kim J, Koh WJ. Mycobacterium abscessus Lung
Disease in a Patient with Kartagener Syndrome. Tuberc Respir Dis (Seoul). 2014
Sep;77(3):136-40. doi: 10.4046/trd.2014.77.3.136. Epub 2014 Sep 30. PubMed PMID:
25309609; PubMed Central PMCID: PMC4192312.

351: Kim SY, Koh WJ, Kim YH, Jeong BH, Park HY, Jeon K, Kim JS, Cho SN, Shin SJ.
Importance of reciprocal balance of T cell immunity in Mycobacterium abscessus
complex lung disease. PLoS One. 2014 Oct 8;9(10):e109941. doi:
10.1371/journal.pone.0109941. eCollection 2014. PubMed PMID: 25295870; PubMed
Central PMCID: PMC4190320.

352: Morino E, Yanagawa Y, Takasaki J, Shimbo T, Sugiyama H, Kobayashi N. [New
criteria enable shorter hospitalization of patients with continuously
smear-positive pulmonary tuberculosis]. Kekkaku. 2014 Aug;89(8):697-702.
Japanese. PubMed PMID: 25283010.

353: Andrade BB, Singh A, Narendran G, Schechter ME, Nayak K, Subramanian S,
Anbalagan S, Jensen SM, Porter BO, Antonelli LR, Wilkinson KA, Wilkinson RJ,
Meintjes G, van der Plas H, Follmann D, Barber DL, Swaminathan S, Sher A, Sereti
I. Mycobacterial antigen driven activation of CD14++CD16- monocytes is a
predictor of tuberculosis-associated immune reconstitution inflammatory syndrome.
PLoS Pathog. 2014 Oct 2;10(10):e1004433. doi: 10.1371/journal.ppat.1004433.
eCollection 2014 Oct. PubMed PMID: 25275318; PubMed Central PMCID: PMC4183698.

354: Arnedo-Pena A, Juan-CerdÃ¡n JV, Romeu-GarcÃa MA, GarcÃa-Ferrer D,

HolguÃ-n-GÃ³mez R, Iborra-Millet J, Pardo-Serrano F. Vitamin D status and incidence
of tuberculosis infection conversion in contacts of pulmonary tuberculosis
patients: a prospective cohort study. Epidemiol Infect. 2015 Jun;143(8):1731-41.
doi: 10.1017/S0950268814002386. Epub 2014 Oct 2. PubMed PMID: 25274036.

355: Ito Y, Hirai T, Fujita K, Kubo T, Maekawa K, Ichiyama S, Togashi K, Mishima
M. The influence of environmental exposure on the response to antimicrobial
treatment in pulmonary Mycobacterial avium complex disease. BMC Infect Dis. 2014
Sep 29;14:522. doi: 10.1186/1471-2334-14-522. PubMed PMID: 25266993; PubMed
Central PMCID: PMC4261885.

356: Ahsan MJ, Ansari MY, Yasmin S, Jadav SS, Kumar P, Garg SK, Aseri A,
Khalilullah H. Tuberculosis: current treatment, diagnostics, and newer
antitubercular agents in clinical trials. Infect Disord Drug Targets.
2015;15(1):32-41. Review. PubMed PMID: 25246035.

357: Tierney DB, Franke MF, Becerra MC, AlcÃ¡ntara VirÃº FA, Bonilla CA, SÃ¡nchez E,
Guerra D, MuÃ±oz M, Llaro K, Palacios E, Mestanza L, Hurtado RM, Furin JJ, Shin S,
Mitnick CD. Time to culture conversion and regimen composition in
multidrug-resistant tuberculosis treatment. PLoS One. 2014 Sep 19;9(9):e108035.
doi: 10.1371/journal.pone.0108035. eCollection 2014. PubMed PMID: 25238411;
PubMed Central PMCID: PMC4169600.

358: Hire R, Kale AS, Dakhale GN, Gaikwad N. A prospective, observational study
of adverse reactions to drug regimen for multi-drug resistant pulmonary
tuberculosis in central India. Mediterr J Hematol Infect Dis. 2014 Sep
1;6(1):e2014061. doi: 10.4084/MJHID.2014.061. eCollection 2014. PubMed PMID:
25237474; PubMed Central PMCID: PMC4165500.

359: Tang S, Yao L, Hao X, Zhang X, Liu G, Liu X, Wu M, Zen L, Sun H, Liu Y, Gu

J, Lin F, Wang X, Zhang Z. Efficacy, safety and tolerability of linezolid for the
treatment of XDR-TB: a study in China. Eur Respir J. 2015 Jan;45(1):161-70. doi:
10.1183/09031936.00035114. Epub 2014 Sep 18. PubMed PMID: 25234807.

360: Aung KJ, Van Deun A, Declercq E, Sarker MR, Das PK, Hossain MA, Rieder HL.
Successful '9-month Bangladesh regimen' for multidrug-resistant tuberculosis
among over 500 consecutive patients. Int J Tuberc Lung Dis. 2014
Oct;18(10):1180-7. doi: 10.5588/ijtld.14.0100. PubMed PMID: 25216831.

361: Nunn AJ, Rusen ID, Van Deun A, Torrea G, Phillips PP, Chiang CY, Squire SB,
Madan J, Meredith SK. Evaluation of a standardized treatment regimen of
anti-tuberculosis drugs for patients with multi-drug-resistant tuberculosis
(STREAM): study protocol for a randomized controlled trial. Trials. 2014 Sep
9;15:353. doi: 10.1186/1745-6215-15-353. PubMed PMID: 25199531; PubMed Central
PMCID: PMC4164715.

362: How SH, Kuan YC, Ng TH, Razali MR, Fauzi AR. Monitoring treatment response
in sputum smear positive pulmonary tuberculosis patients: comparison of weight
gain, sputum conversion and chest radiograph. Malays J Pathol. 2014
Aug;36(2):91-6. PubMed PMID: 25194531.

363: Pandie S, Engel ME, Kerbelker ZS, Mayosi BM. Mycobacterium w Immunotherapy
for Treating Pulmonary Tuberculosis - a Systematic Review. Curr Pharm Des.
2014;20(39):6207-14. Review. PubMed PMID: 25190062.

364: Grisaru-Soen G, Savyon M, Sadot E, Schechner V, Sivan Y, Schwartz D,
Tarabeia J, Amitai Z, Yoabov I, Carmeli Y. Congenital tuberculosis and management
of exposure in neonatal and pediatric intensive care units. Int J Tuberc Lung
Dis. 2014 Sep;18(9):1062-5. doi: 10.5588/ijtld.14.0160. PubMed PMID: 25189553.

365: Dharmadhikari AS, Mphahlele M, Venter K, Stoltz A, Mathebula R, Masotla T,

van der Walt M, Pagano M, Jensen P, Nardell E. Rapid impact of effective
treatment on transmission of multidrug-resistant tuberculosis. Int J Tuberc Lung
Dis. 2014 Sep;18(9):1019-25. doi: 10.5588/ijtld.13.0834. PubMed PMID: 25189547;
PubMed Central PMCID: PMC4692272.

366: Mayosi BM, Ntsekhe M, Bosch J, Pandie S, Jung H, Gumedze F, Pogue J, Thabane
L, Smieja M, Francis V, Joldersma L, Thomas KM, Thomas B, Awotedu AA, Magula NP,
Naidoo DP, Damasceno A, Chitsa Banda A, Brown B, Manga P, Kirenga B, Mondo C,
Mntla P, Tsitsi JM, Peters F, Essop MR, Russell JB, Hakim J, Matenga J, Barasa
AF, Sani MU, Olunuga T, Ogah O, Ansa V, Aje A, Danbauchi S, Ojji D, Yusuf S; IMPI
Trial Investigators. Prednisolone and Mycobacterium indicus pranii in tuberculous
pericarditis. N Engl J Med. 2014 Sep 18;371(12):1121-30. doi:
10.1056/NEJMoa1407380. Epub 2014 Sep 1. PubMed PMID: 25178809; PubMed Central
PMCID: PMC4912834.

367: Anyim MC, Oshi DC, Chukwu JN, Aguwa EN, Johnson IN, Nwafor C, Meka AO,
Ogbudebe C, Madichie NO, Ekeke N, Olanisebe SB. Sputum conversion at the end of
8weeks among category 1 tuberculosis patients: How reliable are the peripheral
laboratory results? Int J Mycobacteriol. 2014 Sep;3(3):178-83. doi:
10.1016/j.ijmyco.2014.06.005. Epub 2014 Jul 12. PubMed PMID: 26786486.

368: Olaru ID, Heyckendorf J, Grossmann S, Lange C. Time to culture positivity
and sputum smear microscopy during tuberculosis therapy. PLoS One. 2014 Aug
29;9(8):e106075. doi: 10.1371/journal.pone.0106075. eCollection 2014. PubMed
PMID: 25171337; PubMed Central PMCID: PMC4149502.

369: Diacon AH, Pym A, Grobusch MP, de los Rios JM, Gotuzzo E, Vasilyeva I,
Leimane V, Andries K, Bakare N, De Marez T, Haxaire-Theeuwes M, Lounis N,
Meyvisch P, De Paepe E, van Heeswijk RP, Dannemann B; TMC207-C208 Study Group.
Multidrug-resistant tuberculosis and culture conversion with bedaquiline. N Engl

J Med. 2014 Aug 21;371(8):723-32. doi: 10.1056/NEJMoa1313865. PubMed PMID:
25140958.

370: Lee HY, Chae KO, Lee CH, Choi SM, Lee J, Park YS, Lee SM, Yoo CG, Kim YW,
Han SK, Yim JJ. Culture conversion rate at 2 months of treatment according to
diagnostic methods among patients with culture-positive pulmonary tuberculosis.
PLoS One. 2014 Aug 8;9(8):e103768. doi: 10.1371/journal.pone.0103768. eCollection
2014. PubMed PMID: 25105410; PubMed Central PMCID: PMC4126681.

371: Enomoto Y, Hagiwara E, Komatsu S, Nishihira R, Baba T, Kitamura H, Sekine A,
Nakazawa A, Ogura T. Pilot quasi-randomized controlled study of herbal medicine
Hochuekkito as an adjunct to conventional treatment for progressed pulmonary
Mycobacterium avium complex disease. PLoS One. 2014 Aug 5;9(8):e104411. doi:
10.1371/journal.pone.0104411. eCollection 2014. PubMed PMID: 25093868; PubMed
Central PMCID: PMC4122490.

372: Gumbo T, Pasipanodya JG, Wash P, Burger A, McIlleron H. Redefining
multidrug-resistant tuberculosis based on clinical response to combination
therapy. Antimicrob Agents Chemother. 2014 Oct;58(10):6111-5. doi:
10.1128/AAC.03549-14. Epub 2014 Aug 4. PubMed PMID: 25092691; PubMed Central
PMCID: PMC4187940.

373: Kipiani M, Mirtskhulava V, Tukvadze N, Magee M, Blumberg HM, Kempker RR.
Significant clinical impact of a rapid molecular diagnostic test (Genotype
MTBDRplus assay) to detect multidrug-resistant tuberculosis. Clin Infect Dis.
2014 Dec 1;59(11):1559-66. doi: 10.1093/cid/ciu631. Epub 2014 Aug 4. PubMed PMID:
25091301; PubMed Central PMCID: PMC4357804.

374: Andrews B, Muchemwa L, Kelly P, Lakhi S, Heimburger DC, Bernard GR.
Simplified severe sepsis protocol: a randomized controlled trial of modified
early goal-directed therapy in Zambia. Crit Care Med. 2014 Nov;42(11):2315-24.

doi: 10.1097/CCM.0000000000000541. PubMed PMID: 25072757; PubMed Central PMCID:
PMC4199893.

375: Charles M, Vilbrun SC, Koenig SP, Hashiguchi LM, Mabou MM, Ocheretina O,
Pape JW. Treatment outcomes for patients with multidrug-resistant tuberculosis in
post-earthquake Port-au-Prince, Haiti. Am J Trop Med Hyg. 2014 Oct;91(4):715-21.
doi: 10.4269/ajtmh.14-0161. Epub 2014 Jul 28. PubMed PMID: 25071001; PubMed
Central PMCID: PMC4183393.

376: Nijenbandring de Boer R, Cobelens FG, Kritski AL. Response from authors to
letter from Eduardo HernÃ¡ndez-GarduÃ±o (TUBE 2013_75) Tuberculosis monoresistance
and culture conversion in smokers Nijenbandring de Boer R, et al., "Delayed
culture conversion due to cigarette smoking in active pulmonary tuberculosis
patients, Tuberculosis (2014) January". Tuberculosis (Edinb). 2014 Sep;94(5):532.
doi: 10.1016/j.tube.2014.06.003. Epub 2014 Jun 28. PubMed PMID: 25066945.

377: Riou C, Gray CM, Lugongolo M, Gwala T, Kiravu A, Deniso P, Stewart-Isherwood
L, Omar SV, Grobusch MP, Coetzee G, Conradie F, Ismail N, Kaplan G, Fallows D. A
subset of circulating blood mycobacteria-specific CD4 T cells can predict the
time to Mycobacterium tuberculosis sputum culture conversion. PLoS One. 2014 Jul
21;9(7):e102178. doi: 10.1371/journal.pone.0102178. eCollection 2014. PubMed
PMID: 25048802; PubMed Central PMCID: PMC4105550.

378: Mendham AE, Duffield R, Marino F, Coutts AJ. Small-sided games training
reduces CRP, IL-6 and leptin in sedentary, middle-aged men. Eur J Appl Physiol.
2014 Nov;114(11):2289-97. doi: 10.1007/s00421-014-2953-3. Epub 2014 Jul 22.
PubMed PMID: 25048075.

379: Moraes ML, Ramalho DM, Delogo KN, Miranda PF, Mesquita ED, Oliveira HM,
Ruffino-Netto A, Almeida PC, Hauser-Davis RA, Campos RC, Kritski AL, Oliveira MM.

Association between serum selenium level and conversion of bacteriological tests
during antituberculosis treatment. J Bras Pneumol. 2014 May-Jun;40(3):269-78.
English, Portuguese. PubMed PMID: 25029650; PubMed Central PMCID: PMC4109199.

380: Velayutham BV, Allaudeen IS, Sivaramakrishnan GN, Perumal V, Nair D,
Chinnaiyan P, Paramasivam PK, Dhanaraj B, Santhanakrishnan RK, Navaneethapandian
GP, Marimuthu MK, Kumar V, Kandasamy C, Dharuman K, Elangovan T, Narasimhan M,
Rathinam S, Vadivelu G, Rathinam P, Chockalingam C, Jayabal L, Swaminathan S,
Shaheed JM. Sputum culture conversion with moxifloxacin-containing regimens in
the treatment of patients with newly diagnosed sputum-positive pulmonary
tuberculosis in South India. Clin Infect Dis. 2014 Nov 15;59(10):e142-9. doi:
10.1093/cid/ciu550. Epub 2014 Jul 14. PubMed PMID: 25028463.

381: Sundy JS, Schumacher HR, Kivitz A, Weinstein SP, Wu R, King-Davis S, Evans
RR. Rilonacept for gout flare prevention in patients receiving uric acid-lowering
therapy: results of RESURGE, a phase III, international safety study. J
Rheumatol. 2014 Aug;41(8):1703-11. doi: 10.3899/jrheum.131226. Epub 2014 Jul 15.
PubMed PMID: 25028379.

382: Mooventhan A, Khode V, Nivethitha L. Effect of yogic breathing techniques in
new sputum positive pulmonary tuberculosis. Int J Prev Med. 2014 Jun;5(6):787-90.
PubMed PMID: 25013700; PubMed Central PMCID: PMC4085933.

383: HernÃ¡ndez-GarduÃ±o E. Tuberculosis monoresistance and culture conversion in
smokers. Tuberculosis (Edinb). 2014 Sep;94(5):531. doi:
10.1016/j.tube.2014.06.008. Epub 2014 Jun 27. PubMed PMID: 25012140.

384: Kigozi NG, Chikobvu P, Heunis JC, van der Merwe S. A Retrospective Analysis
of Two-Month Sputum Smear Non-Conversion in New Sputum Smear Positive
Tuberculosis Patients in the Free State Province, South Africa. J Public Health

Afr. 2014 Jul 9;5(2):324. doi: 10.4081/jphia.2014.324. eCollection 2014 Jun 29.
PubMed PMID: 28299125; PubMed Central PMCID: PMC5345412.

385: Moraes ML, Ramalho DM, Delogo KN, Miranda PF, Mesquita ED, de Melo Guedes de
Oliveira HM, Netto AR, Dos Anjos MJ, Kritski AL, de Oliveira MM. Association of
serum levels of iron, copper, and zinc, and inflammatory markers with
bacteriological sputum conversion during tuberculosis treatment. Biol Trace Elem
Res. 2014 Aug;160(2):176-84. doi: 10.1007/s12011-014-0046-0. Epub 2014 Jun 24.
PubMed PMID: 24958018.

386: Fanai S, Viney K, Tarivonda L, Roseveare C, Tagaro M, Marais BJ. Profile of
tuberculosis patients with delayed sputum smear conversion in the Pacific island
of Vanuatu. Public Health Action. 2014 Jun 21;4(Suppl 1):S19-24. doi:
10.5588/pha.13.0075. PubMed PMID: 26477281; PubMed Central PMCID: PMC4547598.

387: Muzzi A, Seminari E, Feletti T, Scudeller L, Marone P, Tinelli C, Minoli L,
Marena C, Mangiarotti P, Strosselli M. Post-exposure rate of tuberculosis
infection among health care workers measured with tuberculin skin test conversion
after unprotected exposure to patients with pulmonary tuberculosis: 6-year
experience in an Italian teaching hospital. BMC Infect Dis. 2014 Jun 12;14:324.
doi: 10.1186/1471-2334-14-324. PubMed PMID: 24919953; PubMed Central PMCID:
PMC4065580.

388: Bar-On O, Mussaffi H, Mei-Zahav M, Prais D, Steuer G, Stafler P, Hananya S,
Blau H. Increasing nontuberculous mycobacteria infection in cystic fibrosis. J
Cyst Fibros. 2015 Jan;14(1):53-62. doi: 10.1016/j.jcf.2014.05.008. Epub 2014 Jun
7. PubMed PMID: 24917112.

389: Putri FA, Burhan E, Nawas A, Soepandi PZ, Sutoyo DK, Agustin H, Isbaniah F,
Dowdy DW. Body mass index predictive of sputum culture conversion among MDR-TB

patients in Indonesia. Int J Tuberc Lung Dis. 2014 May;18(5):564-70. doi:
10.5588/ijtld.13.0602. PubMed PMID: 24903794.

390: Yoshiyama T, Morimoto K, Okumura M, Sasaki Y, Ogata H, Shiraishi Y, Kudou S.
Long term outcome of multidrug-resistant TB patients in Fukujuji Hospital in
Japan. Trans R Soc Trop Med Hyg. 2014 Sep;108(9):589-90. doi:
10.1093/trstmh/tru080. Epub 2014 Jun 5. PubMed PMID: 24902580.

391: Tian K, Kang Y, Deng L, Liu H, Li H, Wang Z, Zhao G. [Effects of different
anesthesia depth on stress response in elderly patients undergoing elective
laparoscopic surgery for colorectal cancer]. Nan Fang Yi Ke Da Xue Xue Bao. 2014
May;34(5):694-8. Chinese. PubMed PMID: 24849439.

392: Ukwaja KN, Oshi DC, Oshi SN, Alobu I. Profile and treatment outcome of
smear-positive TB patients who failed to smear convert after 2 months of
treatment in Nigeria. Trans R Soc Trop Med Hyg. 2014 Jul;108(7):431-8. doi:
10.1093/trstmh/tru070. Epub 2014 May 20. PubMed PMID: 24846911.

393: Gumbo T, Chigutsa E, Pasipanodya J, Visser M, van Helden PD, Sirgel FA,
McIlleron H. The pyrazinamide susceptibility breakpoint above which combination
therapy fails. J Antimicrob Chemother. 2014 Sep;69(9):2420-5. doi:
10.1093/jac/dku136. Epub 2014 May 12. PubMed PMID: 24821594; PubMed Central
PMCID: PMC4130380.

394: Mfinanga SG, Kirenga BJ, Chanda DM, Mutayoba B, Mthiyane T, Yimer G, Ezechi
O, Connolly C, Kapotwe V, Muwonge C, Massaga J, Sinkala E, Kohi W, Lyantumba L,
Nyakoojo G, Luwaga H, Doulla B, Mzyece J, Kapata N, Vahedi M, Mwaba P, Egwaga S,
Adatu F, Pym A, Joloba M, Rustomjee R, Zumla A, Onyebujoh P. Early versus delayed
initiation of highly active antiretroviral therapy for HIV-positive adults with
newly diagnosed pulmonary tuberculosis (TB-HAART): a prospective, international,
randomised, placebo-controlled trial. Lancet Infect Dis. 2014
Jul;14(7):563-71.

doi: 10.1016/S1473-3099(14)70733-9. Epub 2014 May 5. Erratum in: Lancet Infect
Dis. 2014 Jul;14(7):548. PubMed PMID: 24810491.

395: Jain K, Desai M, Solanki R, Dikshit RK. Treatment outcome of standardized
regimen in patients with multidrug resistant tuberculosis. J Pharmacol
Pharmacother. 2014 Apr;5(2):145-9. doi: 10.4103/0976-500X.130062. PubMed PMID:
24799815; PubMed Central PMCID: PMC4008910.

396: Kalita J, Misra UK, Prasad S, Bhoi SK. Safety and efficacy of levofloxacin
versus rifampicin in tuberculous meningitis: an open-label randomized controlled
trial. J Antimicrob Chemother. 2014 Aug;69(8):2246-51. doi: 10.1093/jac/dku103.
Epub 2014 Apr 20. PubMed PMID: 24752957.

397: Magee MJ, Kempker RR, Kipiani M, Tukvadze N, Howards PP, Narayan KM,
Blumberg HM. Diabetes mellitus, smoking status, and rate of sputum culture
conversion in patients with multidrug-resistant tuberculosis: a cohort study from
the country of Georgia. PLoS One. 2014 Apr 15;9(4):e94890. doi:
10.1371/journal.pone.0094890. eCollection 2014. PubMed PMID: 24736471; PubMed
Central PMCID: PMC3988137.

398: Ong CW, Elkington PT, Friedland JS. Tuberculosis, pulmonary cavitation, and
matrix metalloproteinases. Am J Respir Crit Care Med. 2014 Jul 1;190(1):9-18.
doi: 10.1164/rccm.201311-2106PP. PubMed PMID: 24713029; PubMed Central PMCID:
PMC4226026.

399: Ibrahim LM, Oleribe OO, Nguku P, Tongwong GC, Mato LG, Longkyer MI, Ogiri S,
Nsubuga P. Evaluation of quality of TB control services by private health care
providers in Plateau state, Nigeria; 2012. Pan Afr Med J. 2014 Jan 31;17:77. doi:
10.11604/pamj.2014.17.77.3412. eCollection 2014. PubMed PMID: 24711883; PubMed
Central PMCID: PMC3972903.

400: Chand KS, Manchanda RK, Mittal R, Batra S, Banavaliker JN, De I. Homeopathic
treatment in addition to standard care in multi drug resistant pulmonary
tuberculosis: a randomized, double blind, placebo controlled clinical trial.
Homeopathy. 2014 Apr;103(2):97-107. doi: 10.1016/j.homp.2013.12.003. Erratum in:
Homeopathy. 2015 Jul;104(3):163. PubMed PMID: 24685414.

401: Hasan Z, Salahuddin N, Rao N, Aqeel M, Mahmood F, Ali F, Ashraf M, Rahman F,
Mahmood S, Islam M, Dildar B, Anwer T, Oiighor F, Sharif N, Ullah AR. Change in
serum CXCL10 levels during anti-tuberculosis treatment depends on vitamin D
status [Short Communication]. Int J Tuberc Lung Dis. 2014 Apr;18(4):466-9. doi:
10.5588/ijtld.13.0460. PubMed PMID: 24670704.

402: Heunis JC, Kigozi NG, van der Merwe S, Chikobvu P, Beyers N. Sex-related
trends in non-conversion of new smear-positive tuberculosis patients in the Free
State, South Africa. Public Health Action. 2014 Mar 21;4(1):66-71. doi:
10.5588/pha.13.0108. PubMed PMID: 26423765; PubMed Central PMCID: PMC4479093.

403: Nagu TJ, Spiegelman D, Hertzmark E, Aboud S, Makani J, Matee MI, Fawzi W,
Mugusi F. Anemia at the initiation of tuberculosis therapy is associated with
delayed sputum conversion among pulmonary tuberculosis patients in Dar-es-Salaam,
Tanzania. PLoS One. 2014 Mar 18;9(3):e91229. doi: 10.1371/journal.pone.0091229.
eCollection 2014. PubMed PMID: 24642636; PubMed Central PMCID: PMC3958362.

404: Pefura-Yone EW, Kengne AP, Kuaban C. Non-conversion of sputum culture among
patients with smear positive pulmonary tuberculosis in Cameroon: a prospective
cohort study. BMC Infect Dis. 2014 Mar 11;14:138. doi: 10.1186/1471-2334-14-138.
PubMed PMID: 24618155; PubMed Central PMCID: PMC3984706.

405: Lakshmanan M, Xavier AS. Bedaquiline - The first ATP synthase inhibitor

against multi drug resistant tuberculosis. J Young Pharm. 2013
Dec;5(4):112-5.
doi: 10.1016/j.jyp.2013.12.002. Epub 2013 Dec 30. Review. PubMed
PMID: 24563587;
PubMed Central PMCID: PMC3930122.

406: Stoffel C, Lorenz R, Arce M, Rico M, FernÃ¡ndez L, Imaz MS.
[Treatment of
pulmonary tuberculosis in a low-prevalence urban area. Compliance
and sputum
conversion]. Medicina (B Aires). 2014;74(1):9-18. Spanish. PubMed
PMID: 24561834.

407: Nakamura A, Hagiwara E, Hamai J, Taguri M, Terauchi Y. Impact
of underlying
diabetes and presence of lung cavities on treatment outcomes in
patients with
pulmonary tuberculosis. Diabet Med. 2014 Jun;31(6):707-13. doi:
10.1111/dme.12414. Epub 2014 Mar 13. PubMed PMID: 24547904.

408: Skrahin A, Ahmed RK, Ferrara G, Rane L, Poiret T, Isaikina Y,
Skrahina A,
Zumla A, Maeurer MJ. Autologous mesenchymal stromal cell infusion as
adjunct
treatment in patients with multidrug and extensively drug-resistant
tuberculosis:
an open-label phase 1 safety trial. Lancet Respir Med. 2014
Feb;2(2):108-22. doi:
10.1016/S2213-2600(13)70234-0. Epub 2014 Jan 9. PubMed PMID:
24503266.

409: Viswanathan V, Vigneswari A, Selvan K, Satyavani K, Rajeswari
R, Kapur A.
Effect of diabetes on treatment outcome of smear-positive pulmonary
tuberculosis--a report from South India. J Diabetes Complications.
2014
Mar-Apr;28(2):162-5. doi: 10.1016/j.jdiacomp.2013.12.003. Epub 2013
Dec 24.
PubMed PMID: 24461545.

410: Zheng Y, Li XG, Wang QZ, Ma AG, Bygbjerg IC, Sun YY, Li Y,
Zheng MC, Wang X.
Enhancement of vitamin A combined vitamin D supplementation on
immune response to
Bacille Calmette-GuÃ©rin vaccine revaccinated in Chinese infants.
Asian Pac J Trop
Med. 2014 Feb;7(2):130-5. doi: 10.1016/S1995-7645(14)60008-0. PubMed
PMID:
24461526.

411: Pefura-Yone EW, Kuaban C, Assamba-Mpom SA, Moifo B, Kengne AP. Derivation,
validation and comparative performance of a simplified chest X-ray score for
assessing the severity and outcome of pulmonary tuberculosis. Clin Respir J. 2015
Apr;9(2):157-64. doi: 10.1111/crj.12112. Epub 2014 Feb 21. PubMed PMID: 24460763.

412: Wallace RJ Jr, Brown-Elliott BA, McNulty S, Philley JV, Killingley J, Wilson
RW, York DS, Shepherd S, Griffith DE. Macrolide/Azalide therapy for
nodular/bronchiectatic mycobacterium avium complex lung disease. Chest. 2014
Aug;146(2):276-282. doi: 10.1378/chest.13-2538. PubMed PMID: 24457542; PubMed
Central PMCID: PMC4694082.

413: Marx RE. The deception and fallacies of sponsored randomized prospective
double-blinded clinical trials: the bisphosphonate research example. Int J Oral
Maxillofac Implants. 2014 Jan-Feb;29(1):e37-44. doi: 10.11607/jomi.te40. PubMed
PMID: 24451886.

414: Chaudhry LA, Al-Shamri AS, Ba Essa EM, Robert AA, Al-Solaiman S, Al-Nwasser
AH. The rates of sputum conversion among new smear positive open pulmonary
tuberculosis patients treated under directly observed treatment, short course
strategy. Saudi Med J. 2014 Jan;35(1):39-43. PubMed PMID: 24445888.

415: Pietersen E, Ignatius E, Streicher EM, Mastrapa B, Padanilam X, Pooran A,
Badri M, Lesosky M, van Helden P, Sirgel FA, Warren R, Dheda K. Long-term
outcomes of patients with extensively drug-resistant tuberculosis in South
Africa: a cohort study. Lancet. 2014 Apr 5;383(9924):1230-9. doi:
10.1016/S0140-6736(13)62675-6. Epub 2014 Jan 17. PubMed PMID: 24439237.

416: Duan HF, Chu NH, Wang QF, Wang J, Huang HR, Liang Q. [Mycobacterium
abscessus group lung disease: case reports and review of the literature].
Zhonghua Jie He He Hu Xi Za Zhi. 2013 Sep;36(9):671-4. Review. Chinese. PubMed
PMID: 24423821.

417: Bonnet M, Baudin E, Jani IV, Nunes E, Verhoustraten F, Calmy A, Bastos R,
Bhatt NB, Michon C. Incidence of paradoxical tuberculosis-associated immune
reconstitution inflammatory syndrome and impact on patient outcome. PLoS One.
2013 Dec 18;8(12):e84585. doi: 10.1371/journal.pone.0084585. eCollection 2013.
Erratum in: PLoS One. 2014;9(1).
doi:10.1371/annotation/15d01128-2495-4b95-8fe8-8702190fdb0e. PubMed PMID:
24367678; PubMed Central PMCID: PMC3867516.

418: Kim SY, Koh WJ, Park HY, Jeon K, Kwon OJ, Cho SN, Shin SJ. Changes in serum
immunomolecules during antibiotic therapy for Mycobacterium avium complex lung
disease. Clin Exp Immunol. 2014 Apr;176(1):93-101. doi: 10.1111/cei.12253. PubMed
PMID: 24354934; PubMed Central PMCID: PMC3958158.

419: Roongruangpitayakul C, Chuchottaworn C. Outcomes of MDR/XDR-TB patients
treated with linezolid: experience in Thailand. J Med Assoc Thai. 2013
Oct;96(10):1273-82. PubMed PMID: 24350407.

420: Takeuchi M, Kurata M, Hayashi S. [Nutritional assessment upon admission for
predicting delay in negative bacillary conversion among patients treated for
pulmonary tuberculosis]. Kekkaku. 2013 Oct;88(10):697-702. Japanese. PubMed PMID:
24341174.

421: Nijenbandring de Boer R, Oliveira e Souza Filho JB, Cobelens F, Ramalho Dde
P, Campino Miranda PF, Logo Kd, Oliveira H, Mesquita E, Oliveira MM, Kritski A.
Delayed culture conversion due to cigarette smoking in active pulmonary
tuberculosis patients. Tuberculosis (Edinb). 2014 Jan;94(1):87-91. doi:
10.1016/j.tube.2013.10.005. Epub 2013 Oct 31. PubMed PMID: 24321739.

422: Miwa S, Shirai M, Toyoshima M, Shirai T, Yasuda K, Yokomura K, Yamada T,
Masuda M, Inui N, Chida K, Suda T, Hayakawa H. Efficacy of clarithromycin and

ethambutol for Mycobacterium avium complex pulmonary disease. A
preliminary
study. Ann Am Thorac Soc. 2014 Jan;11(1):23-9. doi:
10.1513/AnnalsATS.201308-266OC. PubMed PMID: 24298907.

423: Singla R, Bharty SK, Gupta UA, Khayyam KU, Vohra V, Singla N,
Myneedu VP,
Behera D. Sputum smear positivity at two months in previously
untreated pulmonary
tuberculosis patients. Int J Mycobacteriol. 2013 Dec;2(4):199-205.
doi:
10.1016/j.ijmyco.2013.08.002. Epub 2013 Sep 14. PubMed PMID:
26786122.

424: Rueda ZV, LÃ³pez L, VÃ©lez LA, MarÃn D, Giraldo MR, Pulido H,
Orozco LC,
Montes F, ArbelÃ¡ez MP. High incidence of tuberculosis, low
sensitivity of current
diagnostic scheme and prolonged culture positivity in four colombian
prisons. A
cohort study. PLoS One. 2013 Nov 21;8(11):e80592. doi:
10.1371/journal.pone.0080592. eCollection 2013. PubMed PMID:
24278293; PubMed
Central PMCID: PMC3836852.

425: Mpagama SG, Ndusilo N, Stroup S, Kumburu H, Peloquin CA, Gratz
J, Houpt ER,
Kibiki GS, Heysell SK. Plasma drug activity in patients on treatment
for
multidrug-resistant tuberculosis. Antimicrob Agents Chemother.
2014;58(2):782-8.
doi: 10.1128/AAC.01549-13. Epub 2013 Nov 18. PubMed PMID: 24247125;
PubMed
Central PMCID: PMC3910816.

426: Shah I, Meshram L. High dose versus low dose steroids in
children with
tuberculous meningitis. J Clin Neurosci. 2014 May;21(5):761-4. doi:
10.1016/j.jocn.2013.07.021. Epub 2013 Nov 11. PubMed PMID: 24231560.

427: Luetkemeyer AF, Kendall MA, Nyirenda M, Wu X, Ive P, Benson CA,
Andersen JW,
Swindells S, Sanne IM, Havlir DV, Kumwenda J; Adult AIDS Clinical
Trials Group
A5221 Study Team. Tuberculosis immune reconstitution inflammatory
syndrome in
A5221 STRIDE: timing, severity, and implications for HIV-TB
programs. J Acquir
Immune Defic Syndr. 2014 Apr 1;65(4):423-8. doi:
10.1097/QAI.0000000000000030.
PubMed PMID: 24226057; PubMed Central PMCID: PMC3943693.

428: Bouchikh M, Achir A, Caidi M, El Aziz S, Benosman A. [Role of pulmonary
resections in management of multidrug-resistant tuberculosis. A monocentric
series of 29 patients]. Rev Pneumol Clin. 2013 Dec;69(6):326-30. doi:
10.1016/j.pneumo.2013.09.002. Epub 2013 Nov 6. French. PubMed PMID: 24210152.

429: Ungurs MJ, Sinden NJ, Stockley RA. Progranulin is a substrate for
neutrophil-elastase and proteinase-3 in the airway and its concentration
correlates with mediators of airway inflammation in COPD. Am J Physiol Lung Cell
Mol Physiol. 2014 Jan 1;306(1):L80-7. doi: 10.1152/ajplung.00221.2013. Epub 2013
Nov 1. PubMed PMID: 24186875.

430: Mukherjee A, Saini S, Kabra SK, Gupta N, Singh V, Singh S, Bhatnagar S,
Saini D, Grewal HM, Lodha R; Delhi TB Study group. Effect of micronutrient
deficiency on QuantiFERON-TB Gold In-Tube test and tuberculin skin test in
diagnosis of childhood intrathoracic tuberculosis. Eur J Clin Nutr. 2014
Jan;68(1):38-42. doi: 10.1038/ejcn.2013.216. Epub 2013 Oct 30. PubMed PMID:
24169461.

431: Centers for Disease Control and Prevention. Provisional CDC guidelines for
the use and safety monitoring of bedaquiline fumarate (Sirturo) for the treatment
of multidrug-resistant tuberculosis. MMWR Recomm Rep. 2013 Oct 25;62(RR-09):1-12.
Erratum in: MMWR Recomm Rep. 2013 Nov 15;62(45):906. PubMed PMID: 24157696.

432: Tadokera R, Meintjes GA, Wilkinson KA, Skolimowska KH, Walker N, Friedland
JS, Maartens G, Elkington PT, Wilkinson RJ. Matrix metalloproteinases and tissue
damage in HIV-tuberculosis immune reconstitution inflammatory syndrome. Eur J
Immunol. 2014 Jan;44(1):127-36. doi: 10.1002/eji.201343593. Epub 2013 Oct 30.
PubMed PMID: 24136296; PubMed Central PMCID: PMC3992843.

433: Mi F, Tan S, Liang L, Harries AD, Hinderaker SG, Lin Y, Yue W, Chen X, Liang
B, Gong F, Du J. Diabetes mellitus and tuberculosis: pattern of tuberculosis,
two-month smear conversion and treatment outcomes in Guangzhou, China. Trop Med
Int Health. 2013 Nov;18(11):1379-85. doi: 10.1111/tmi.12198. Epub 2013 Sep 23.
PubMed PMID: 24112411.

434: Dooley KE, Nuermberger EL, Diacon AH. Pipeline of drugs for related
diseases: tuberculosis. Curr Opin HIV AIDS. 2013 Nov;8(6):579-85. doi:
10.1097/COH.0000000000000009. Review. PubMed PMID: 24100880; PubMed Central
PMCID: PMC5206795.

435: Vashishtha R, Mohan K, Singh B, Devarapu SK, Sreenivas V, Ranjan S, Gupta D,
Sinha S, Sharma SK. Efficacy and safety of thrice weekly DOTS in tuberculosis
patients with and without HIV co-infection: an observational study. BMC Infect
Dis. 2013 Oct 7;13:468. doi: 10.1186/1471-2334-13-468. PubMed PMID: 24099345;
PubMed Central PMCID: PMC3852441.

436: Dobler CC, Luu Q, Marks GB. What patient factors predict physicians'
decision not to treat latent tuberculosis infection in tuberculosis contacts?
PLoS One. 2013 Sep 30;8(9):e76552. doi: 10.1371/journal.pone.0076552. eCollection
2013. PubMed PMID: 24098794; PubMed Central PMCID: PMC3786986.

437: Kenangalem E, Waramori G, Pontororing GJ, Sandjaja, Tjitra E, Maguire G,
Kelly PM, Anstey NM, Ralph AP. Tuberculosis outcomes in Papua, Indonesia: the
relationship with different body mass index characteristics between papuan and
non-Papuan ethnic groups. PLoS One. 2013 Sep 27;8(9):e76077. doi:
10.1371/journal.pone.0076077. eCollection 2013. PubMed PMID: 24086690; PubMed
Central PMCID: PMC3785441.

438: Kayigamba FR, Bakker MI, Mugisha V, De Naeyer L, Gasana M, Cobelens F, van

der Loeff MS. Adherence to tuberculosis treatment, sputum smear conversion and
mortality: a retrospective cohort study in 48 Rwandan clinics. PLoS One. 2013 Sep
16;8(9):e73501. doi: 10.1371/journal.pone.0073501. eCollection 2013. PubMed PMID:
24066053; PubMed Central PMCID: PMC3774710.

439: Tripathy SK, Kumar P, Sagili KD, Enarson DA. Effectiveness of a
community-based observation of anti-tuberculosis treatment in Bangalore City,
India, 2010-2011. Public Health Action. 2013 Sep 21;3(3):230-4. doi:
10.5588/pha.13.0043. PubMed PMID: 26393035; PubMed Central PMCID:
PMC4463128.

440: Brust JC, Berman AR, Zalta B, Haramati LB, Ning Y, Heo M, van der Merwe TL,
Bamber S, Moll AP, Friedland GH, Shah NS, Gandhi NR. Chest radiograph findings
and time to culture conversion in patients with multidrug-resistant tuberculosis
and HIV in Tugela Ferry, South Africa. PLoS One. 2013 Sep 6;8(9):e73975. doi:
10.1371/journal.pone.0073975. eCollection 2013. PubMed PMID: 24040132; PubMed
Central PMCID: PMC3765317.

441: Seung KJ, Becerra MC, Atwood SS, AlcÃ¡ntara F, Bonilla CA, Mitnick CD.
Salvage therapy for multidrug-resistant tuberculosis. Clin Microbiol Infect. 2014
May;20(5):441-6. doi: 10.1111/1469-0691.12335. Epub 2013 Aug 30. PubMed PMID:
23991934.

442: Yang SH, Zhan P, Mao HH, Shi XD, Wang LL. Perfusing chemotherapy by
percutaneous lung puncture "holing" for pulmonary tuberculoma-a ten-year single
center experience. J Thorac Dis. 2013 Aug;5(4):466-71. doi:
10.3978/j.issn.2072-1439.2013.07.03. PubMed PMID: 23991304; PubMed Central PMCID:
PMC3755674.

443: Ralph AP, Waramori G, Pontororing GJ, Kenangalem E, Wiguna A, Tjitra E,
Sandjaja, Lolong DB, Yeo TW, Chatfield MD, Soemanto RK, Bastian I, Lumb R,
Maguire GP, Eisman J, Price RN, Morris PS, Kelly PM, Anstey NM. L-arginine and

vitamin D adjunctive therapies in pulmonary tuberculosis: a randomised,
double-blind, placebo-controlled trial. PLoS One. 2013 Aug 14;8(8):e70032. doi:
10.1371/journal.pone.0070032. eCollection 2013. PubMed PMID: 23967066; PubMed
Central PMCID: PMC3743888.

444: Schiff M, Weinblatt ME, Valente R, van der Heijde D, Citera G, Elegbe A,
Maldonado M, Fleischmann R. Head-to-head comparison of subcutaneous abatacept
versus adalimumab for rheumatoid arthritis: two-year efficacy and safety findings
from AMPLE trial. Ann Rheum Dis. 2014 Jan;73(1):86-94. doi:
10.1136/annrheumdis-2013-203843. Epub 2013 Aug 20. PubMed PMID: 23962455; PubMed
Central PMCID: PMC3888617.

445: Unsematham S, Kateruttanakul P. Factors predicting sputum smear conversion
and treatment outcomes in new smear-positive pulmonary tuberculosis. J Med Assoc
Thai. 2013 Jun;96(6):644-9. PubMed PMID: 23951819.

446: Chang B, Han SG, Kim W, Ko Y, Song J, Hong G, Eom JS, Lee JH, Jhun BW, Koh
WJ. Normalization of Elevated CA 19-9 Level after Treatment in a Patient with the
Nodular Bronchiectatic Form of Mycobacterium abscessus Lung Disease. Tuberc
Respir Dis (Seoul). 2013 Jul;75(1):25-7. doi: 10.4046/trd.2013.75.1.25. Epub 2013
Jul 31. PubMed PMID: 23946755; PubMed Central PMCID: PMC3741470.

447: Koh WJ, Lee SH, Kang YA, Lee CH, Choi JC, Lee JH, Jang SH, Yoo KH, Jung KH,
Kim KU, Choi SB, Ryu YJ, Chan Kim K, Um S, Kwon YS, Kim YH, Choi WI, Jeon K,
Hwang YI, Kim SJ, Lee YS, Heo EY, Lee J, Ki YW, Shim TS, Yim JJ. Comparison of
levofloxacin versus moxifloxacin for multidrug-resistant tuberculosis. Am J
Respir Crit Care Med. 2013 Oct 1;188(7):858-64. doi: 10.1164/rccm.201303-0604OC.
PubMed PMID: 23927582.

448: Flora MS, Amin MN, Karim MR, Afroz S, Islam S, Alam A, Hossain M. Risk
factors of multi-drug-resistant tuberculosis in Bangladeshi population: a case

control study. Bangladesh Med Res Counc Bull. 2013 Apr;39(1):34-41. PubMed PMID:
23923410.

449: Kobayashi H, Koyanagi K, Kato O, Oe T. [Outbreak of extensively drug-resistant pulmonary tuberculosis in a hemodialysis facility]. Kekkaku. 2013
May;88(5):477-84. Japanese. PubMed PMID: 23882728.

450: da Silva TP, Giacoia-Gripp CB, Schmaltz CA, Sant Anna FM, Rolla V, Morgado
MG. T Cell Activation and Cytokine Profile of Tuberculosis and HIV-Positive
Individuals during Antituberculous Treatment and Efavirenz-Based Regimens. PLoS
One. 2013 Jun 19;8(6):e66095. doi: 10.1371/journal.pone.0066095. Print 2013.
PubMed PMID: 23840403; PubMed Central PMCID: PMC3686825.

451: Wejse C, Furtado A, Camara C, LÃ¼neborg-Nielsen M, Sodemann M, Gerstoft J,
Katzenstein TL. Impact of tuberculosis treatment on CD4 cell count, HIV RNA, and
p24 antigen in patients with HIV and tuberculosis. Int J Infect Dis. 2013
Oct;17(10):e907-12. doi: 10.1016/j.ijid.2013.05.003. Epub 2013 Jun 28. PubMed
PMID: 23816410.

452: Faurholt-Jepsen D. The double burden. Dan Med J. 2013 Jul;60(7):B4673.
PubMed PMID: 23809978.

453: Chang KC, Yew WW, Tam CM, Leung CC. WHO group 5 drugs and difficult
multidrug-resistant tuberculosis: a systematic review with cohort analysis and
meta-analysis. Antimicrob Agents Chemother. 2013 Sep;57(9):4097-104. doi:
10.1128/AAC.00120-13. Epub 2013 Jun 17. Review. PubMed PMID: 23774431; PubMed
Central PMCID: PMC3754286.

454: Sandborn WJ, Feagan BG, Marano C, Zhang H, Strauss R, Johanns J, Adedokun
OJ, Guzzo C, Colombel JF, Reinisch W, Gibson PR, Collins J, JÃ¤rnerot G, Rutgeerts
P; PURSUIT-Maintenance Study Group. Subcutaneous golimumab maintains clinical
response in patients with moderate-to-severe ulcerative colitis.

Gastroenterology. 2014 Jan;146(1):96-109.e1. doi: 10.1053/j.gastro.2013.06.010.
Epub 2013 Jun 14. PubMed PMID: 23770005.

455: Duangrithi D, Thanachartwet V, Desakorn V, Jitruckthai P, Phojanamongkolkij
K, Rienthong S, Chuchottaworn C, Pitisuttithum P. Impact of diabetes mellitus on
clinical parameters and treatment outcomes of newly diagnosed pulmonary
tuberculosis patients in Thailand. Int J Clin Pract. 2013 Nov;67(11):1199-209.
doi: 10.1111/ijcp.12215. Epub 2013 Jun 10. PubMed PMID: 23750554; PubMed Central
PMCID: PMC4232236.

456: Xie B, Yang Y, He W, Xie D, Jiang G. Pulmonary resection in the treatment of
43 patients with well-localized, cavitary pulmonary multidrug-resistant
tuberculosis in Shanghai. Interact Cardiovasc Thorac Surg. 2013 Sep;17(3):455-9.
doi: 10.1093/icvts/ivt251. Epub 2013 Jun 7. PubMed PMID: 23748869; PubMed Central
PMCID: PMC3745155.

457: Ziganshina LE, Titarenko AF, Davies GR. Fluoroquinolones for treating
tuberculosis (presumed drug-sensitive). Cochrane Database Syst Rev. 2013 Jun
6;(6):CD004795. doi: 10.1002/14651858.CD004795.pub4. Review. PubMed PMID:
23744519.

458: Click ES, Winston CA, Oeltmann JE, Moonan PK, Mac Kenzie WR. Association
between Mycobacterium tuberculosis lineage and time to sputum culture conversion.
Int J Tuberc Lung Dis. 2013 Jul;17(7):878-84. doi: 10.5588/ijtld.12.0732. PubMed
PMID: 23743308.

459: Ralph AP, Yeo TW, Salome CM, Waramori G, Pontororing GJ, Kenangalem E,
Sandjaja, Tjitra E, Lumb R, Maguire GP, Price RN, Chatfield MD, Kelly PM, Anstey
NM. Impaired pulmonary nitric oxide bioavailability in pulmonary tuberculosis:
association with disease severity and delayed mycobacterial clearance with

treatment. J Infect Dis. 2013 Aug 15;208(4):616-26. doi: 10.1093/infdis/jit248.
Epub 2013 Jun 3. PubMed PMID: 23737604; PubMed Central PMCID: PMC3719909.

460: Carroll MW, Jeon D, Mountz JM, Lee JD, Jeong YJ, Zia N, Lee M, Lee J, Via
LE, Lee S, Eum SY, Lee SJ, Goldfeder LC, Cai Y, Jin B, Kim Y, Oh T, Chen RY, Dodd
LE, Gu W, Dartois V, Park SK, Kim CT, Barry CE 3rd, Cho SN. Efficacy and safety
of metronidazole for pulmonary multidrug-resistant tuberculosis. Antimicrob
Agents Chemother. 2013 Aug;57(8):3903-9. doi: 10.1128/AAC.00753-13. Epub 2013 Jun
3. PubMed PMID: 23733467; PubMed Central PMCID: PMC3719751.

461: Narendran G, Andrade BB, Porter BO, Chandrasekhar C, Venkatesan P, Menon PA,
Subramanian S, Anbalagan S, Bhavani KP, Sekar S, Padmapriyadarshini C, Kumar S,
Ravichandran N, Raja K, Bhanu K, Mahilmaran A, Sekar L, Sher A, Sereti I,
Swaminathan S. Paradoxical tuberculosis immune reconstitution inflammatory
syndrome (TB-IRIS) in HIV patients with culture confirmed pulmonary tuberculosis
in India and the potential role of IL-6 in prediction. PLoS One. 2013 May
17;8(5):e63541. doi: 10.1371/journal.pone.0063541. Print 2013. PubMed PMID:
23691062; PubMed Central PMCID: PMC3656926.

462: Burhan E, Ruesen C, Ruslami R, Ginanjar A, Mangunnegoro H, Ascobat P,
Donders R, van Crevel R, Aarnoutse R. Isoniazid, rifampin, and pyrazinamide
plasma concentrations in relation to treatment response in Indonesian pulmonary
tuberculosis patients. Antimicrob Agents Chemother. 2013 Aug;57(8):3614-9. doi:
10.1128/AAC.02468-12. Epub 2013 May 20. PubMed PMID: 23689725; PubMed Central
PMCID: PMC3719785.

463: Masuyama H, Igari H. [Reconsideration of the admission and discharge
criteria of tuberculosis patients in Japan]. Kekkaku. 2013 Mar;88(3):373-85.
Japanese. PubMed PMID: 23672177.

464: Ogawa K, Sano C. [Strategies for Mycobacterium avium complex infection
control in Japan: how do they improve the present situation?]. Kekkaku. 2013
Mar;88(3):355-71. Japanese. PubMed PMID: 23672176.

465: Shean K, Streicher E, Pieterson E, Symons G, van Zyl Smit R, Theron G,
Lehloenya R, Padanilam X, Wilcox P, Victor TC, van Helden P, Grobusch MP, Warren
R, Badri M, Dheda K. Drug-associated adverse events and their relationship with
outcomes in patients receiving treatment for extensively drug-resistant
tuberculosis in South Africa. PLoS One. 2013 May 7;8(5):e63057. doi:
10.1371/journal.pone.0063057. Print 2013. Erratum in: PLoS One. 2013;8(5).
doi:10.1371/annotation/644591a8-8ae6-450e-974e-1cd1f08f52c7. Groubusch, Martin
[corrected to Grobusch, Martin P]. PubMed PMID: 23667572; PubMed Central PMCID:
PMC3646906.

466: Chang KC, Yew WW, Cheung SW, Leung CC, Tam CM, Chau CH, Wen PK, Chan RC. Can
intermittent dosing optimize prolonged linezolid treatment of difficult
multidrug-resistant tuberculosis? Antimicrob Agents Chemother. 2013
Jul;57(7):3445-9. doi: 10.1128/AAC.00388-13. Epub 2013 May 6. PubMed PMID:
23650165; PubMed Central PMCID: PMC3697330.

467: Faurholt-Jepsen D, Range N, PrayGod G, Jeremiah K, Faurholt-Jepsen M, Aabye
MG, Changalucha J, Christensen DL, Grewal HM, Martinussen T, Krarup H, Witte DR,
Andersen AB, Friis H. Diabetes is a strong predictor of mortality during
tuberculosis treatment: a prospective cohort study among tuberculosis patients
from Mwanza, Tanzania. Trop Med Int Health. 2013 Jul;18(7):822-9. doi:
10.1111/tmi.12120. Epub 2013 May 6. PubMed PMID: 23648145.

468: O'Donnell MR, Padayatchi N, Kvasnovsky C, Werner L, Master I, Horsburgh CR
Jr. Treatment outcomes for extensively drug-resistant tuberculosis and HIV
co-infection. Emerg Infect Dis. 2013 Mar;19(3):416-24. doi:
10.3201/eid1903.120998. PubMed PMID: 23622055; PubMed Central PMCID:
PMC3647656.

469: Ugarte-Gil CA, Elkington P, Gilman RH, Coronel J, Tezera LB, Bernabe-Ortiz
A, Gotuzzo E, Friedland JS, Moore DA. Induced sputum MMP-1, -3 & -8
concentrations during treatment of tuberculosis. PLoS One. 2013 Apr
22;8(4):e61333. doi: 10.1371/journal.pone.0061333. Print 2013. PubMed PMID:
23613834; PubMed Central PMCID: PMC3632571.

470: Jiang RH, Xu HB, Li L. Comparative roles of moxifloxacin and levofloxacin in
the treatment of pulmonary multidrug-resistant tuberculosis: a retrospective
study. Int J Antimicrob Agents. 2013 Jul;42(1):36-41. doi:
10.1016/j.ijantimicag.2013.02.019. Epub 2013 Apr 11. PubMed PMID:
23582696.

471: Mandal PK, Mandal A, Bhattacharyya SK. Comparing the Daily Versus the
Intermittent Regimens of the Anti-Tubercular Chemotherapy in the Initial
Intensive Phase in Non-HIV, Sputum Positive, Pulmonary Tuberculosis Patients. J
Clin Diagn Res. 2013 Feb;7(2):292-5. doi:
10.7860/JCDR/2013/5122.2750. Epub 2012
Dec 24. PubMed PMID: 23542708; PubMed Central PMCID: PMC3592295.

472: Jayakody W, Harries AD, Malhotra S, de Alwis S, Samaraweera S, Pallewatta N.
Characteristics and outcomes of tuberculosis patients who fail to smear convert
at two months in Sri Lanka. Public Health Action. 2013 Mar
21;3(1):26-30. doi:
10.5588/pha.12.0090. PubMed PMID: 26392992; PubMed Central PMCID:
PMC4463095.

473: Koh WJ, Hong G, Kim SY, Jeong BH, Park HY, Jeon K, Kwon OJ, Lee SH, Kim CK,
Shin SJ. Treatment of refractory Mycobacterium avium complex lung disease with a
moxifloxacin-containing regimen. Antimicrob Agents Chemother. 2013
May;57(5):2281-5. doi: 10.1128/AAC.02281-12. Epub 2013 Mar 11. PubMed PMID:
23478956; PubMed Central PMCID: PMC3632919.

474: Huang F, Gu J, Zhu P, Bao C, Xu J, Xu H, Wu H, Wang G, Shi Q, Andhivarothai
N, Anderson J, Pangan AL. Efficacy and safety of adalimumab in Chinese adults
with active ankylosing spondylitis: results of a randomised, controlled trial.

Ann Rheum Dis. 2014 Mar;73(3):587-94. doi: 10.1136/annrheumdis-2012-202533. Epub
2013 Mar 8. PubMed PMID: 23475983.

475: Escalante P, McKean-Cowdin R, Ramaswamy SV, Williams-Bouyer N, Teeter LD,
Jones BE, Graviss EA. Can mycobacterial katG genetic changes in isoniazid-resistant tuberculosis influence human disease features? Int J Tuberc
Lung Dis. 2013 May;17(5):644-51. doi: 10.5588/ijtld.12.0380. Epub 2013 Mar 1.
PubMed PMID: 23453008.

476: Wang Q, Ma A, Bygbjerg IC, Han X, Liu Y, Zhao S, Cai J. Rationale and design
of a randomized controlled trial of the effect of retinol and vitamin D
supplementation on treatment in active pulmonary tuberculosis patients with
diabetes. BMC Infect Dis. 2013 Feb 26;13:104. doi: 10.1186/1471-2334-13-104.
PubMed PMID: 23442225; PubMed Central PMCID: PMC3599006.

477: Parikh R, Nataraj G, Kanade S, Khatri V, Mehta P. Time to sputum conversion
in smear positive pulmonary TB patients on category I DOTS and factors delaying
it. J Assoc Physicians India. 2012 Aug;60:22-6. PubMed PMID: 23405517.

478: Tiwari S, Kumar A, Kapoor SK. Relationship between sputum smear grading and
smear conversion rate and treatment outcome in the patients of pulmonary
tuberculosis undergoing dots--a prospective cohort study. Indian J Tuberc. 2012
Jul;59(3):135-40. PubMed PMID: 23362709.

479: Mayosi BM, Ntsekhe M, Bosch J, Pogue J, Gumedze F, Badri M, Jung H, Pandie
S, Smieja M, Thabane L, Francis V, Thomas KM, Thomas B, Awotedu AA, Magula NP,
Naidoo DP, Damasceno A, Banda AC, Mutyaba A, Brown B, Ntuli P, Mntla P,
Ntyintyane L, Ramjee R, Manga P, Kirenga B, Mondo C, Russell JB, Tsitsi JM,
Peters F, Essop MR, Barasa AF, Mijinyawa MS, Sani MU, Olunuga T, Ogah O, Adebiyi
A, Aje A, Ansa V, Ojji D, Danbauchi S, Hakim J, Matenga J, Yusuf S. Rationale and

design of the Investigation of the Management of Pericarditis (IMPI) trial: a 2 Ã—
2 factorial randomized double-blind multicenter trial of adjunctive prednisolone
and Mycobacterium w immunotherapy in tuberculous pericarditis. Am Heart J. 2013
Feb;165(2):109-15.e3. doi: 10.1016/j.ahj.2012.08.006. Epub 2012 Dec 13. PubMed
PMID: 23351812.

480: Salahuddin N, Ali F, Hasan Z, Rao N, Aqeel M, Mahmood F. Vitamin D
accelerates clinical recovery from tuberculosis: results of the SUCCINCT Study
[Supplementary Cholecalciferol in recovery from tuberculosis]. A randomized,
placebo-controlled, clinical trial of vitamin D supplementation in patients with
pulmonary tuberculosis'. BMC Infect Dis. 2013 Jan 19;13:22. doi:
10.1186/1471-2334-13-22. PubMed PMID: 23331510; PubMed Central PMCID: PMC3556334.

481: Hafkin J, Modongo C, Newcomb C, Lowenthal E, MacGregor RR, Steenhoff AP,
Friedman H, Bisson GP. Impact of the human immunodeficiency virus on early
multidrug-resistant tuberculosis treatment outcomes in Botswana. Int J Tuberc
Lung Dis. 2013 Mar;17(3):348-53. doi: 10.5588/ijtld.12.0100. Epub 2013 Jan 14.
PubMed PMID: 23321297; PubMed Central PMCID: PMC4393740.

482: Maciel EL, Brioschi AP, Peres RL, Guidoni LM, Ribeiro FK, Hadad DJ, Vinhas
SA, Zandonade E, Palaci M, Dietze R, Johnson JL. Smoking and 2-month culture
conversion during anti-tuberculosis treatment. Int J Tuberc Lung Dis. 2013
Feb;17(2):225-8. doi: 10.5588/ijtld.12.0426. PubMed PMID: 23317958; PubMed
Central PMCID: PMC4497564.

483: BabalÄ±k A, CalÄ±ÅŸÄ±r H, BakÄ±rcÄ± N, Arda H, KÄ±zÄ±ltaÅŸ Åž, OruÃ§ K, Ã‡etintaÅŸ G. The
outcome of tuberculosis cases with persistent smear positivity at the end of
extended initial phase. Tuberk Toraks. 2012;60(4):344-9. PubMed PMID: 23289464.

484: Chien JY, Chen YT, Shu CC, Lee JJ, Wang JY, Yu CJ, Yang PC. Outcome

correlation of smear-positivity for acid-fast bacilli at the fifth month of
treatment in non-multidrug-resistant TB. Chest. 2013 Jun;143(6):1725-1732. doi:
10.1378/chest.12-2051. PubMed PMID: 23288018.

485: Kim S, Lee J, Lee J. Changes in chest CT findings of pulmonary tuberculosis
after linezolid treatment. Springerplus. 2013 Nov 18;2:615. doi:
10.1186/2193-1801-2-615. eCollection 2013. PubMed PMID: 25674418; PubMed Central
PMCID: PMC4320169.

486: Horita N, Miyazawa N, Yoshiyama T, Kojima R, Omori N, Inoue M, Kaneko T,
Ishigatsubo Y. The presence of pretreatment cavitations and the bacterial load on
smears predict tuberculosis infectivity negative conversion judged on sputum
smear or culture. Intern Med. 2012;51(24):3367-72. Epub 2012 Dec 15. PubMed PMID:
23257521.

487: JimÃ©nez-Corona ME, Cruz-Hervert LP, GarcÃ-a-GarcÃ-a L, Ferreyra-Reyes L,
Delgado-SÃ¡nchez G, Bobadilla-Del-Valle M, Canizales-Quintero S, Ferreira-Guerrero
E, BÃ¡ez-SaldaÃ±a R, TÃ©llez-VÃ¡zquez N, Montero-Campos R, Mongua-Rodriguez N,
MartÃ-nez-Gamboa RA, Sifuentes-Osornio J, Ponce-de-LeÃ³n A. Association of diabetes
and tuberculosis: impact on treatment and post-treatment outcomes. Thorax. 2013
Mar;68(3):214-20. doi: 10.1136/thoraxjnl-2012-201756. Epub 2012 Dec 18. PubMed
PMID: 23250998; PubMed Central PMCID: PMC3585483.

488: Kimball AB, Kerdel F, Adams D, Mrowietz U, Gelfand JM, Gniadecki R, Prens
EP, Schlessinger J, Zouboulis CC, van der Zee HH, Rosenfeld M, Mulani P, Gu Y,
Paulson S, Okun M, Jemec GB. Adalimumab for the treatment of moderate to severe
Hidradenitis suppurativa: a parallel randomized trial. Ann Intern Med. 2012 Dec
18;157(12):846-55. doi: 10.7326/0003-4819-157-12-201212180-00004. PubMed PMID:
23247938.

489: Weiss CH, Glassroth J. Pulmonary disease caused by nontuberculous

mycobacteria. Expert Rev Respir Med. 2012 Dec;6(6):597-612; quiz 613. doi:
10.1586/ers.12.58. Review. Erratum in: Expert Rev Respir Med. 2013 Apr;7(2):195-6. PubMed PMID: 23234447.

490: Hanrahan CF, Dorman SE, Erasmus L, Koornhof H, Coetzee G, Golub JE. The
impact of expanded testing for multidrug resistant tuberculosis using genotype
[correction of geontype] MTBDRplus in South Africa: an observational cohort
study. PLoS One. 2012;7(11):e49898. doi: 10.1371/journal.pone.0049898. Epub 2012
Nov 30. Erratum in: PLoS One. 2013;8(6). doi:
10.1371/annotation/47420ec0-db00-4994-a0de-86b5e5d713f4. PubMed PMID: 23226229;
PubMed Central PMCID: PMC3511489.

491: Janssen S, Padanilam X, Louw R, Mahanyele R, Coetzee G, HÃ¤nscheid T,
Leenstra T, Grobusch MP. How many sputum culture results do we need to monitor
multidrug-resistant-tuberculosis (MDR-TB) patients during treatment? J Clin
Microbiol. 2013 Feb;51(2):644-6. doi: 10.1128/JCM.02837-12. Epub 2012 Dec 5.
PubMed PMID: 23224098; PubMed Central PMCID: PMC3553933.

492: Franke MF, Appleton SC, Mitnick CD, Furin JJ, Bayona J, Chalco K, Shin S,
Murray M, Becerra MC. Aggressive regimens for multidrug-resistant tuberculosis
reduce recurrence. Clin Infect Dis. 2013 Mar;56(6):770-6. doi:
10.1093/cid/cis1008. Epub 2012 Dec 7. PubMed PMID: 23223591; PubMed Central
PMCID: PMC3582355.

493: Denkinger CM, Pai M, Patel M, Menzies D. Gamma interferon release assay for
monitoring of treatment response for active tuberculosis: an explosion in the
spaghetti factory. J Clin Microbiol. 2013 Feb;51(2):607-10. doi:
10.1128/JCM.02278-12. Epub 2012 Nov 21. PubMed PMID: 23175268; PubMed Central
PMCID: PMC3553895.

494: Baumann R, Kaempfer S, Chegou NN, Nene NF, Veenstra H, Spallek R, Bolliger
CT, Lukey PT, van Helden PD, Singh M, Walzl G. Serodiagnostic markers for the

prediction of the outcome of intensive phase tuberculosis therapy. Tuberculosis
(Edinb). 2013 Mar;93(2):239-45. doi: 10.1016/j.tube.2012.09.003. Epub 2012 Nov 2.
PubMed PMID: 23127778.

495: Lee J, Lee BJ, Yoon HI, Lee CT, Lee JH. Influence of previous tuberculosis
treatment history on acid-fast bacilli smear and culture conversion. Int J Tuberc
Lung Dis. 2012 Oct;16(10):1344-8. doi: 10.5588/ijtld.12.0113. PubMed PMID:
23107634.

496: Kurbatova EV, Gammino VM, Bayona J, Becerra MC, Danilovitz M, Falzon D,
Gelmanova I, Keshavjee S, Leimane V, Mitnick CD, Quelapio MI, Riekstina V, Taylor
A, Viiklepp P, Zignol M, Cegielski JP. Predictors of sputum culture conversion
among patients treated for multidrug-resistant tuberculosis. Int J Tuberc Lung
Dis. 2012 Oct;16(10):1335-43. doi: 10.5588/ijtld.11.0811. PubMed PMID: 23107633.

497: Ruslami R, Ganiem AR, Dian S, Apriani L, Achmad TH, van der Ven AJ, Borm G,
Aarnoutse RE, van Crevel R. Intensified regimen containing rifampicin and
moxifloxacin for tuberculous meningitis: an open-label, randomised controlled
phase 2 trial. Lancet Infect Dis. 2013 Jan;13(1):27-35. doi:
10.1016/S1473-3099(12)70264-5. Epub 2012 Oct 25. PubMed PMID: 23103177.

498: Sanwalka N, Khadilkar A, Chiplonkar S, Khatod K, Phadke N, Khadilkar V.
Vitamin D receptor gene polymorphisms and bone mass indices in post-menarchal
Indian adolescent girls. J Bone Miner Metab. 2013 Jan;31(1):108-15. doi:
10.1007/s00774-012-0390-0. Epub 2012 Oct 19. PubMed PMID: 23081732.

499: Lee M, Lee J, Carroll MW, Choi H, Min S, Song T, Via LE, Goldfeder LC, Kang
E, Jin B, Park H, Kwak H, Kim H, Jeon HS, Jeong I, Joh JS, Chen RY, Olivier KN,
Shaw PA, Follmann D, Song SD, Lee JK, Lee D, Kim CT, Dartois V, Park SK, Cho SN,
Barry CE 3rd. Linezolid for treatment of chronic extensively drug-resistant

tuberculosis. N Engl J Med. 2012 Oct 18;367(16):1508-18. doi:
10.1056/NEJMoa1201964. PubMed PMID: 23075177; PubMed Central PMCID:
PMC3814175.

500: Marx FM, Dunbar R, Enarson DA, Beyers N. The rate of sputum
smear-positive
tuberculosis after treatment default in a high-burden setting: a
retrospective
cohort study. PLoS One. 2012;7(9):e45724. doi:
10.1371/journal.pone.0045724. Epub
2012 Sep 25. Erratum in: PLoS One. 2013;8(8). doi:
10.1371/annotation/ea5f2a13-4394-41af-84c8-3e6af4a07770. PubMed
PMID: 23049846;
PubMed Central PMCID: PMC3458061.

501: Singh UB, Rana T, Kaushik A, Porwal C, Makkar N. Day zero
quantitative mRNA
analysis as a prognostic marker in pulmonary tuberculosis category
II patients on
treatment. Clin Microbiol Infect. 2012 Nov;18(11):E473-81. doi:
10.1111/j.1469-0691.2012.04004.x. Epub 2012 Sep 25. PubMed PMID:
23005345.

502: Rathored J, Sharma SK, Singh B, Banavaliker JN, Sreenivas V,
Srivastava AK,
Mohan A, Sachan A, Harinarayan CV, Goswami R. Risk and outcome of
multidrug-resistant tuberculosis: vitamin D receptor polymorphisms
and serum
25(OH)D. Int J Tuberc Lung Dis. 2012 Nov;16(11):1522-8. doi:
10.5588/ijtld.12.0122. Epub 2012 Sep 14. PubMed PMID: 22990231.

503: Coussens AK, Wilkinson RJ, Hanifa Y, Nikolayevskyy V, Elkington
PT, Islam K,
Timms PM, Venton TR, Bothamley GH, Packe GE, Darmalingam M, Davidson
RN, Milburn
HJ, Baker LV, Barker RD, Mein CA, Bhaw-Rosun L, Nuamah R, Young DB,
Drobniewski
FA, Griffiths CJ, Martineau AR. Vitamin D accelerates resolution of
inflammatory
responses during tuberculosis treatment. Proc Natl Acad Sci U S A.
2012 Sep
18;109(38):15449-54. Epub 2012 Sep 4. PubMed PMID: 22949664; PubMed
Central
PMCID: PMC3458393.

504: Naidoo K, Yende-Zuma N, Padayatchi N, Naidoo K, Jithoo N, Nair
G, Bamber S,
Gengiah S, El-Sadr WM, Friedland G, Abdool Karim S. The immune
reconstitution
inflammatory syndrome after antiretroviral therapy initiation in
patients with

tuberculosis: findings from the SAPiT trial. Ann Intern Med. 2012 Sep
4;157(5):313-24. doi: 10.7326/0003-4819-157-5-201209040-00004. PubMed PMID:
22944873; PubMed Central PMCID: PMC3534856.

505: Kerstjens HA, Engel M, Dahl R, Paggiaro P, Beck E, Vandewalker M, Sigmund R,
Seibold W, Moroni-Zentgraf P, Bateman ED. Tiotropium in asthma poorly controlled
with standard combination therapy. N Engl J Med. 2012 Sep 27;367(13):1198-207.
Epub 2012 Sep 2. PubMed PMID: 22938706.

506: Harada T, Akiyama Y, Kurashima A, Nagai H, Tsuyuguchi K, Fujii T, Yano S,
Shigeto E, Kuraoka T, Kajiki A, Kobashi Y, Kokubu F, Sato A, Yoshida S, Iwamoto
T, Saito H. Clinical and microbiological differences between Mycobacterium
abscessus and Mycobacterium massiliense lung diseases. J Clin Microbiol. 2012
Nov;50(11):3556-61. doi: 10.1128/JCM.01175-12. Epub 2012 Aug 22. Erratum in: J
Clin Microbiol. 2013 Feb;51(2):736. J Clin Microbiol. 2013 Mar;51(3)1061. PubMed
PMID: 22915613; PubMed Central PMCID: PMC3486228.

507: Shneider BL, Magee JC, Bezerra JA, Haber B, Karpen SJ, Raghunathan T,
Rosenthal P, Schwarz K, Suchy FJ, Kerkar N, Turmelle Y, Whitington PF, Robuck PR,
Sokol RJ; Childhood Liver Disease Research Education Network (ChiLDREN). Efficacy
of fat-soluble vitamin supplementation in infants with biliary atresia.
Pediatrics. 2012 Sep;130(3):e607-14. Epub 2012 Aug 13. PubMed PMID: 22891232;
PubMed Central PMCID: PMC3428752.

508: Yang HZ, Wang K, Jin HZ, Gao TW, Xiao SX, Xu JH, Wang BX, Zhang FR, Li CY,
Liu XM, Tu CX, Ji SZ, Shen Y, Zhu XJ. Infliximab monotherapy for Chinese patients
with moderate to severe plaque psoriasis: a randomized, double-blind,
placebo-controlled multicenter trial. Chin Med J (Engl). 2012
Jun;125(11):1845-51. PubMed PMID: 22884040.

509: van Vollenhoven RF, Fleischmann R, Cohen S, Lee EB, GarcÃ-a Meijide JA,

Wagner S, Forejtova S, Zwillich SH, Gruben D, Koncz T, Wallenstein GV,
Krishnaswami S, Bradley JD, Wilkinson B; ORAL Standard Investigators. Tofacitinib
or adalimumab versus placebo in rheumatoid arthritis. N Engl J Med. 2012 Aug
9;367(6):508-19. doi: 10.1056/NEJMoa1112072. Erratum in: N Engl J Med. 2013 Jul
18;369(3):293. PubMed PMID: 22873531.

510: Chang KC, Leung CC, Yew WW, Leung EC, Leung WM, Tam CM, Zhang Y.
Pyrazinamide may improve fluoroquinolone-based treatment of multidrug-resistant
tuberculosis. Antimicrob Agents Chemother. 2012 Nov;56(11):5465-75. doi:
10.1128/AAC.01300-12. Epub 2012 Aug 6. PubMed PMID: 22869570; PubMed Central
PMCID: PMC3486566.

511: Butov DA, Efremenko YV, Prihoda ND, Yurchenko LI, Sokolenko NI, Arjanova OV,
Stepanenko AL, Butova TS, Zaitzeva SS, Jirathitikal V, Bourinbaiar AS, Kutsyna
GA. Adjunct immune therapy of first-diagnosed TB, relapsed TB, treatment-failed
TB, multidrug-resistant TB and TB/HIV. Immunotherapy. 2012 Jul;4(7):687-95. doi:
10.2217/imt.12.59. PubMed PMID: 22853755.

512: Demidik SN, Sukhanov DS. [Cycloferon in the complex therapy of patients with
widespread forms of pulmonary tuberculosis]. Eksp Klin Farmakol.
2012;75(5):17-20. Russian. PubMed PMID: 22834123.

513: Kumar SR, Gopalan N, Patrawalla P, Menon P, Mayer K, Swaminathan S. Immune
reconstitution inflammatory syndrome in HIV-infected patients with and without
prior tuberculosis. Int J STD AIDS. 2012 Jun;23(6):419-23. doi:
10.1258/ijsa.2009.009439. PubMed PMID: 22807536; PubMed Central PMCID:
PMC5592838.

514: Kayigamba FR, Bakker MI, Mugisha V, Gasana M, Schim van der Loeff MF. Sputum
completion and conversion rates after intensive phase of tuberculosis treatment:
an assessment of the Rwandan control program. BMC Res Notes. 2012 Jul 16;5:357.

doi: 10.1186/1756-0500-5-357. PubMed PMID: 22800438; PubMed Central PMCID:
PMC3413528.

515: Chakrabarti S, Saha I, Das DK, Prasad Sarkar A, Roy R, Hossain A.
Comparative study of the profiles of tribal and non-tribal tuberculosis patients
in a tuberculosis unit of West Bengal, India. Int J Tuberc Lung Dis. 2012
Sep;16(9):1205-9. doi: 10.5588/ijtld.11.0501. Epub 2012 Jul 12. PubMed PMID:
22793518.

516: Hsia EC, Cush JJ, Matteson EL, Beutler A, Doyle MK, Hsu B, Xu S, Rahman MU.
Comprehensive tuberculosis screening program in patients with inflammatory
arthritides treated with golimumab, a human anti-tumor necrosis factor antibody,
in Phase III clinical trials. Arthritis Care Res (Hoboken). 2013
Feb;65(2):309-13. doi: 10.1002/acr.21788. PubMed PMID: 22782640.

517: Nagaraja C, Shashibhushan BL, Asif M, Manjunath PH, Sagar C. Pattern of
drug-resistance and treatment outcome in multidrug-resistant pulmonary
tuberculosis. Indian J Chest Dis Allied Sci. 2012 Jan-Mar;54(1):23-6. PubMed
PMID: 22779119.

518: Ganmaa D, Giovannucci E, Bloom BR, Fawzi W, Burr W, Batbaatar D, Sumberzul
N, Holick MF, Willett WC. Vitamin D, tuberculin skin test conversion, and latent
tuberculosis in Mongolian school-age children: a randomized, double-blind,
placebo-controlled feasibility trial. Am J Clin Nutr. 2012 Aug;96(2):391-6. doi:
10.3945/ajcn.112.034967. Epub 2012 Jul 3. PubMed PMID: 22760564; PubMed Central
PMCID: PMC3396446.

519: Van Deun A, Maug AK, Hossain A, Gumusboga M, de Jong BC. Fluorescein
diacetate vital staining allows earlier diagnosis of rifampicin-resistant
tuberculosis. Int J Tuberc Lung Dis. 2012 Sep;16(9):1174-9. doi:
10.5588/ijtld.11.0166. Epub 2012 Jun 28. PubMed PMID: 22747903.

520: Feng JY, Huang SF, Ting WY, Chen YC, Lin YY, Huang RM, Lin CH, Hwang JJ, Lee
JJ, Yu MC, Yu KW, Lee YC, Su WJ. Gender differences in treatment outcomes of
tuberculosis patients in Taiwan: a prospective observational study. Clin
Microbiol Infect. 2012 Sep;18(9):E331-7. doi: 10.1111/j.1469-0691.2012.03931.x.
Epub 2012 Jun 27. PubMed PMID: 22734962.

521: Meintjes G, Skolimowska KH, Wilkinson KA, Matthews K, Tadokera R,
Conesa-Botella A, Seldon R, Rangaka MX, Rebe K, Pepper DJ, Morroni C, Colebunders
R, Maartens G, Wilkinson RJ. Corticosteroid-modulated immune activation in the
tuberculosis immune reconstitution inflammatory syndrome. Am J Respir Crit Care
Med. 2012 Aug 15;186(4):369-77. doi: 10.1164/rccm.201201-0094OC. Epub 2012 Jun
14. PubMed PMID: 22700860; PubMed Central PMCID: PMC3443811.

522: Gler MT, Skripconoka V, Sanchez-Garavito E, Xiao H, Cabrera-Rivero JL,
Vargas-Vasquez DE, Gao M, Awad M, Park SK, Shim TS, Suh GY, Danilovits M, Ogata
H, Kurve A, Chang J, Suzuki K, Tupasi T, Koh WJ, Seaworth B, Geiter LJ, Wells CD.
Delamanid for multidrug-resistant pulmonary tuberculosis. N Engl J Med. 2012 Jun
7;366(23):2151-60. doi: 10.1056/NEJMoa1112433. PubMed PMID: 22670901.

523: Bhatt G, Vyas S, Trivedil K. An epidemiological study of multi drug
resistant tuberculosis cases registered under Revised National Tuberculosis
Control Programme of Ahmedabad City. Indian J Tuberc. 2012 Jan;59(1):18-27.
PubMed PMID: 22670507.

524: Kakchapati S, Gyawali BN, Jha RK, Choonpradub C. Treatment outcome of
multidrug-resistant Mycobacterium tuberculosis in Nepal. Asia Pac J Public
Health. 2012 Jul;24(4):631-40. doi: 10.1177/1010539511408067. Epub 2012 May 31.
PubMed PMID: 22652251.

525: Chaudhry LA, Essa EB, Al-Solaiman S, Al-Sindi K. Prevalence of diabetes

type-2 & pulmonary tuberculosis among Filipino and treatment outcomes: A
surveillance study in the Eastern Saudi Arabia. Int J Mycobacteriol. 2012
Jun;1(2):106-9. doi: 10.1016/j.ijmyco.2012.03.002. Epub 2012 May 4. PubMed PMID:
26787066.

526: Chaudhry LA, Zamzami M, Aldin S, Pazdirek J. Clinical consequences of
non-compliance with directly observed therapy short course (DOTS): Story of a
recurrent defaulter. Int J Mycobacteriol. 2012 Jun;1(2):99-103. doi:
10.1016/j.ijmyco.2012.05.003. Epub 2012 Jun 21. PubMed PMID:
26787064.

527: Xu HB, Jiang RH, Li L, Xiao HP. Linezolid in the treatment of MDR-TB: a
retrospective clinical study. Int J Tuberc Lung Dis. 2012;16(3):358-63. doi:
10.5588/ijtld.11.0493. PubMed PMID: 22640450.

528: Iddriss A, Padayatchi N, Reddy D, Reddi A. Pulmonary resection for
extensively drug resistant tuberculosis in Kwazulu-Natal, South Africa. Ann
Thorac Surg. 2012 Aug;94(2):381-6. doi: 10.1016/j.athoracsur.2012.03.072. Epub
2012 May 24. PubMed PMID: 22633500; PubMed Central PMCID: PMC3567439.

529: Theron G, Peter J, Lenders L, van Zyl-Smit R, Meldau R, Govender U, Dheda K.
Correlation of mycobacterium tuberculosis specific and non-specific quantitative
Th1 T-cell responses with bacillary load in a high burden setting. PLoS One.
2012;7(5):e37436. doi: 10.1371/journal.pone.0037436. Epub 2012 May 22. PubMed
PMID: 22629395; PubMed Central PMCID: PMC3358317.

530: Chotirmall SH, Smith SG, Gunaratnam C, Cosgrove S, Dimitrov BD, O'Neill SJ,
Harvey BJ, Greene CM, McElvaney NG. Effect of estrogen on pseudomonas mucoidy and
exacerbations in cystic fibrosis. N Engl J Med. 2012 May 24;366(21):1978-86. doi:
10.1056/NEJMoa1106126. Epub 2012 May 20. PubMed PMID: 22607135.

531: Riou C, Perez Peixoto B, Roberts L, Ronacher K, Walzl G, Manca C, Rustomjee
R, Mthiyane T, Fallows D, Gray CM, Kaplan G. Effect of standard tuberculosis
treatment on plasma cytokine levels in patients with active pulmonary
tuberculosis. PLoS One. 2012;7(5):e36886. doi: 10.1371/journal.pone.0036886. Epub
2012 May 14. PubMed PMID: 22606304; PubMed Central PMCID: PMC3351475.

532: Manosuthi W, Mankatitham W, Lueangniyomkul A, Thongyen S, Likanonsakul S,
Suwanvattana P, Thawornwan U, Suntisuklappon B, Nilkamhang S, Sungkanuparph S;
TIME Study Team. Time to initiate antiretroviral therapy between 4 weeks and 12
weeks of tuberculosis treatment in HIV-infected patients: results from the TIME
study. J Acquir Immune Defic Syndr. 2012 Aug 1;60(4):377-83. doi:
10.1097/QAI.0b013e31825b5e06. PubMed PMID: 22592586.

533: Sotgiu G, Centis R, D'Ambrosio L, Alffenaar JW, Anger HA, Caminero JA,
Castiglia P, De Lorenzo S, Ferrara G, Koh WJ, Schecter GF, Shim TS, Singla R,
Skrahina A, Spanevello A, Udwadia ZF, Villar M, Zampogna E, Zellweger JP, Zumla
A, Migliori GB. Efficacy, safety and tolerability of linezolid containing
regimens in treating MDR-TB and XDR-TB: systematic review and meta-analysis. Eur
Respir J. 2012 Dec;40(6):1430-42. doi: 10.1183/09031936.00022912. Epub 2012 Apr
10. Review. PubMed PMID: 22496332.

534: Pasipanodya JG, Srivastava S, Gumbo T. Meta-analysis of clinical studies
supports the pharmacokinetic variability hypothesis for acquired drug resistance
and failure of antituberculosis therapy. Clin Infect Dis. 2012 Jul;55(2):169-77.
doi: 10.1093/cid/cis353. Epub 2012 Mar 30. PubMed PMID: 22467670; PubMed Central
PMCID: PMC3491771.

535: Gegia M, Kalandadze I, Kempker RR, Magee MJ, Blumberg HM. Adjunctive surgery
improves treatment outcomes among patients with multidrug-resistant and
extensively drug-resistant tuberculosis. Int J Infect Dis. 2012 May;16(5):e391-6.

doi: 10.1016/j.ijid.2011.12.018. Epub 2012 Mar 17. PubMed PMID: 22425494; PubMed
Central PMCID: PMC3786004.

536: Didilescu C, Craiova UM. [Present and future in the use of anti-tubercular
drugs]. Pneumologia. 2011 Oct-Dec;60(4):198-201. Romanian. PubMed PMID: 22420168.

537: Goodridge A, Cueva C, Lahiff M, Muzanye G, Johnson JL, Nahid P, Riley LW.
Anti-phospholipid antibody levels as biomarker for monitoring tuberculosis
treatment response. Tuberculosis (Edinb). 2012 May;92(3):243-7. doi:
10.1016/j.tube.2012.02.004. Epub 2012 Mar 10. PubMed PMID: 22406155; PubMed
Central PMCID: PMC4408545.

538: Efremenko YV, Arjanova OV, Prihoda ND, Yurchenko LV, Sokolenko NI, Mospan
IV, Pylypchuk VS, Rowe J, Jirathitikal V, Bourinbaiar AS, Kutsyna GA. Clinical
validation of sublingual formulations of Immunoxel (Dzherelo) as an adjuvant
immunotherapy in treatment of TB patients. Immunotherapy. 2012 Mar;4(3):273-82.
doi: 10.2217/imt.11.176. PubMed PMID: 22401633.

539: Diacon AH, Donald PR, Pym A, Grobusch M, Patientia RF, Mahanyele R,
Bantubani N, Narasimooloo R, De Marez T, van Heeswijk R, Lounis N, Meyvisch P,
Andries K, McNeeley DF. Randomized pilot trial of eight weeks of bedaquiline
(TMC207) treatment for multidrug-resistant tuberculosis: long-term outcome,
tolerability, and effect on emergence of drug resistance. Antimicrob Agents
Chemother. 2012 Jun;56(6):3271-6. doi: 10.1128/AAC.06126-11. Epub 2012 Mar 5.
PubMed PMID: 22391540; PubMed Central PMCID: PMC3370813.

540: Kim HS, Lee KS, Koh WJ, Jeon K, Lee EJ, Kang H, Ahn J. Serial CT findings of
Mycobacterium massiliense pulmonary disease compared with Mycobacterium abscessus
disease after treatment with antibiotic therapy. Radiology. 2012
Apr;263(1):260-70. doi: 10.1148/radiol.12111374. Epub 2012 Feb 27. PubMed PMID:
22371609.

541: Mordant P, Badia A, Le Pimpec-Barthes F, Riquet M. [Thoracic surgery in
tuberculosis and non-tuberculous mycobacterial diseases]. Rev Pneumol Clin. 2012
Apr;68(2):77-83. doi: 10.1016/j.pneumo.2012.01.003. Epub 2012 Feb 22. Review.
French. PubMed PMID: 22361064.

542: Kunawararak P, Pongpanich S, Chantawong S, Pokaew P, Traisathit P,
Srithanaviboonchai K, Plipat T. Tuberculosis treatment with mobile-phone
medication reminders in northern Thailand. Southeast Asian J Trop Med Public
Health. 2011 Nov;42(6):1444-51. PubMed PMID: 22299414.

543: Gammino VM, Taylor AB, Rich ML, Bayona J, Becerra MC, Bonilla C, Gelmanova
I, Hollo V, Jaramillo E, Keshavjee S, Leimane V, Mitnick CD, Quelapio MI,
Riektsina V, Tupasi TE, Wells CD, Zignol M, Cegielski PJ. Bacteriologic
monitoring of multidrug-resistant tuberculosis patients in five DOTS-Plus pilot
projects. Int J Tuberc Lung Dis. 2011 Oct;15(10):1315-22. doi:
10.5588/ijtld.10.0221. PubMed PMID: 22283887.

544: Caetano Mota P, Carvalho A, Valente I, Braga R, Duarte R. Predictors of
delayed sputum smear and culture conversion among a Portuguese population with
pulmonary tuberculosis. Rev Port Pneumol. 2012 Mar-Apr;18(2):72-9. doi:
10.1016/j.rppneu.2011.12.005. Epub 2012 Jan 25. English, Portuguese. PubMed PMID:
22277838.

545: Okumura M, Yoshiyama T, Ogata H, Morimoto K, Kokuto H, Kurashima A, Kudoh S.
[Factors related to the occurrence of multi- (extensively-) drug resistant
tuberculosis (M/XDR-TB) in our hospital]. Kekkaku. 2011 Nov;86(11):863-8.
Japanese. PubMed PMID: 22250465.

546: Visser ME, Stead MC, Walzl G, Warren R, Schomaker M, Grewal HM, Swart EC,
Maartens G. Baseline predictors of sputum culture conversion in pulmonary

tuberculosis: importance of cavities, smoking, time to detection and W-Beijing
genotype. PLoS One. 2012;7(1):e29588. doi: 10.1371/journal.pone.0029588. Epub
2012 Jan 4. PubMed PMID: 22238625; PubMed Central PMCID: PMC3251579.

547: Hsia EC, Schluger N, Cush JJ, Chaisson RE, Matteson EL, Xu S, Beutler A,
Doyle MK, Hsu B, Rahman MU. Interferon-Î³ release assay versus tuberculin skin
test prior to treatment with golimumab, a human anti-tumor necrosis factor
antibody, in patients with rheumatoid arthritis, psoriatic arthritis, or
ankylosing spondylitis. Arthritis Rheum. 2012 Jul;64(7):2068-77. doi:
10.1002/art.34382. PubMed PMID: 22238071.

548: Ito Y, Hirai T, Maekawa K, Fujita K, Imai S, Tatsumi S, Handa T, Matsumoto
H, Muro S, Niimi A, Mishima M. Predictors of 5-year mortality in pulmonary
Mycobacterium avium-intracellulare complex disease. Int J Tuberc Lung Dis.
2012;16(3):408-14. doi: 10.5588/ijtld.11.0148. Epub 2012 Jan 5. PubMed PMID:
22230733.

549: Kim SH, Park C, Kwon EY, Shin NY, Kwon JC, Park SH, Choi SM, Lee DG, Choi
JH, Yoo JH. Real-time nucleic acid sequence-based amplification to predict the
clinical outcome of invasive aspergillosis. J Korean Med Sci. 2012
Jan;27(1):10-5. doi: 10.3346/jkms.2012.27.1.10. Epub 2011 Dec 19. PubMed PMID:
22219607; PubMed Central PMCID: PMC3247764.

550: Kobashi Y, Abe M, Mouri K, Obase Y, Kato S, Oka M. Relationship between
clinical efficacy for pulmonary MAC and drug-sensitivity test for isolated MAC in
a recent 6-year period. J Infect Chemother. 2012 Aug;18(4):436-43. doi:
10.1007/s10156-011-0351-x. Epub 2011 Dec 17. PubMed PMID: 22205543.

551: Xu HB, Jiang RH, Xiao HP. Clofazimine in the treatment of
multidrug-resistant tuberculosis. Clin Microbiol Infect. 2012 Nov;18(11):1104-10.
doi: 10.1111/j.1469-0691.2011.03716.x. Epub 2011 Dec 22. PubMed PMID: 22192631.

552: TÃ¶rÃ¶k ME, Nguyen DB, Tran TH, Nguyen TB, Thwaites GE, Hoang TQ, Nguyen HD,
Tran TH, Nguyen TC, Hoang HT, Wolbers M, Farrar JJ. Dexamethasone and long-term
outcome of tuberculous meningitis in Vietnamese adults and adolescents. PLoS One.
2011;6(12):e27821. doi: 10.1371/journal.pone.0027821. Epub 2011 Dec 8. PubMed
PMID: 22174748; PubMed Central PMCID: PMC3234244.

553: Arnedo-Pena A, Juan-CerdÃ¡n JV, Romeu-Garcia A, Garcia-Ferrer D,
HolguÃ-n-GÃ³mez R, Iborra-Millet J, Herrero-Carot C, PiÃ±ana MJ, Bellido-Blasco J,
Ferrero-Vega JA, Adsuara LS, Silvestre ES, Ferrer NM, Bartual VR. Latent
tuberculosis infection, tuberculin skin test and vitamin D status in contacts of
tuberculosis patients: a cross-sectional and case-control study. BMC Infect Dis.
2011 Dec 15;11:349. doi: 10.1186/1471-2334-11-349. PubMed PMID: 22171844; PubMed
Central PMCID: PMC3292546.

554: Hyams J, Damaraju L, Blank M, Johanns J, Guzzo C, Winter HS, Kugathasan S,
Cohen S, Markowitz J, Escher JC, Veereman-Wauters G, Crandall W, Baldassano R,
Griffiths A; T72 Study Group. Induction and maintenance therapy with infliximab
for children with moderate to severe ulcerative colitis. Clin Gastroenterol
Hepatol. 2012 Apr;10(4):391-9.e1. doi: 10.1016/j.cgh.2011.11.026. Epub 2011 Dec
8. PubMed PMID: 22155755.

555: Tangg SJ, Zhang Q, Zheng LH, Sun H, Gu J, Hao XH, Liu YD, Yao L, Xiao HP.
Efficacy and safety of linezolid in the treatment of extensively drug-resistant
tuberculosis. Jpn J Infect Dis. 2011;64(6):509-12. PubMed PMID: 22116331.

556: Kunogi M, Kawabe Y, Suzuki J, Shimada M, Kaneko Y, Matsui Y, Kawashima M,
Ohshima N, Ariga H, Masuda K, Matsui H, Tamura A, Nagai H, Akagawa S, Nagayama N,
Toyota E, Machida K, Nakajima Y. [Evaluation of tuberculosis treatment including
levofloxacin (LVFX) in cases who could not continue standard regimen]. Kekkaku.

2011 Sep;86(9):773-9. Japanese. PubMed PMID: 22111385.

557: Bucci FA Jr, Waterbury LD. Prostaglandin E2 inhibition of ketorolac 0.45%,
bromfenac 0.09%, and nepafenac 0.1% in patients undergoing phacoemulsification.
Adv Ther. 2011 Dec;28(12):1089-95. doi: 10.1007/s12325-011-0080-7. Epub 2011 Nov
18. PubMed PMID: 22105509.

558: Radovic M, Ristic L, Stankovic I, Pejcic T, Rancic M, Ciric Z, Dinic-Radovic
V. Chronic airflow obstruction syndrome due to pulmonary tuberculosis treated
with directly observed therapy--a serious changes in lung function. Med Arh.
2011;65(5):265-9. PubMed PMID: 22073848.

559: Shiobara Y, Goto H, Hoshino M, Tsukahara T, Yamaguchi N, Ito M, Nozawa A,
Tsukiji J, Ishigatsubo Y, Kaneko T. [Two cases of juvenile-onset and adult-onset
recurrent respiratory papillomatosis]. Nihon Kokyuki Gakkai Zasshi. 2011
Sep;49(9):667-73. Japanese. PubMed PMID: 22073613.

560: Tao Y, Kitasato Y, Kawasaki M, Oe T, Kitahara Y, Kawakami K, Takikawa S,
Miyagi S, Yamanaka T, Toshihiko I, Kawabata M. [Clinical investigation of
multidrug-resistant tuberculosis --investigation of inpatients in the Kyushu
region between 2004 and 2009]. Kekkaku. 2011 Aug;86(8):751-5. Japanese. PubMed
PMID: 22073593.

561: Seddon JA, Hesseling AC, Willemse M, Donald PR, Schaaf HS. Culture-confirmed
multidrug-resistant tuberculosis in children: clinical features, treatment, and
outcome. Clin Infect Dis. 2012 Jan 15;54(2):157-66. doi: 10.1093/cid/cir772. Epub
2011 Nov 3. PubMed PMID: 22052896.

562: Singla N, Gupta D, Joshi A, Batra N, Singh J, Birbian N. Association of
mannose-binding lectin gene polymorphism with tuberculosis susceptibility and
sputum conversion time. Int J Immunogenet. 2012 Feb;39(1):10-4. doi:

10.1111/j.1744-313X.2011.01047.x. Epub 2011 Nov 3. PubMed PMID:
22050925.

563: Park SW, Shin JW, Kim JY, Park IW, Choi BW, Choi JC, Kim YS.
The effect of
diabetic control status on the clinical features of pulmonary
tuberculosis. Eur J
Clin Microbiol Infect Dis. 2012 Jul;31(7):1305-10. doi:
10.1007/s10096-011-1443-3. Epub 2011 Oct 25. PubMed PMID: 22042559.

564: Oliveira HB, Mateus SH. [Characterization of multidrug-
resistant
tuberculosis during pregnancy in Campinas, State of SÃ£o Paulo,
Brazil, from 1995
to 2007]. Rev Soc Bras Med Trop. 2011 Oct;44(5):627-30. Portuguese.
PubMed PMID:
22031080.

565: Christopher DJ, James P, Daley P, Armstrong L, Isaac BT,
Thangakunam B,
Premkumar B, Zwerling A, Pai M. High annual risk of tuberculosis
infection among
nursing students in South India: a cohort study. PLoS One.
2011;6(10):e26199.
doi: 10.1371/journal.pone.0026199. Epub 2011 Oct 12. PubMed PMID:
22022565;
PubMed Central PMCID: PMC3192164.

566: Abdool Karim SS, Naidoo K, Grobler A, Padayatchi N, Baxter C,
Gray AL,
Gengiah T, Gengiah S, Naidoo A, Jithoo N, Nair G, El-Sadr WM,
Friedland G, Abdool
Karim Q. Integration of antiretroviral therapy with tuberculosis
treatment. N
Engl J Med. 2011 Oct 20;365(16):1492-501. doi:
10.1056/NEJMoa1014181. PubMed
PMID: 22010915; PubMed Central PMCID: PMC3233684.

567: Havlir DV, Kendall MA, Ive P, Kumwenda J, Swindells S, Qasba
SS, Luetkemeyer
AF, Hogg E, Rooney JF, Wu X, Hosseinipour MC, Lalloo U, Veloso VG,
Some FF,
Kumarasamy N, Padayatchi N, Santos BR, Reid S, Hakim J, Mohapi L,
Mugyenyi P,
Sanchez J, Lama JR, Pape JW, Sanchez A, Asmelash A, Moko E, Sawe F,
Andersen J,
Sanne I; AIDS Clinical Trials Group Study A5221. Timing of
antiretroviral therapy
for HIV-1 infection and tuberculosis. N Engl J Med. 2011 Oct
20;365(16):1482-91.

doi: 10.1056/NEJMoa1013607. PubMed PMID: 22010914; PubMed Central PMCID:
PMC3327101.

568: Blanc FX, Sok T, Laureillard D, Borand L, Rekacewicz C, Nerrienet E, Madec
Y, Marcy O, Chan S, Prak N, Kim C, Lak KK, Hak C, Dim B, Sin CI, Sun S, Guillard
B, Sar B, Vong S, Fernandez M, Fox L, Delfraissy JF, Goldfeld AE; CAMELIA (ANRS
1295â€"CIPRA KH001) Study Team. Earlier versus later start of antiretroviral
therapy in HIV-infected adults with tuberculosis. N Engl J Med. 2011 Oct
20;365(16):1471-81. doi: 10.1056/NEJMoa1013911. PubMed PMID: 22010913; PubMed
Central PMCID: PMC4879711.

569: Qazi F, Khan U, Khowaja S, Javaid M, Ahmed A, Salahuddin N, Hussain H,
Becerra MC, Golub JE, Khan AJ. Predictors of delayed culture conversion in
patients treated for multidrug-resistant tuberculosis in Pakistan. Int J Tuberc
Lung Dis. 2011 Nov;15(11):1556-9, i. doi: 10.5588/ijtld.10.0679. PubMed PMID:
22008773; PubMed Central PMCID: PMC6203961.

570: Kurbatova EV, Gammino VM, Bayona J, Becerra M, Danilovitz M, Falzon D,
Gelmanova I, Keshavjee S, Leimane V, Mitnick CD, Quelapio MI, Riekstina V, Taylor
A, Viiklepp P, Zignol M, Cegielski JP. Frequency and type of microbiological
monitoring of multidrug-resistant tuberculosis treatment. Int J Tuberc Lung Dis.
2011 Nov;15(11):1553-5, i. doi: 10.5588/ijtld.11.0101. PubMed PMID: 22008772.

571: Skenders GK, Holtz TH, Riekstina V, Leimane V. Implementation of the
INNO-LiPA Rif. TBÂ® line-probe assay in rapid detection of multidrug-resistant
tuberculosis in Latvia. Int J Tuberc Lung Dis. 2011 Nov;15(11):1546-52, i. doi:
10.5588/ijtld.11.0067. PubMed PMID: 22008771.

572: Bucci FA Jr, Waterbury LD. A randomized comparison of to-aqueous penetration
of ketorolac 0.45%, bromfenac 0.09% and nepafenac 0.1% in cataract patients

undergoing phacoemulsification. Curr Med Res Opin. 2011
Dec;27(12):2235-9. doi:
10.1185/03007995.2011.626018. Epub 2011 Oct 12. PubMed PMID:
21992076.

573: Mukhopadhyay S, Sarkar AP. Comparative Analysis of RNTCP Indicators in a
Rural and an Urban Tuberculosis Unit of Burdwan District in West Bengal. Indian J
Community Med. 2011 Apr;36(2):146-9. doi: 10.4103/0970-0218.84136. PubMed PMID:
21976802; PubMed Central PMCID: PMC3180942.

574: Jung KS, Park HY, Park SY, Kim SK, Kim YK, Shim JJ, Moon HS, Lee KH, Yoo JH,
Lee SD; Korean Academy of Tuberculosis and Respiratory Diseases study group;
Korea Chronic Obstructive Pulmonary Disease study group. Comparison of tiotropium
plus fluticasone propionate/salmeterol with tiotropium in COPD: a randomized
controlled study. Respir Med. 2012 Mar;106(3):382-9. doi:
10.1016/j.rmed.2011.09.004. Epub 2011 Oct 4. PubMed PMID: 21975275.

575: Singla R, Caminero JA, Jaiswal A, Singla N, Gupta S, Bali RK, Behera D.
Linezolid: an effective, safe and cheap drug for patients failing
multidrug-resistant tuberculosis treatment in India. Eur Respir J. 2012
Apr;39(4):956-62. doi: 10.1183/09031936.00076811. Epub 2011 Sep 29. PubMed PMID:
21965225.

576: Vadwai V, Daver G, Udwadia Z, Sadani M, Shetty A, Rodrigues C. Clonal
population of Mycobacterium tuberculosis strains reside within multiple lung
cavities. PLoS One. 2011;6(9):e24770. doi: 10.1371/journal.pone.0024770. Epub
2011 Sep 14. PubMed PMID: 21935462; PubMed Central PMCID: PMC3173478.

577: Luzzati R, Confalonieri M, Cazzadori A, Della Loggia P, Cifaldi R, Fabris C,
Biolo M, Borelli M, Longo C, Concia E. Prolonged hospitalisation for immigrants
and high risk patients with positive smear pulmonary tuberculosis. Monaldi Arch
Chest Dis. 2011 Jun;75(2):141-5. PubMed PMID: 21932701.

578: Lebwohl M, Leonardi C, Griffiths CE, Prinz JC, Szapary PO, Yeilding N, Guzzo
C, Li S, Hsu MC, Strober B. Long-term safety experience of ustekinumab in
patients with moderate-to-severe psoriasis (Part I of II): results from analyses
of general safety parameters from pooled Phase 2 and 3 clinical trials. J Am Acad
Dermatol. 2012 May;66(5):731-41. doi: 10.1016/j.jaad.2011.06.011. Epub 2011 Sep
17. PubMed PMID: 21930328.

579: Yang XY, Chen QF, Li YP, Wu SM. Mycobacterium vaccae as adjuvant therapy to
anti-tuberculosis chemotherapy in never-treated tuberculosis patients: a
meta-analysis. PLoS One. 2011;6(9):e23826. doi: 10.1371/journal.pone.0023826.
Epub 2011 Sep 6. PubMed PMID: 21909406; PubMed Central PMCID: PMC3167806.

580: Chen YC, Chin CH, Liu SF, Wu CC, Tsen CC, Wang YH, Chao TY, Lie CH, Chen CJ,
Wang CC, Lin MC. Prognostic values of serum IP-10 and IL-17 in patients with
pulmonary tuberculosis. Dis Markers. 2011;31(2):101-10. doi:
10.3233/DMA-2011-0808. PubMed PMID: 21897004; PubMed Central PMCID: PMC3826581.

581: SchÃ¶n T, Idh J, Westman A, Elias D, Abate E, Diro E, Moges F, Kassu A, Ayele
B, Forslund T, Getachew A, Britton S, Stendahl O, Sundqvist T. Effects of a food
supplement rich in arginine in patients with smear positive pulmonary
tuberculosis--a randomised trial. Tuberculosis (Edinb). 2011 Sep;91(5):370-7.
doi: 10.1016/j.tube.2011.06.002. Epub 2011 Aug 2. PubMed PMID: 21813328.

582: Liesker JJ, Bathoorn E, Postma DS, Vonk JM, Timens W, Kerstjens HA. Sputum
inflammation predicts exacerbations after cessation of inhaled corticosteroids in
COPD. Respir Med. 2011 Dec;105(12):1853-60. doi: 10.1016/j.rmed.2011.07.002. Epub
2011 Jul 29. PubMed PMID: 21802933.

583: Gler MT, Macalintal LE, Raymond L, Guilatco R, Quelapio MI, Tupasi TE.

Multidrug-resistant tuberculosis among previously treated patients in the
Philippines. Int J Tuberc Lung Dis. 2011 May;15(5):652-6. doi:
10.5588/ijtld.10.0400. PubMed PMID: 21756517.

584: Ozsahin SL, Arslan S, Epozturk K, Remziye E, Dogan OT. Chest X-ray and
bacteriology in the initial phase of treatment of 800 male patients with
pulmonary tuberculosis. J Bras Pneumol. 2011 May-Jun;37(3):294-301. English,
Portuguese. PubMed PMID: 21755183.

585: Koh WJ, Choi GE, Lee SH, Park YK, Lee NY, Shin SJ. First case of
Segniliparus rotundus pneumonia in a patient with bronchiectasis. J Clin
Microbiol. 2011 Sep;49(9):3403-5. doi: 10.1128/JCM.01023-11. Epub 2011 Jul 13.
PubMed PMID: 21752969; PubMed Central PMCID: PMC3165615.

586: Baker MA, Harries AD, Jeon CY, Hart JE, Kapur A, LÃ¶nnroth K, Ottmani SE,
Goonesekera SD, Murray MB. The impact of diabetes on tuberculosis treatment
outcomes: a systematic review. BMC Med. 2011 Jul 1;9:81. doi:
10.1186/1741-7015-9-81. PubMed PMID: 21722362; PubMed Central PMCID:
PMC3155828.

587: Gao XF, Yang ZW, Li J. Adjunctive therapy with interferon-gamma for the
treatment of pulmonary tuberculosis: a systematic review. Int J Infect Dis. 2011
Sep;15(9):e594-600. doi: 10.1016/j.ijid.2011.05.002. Epub 2011 Jun 28. Review.
PubMed PMID: 21715206.

588: Yehia MM, Abdulla ZA. Isolation of Histoplasma capsulatum and Blastomyces
dermatitidis from Iraqi Patients with Lower Respiratory Tract Infections. J IMA.
2011 Jul;43(2):68-73. doi: 10.5915/43-2-5929. PubMed PMID: 23610487; PubMed
Central PMCID: PMC3516054.

589: Chigutsa E, Visser ME, Swart EC, Denti P, Pushpakom S, Egan D, Holford NH,
Smith PJ, Maartens G, Owen A, McIlleron H. The SLCO1B1 rs4149032 polymorphism is

highly prevalent in South Africans and is associated with reduced rifampin
concentrations: dosing implications. Antimicrob Agents Chemother. 2011
Sep;55(9):4122-7. doi: 10.1128/AAC.01833-10. Epub 2011 Jun 27. PubMed PMID:
21709081; PubMed Central PMCID: PMC3165308.

590: Bai L, Hong Z, Gong C, Yan D, Liang Z. Surgical treatment efficacy in 172
cases of tuberculosis-destroyed lungs. Eur J Cardiothorac Surg. 2012
Feb;41(2):335-40. doi: 10.1016/j.ejcts.2011.05.028. Epub 2011 Dec 12. PubMed
PMID: 21684172.

591: Schoeman JF, Janse van Rensburg A, Laubscher JA, Springer P. The role of
aspirin in childhood tuberculous meningitis. J Child Neurol. 2011
Aug;26(8):956-62. doi: 10.1177/0883073811398132. Epub 2011 May 31. PubMed PMID:
21628697.

592: Joseph P, Desai VB, Mohan NS, Fredrick JS, Ramachandran R, Raman B, Wares F,
Ramachandran R, Thomas A. Outcome of standardized treatment for patients with
MDR-TB from Tamil Nadu, India. Indian J Med Res. 2011 May;133:529-34. PubMed
PMID: 21623039; PubMed Central PMCID: PMC3121285.

593: Sha W, Liang L, Jiang RH, Fang Y, Yin HY, Xiao HP. [The effect of the
regimen containing cefoxitin on highly drug-resistant rapidly growing
nontuberculous mycobacterial pulmonary disease]. Zhonghua Jie He He Hu Xi Za Zhi.
2011 Apr;34(4):265-8. Chinese. PubMed PMID: 21609609.

594: Phua CK, Chee CB, Chua AP, Gan SH, Ahmed AD, Wang YT. Managing a case of
extensively drug-resistant (XDR) pulmonary tuberculosis in Singapore. Ann Acad
Med Singapore. 2011 Mar;40(3):132-5. PubMed PMID: 21603731.

595: TÃ¶rÃ¶k ME, Yen NT, Chau TT, Mai NT, Phu NH, Mai PP, Dung NT, Chau NV, Bang
ND, Tien NA, Minh NH, Hien NQ, Thai PV, Dong DT, Anh DT, Thoa NT, Hai NN, Lan NN,
Lan NT, Quy HT, Dung NH, Hien TT, Chinh NT, Simmons CP, de Jong M, Wolbers M,

Farrar JJ. Timing of initiation of antiretroviral therapy in human immunodeficiency virus (HIV)--associated tuberculous meningitis. Clin Infect Dis.
2011 Jun;52(11):1374-83. doi: 10.1093/cid/cir230. PubMed PMID: 21596680; PubMed
Central PMCID: PMC4340579.

596: Pratt RH, Winston CA, Kammerer JS, Armstrong LR. Tuberculosis in older
adults in the United States, 1993-2008. J Am Geriatr Soc. 2011 May;59(5):851-7.
doi: 10.1111/j.1532-5415.2011.03369.x. Epub 2011 Apr 21. PubMed PMID: 21517786.

597: Mac Kenzie WR, Heilig CM, Bozeman L, Johnson JL, Muzanye G, Dunbar D, Jost
KC Jr, Diem L, Metchock B, Eisenach K, Dorman S, Goldberg S. Geographic
differences in time to culture conversion in liquid media: Tuberculosis Trials
Consortium study 28. Culture conversion is delayed in Africa. PLoS One. 2011 Apr
11;6(4):e18358. doi: 10.1371/journal.pone.0018358. PubMed PMID: 21494548; PubMed
Central PMCID: PMC3073969.

598: Kota SK, Jammula S, Kota SK, Tripathy PR, Panda S, Modi KD. Effect of
vitamin D supplementation in type 2 diabetes patients with pulmonary
tuberculosis. Diabetes Metab Syndr. 2011 Apr-Jun;5(2):85-9. doi:
10.1016/j.dsx.2012.02.021. Epub 2012 Mar 15. PubMed PMID: 22813409.

599: RadoviÄ‡ M, RistiÄ‡ L, StankoviÄ‡ I, PetroviÄ‡ D. [Rare congenital heart disease
as a cause of tuberculosis activation]. Med Pregl. 2010 Jul-Aug;63(7-8):565-9.
Serbian. PubMed PMID: 21446150.

600: Selvaraj P. Vitamin D, vitamin D receptor, and cathelicidin in the treatment
of tuberculosis. Vitam Horm. 2011;86:307-25. doi:
10.1016/B978-0-12-386960-9.00013-7. Review. PubMed PMID: 21419277.

601: Klareskog L, Gaubitz M, RodrÃ-guez-Valverde V, Malaise M, Dougados M, Wajdula
J; Etanercept Study 301 Investigators. Assessment of long-term safety and
efficacy of etanercept in a 5-year extension study in patients with rheumatoid

arthritis. Clin Exp Rheumatol. 2011 Mar-Apr;29(2):238-47. Epub 2011 Apr 19.
PubMed PMID: 21418785.

602: Monedero I, Caminero JA. Evidence for promoting fixed-dose combination drugs
in tuberculosis treatment and control: a review. Int J Tuberc Lung Dis. 2011
Apr;15(4):433-9. doi: 10.5588/ijtld.09.0439. Review. PubMed PMID: 21396199.

603: Greenwald MW, Shergy WJ, Kaine JL, Sweetser MT, Gilder K, Linnik MD.
Evaluation of the safety of rituximab in combination with a tumor necrosis factor
inhibitor and methotrexate in patients with active rheumatoid arthritis: results
from a randomized controlled trial. Arthritis Rheum. 2011 Mar;63(3):622-32. doi:
10.1002/art.30194. PubMed PMID: 21360491.

604: Steingart KR, Jotblad S, Robsky K, Deck D, Hopewell PC, Huang D, Nahid P.
Higher-dose rifampin for the treatment of pulmonary tuberculosis: a systematic
review. Int J Tuberc Lung Dis. 2011 Mar;15(3):305-16. Review. PubMed PMID:
21333096.

605: Wateba MI, Diop SA, Salou M, Womitso K, Nichols S, Tidjani O. [Sputum smear
conversion during intensive TB treatment phase according to HIV status, in
hospitalised patients in Togo]. Med Mal Infect. 2011 Mar;41(3):140-4. doi:
10.1016/j.medmal.2010.12.011. Epub 2011 Feb 1. French. PubMed PMID: 21282024.

606: Atkins S, Lewin S, Jordaan E, Thorson A. Lay health worker-supported
tuberculosis treatment adherence in South Africa: an interrupted time-series
study. Int J Tuberc Lung Dis. 2011 Jan;15(1):84-9, i. PubMed PMID: 21276302.

607: Dharmadhikari AS, Nardell E. Serial acid fast bacilli smear and culture
conversion rates over 26 weeks in a cohort of 93 sputum cultureâ€"positive
tuberculosis (TB). Clin Infect Dis. 2011 Feb 15;52(4):554-6. doi:

10.1093/cid/ciq182. Erratum in: Clin Infect Dis. 2012 Nov 15;55(10):1439. PubMed
PMID: 21258108; PubMed Central PMCID: PMC3060903.

608: Brust JC, Lygizos M, Chaiyachati K, Scott M, van der Merwe TL, Moll AP, Li
X, Loveday M, Bamber SA, Lalloo UG, Friedland GH, Shah NS, Gandhi NR. Culture
conversion among HIV co-infected multidrug-resistant tuberculosis patients in
Tugela Ferry, South Africa. PLoS One. 2011 Jan 6;6(1):e15841. doi:
10.1371/journal.pone.0015841. PubMed PMID: 21253585; PubMed Central PMCID:
PMC3017058.

609: Butov DA, Pashkov YN, Stepanenko AL, Choporova AI, Butova TS, Batdelger D,
Jirathitikal V, Bourinbaiar AS, Zaitzeva SI. Phase IIb randomized trial of
adjunct immunotherapy in patients with first-diagnosed tuberculosis, relapsed and
multi-drug-resistant (MDR) TB. J Immune Based Ther Vaccines. 2011 Jan 18;9:3.
doi: 10.1186/1476-8518-9-3. PubMed PMID: 21244690; PubMed Central PMCID:
PMC3031205.

610: Chappell GP, Xiao X, Pica-Mendez A, Varnell T, Green S, Tanaka WK, Laterza
O. Quantitative measurement of cysteinyl leukotrienes and leukotriene Bâ,„ in human
sputum using ultra high pressure liquid chromatography-tandem mass spectrometry.
J Chromatogr B Analyt Technol Biomed Life Sci. 2011 Feb 1;879(3-4):277-84. doi:
10.1016/j.jchromb.2010.12.014. Epub 2010 Dec 23. PubMed PMID: 21227760.

611: Biere SS, Maas KW, Bonavina L, Garcia JR, van Berge Henegouwen MI, Rosman C,
Sosef MN, de Lange ES, Bonjer HJ, Cuesta MA, van der Peet DL. Traditional
invasive vs. minimally invasive esophagectomy: a multi-center, randomized trial
(TIME-trial). BMC Surg. 2011 Jan 12;11:2. doi: 10.1186/1471-2482-11-2. PubMed
PMID: 21226918; PubMed Central PMCID: PMC3031195.

612: Jahnavi G, Sudha CH. Randomised controlled trial of food supplements in

patients with newly diagnosed tuberculosis and wasting. Singapore Med J. 2010
Dec;51(12):957-62. PubMed PMID: 21221502.

613: Rasmussen TA, SÃ¸gaard OS, Camara C, Andersen PL, Wejse C. Serum
procalcitonin in pulmonary tuberculosis. Int J Tuberc Lung Dis. 2011
Feb;15(2):251-6, i. PubMed PMID: 21219690.

614: Martineau AR, Timms PM, Bothamley GH, Hanifa Y, Islam K, Claxton AP, Packe
GE, Moore-Gillon JC, Darmalingam M, Davidson RN, Milburn HJ, Baker LV, Barker RD,
Woodward NJ, Venton TR, Barnes KE, Mullett CJ, Coussens AK, Rutterford CM, Mein
CA, Davies GR, Wilkinson RJ, Nikolayevskyy V, Drobniewski FA, Eldridge SM,
Griffiths CJ. High-dose vitamin D(3) during intensive-phase antimicrobial
treatment of pulmonary tuberculosis: a double-blind randomised controlled trial.
Lancet. 2011 Jan 15;377(9761):242-50. doi: 10.1016/S0140-6736(10)61889-2. Epub
2011 Jan 5. PubMed PMID: 21215445; PubMed Central PMCID: PMC4176755.

615: Reinisch W, Sandborn WJ, Hommes DW, D'Haens G, Hanauer S, Schreiber S,
Panaccione R, Fedorak RN, Tighe MB, Huang B, Kampman W, Lazar A, Thakkar R.
Adalimumab for induction of clinical remission in moderately to severely active
ulcerative colitis: results of a randomised controlled trial. Gut. 2011
Jun;60(6):780-7. doi: 10.1136/gut.2010.221127. Epub 2011 Jan 5. PubMed PMID:
21209123.

616: Joseph N, Nagaraj K, Bhat J, Babu R, Kotian S, Ranganatha Y, Hocksan A,
Shetty V, Zaki N, Swasthik K, Hamzah N. Treatment outcomes among new smear
positive and retreatment cases of tuberculosis in Mangalore, South India - a
descriptive study. Australas Med J. 2011;4(4):162-7. doi: 10.4066/AMJ.2011.585.
Epub 2011 Apr 30. PubMed PMID: 23393507; PubMed Central PMCID: PMC3562894.

617: Siddiqui UA, O'Toole M, Kabir Z, Qureshi S, Gibbons N, Kane M, Keane J.

Smoking prolongs the infectivity of patients with tuberculosis. Ir Med J. 2010
Oct;103(9):278-80. PubMed PMID: 21186753.

618: Feurle GE, Moos V, Schinnerling K, Geelhaar A, Allers K, Biagi F, BlÃ¤ker H,
Moter A, Loddenkemper C, Jansen A, Schneider T. The immune reconstitution
inflammatory syndrome in whipple disease: a cohort study. Ann Intern Med. 2010
Dec 7;153(11):710-7. doi: 10.7326/0003-4819-153-11-201012070-00004. PubMed PMID:
21135294.

619: Kobashi Y, Abe M, Mouri K, Obase Y, Miyashita N, Oka M. Clinical usefulness
of combination chemotherapy for pulmonary Mycobacterium avium complex disease. J
Infect. 2010 Nov 19. [Epub ahead of print] PubMed PMID: 21094662.

620: Visser ME, Grewal HM, Swart EC, Dhansay MA, Walzl G, Swanevelder S, Lombard
C, Maartens G. The effect of vitamin A and zinc supplementation on treatment
outcomes in pulmonary tuberculosis: a randomized controlled trial. Am J Clin
Nutr. 2011 Jan;93(1):93-100. doi: 10.3945/ajcn.110.001784. Epub 2010 Nov 10.
PubMed PMID: 21068353.

621: Okumura M, Yoshiyama T, Maeda S, Kazumi Y, Azuma Y, Ueyama M, Morimoto K,
Kurashima A, Ogata H, Kudoh S. [Clinical analysis of extensively-drug resistant
tuberculosis (XDR-TB) in our hospital]. Kekkaku. 2010 Oct;85(10):737-42.
Japanese. PubMed PMID: 21061563.

622: Lawson L, Thacher TD, Yassin MA, Onuoha NA, Usman A, Emenyonu NE, Shenkin A,
Davies PD, Cuevas LE. Randomized controlled trial of zinc and vitamin A as
co-adjuvants for the treatment of pulmonary tuberculosis. Trop Med Int Health.
2010 Dec;15(12):1481-90. doi: 10.1111/j.1365-3156.2010.02638.x. Epub 2010 Oct 19.
PubMed PMID: 20958890.

623: Pardeshi GS. Time of default in tuberculosis patients on directly observed

treatment. J Glob Infect Dis. 2010 Sep;2(3):226-30. doi:
10.4103/0974-777X.68533.
PubMed PMID: 20927282; PubMed Central PMCID: PMC2946677.

624: Maneechotesuwan K, Ekjiratrakul W, Kasetsinsombat K,
Wongkajornsilp A,
Barnes PJ. Statins enhance the anti-inflammatory effects of inhaled
corticosteroids in asthmatic patients through increased induction of
indoleamine
2, 3-dioxygenase. J Allergy Clin Immunol. 2010 Oct;126(4):754-
762.e1. doi:
10.1016/j.jaci.2010.08.005. PubMed PMID: 20920765.

625: Pakasi TA, Karyadi E, Suratih NM, Salean M, Darmawidjaja N, Bor
H, van der
Velden K, Dolmans WM, van der Meer JW. Zinc and vitamin A
supplementation fails
to reduce sputum conversion time in severely malnourished pulmonary
tuberculosis
patients in Indonesia. Nutr J. 2010 Sep 28;9:41. doi: 10.1186/1475-
2891-9-41.
PubMed PMID: 20920186; PubMed Central PMCID: PMC2957385.

626: Jeremiah K, Praygod G, Faurholt-Jepsen D, Range N, Andersen AB,
Grewal HM,
Friis H. BCG vaccination status may predict sputum conversion in
patients with
pulmonary tuberculosis: a new consideration for an old vaccine?
Thorax. 2010
Dec;65(12):1072-6. doi: 10.1136/thx.2010.134767. Epub 2010 Oct 1.
PubMed PMID:
20889521.

627: Sim YS, Park HY, Jeon K, Suh GY, Kwon OJ, Koh WJ. Standardized
combination
antibiotic treatment of Mycobacterium avium complex lung disease.
Yonsei Med J.
2010 Nov;51(6):888-94. doi: 10.3349/ymj.2010.51.6.888. PubMed PMID:
20879056;
PubMed Central PMCID: PMC2995975.

628: Papp KA, Poulin Y, Bissonnette R, Bourcier M, Toth D, Rosoph L,
Poulin-Costello M, Setterfield M, Syrotuik J. Assessment of the
long-term safety
and effectiveness of etanercept for the treatment of psoriasis in an
adult
population. J Am Acad Dermatol. 2012 Feb;66(2):e33-45. doi:
10.1016/j.jaad.2010.07.026. Epub 2010 Sep 17. PubMed PMID: 20850895.

629: Thomas TA, Shenoi SV, Heysell SK, Eksteen FJ, Sunkari VB, Gandhi NR,
Friedland G, Shah NS. Extensively drug-resistant tuberculosis in children with
human immunodeficiency virus in rural South Africa. Int J Tuberc Lung Dis. 2010
Oct;14(10):1244-51. PubMed PMID: 20843414; PubMed Central PMCID: PMC3030274.

630: Koh WJ, Jeon K, Lee NY, Kim BJ, Kook YH, Lee SH, Park YK, Kim CK, Shin SJ,
Huitt GA, Daley CL, Kwon OJ. Clinical significance of differentiation of
Mycobacterium massiliense from Mycobacterium abscessus. Am J Respir Crit Care
Med. 2011 Feb 1;183(3):405-10. doi: 10.1164/rccm.201003-0395OC. Epub 2010 Sep 10.
PubMed PMID: 20833823.

631: Meintjes G, Wilkinson RJ, Morroni C, Pepper DJ, Rebe K, Rangaka MX, Oni T,
Maartens G. Randomized placebo-controlled trial of prednisone for paradoxical
tuberculosis-associated immune reconstitution inflammatory syndrome. AIDS. 2010
Sep 24;24(15):2381-90. doi: 10.1097/QAD.0b013e32833dfc68. PubMed PMID: 20808204;
PubMed Central PMCID: PMC2940061.

632: Chen YC, Chang HC, Chen CJ, Liu SF, Chin CH, Wu CC, Chao TY, Lie CH, Wang
CC, Lin MC. Blood absolute T cell counts may predict 2-month treatment response
in patients with pulmonary tuberculosis. Dis Markers. 2010;28(6):343-52. doi:
10.3233/DMA-2010-0714. PubMed PMID: 20683148; PubMed Central PMCID: PMC3833334.

633: Senkoro M, Mfinanga SG, MÃ¸rkve O. Smear microscopy and culture conversion
rates among smear positive pulmonary tuberculosis patients by HIV status in Dar
es Salaam, Tanzania. BMC Infect Dis. 2010 Jul 16;10:210. doi:
10.1186/1471-2334-10-210. PubMed PMID: 20637077; PubMed Central PMCID:
PMC2918607.

634: Fitzwater SP, Caviedes L, Gilman RH, Coronel J, LaChira D, Salazar C,
Saravia JC, Reddy K, Friedland JS, Moore DA. Prolonged infectiousness of

tuberculosis patients in a directly observed therapy short-course program with
standardized therapy. Clin Infect Dis. 2010 Aug 15;51(4):371-8. doi:
10.1086/655127. PubMed PMID: 20624064; PubMed Central PMCID:
PMC4465448.

635: Grant PM, Komarow L, Andersen J, Sereti I, Pahwa S, Lederman MM, Eron J,
Sanne I, Powderly W, Hogg E, Suckow C, Zolopa A. Risk factor analyses for immune
reconstitution inflammatory syndrome in a randomized study of early vs. deferred
ART during an opportunistic infection. PLoS One. 2010 Jul 1;5(7):e11416. doi:
10.1371/journal.pone.0011416. PubMed PMID: 20617176; PubMed Central PMCID:
PMC2895658.

636: Yamshchikov AV, Kurbatova EV, Kumari M, Blumberg HM, Ziegler TR, Ray SM,
Tangpricha V. Vitamin D status and antimicrobial peptide cathelicidin (LL-37)
concentrations in patients with active pulmonary tuberculosis. Am J Clin Nutr.
2010 Sep;92(3):603-11. doi: 10.3945/ajcn.2010.29411. Epub 2010 Jul 7. PubMed
PMID: 20610636; PubMed Central PMCID: PMC2921537.

637: Matteelli A, Carvalho AC, Dooley KE, Kritski A. TMC207: the first compound
of a new class of potent anti-tuberculosis drugs. Future Microbiol. 2010
Jun;5(6):849-58. doi: 10.2217/fmb.10.50. Review. PubMed PMID: 20521931; PubMed
Central PMCID: PMC2921705.

638: UzundaÄŸ IÅŸeri A, Dulkar G, SelÃ§uk SÃ¶nmez O, Yilmaz Aydin L, Yilmaz B.
Factors that effect sputum culture conversion rate in hospitalized patients with
pulmonary tuberculosis who were applied directly observation therapy and
non-directly observation therapy. Tuberk Toraks. 2010;58(1):44-52. PubMed PMID:
20517728.

639: Su WJ, Feng JY, Chiu YC, Huang SF, Lee YC. Role of 2-month sputum smears in
predicting culture conversion in pulmonary tuberculosis. Eur Respir J. 2011

Feb;37(2):376-83. doi: 10.1183/09031936.00007410. Epub 2010 Jun 1. PubMed PMID:
20516049.

640: Don BR, Kim K, Li J, Dwyer T, Alexander F, Kaysen GA. The effect of
etanercept on suppression of the systemic inflammatory response in chronic
hemodialysis patients. Clin Nephrol. 2010 Jun;73(6):431-8. PubMed PMID: 20497755.

641: Chiang CY, Schaaf HS. Management of drug-resistant tuberculosis. Int J
Tuberc Lung Dis. 2010 Jun;14(6):672-82. Review. PubMed PMID: 20487603.

642: Armijos RX, Weigel MM, Chacon R, Flores L, Campos A. Adjunctive
micronutrient supplementation for pulmonary tuberculosis. Salud Publica Mex. 2010
May-Jun;52(3):185-9. PubMed PMID: 20485880.

643: MartÃ-n-Mola E, Sieper J, Leirisalo-Repo M, Dijkmans BA, Vlahos B, Pedersen
R, Koenig AS, Freundlich B. Sustained efficacy and safety, including
patient-reported outcomes, with etanercept treatment over 5 years in patients
with ankylosing spondylitis. Clin Exp Rheumatol. 2010 Mar-Apr;28(2):238-45. Epub
2010 May 13. PubMed PMID: 20483046.

644: Misra UK, Kalita J, Nair PP. Role of aspirin in tuberculous meningitis: a
randomized open label placebo controlled trial. J Neurol Sci. 2010 Jun
15;293(1-2):12-7. doi: 10.1016/j.jns.2010.03.025. Epub 2010 Apr 24. PubMed PMID:
20421121.

645: Hesseling AC, Walzl G, Enarson DA, Carroll NM, Duncan K, Lukey PT, Lombard
C, Donald PR, Lawrence KA, Gie RP, van Helden PD, Beyers N. Baseline sputum time
to detection predicts month two culture conversion and relapse in
non-HIV-infected patients. Int J Tuberc Lung Dis. 2010 May;14(5):560-70. PubMed
PMID: 20392348.

646: Centers for Disease Control and Prevention (CDC). Monitoring tuberculosis

programs - National Tuberculosis Indicator Project, United States, 2002-2008.
MMWR Morb Mortal Wkly Rep. 2010 Mar 19;59(10):295-8. PubMed PMID: 20300056.

647: Shu CC, Wang JT, Lee CH, Wang JY, Lee LN, Yu CJ. Predicting results of
mycobacterial culture on sputum smear reversion after anti-tuberculous treatment:
a case control study. BMC Infect Dis. 2010 Mar 6;10:48. doi: 10.1186/1471-2334-10-48. PubMed PMID: 20205743; PubMed Central PMCID: PMC2845134.

648: Heller T, Lessells RJ, Wallrauch CG, BÃ¤rnighausen T, Cooke GS, Mhlongo L,
Master I, Newell ML. Community-based treatment for multidrug-resistant
tuberculosis in rural KwaZulu-Natal, South Africa. Int J Tuberc Lung Dis. 2010
Apr;14(4):420-6. PubMed PMID: 20202299.

649: Varkey P, Harris S, Edmonson L, McCoy K, Aksamit T, Brennan MD. An
innovative model for tuberculosis control: an academic medical center-public
health department partnership. Minn Med. 2010 Jan;93(1):39-41. PubMed PMID:
20191731.

650: Eum SY, Lee YJ, Min JH, Kwak HK, Hong MS, Kong JH, Hwang SH, Park SK,
Leblanc JJ, Via LE, Barry CE 3rd, Cho SN. Association of antigen-stimulated
release of tumor necrosis factor-alpha in whole blood with response to
chemotherapy in patients with pulmonary multidrug-resistant tuberculosis.
Respiration. 2010;80(4):275-84. doi: 10.1159/000283687. Epub 2010 Feb 10. PubMed
PMID: 20145387; PubMed Central PMCID: PMC2955738.

651: Qureshi SA, Bashir MU, Yaqinuddin A. Utility of DNA methylation markers for
diagnosing cancer. Int J Surg. 2010;8(3):194-8. doi: 10.1016/j.ijsu.2010.02.001.
Epub 2010 Feb 6. Review. PubMed PMID: 20139036.

652: Kuaban C, Bame R, Mouangue L, Djella S, Yomgni C. Non conversion of sputum

smears in new smear positive pulmonary tuberculosis patients in
YaoundÃ©,
Cameroon. East Afr Med J. 2009 May;86(5):219-25. PubMed PMID:
20084990.

653: Huang F, Zhang FC, Bao CD, Tao Y, Gu JR, Xu JH, Zhu P, Xu HJ,
Zhang ZY, Zhao
DB, Wu DH. [Adalimumab plus methotrexate for the treatment of
rheumatoid
arthritis: a multi-center randomized, double-blind, placebo-
controlled clinical
study.]. Zhonghua Nei Ke Za Zhi. 2009 Nov;48(11):916-21. Chinese.
PubMed PMID:
20079321.

654: Fairall L, Bachmann MO, Zwarenstein M, Bateman ED, Niessen LW,
Lombard C,
Majara B, English R, Bheekie A, van Rensburg D, Mayers P, Peters A,
Chapman R.
Cost-effectiveness of educational outreach to primary care nurses to
increase
tuberculosis case detection and improve respiratory care: economic
evaluation
alongside a randomised trial. Trop Med Int Health. 2010
Mar;15(3):277-86. doi:
10.1111/j.1365-3156.2009.02455.x. Epub 2010 Jan 11. PubMed PMID:
20070633.

655: Kwange SO, Budambula NL. Effectiveness of anti-tuberculosis
treatment among
patients receiving highly active antiretroviral therapy at Vihiga
district
hospital in 2007. Indian J Med Microbiol. 2010 Jan-Mar;28(1):21-5.
doi:
10.4103/0255-0857.58723. PubMed PMID: 20061758.

656: Mardining Raras TY, Noor Chozin I. The Soluble Plasminogen
Activator
Receptor as a Biomarker on Monitoring the Therapy Progress of
Pulmonary TB-AFB(+)
Patients. Tuberc Res Treat. 2010;2010:406346. doi:
10.1155/2010/406346. Epub 2010
Oct 4. PubMed PMID: 22567258; PubMed Central PMCID: PMC3335589.

657: Via LE, Cho SN, Hwang S, Bang H, Park SK, Kang HS, Jeon D, Min
SY, Oh T, Kim
Y, Kim YM, Rajan V, Wong SY, Shamputa IC, Carroll M, Goldfeder L,
Lee SA, Holland
SM, Eum S, Lee H, Barry CE 3rd. Polymorphisms associated with
resistance and

cross-resistance to aminoglycosides and capreomycin in Mycobacterium tuberculosis
isolates from South Korean Patients with drug-resistant tuberculosis. J Clin
Microbiol. 2010 Feb;48(2):402-11. doi: 10.1128/JCM.01476-09. Epub 2009 Dec 23.
PubMed PMID: 20032248; PubMed Central PMCID: PMC2815586.

658: Moreno A, SÃ¡nchez F, Nelson J, MirÃ³ JM, CaylÃ JA; Grupo de Trabajo de la
UITB-TBTC (site 31). [On the way to shortening tuberculosis treatments: clinical
trials of the Unitat d' InvestagaciÃ³ en Tuberculosi de Barcelona supported by the
Centers for Disease Control and Prevention]. Gac Sanit. 2010
Mar-Apr;24(2):171.e1-6. doi: 10.1016/j.gaceta.2009.08.008. Epub 2009 Dec 10.
Spanish. PubMed PMID: 20005019.

659: Wang JY, Wang JT, Tsai TH, Hsu CL, Yu CJ, Hsueh PR, Lee LN, Yang PC. Adding
moxifloxacin is associated with a shorter time to culture conversion in pulmonary
tuberculosis. Int J Tuberc Lung Dis. 2010 Jan;14(1):65-71. PubMed PMID: 20003697.

660: Shao HJ, Crump JA, Ramadhani HO, Uiso LO, Ole-Nguyaine S, Moon AM, Kiwera
RA, Woods CW, Shao JF, Bartlett JA, Thielman NM. Early versus delayed fixed dose
combination abacavir/lamivudine/zidovudine in patients with HIV and tuberculosis
in Tanzania. AIDS Res Hum Retroviruses. 2009 Dec;25(12):1277-85. doi:
10.1089/aid.2009.0100. PubMed PMID: 20001518; PubMed Central PMCID: PMC2858925.

661: GullÃ³n JA, SuÃ¡rez I, Lecuona M, FernÃ¡ndez R, Rubinos G, Medina A, Cabrera C,
GonzÃ¡lez I. Time to culture conversion in smokers with pulmonary tuberculosis.
Monaldi Arch Chest Dis. 2009 Sep;71(3):127-31. PubMed PMID: 19999959.

662: Horne DJ, Johnson CO, Oren E, Spitters C, Narita M. How soon should patients
with smear-positive tuberculosis be released from inpatient isolation? Infect
Control Hosp Epidemiol. 2010 Jan;31(1):78-84. doi: 10.1086/649022. PubMed PMID:
19968490; PubMed Central PMCID: PMC3046814.

663: Yu DP, Fu Y. [Surgical treatment of 133 cases of multi-drug-resistant
pulmonary tuberculosis]. Zhonghua Jie He He Hu Xi Za Zhi. 2009 Jun;32(6):450-3.
Chinese. PubMed PMID: 19957782.

664: Lounis N, Guillemont J, Veziris N, Koul A, Jarlier V, Andries K. [R207910
(TMC207): a new antibiotic for the treatment of tuberculosis]. Med Mal Infect.
2010 Jul;40(7):383-90. doi: 10.1016/j.medmal.2009.09.007. Epub 2009 Dec 1.
Review. French. PubMed PMID: 19954909.

665: Malhotra HS, Garg RK, Singh MK, Agarwal A, Verma R. Corticosteroids
(dexamethasone versus intravenous methylprednisolone) in patients with
tuberculous meningitis. Ann Trop Med Parasitol. 2009 Oct;103(7):625-34. doi:
10.1179/000349809X12502035776315. PubMed PMID: 19825284.

666: Green JA, Tran CT, Farrar JJ, Nguyen MT, Nguyen PH, Dinh SX, Ho ND, Ly CV,
Tran HT, Friedland JS, Thwaites GE. Dexamethasone, cerebrospinal fluid matrix
metalloproteinase concentrations and clinical outcomes in tuberculous meningitis.
PLoS One. 2009 Sep 30;4(9):e7277. doi: 10.1371/journal.pone.0007277. PubMed PMID:
19789647; PubMed Central PMCID: PMC2748711.

667: Huang HC, Lee LA, Fang TJ, Li HY, Lo CC, Wu JH. Transnasal butorphanol for
pain relief after uvulopalatopharyngoplasty - a hospital-based,randomized study.
Chang Gung Med J. 2009 Jul-Aug;32(4):390-9. PubMed PMID: 19664345.

668: Jeon K, Kwon OJ, Lee NY, Kim BJ, Kook YH, Lee SH, Park YK, Kim CK, Koh WJ.
Antibiotic treatment of Mycobacterium abscessus lung disease: a retrospective
analysis of 65 patients. Am J Respir Crit Care Med. 2009 Nov 1;180(9):896-902.
doi: 10.1164/rccm.200905-0704OC. Epub 2009 Aug 6. PubMed PMID: 19661243.

669: Wang JY, Lee LN, Yu CJ, Chien YJ, Yang PC; Tami Group. Factors influencing
time to smear conversion in patients with smear-positive pulmonary tuberculosis.
Respirology. 2009 Sep;14(7):1012-9. doi: 10.1111/j.1440-1843.2009.01598.x. Epub
2009 Jul 30. PubMed PMID: 19659516.

670: Sandborn WJ, Rutgeerts P, Feagan BG, Reinisch W, Olson A, Johanns J, Lu J,
Horgan K, Rachmilewitz D, Hanauer SB, Lichtenstein GR, de Villiers WJ, Present D,
Sands BE, Colombel JF. Colectomy rate comparison after treatment of ulcerative
colitis with placebo or infliximab. Gastroenterology. 2009 Oct;137(4):1250-60;
quiz 1520. doi: 10.1053/j.gastro.2009.06.061. Epub 2009 Jul 28. PubMed PMID:
19596014.

671: Kang WL, Xie YG, Tan WG, Chu NH, Li L, You YH, Yang YZ, Wang XM, Yan XL,
Miao ZP, Duanmu HJ. [Study on the efficacy and safety of short-term treatment
including fluoroquinolones anti-tuberculosis drugs for rifampicin resistant
pulmonary tuberculosis]. Zhonghua Liu Xing Bing Xue Za Zhi. 2009
Feb;30(2):179-83. Chinese. PubMed PMID: 19565883.

672: Cimen NO, Arslan Z, Saygi A, Ocak K, Babacan F, Kurutepe M. Radiometric vs.
agar proportion methods for assessing ethionamide resistance and its clinical
effects in multidrug-resistant tuberculosis. Int J Tuberc Lung Dis. 2009
Jul;13(7):907-13. PubMed PMID: 19555543.

673: O'Donnell MR, Padayatchi N, Master I, Osburn G, Horsburgh CR. Improved early
results for patients with extensively drug-resistant tuberculosis and HIV in
South Africa. Int J Tuberc Lung Dis. 2009 Jul;13(7):855-61. PubMed PMID:
19555535; PubMed Central PMCID: PMC2855970.

674: Gao XF, Li J, Yang ZW, Li YP. Rifapentine vs. rifampicin for the treatment
of pulmonary tuberculosis: a systematic review. Int J Tuberc Lung Dis. 2009
Jul;13(7):810-9. Review. PubMed PMID: 19555529.

675: Johnson JL, Hadad DJ, Dietze R, Maciel EL, Sewali B, Gitta P, Okwera A,
Mugerwa RD, Alcaneses MR, Quelapio MI, Tupasi TE, Horter L, Debanne SM, Eisenach
KD, Boom WH. Shortening treatment in adults with noncavitary tuberculosis and
2-month culture conversion. Am J Respir Crit Care Med. 2009 Sep 15;180(6):558-63.
doi: 10.1164/rccm.200904-0536OC. Epub 2009 Jun 19. PubMed PMID: 19542476; PubMed
Central PMCID: PMC2742745.

676: Hasegawa N, Nishimura T, Ohtani S, Takeshita K, Fukunaga K, Tasaka S, Urano
T, Ishii K, Miyairi M, Ishizaka A. Therapeutic effects of various initial
combinations of chemotherapy including clarithromycin against Mycobacterium avium
complex pulmonary disease. Chest. 2009 Dec;136(6):1569-1575. doi:
10.1378/chest.08-2567. Epub 2009 Jun 19. PubMed PMID: 19542259.

677: Turusov AA, Valiev RSh, Chesnokova RV. [Comparative study of microscopy by
the Ziehl Neelsen method, routine fluorescence microscopy, and fluorescence
microscopy using a lumin attachment in the diagnosis of acid-resistant
mycobacteria]. Probl Tuberk Bolezn Legk. 2009;(4):41-5. Russian. PubMed PMID:
19514454.

678: Diacon AH, Pym A, Grobusch M, Patientia R, Rustomjee R, Page-Shipp L,
Pistorius C, Krause R, Bogoshi M, Churchyard G, Venter A, Allen J, Palomino JC,
De Marez T, van Heeswijk RP, Lounis N, Meyvisch P, Verbeeck J, Parys W, de Beule
K, Andries K, Mc Neeley DF. The diarylquinoline TMC207 for multidrug-resistant
tuberculosis. N Engl J Med. 2009 Jun 4;360(23):2397-405. doi:
10.1056/NEJMoa0808427. PubMed PMID: 19494215.

679: Koh WJ, Kwon OJ, Gwak H, Chung JW, Cho SN, Kim WS, Shim TS. Daily 300 mg
dose of linezolid for the treatment of intractable multidrug-resistant and
extensively drug-resistant tuberculosis. J Antimicrob Chemother. 2009
Aug;64(2):388-91. doi: 10.1093/jac/dkp171. Epub 2009 May 25. PubMed PMID:
19468028.

680: Bartacek A, SchÃ¼tt D, Panosch B, Borek M; Rimstar 4-FDC Study Group.
Comparison of a four-drug fixed-dose combination regimen with a single tablet
regimen in smear-positive pulmonary tuberculosis. Int J Tuberc Lung Dis. 2009
Jun;13(6):760-6. PubMed PMID: 19460254.

681: Zaitzeva SI, Matveeva SL, Gerasimova TG, Pashkov YN, Butov DA, Pylypchuk VS,
Frolov VM, Kutsyna GA. Treatment of cavitary and infiltrating pulmonary
tuberculosis with and without the immunomodulator Dzherelo. Clin Microbiol
Infect. 2009 Dec;15(12):1154-62. doi: 10.1111/j.1469-0691.2009.02760.x. Epub 2009
May 16. PubMed PMID: 19456829.

682: Jain NK, Agnihotri SP, Sharma KK, Gupta S, Gupta P. Extensively drug
resistance (XDR) tb is not always fatal. Indian J Tuberc. 2009 Jan;56(1):48-50.
PubMed PMID: 19402272.

683: Dooley KE, Tang T, Golub JE, Dorman SE, Cronin W. Impact of diabetes
mellitus on treatment outcomes of patients with active tuberculosis. Am J Trop
Med Hyg. 2009 Apr;80(4):634-9. PubMed PMID: 19346391; PubMed Central PMCID:
PMC2750857.

684: Conde MB, Efron A, Loredo C, De Souza GR, GraÃ§a NP, Cezar MC, Ram M,
Chaudhary MA, Bishai WR, Kritski AL, Chaisson RE. Moxifloxacin versus ethambutol
in the initial treatment of tuberculosis: a double-blind, randomised, controlled
phase II trial. Lancet. 2009 Apr 4;373(9670):1183-9. doi:
10.1016/S0140-6736(09)60333-0. PubMed PMID: 19345831; PubMed Central PMCID:
PMC2866651.

685: Mukherjee A, Sarkar A, Saha I, Biswas B, Bhattacharyya PS. Outcomes of
different subgroups of smear-positive retreatment patients under RNTCP in rural
West Bengal, India. Rural Remote Health. 2009 Jan-Mar;9(1):926. Epub 2009 Feb 27.

PubMed PMID: 19260766.

686: Wejse C, Gomes VF, Rabna P, Gustafson P, Aaby P, Lisse IM, Andersen PL,
Glerup H, Sodemann M. Vitamin D as supplementary treatment for tuberculosis: a
double-blind, randomized, placebo-controlled trial. Am J Respir Crit Care Med.
2009 May 1;179(9):843-50. doi: 10.1164/rccm.200804-567OC. Epub 2009 Jan 29.
PubMed PMID: 19179490.

687: Kobashi Y, Oka M. [Long-term observation of pulmonary Mycobacterium avium
complex disease treated with chemotherapy-- following the guidelines for
treatment]. Kekkaku. 2008 Dec;83(12):779-84. Japanese. PubMed PMID: 19172823.

688: Li L, Zheng SH, Chu NH, Xie YG, Yang YZ, Li Q, Wang XM, Yan XL, Tan WG, Miao
ZP, Duanmu HJ. [Effects of two treatment regimens for drug-resistant tuberculosis
in tuberculosis control project areas: a comparative study]. Zhonghua Yi Xue Za
Zhi. 2008 Dec 30;88(48):3387-91. Chinese. PubMed PMID: 19159566.

689: Menter A, Reich K, Gottlieb AB, Bala M, Li S, Hsu MC, Guzzo C, Diels J,
Gelfand JM. Adverse drug events in infliximab-treated patients compared with the
general and psoriasis populations. J Drugs Dermatol. 2008 Dec;7(12):1137-46.
PubMed PMID: 19137767.

690: Martineau AR, Nanzer AM, Satkunam KR, Packe GE, Rainbow SJ, Maunsell ZJ,
Timms PM, Venton TR, Eldridge SM, Davidson RN, Wilkinson RJ, Griffiths CJ.
Influence of a single oral dose of vitamin D(2) on serum 25-hydroxyvitamin D
concentrations in tuberculosis patients. Int J Tuberc Lung Dis. 2009
Jan;13(1):119-25. PubMed PMID: 19105889.

691: Kawada H, Yamazato M, Shinozawa Y, Suzuki K, Otani S, Ouchi M, Miyairi M.
[Achievement of sputum culture negative conversion by minocycline in a case with
extensively drug-resistant pulmonary tuberculosis]. Kekkaku. 2008
Nov;83(11):725-8. Japanese. PubMed PMID: 19086436.

692: Ramirez J, Mason C, Ali J, Lopez FA. Mycobacterium avium complex pulmonary
disease: management options in HIV-negative patients. J La State Med Soc. 2008
Sep-Oct;160(5):248-54; quiz 254, 293. PubMed PMID: 19048978.

693: Matsunaga K, Nagata N, Iwata Y, Kumazoe H, Komori M, Wakamatsu K, Kajiki A,
Kitahara Y. [Treatment outcome of patients with pulmonary tuberculosis before and
after the induction of directly observed therapy (DOT)]. Kekkaku. 2008
Oct;83(10):667-72. Japanese. PubMed PMID: 19048942.

694: Hu Y, Jiang WL, Wang WB, Xu B. [A-cohort study on the standard short-course
chemotherapy program for drug resistant tuberculosis in the rural counties in
Eastern China]. Zhonghua Liu Xing Bing Xue Za Zhi. 2008
Jun;29(6):540-4. Chinese.
PubMed PMID: 19040032.

695: Parrish SC, Myers J, Lazarus A. Nontuberculous mycobacterial pulmonary
infections in Non-HIV patients. Postgrad Med. 2008 Nov;120(4):78-86. doi:
10.3810/pgm.2008.11.1942. Review. PubMed PMID: 19020369.

696: Shah NS, Pratt R, Armstrong L, Robison V, Castro KG, Cegielski JP.
Extensively drug-resistant tuberculosis in the United States, 1993-2007. JAMA.
2008 Nov 12;300(18):2153-60. doi: 10.1001/jama.300.18.2153. PubMed PMID:
19001626.

697: Kobashi Y, Mouri K, Yagi S, Obase Y, Miyashita N, Oka M. Transitional
changes in T-cell responses to Mycobacterium tuberculosis-specific antigens
during treatment. J Infect. 2009 Mar;58(3):197-204. doi:
10.1016/j.jinf.2008.08.009. Epub 2008 Oct 10. PubMed PMID: 18848730.

698: Pajankar S, Khandekar R, Al Amri MA, Al Lawati MR. Factors influencing
sputum smear conversion at one and two months of tuberculosis treatment. Oman Med

J. 2008 Oct;23(4):263-8. PubMed PMID: 22334839; PubMed Central
PMCID: PMC3273921.

699: Shiraishi Y, Katsuragi N, Kita H, Toishi M, Onda T. Experience
with
pulmonary resection for extensively drug-resistant tuberculosis.
Interact
Cardiovasc Thorac Surg. 2008 Dec;7(6):1075-8. doi:
10.1510/icvts.2008.185124.
Epub 2008 Sep 23. PubMed PMID: 18812334.

700: Krapp F, VÃ©liz JC, Cornejo E, Gotuzzo E, Seas C. Bodyweight
gain to predict
treatment outcome in patients with pulmonary tuberculosis in Peru.
Int J Tuberc
Lung Dis. 2008 Oct;12(10):1153-9. PubMed PMID: 18812045.

701: Temple B, Ayakaka I, Ogwang S, Nabanjja H, Kayes S, Nakubulwa
S, Worodria W,
Levin J, Joloba M, Okwera A, Eisenach KD, McNerney R, Elliott AM,
Smith PG,
Mugerwa RD, Ellner JJ, Jones-LÃ³pez EC. Rate and amplification of
drug resistance
among previously-treated patients with tuberculosis in Kampala,
Uganda. Clin
Infect Dis. 2008 Nov 1;47(9):1126-34. doi: 10.1086/592252. PubMed
PMID: 18808360;
PubMed Central PMCID: PMC2883442.

702: Fujino T, Fusegawa H, Nishiumi M, Okubo Y, Kakizaki T, Maejima
K, Sugimori
H. [Epidemiological study on factors affecting the hospitalization
period of
patients with active tuberculosis]. Kekkaku. 2008 Aug;83(8):567-72.
Japanese.
PubMed PMID: 18800648.

703: Zachariae C, MÃ¸rk NJ, Reunala T, Lorentzen H, Falk E, Karvonen
SL,
Johannesson A, ClarÃ©us B, Skov L, MÃ¸rk G, Walker S, Qvitzau S. The
combination of
etanercept and methotrexate increases the effectiveness of treatment
in active
psoriasis despite inadequate effect of methotrexate therapy. Acta
Derm Venereol.
2008;88(5):495-501. doi: 10.2340/00015555-0511. PubMed PMID:
18779890.

704: Casanova-Cardiel LJ, Flores-Barrientos OI, Schabib-Hany M,
Miranda-Ruiz R,

CastaÃ±Ã³n-GonzÃ¡lez JA. [Cosyntropin test in severe active tuberculosis]. Cir Cir.
2008 Jul-Aug;76(4):305-9. Spanish. PubMed PMID: 18778540.

705: MaÃ¢lej S, Belhaoui N, Bourguiba M, Mahouachi R, Chtourou A, Taktak S,
Fennira H, Slim L, Kheder AB, Drira I. [Pulmonary tuberculosis and diabetes. A
retrospective study of 60 patients in Tunisia]. Presse Med. 2009 Jan;38(1):20-4.
doi: 10.1016/j.lpm.2008.05.011. Epub 2008 Sep 3. French. PubMed PMID: 18771896.

706: Park SK, Kim JH, Kang H, Cho JS, Smego RA Jr. Pulmonary resection combined
with isoniazid- and rifampin-based drug therapy for patients with
multidrug-resistant and extensively drug-resistant tuberculosis. Int J Infect
Dis. 2009 Mar;13(2):170-5. doi: 10.1016/j.ijid.2008.06.001. Epub 2008 Sep 2.
PubMed PMID: 18768342.

707: GorÃs-Pereiras A, FernÃ¡ndez-Villar A, ChouciÃ±o-Garrido N, Otero-Baamonde M,
VÃ¡zquez-Gallardo R. [Factors predicting new tuberculosis infections and
tuberculin conversions in a contact tracing system]. Enferm Clin. 2008
Jul-Aug;18(4):183-9. Spanish. PubMed PMID: 18724914.

708: van der Heijde D, Schiff MH, Sieper J, Kivitz AJ, Wong RL, Kupper H,
Dijkmans BA, Mease PJ, Davis JC Jr; ATLAS Study Group. Adalimumab effectiveness
for the treatment of ankylosing spondylitis is maintained for up to 2 years:
long-term results from the ATLAS trial. Ann Rheum Dis. 2009 Jun;68(6):922-9. doi:
10.1136/ard.2007.087270. Epub 2008 Aug 13. PubMed PMID: 18701556; PubMed Central
PMCID: PMC2674550.

709: Fu Y, Chu NH, Yuan SL, Chen W, Wang W, Luo YA, Xiao HP, Zhu LZ. [The effect
of interventional therapy in multimodality treatment on multi-drug resistant
pulmonary tuberculosis]. Zhonghua Jie He He Hu Xi Za Zhi. 2008 Feb;31(2):95-8.
Chinese. PubMed PMID: 18683778.

710: van de Kerkhof PC, Segaert S, Lahfa M, Luger TA, Karolyi Z, Kaszuba A,
Leigheb G, Camacho FM, Forsea D, Zang C, Boussuge MP, Paolozzi L, Wajdula J. Once
weekly administration of etanercept 50 mg is efficacious and well tolerated in
patients with moderate-to-severe plaque psoriasis: a randomized controlled trial
with open-label extension. Br J Dermatol. 2008 Nov;159(5):1177-85. doi:
10.1111/j.1365-2133.2008.08771.x. Epub 2008 Jul 31. PubMed PMID: 18673365.

711: Lei JP, Xiong GL, Hu QF, Li Y, Zong PL, Tu SH, Tu RY. [Immunotherapeutic
efficacy of BCG vaccine in pulmonary tuberculosis and its preventive effect on
multidrug-resistant tuberculosis]. Zhonghua Yu Fang Yi Xue Za Zhi. 2008
Feb;42(2):86-9. Chinese. PubMed PMID: 18642658.

712: Condos R, Hadgiangelis N, Leibert E, Jacquette G, Harkin T, Rom WN. Case
series report of a linezolid-containing regimen for extensively drug-resistant
tuberculosis. Chest. 2008 Jul;134(1):187-92. doi: 10.1378/chest.07-1988. PubMed
PMID: 18628223.

713: Banerjee R, Allen J, Westenhouse J, Oh P, Elms W, Desmond E, Nitta A, Royce
S, Flood J. Extensively drug-resistant tuberculosis in california, 1993-2006.
Clin Infect Dis. 2008 Aug 15;47(4):450-7. doi: 10.1086/590009. PubMed PMID:
18616396.

714: Prasad R, Verma SK, Sahai S, Kumar S, Jain A. Efficacy and safety of
kanamycin, ethionamide, PAS and cycloserine in multidrug-resistant pulmonary
tuberculosis patients. Indian J Chest Dis Allied Sci. 2006 Jul-Sep;48(3):183-6.
PubMed PMID: 18610675.

715: Bawri S, Ali S, Phukan C, Tayal B, Baruwa P. A study of sputum conversion in
new smear positive pulmonary tuberculosis cases at the monthly intervals of 1, 2
& 3 month under directly observed treatment, short course (dots) regimen. Lung

India. 2008 Jul;25(3):118-23. doi: 10.4103/0970-2113.44122. PubMed PMID:
20165663; PubMed Central PMCID: PMC2822329.

716: Koh WJ, Kim YH, Kwon OJ, Choi YS, Kim K, Shim YM, Kim J. Surgical treatment
of pulmonary diseases due to nontuberculous mycobacteria. J Korean Med Sci. 2008
Jun;23(3):397-401. doi: 10.3346/jkms.2008.23.3.397. PubMed PMID: 18583873; PubMed
Central PMCID: PMC2526522.

717: Aggarwal D, Mohapatra PR. Sputum conversion at the end of intensive phase
treatment of pulmonary tuberculosis patients with diabetes mellitus or HIV
infection. Indian J Med Res. 2008 Apr;127(4):408; author reply 408-9. PubMed
PMID: 18577800.

718: Pheiffer C, Carroll NM, Beyers N, Donald P, Duncan K, Uys P, van Helden P.
Time to detection of Mycobacterium tuberculosis in BACTEC systems as a viable
alternative to colony counting. Int J Tuberc Lung Dis. 2008 Jul;12(7):792-8.
PubMed PMID: 18544206.

719: Grahmann PR, Braun RK. A new protocol for multiple inhalation of IFN-gamma
successfully treats MDR-TB: a case study. Int J Tuberc Lung Dis. 2008
Jun;12(6):636-44. PubMed PMID: 18492330.

720: Swaminathan S, Deivanayagam CN, Rajasekaran S, Venkatesan P,
Padmapriyadarsini C, Menon PA, Ponnuraja C, Dilip M. Long term follow up of
HIV-infected patients with tuberculosis treated with 6-month intermittent short
course chemotherapy. Natl Med J India. 2008 Jan-Feb;21(1):3-8. PubMed PMID:
18472696.

721: Lovell DJ, Reiff A, Ilowite NT, Wallace CA, Chon Y, Lin SL, Baumgartner SW,
Giannini EH; Pediatric Rheumatology Collaborative Study Group. Safety and
efficacy of up to eight years of continuous etanercept therapy in patients with

juvenile rheumatoid arthritis. Arthritis Rheum. 2008 May;58(5):1496-504. doi:
10.1002/art.23427. PubMed PMID: 18438876.

722: Zaka-Ur-Rehman Z, Jamshaid M, Chaudhry A. Clinical evaluation and monitoring
of adverse effects for fixed multidose combination against single drug therapy in
pulmonary tuberculosis patients. Pak J Pharm Sci. 2008 Apr;21(2):185-94. PubMed
PMID: 18390450.

723: Kaur G, Goel NK, Kumar D, Janmeja AK, Swami HM, Kalia M. Treatment outcomes
of patients placed on treatment under directly observed therapy short-course
(dots). Lung India. 2008 Apr;25(2):75-7. doi: 10.4103/0970-2113.44124. PubMed
PMID: 20165654; PubMed Central PMCID: PMC2822324.

724: Dhingra VK, Rajpal S, Mittal A, Hanif M. Outcome of multi-drug resistant
tuberculosis cases treated by individualized regimens at a tertiary level clinic.
Indian J Tuberc. 2008 Jan;55(1):15-21. PubMed PMID: 18361306.

725: Kuroishi S, Nakamura Y, Hayakawa H, Shirai M, Nakano Y, Yasuda K, Suda T,
Nakamura H, Chida K. Mycobacterium avium complex disease: prognostic implication
of high-resolution computed tomography findings. Eur Respir J. 2008
Jul;32(1):147-52. doi: 10.1183/09031936.00074207. Epub 2008 Mar 5. PubMed PMID:
18321941.

726: Lopes LK, Teles SA, Souza AC, Rabahi MF, Tipple AF. Tuberculosis risk among
nursing professionals from Central Brazil. Am J Infect Control. 2008
Mar;36(2):148-51. doi: 10.1016/j.ajic.2007.01.013. PubMed PMID: 18313518.

727: Suzuki A, Nishimura T, Ohtawa S, Mikami J, Kurashima A, Saito M, Mita M.
[Clinical efficacy of kanamycin for Mycobacterium avium complex disease].
Yakugaku Zasshi. 2008 Mar;128(3):451-60. Japanese. PubMed PMID: 18311066.

728: Ziganshina LE, Squire SB. Fluoroquinolones for treating tuberculosis.
Cochrane Database Syst Rev. 2008 Jan 23;(1):CD004795. doi:
10.1002/14651858.CD004795.pub3. Review. Update in: Cochrane Database Syst Rev.
2013;6:CD004795. PubMed PMID: 18254061.

729: Katiyar SK, Bihari S, Prakash S, Mamtani M, Kulkarni H. A randomised
controlled trial of high-dose isoniazid adjuvant therapy for multidrug-resistant
tuberculosis. Int J Tuberc Lung Dis. 2008 Feb;12(2):139-45. PubMed PMID:
18230245.

730: Rustomjee R, Lienhardt C, Kanyok T, Davies GR, Levin J, Mthiyane T, Reddy C,
Sturm AW, Sirgel FA, Allen J, Coleman DJ, Fourie B, Mitchison DA; Gatifloxacin
for TB (OFLOTUB) study team. A Phase II study of the sterilising activities of
ofloxacin, gatifloxacin and moxifloxacin in pulmonary tuberculosis. Int J Tuberc
Lung Dis. 2008 Feb;12(2):128-38. PubMed PMID: 18230244.

731: Chang KC, Leung CC, Yew WW, Kam KM, Yip CW, Ma CH, Tam CM, Leung EC, Law WS,
Leung WM. Peak plasma rifampicin level in tuberculosis patients with slow culture
conversion. Eur J Clin Microbiol Infect Dis. 2008 Jun;27(6):467-72. doi:
10.1007/s10096-007-0454-6. Epub 2008 Jan 24. PubMed PMID: 18214560.

732: Ciofu O, Lee B, Johannesson M, Hermansen NO, Meyer P, HÃ¸iby N; Scandinavian
Cystic Fibrosis Study Consortium. Investigation of the algT operon sequence in
mucoid and non-mucoid Pseudomonas aeruginosa isolates from 115 Scandinavian
patients with cystic fibrosis and in 88 in vitro non-mucoid revertants.
Microbiology. 2008 Jan;154(Pt 1):103-13. doi: 10.1099/mic.0.2007/010421-0. PubMed
PMID: 18174130.

733: Jeon CY, Hwang SH, Min JH, Prevots DR, Goldfeder LC, Lee H, Eum SY, Jeon DS,
Kang HS, Kim JH, Kim BJ, Kim DY, Holland SM, Park SK, Cho SN, Barry CE 3rd, Via
LE. Extensively drug-resistant tuberculosis in South Korea: risk factors and

treatment outcomes among patients at a tertiary referral hospital. Clin Infect
Dis. 2008 Jan 1;46(1):42-9. doi: 10.1086/524017. PubMed PMID: 18171212.

734: Banu Rekha VV, Balasubramanian R, Swaminathan S, Ramachandran R, Rahman F,
Sundaram V, Thyagarajan K, Selvakumar N, Adhilakshmi AR, Iliayas S, Narayanan PR.
Sputum conversion at the end of intensive phase of Category-1 regimen in the
treatment of pulmonary tuberculosis patients with diabetes mellitus or HIV
infection: An analysis of risk factors. Indian J Med Res. 2007 Nov;126(5):452-8.
PubMed PMID: 18160750.

735: MehiÄ‡ B. The role of inhalatory corticosteroids and long acting beta(2)
agonists in the treatment of patients admitted to hospital due to acute
exacerbations of chronic obstructive pulmonary disease (AECOPD). Bosn J Basic Med
Sci. 2007 Nov;7(4):352-7. PubMed PMID: 18039195; PubMed Central PMCID:
PMC5728610.

736: Dembele SM, Ouedraogo HZ, Combary A, Saleri N, Macq J, Dujardin B.
Conversion rate at two-month follow-up of smear-positive tuberculosis patients in
Burkina Faso. Int J Tuberc Lung Dis. 2007 Dec;11(12):1339-44. PubMed PMID:
18034956.

737: Picon PD, Bassanesi SL, Caramori ML, Ferreira RL, Jarczewski CA, Vieira PR.
Risk factors for recurrence of tuberculosis. J Bras Pneumol. 2007
Sep-Oct;33(5):572-8. English, Portuguese. PubMed PMID: 18026656.

738: Sasaki Y, Yamagishi F, Yagi T, Hashimoto T, Bekku R, Kawasaki T, Shinozaki
A. [Evaluation of care for elderly pulmonary tuberculosis patients]. Kekkaku.
2007 Oct;82(10):733-9. Japanese. PubMed PMID: 18018597.

739: Takahashi K, Hasegawa Y, Abe T, Yamamoto T, Nakashima K, Imaizumi K,
Shimokata K. SLC11A1 (formerly NRAMP1) polymorphisms associated with

multidrug-resistant tuberculosis. Tuberculosis (Edinb). 2008
Jan;88(1):52-7. Epub
2007 Oct 18. PubMed PMID: 17950034.

740: Hsieh CJ, Lin LC, Kuo BI, Chiang CH, Su WJ, Shih JF. Exploring the efficacy
of a case management model using DOTS in the adherence of patients with pulmonary
tuberculosis. J Clin Nurs. 2008 Apr;17(7):869-75. PubMed PMID: 17850292.

741: Ye JJ, Wu TS, Chiang PC, Lee MH. Factors that affect sputum conversion and
treatment outcome in patients with Mycobacterium avium-intracellulare complex
pulmonary disease. J Microbiol Immunol Infect. 2007 Aug;40(4):342-8. PubMed PMID:
17712469.

742: Schreiber S, Khaliq-Kareemi M, Lawrance IC, Thomsen OÃ˜, Hanauer SB, McColm
J, Bloomfield R, Sandborn WJ; PRECISE 2 Study Investigators. Maintenance therapy
with certolizumab pegol for Crohn's disease. N Engl J Med. 2007 Jul
19;357(3):239-50. Erratum in: N Engl J Med. 2007 Sep 27;357(13):1357. PubMed
PMID: 17634459.

743: Hill PC, Brookes RH, Fox A, Jackson-Sillah D, Jeffries DJ, Lugos MD, Donkor
SA, Adetifa IM, de Jong BC, Aiken AM, Adegbola RA, McAdam KP. Longitudinal
assessment of an ELISPOT test for Mycobacterium tuberculosis infection. PLoS Med.
2007 Jun;4(6):e192. PubMed PMID: 17564487; PubMed Central PMCID: PMC1891317.

744: Mathur ML. Role of vitamin A supplementation in the treatment of
tuberculosis. Natl Med J India. 2007 Jan-Feb;20(1):16-21. Review. PubMed PMID:
17557517.

745: Tamura A, Hebisawa A, Masuda K, Shimada M, Ichikawa M, Kunogi M, Kaneko Y,
Kawashima M, Suzuki J, Ariga H, Yagi O, Ohshima N, Matsui H, Nagai H, Akagawa S,
Nagayama N, Kawabe Y, Machida K, Kurashima A, Nakajima Y, Yotsumoto H.

[Coexisting lung cancer and active pulmonary mycobacteriosis]. Nihon Kokyuki
Gakkai Zasshi. 2007 May;45(5):382-93. Japanese. PubMed PMID: 17554981.

746: GullÃ³n Blanco JA, SuÃ¡rez Toste I, Lecuona FernÃ¡ndez M, Galindo Morales R,
FernÃ¡ndez Alvarez R, Rubinos Cuadrado G, Medina GonzÃ¡lvez A, GonzÃ¡lez MartÃn IJ.
[Tobacco smoking and sputum smear conversion in pulmonary tuberculosis]. Med Clin
(Barc). 2007 Apr 21;128(15):565-8. Spanish. PubMed PMID: 17462193.

747: Babb C, van der Merwe L, Beyers N, Pheiffer C, Walzl G, Duncan K, van Helden
P, Hoal EG. Vitamin D receptor gene polymorphisms and sputum conversion time in
pulmonary tuberculosis patients. Tuberculosis (Edinb). 2007 Jul;87(4):295-302.
Epub 2007 Apr 20. PubMed PMID: 17449323.

748: Wiysonge CS, Ntsekhe M, Gumedze F, Sliwa K, Blackett KN, Commerford PJ,
Volmink JA, Mayosi BM. Contemporary use of adjunctive corticosteroids in
tuberculous pericarditis. Int J Cardiol. 2008 Mar 14;124(3):388-90. Epub 2007 Apr
18. PubMed PMID: 17445921.

749: Kurashima A. [Perspective of pulmonary MAC infection treatment]. Kekkaku.
2007 Mar;82(3):195-9. Review. Japanese. PubMed PMID: 17444124.

750: Feng YL, Ling CQ, Chen Z, Li B, Gu W. [Ginsenosides and dexamethasone in
managing the liver injury and renal function after transcatheter arterial
chemoembolization for hepatic carcinoma patient]. Zhonghua Zhong Liu Za Zhi. 2006
Nov;28(11):844-7. Chinese. PubMed PMID: 17416007.

751: FortÃºn J, MartÃn-DÃ¡vila P, Molina A, Navas E, Hermida JM, Cobo J,
GÃ³mez-Mampaso E, Moreno S. Sputum conversion among patients with pulmonary
tuberculosis: are there implications for removal of respiratory isolation? J
Antimicrob Chemother. 2007 Apr;59(4):794-8. PubMed PMID: 17392354.

752: Wada M, Mizutani S, Nakajima Y, Ito K, Mitarai S, Hoshino H, Okumura M,
Yoshiyama T, Ogata H. [A case of multidrug-resistant pulmonary tuberculosis cured
by the regimen including thiacetazone]. Kekkaku. 2007 Jan;82(1):33-7. Japanese.
PubMed PMID: 17310780.

753: Thwaites GE, Macmullen-Price J, Tran TH, Pham PM, Nguyen TD, Simmons CP,
White NJ, Tran TH, Summers D, Farrar JJ. Serial MRI to determine the effect of
dexamethasone on the cerebral pathology of tuberculous meningitis: an
observational study. Lancet Neurol. 2007 Mar;6(3):230-6. PubMed PMID: 17303529;
PubMed Central PMCID: PMC4333204.

754: van de Beek D. Brain teasing effect of dexamethasone. Lancet Neurol. 2007
Mar;6(3):203-4. PubMed PMID: 17303522.

755: Hill PC, Jeffries DJ, Brookes RH, Fox A, Jackson-Sillah D, Lugos MD, Donkor
SA, de Jong BC, Corrah T, Adegbola RA, McAdam KP. Using ELISPOT to expose false
positive skin test conversion in tuberculosis contacts. PLoS One. 2007 Jan
31;2(1):e183. PubMed PMID: 17264885; PubMed Central PMCID: PMC1779806.

756: GÃ¼ler M, Unsal E, Dursun B, Aydln O, Capan N. Factors influencing sputum
smear and culture conversion time among patients with new case pulmonary
tuberculosis. Int J Clin Pract. 2007 Feb;61(2):231-5. PubMed PMID: 17166185.

757: Shmelev EI, Kunicina YL. Comparison of fenspiride with beclomethasone as
adjunctive anti-inflammatory treatment in patients with chronic obstructive
pulmonary disease. Clin Drug Investig. 2006;26(3):151-9. PubMed PMID: 17163246.

758: Yoshinaga Y, Kanamori T, Ota Y, Miyoshi T, Kagawa H, Yamamura M. Clinical
characteristics of Mycobacterium tuberculosis infection among rheumatoid

arthritis patients. Mod Rheumatol. 2004;14(2):143-8. PubMed PMID:
17143664.

759: Van Deun A, Aung KJ, Hamid Salim MA, Ali MA, Naha MS, Das PK,
Hossain MA,
Declercq E. Extension of the intensive phase reduces unfavourable
outcomes with
the 8-month thioacetazone regimen. Int J Tuberc Lung Dis. 2006
Nov;10(11):1255-61. PubMed PMID: 17131785.

760: Srisaenpang S, Pinitsoontorn S, Singhasivanon P, Kitayaporn D,
Kaewkungwal
J, Tatsanavivat P, Patjanasoontorn B, Reechaipichitkul W,
Thiratakulpisan J,
Srinakarin J, Srisaenpang P, Thinkamrop B, Apinyanurak C, Chindawong
BO. Missed
appointments at a tuberculosis clinic increased the risk of clinical
treatment
failure. Southeast Asian J Trop Med Public Health. 2006
Mar;37(2):345-50. PubMed
PMID: 17124997.

761: Reuter H, Burgess LJ, Louw VJ, Doubell AF. Experience with
adjunctive
corticosteroids in managing tuberculous pericarditis. Cardiovasc J S
Afr. 2006
Sep-Oct;17(5):233-8. PubMed PMID: 17117227.

762: Kubitza D, Becka M, Mueck W, Zuehlsdorf M. Rivaroxaban (BAY 59-
7939)--an
oral, direct Factor Xa inhibitor--has no clinically relevant
interaction with
naproxen. Br J Clin Pharmacol. 2007 Apr;63(4):469-76. Epub 2006 Nov
13. PubMed
PMID: 17100983; PubMed Central PMCID: PMC2203251.

763: Jubelt B. Dexamethasone for the treatment of tuberculous
meningitis in
adolescents and adults. Curr Neurol Neurosci Rep. 2006 Nov;6(6):451-
2. PubMed
PMID: 17074278.

764: Zhu LZ, Fu Y, Chu NH, Ye ZZ, Xiao HP, Wang W, Yuan SL, Zhang X,
Luo YA, Ma
LP. [A controlled clinical trial of long course chemotherapy
regimens containing
rifabutin in the treatment of multi-drug resistant pulmonary
tuberculosis].
Zhonghua Jie He He Hu Xi Za Zhi. 2006 Aug;29(8):520-3. Chinese.
PubMed PMID:

17074263.

765: Okutan O, Kartaloglu Z, Kunter E, Apaydin M, Ilvan A. Relation of serum
adenosine deaminase (ADA) levels with sputum smear conversion in patients with
pulmonary tuberculosis. Ann Saudi Med. 2006 Sep-Oct;26(5):406-7. PubMed PMID:
17019093; PubMed Central PMCID: PMC6074108.

766: Kobashi Y, Matsushima T. The microbiological and clinical effects of
combined therapy according to guidelines on the treatment of pulmonary
Mycobacterium avium complex disease in Japan - including a follow-up study.
Respiration. 2007;74(4):394-400. Epub 2006 Sep 5. PubMed PMID: 16954651.

767: Paralija B. [The effectivness of direct observed treatment short course
(DOTS) in tuberculosis treatment]. Med Arh. 2006;60(5):287-91. Bosnian. PubMed
PMID: 16944727.

768: Sprinson JE, Lawton ES, Porco TC, Flood JM, Westenhouse JL. Assessing the
validity of tuberculosis surveillance data in California. BMC Public Health. 2006
Aug 25;6:217. PubMed PMID: 16930492; PubMed Central PMCID: PMC1617097.

769: Wang W, Xiao H, Lu L. Case-control retrospective study of pulmonary
tuberculosis in heroin-abusing patients in China. J Psychoactive Drugs. 2006
Jun;38(2):203-5. PubMed PMID: 16903459.

770: Gopi PG, Chandrasekaran V, Subramani R, Santha T, Thomas A, Selvakumar N,
Narayanan PR. Association of conversion & cure with initial smear grading among
new smear positive pulmonary tuberculosis patients treated with Category I
regimen. Indian J Med Res. 2006 Jun;123(6):807-14. PubMed PMID: 16885603.

771: Griffith DE, Brown-Elliott BA, Langsjoen B, Zhang Y, Pan X, Girard W, Nelson

K, Caccitolo J, Alvarez J, Shepherd S, Wilson R, Graviss EA, Wallace RJ Jr.
Clinical and molecular analysis of macrolide resistance in Mycobacterium avium
complex lung disease. Am J Respir Crit Care Med. 2006 Oct 15;174(8):928-34. Epub
2006 Jul 20. PubMed PMID: 16858014.

772: Chokhani R, Pathak V. DOTS centre at a tertiary care teaching hospital.
Nepal Med Coll J. 2006 Mar;8(1):19-21. PubMed PMID: 16827084.

773: Verster JC, Veldhuijzen DS, Volkerts ER. Effects of an opioid
(oxycodone/paracetamol) and an NSAID (bromfenac) on driving ability, memory
functioning, psychomotor performance, pupil size, and mood. Clin J Pain. 2006
Jun;22(5):499-504. PubMed PMID: 16772806.

774: Kobashi Y, Matsushima T, Oka M. A double-blind randomized study of
aminoglycoside infusion with combined therapy for pulmonary Mycobacterium avium
complex disease. Respir Med. 2007 Jan;101(1):130-8. Epub 2006 Jun 5. PubMed PMID:
16750618.

775: Tada A, Kawata N, Shibayama T, Takahashi S, Hirano A, Kimura G, Takeuchi M,
Okada C, Soda R, Takahashi K. [In vitro antituberculous activity of ofloxacin and
levofloxacin against multidrug-resistant tuberculosis and clinical outcomes].
Kekkaku. 2006 Apr;81(4):337-44. Japanese. PubMed PMID: 16715942.

776: Burman WJ, Goldberg S, Johnson JL, Muzanye G, Engle M, Mosher AW, Choudhri
S, Daley CL, Munsiff SS, Zhao Z, Vernon A, Chaisson RE. Moxifloxacin versus
ethambutol in the first 2 months of treatment for pulmonary tuberculosis. Am J
Respir Crit Care Med. 2006 Aug 1;174(3):331-8. Epub 2006 May 4. PubMed PMID:
16675781.

777: Holtz TH, Sternberg M, Kammerer S, Laserson KF, Riekstina V, Zarovska E,
Skripconoka V, Wells CD, Leimane V. Time to sputum culture conversion in

multidrug-resistant tuberculosis: predictors and relationship to treatment
outcome. Ann Intern Med. 2006 May 2;144(9):650-9. PubMed PMID: 16670134.

778: Vlassov VV, MacLehose HG. Low level laser therapy for treating tuberculosis.
Cochrane Database Syst Rev. 2006 Apr 19;(2):CD003490. Review. PubMed PMID:
16625582.

779: Monteon ML, Arcari CM, Remington PL, Kanarek MS, Oemig TV. Tuberculosis
cases in Wisconsin: documentation of treatment improvement and completion of
treatment, 2000-2002. J Public Health Manag Pract. 2006 May-Jun;12(3):254-61.
PubMed PMID: 16614561.

780: Silverman M, Sheffer AL, DÃ-az PV, Lindberg B. Safety and tolerability of
inhaled budesonide in children in the Steroid Treatment As Regular Therapy in
early asthma (START) trial. Pediatr Allergy Immunol. 2006 May;17 Suppl 17:14-20.
PubMed PMID: 16573704.

781: Tao Y, Ninomiya K, Miyazaki M, Iwanaga T, Toshihiko I, Ootsu T, Takigawa S,
Kawakami K, Kawabata M, Higashi K. [Clinical analysis of cases with
multidrug-resistant tuberculosis--inpatients at National Hospitals in Kyushu
between 1998 and 2003]. Kekkaku. 2006 Jan;81(1):1-5. Japanese. PubMed PMID:
16479994.

782: Nursyam EW, Amin Z, Rumende CM. The effect of vitamin D as supplementary
treatment in patients with moderately advanced pulmonary tuberculous lesion. Acta
Med Indones. 2006 Jan-Mar;38(1):3-5. PubMed PMID: 16479024.

783: Davydov L, Ebert SC, Restino M, Gardner M, Bedenkop G, Uchida KM, Bertino JS
Jr. Prospective evaluation of the treatment and outcome of community-acquired
pneumonia according to the Pneumonia Severity Index in VHA hospitals. Diagn
Microbiol Infect Dis. 2006 Apr;54(4):267-75. Epub 2006 Feb 8. PubMed PMID:

16466891.

784: Singla R, Khan N, Al-Sharif N, Ai-Sayegh MO, Shaikh MA, Osman MM. Influence
of diabetes on manifestations and treatment outcome of pulmonary TB patients. Int
J Tuberc Lung Dis. 2006 Jan;10(1):74-9. PubMed PMID: 16466041.

785: Francois Venter WD, Panz VR, Feldman C, Joffe BI. Adrenocortical function in
hospitalised patients with active pulmonary tuberculosis receiving a
rifampicin-based regimen -- a pilot study. S Afr Med J. 2006 Jan;96(1):62-6.
PubMed PMID: 16440115.

786: Schiff MH, Burmester GR, Kent JD, Pangan AL, Kupper H, Fitzpatrick SB,
Donovan C. Safety analyses of adalimumab (HUMIRA) in global clinical trials and
US postmarketing surveillance of patients with rheumatoid arthritis. Ann Rheum
Dis. 2006 Jul;65(7):889-94. Epub 2006 Jan 26. PubMed PMID: 16439435; PubMed
Central PMCID: PMC1798196.

787: Fujikane T, Fujiuchi S, Yamazaki Y, Sato M, Yamamoto Y, Takeda A, Nishigaki
Y, Fujita Y, Shimizu T. Efficacy and outcomes of clarithromycin treatment for
pulmonary MAC disease. Int J Tuberc Lung Dis. 2005 Nov;9(11):1281-7. PubMed PMID:
16333938.

788: Mikami A, Toyota E, Ishizuka N, Morino E, Naka G, Yoshizawa A, Houjo M,
Kawana A, Kobayashi N, Keicho N, Kudo K. [Impact of the change in criteria for
hospital discharge of patients with tuberculosis based on the duration of
hospitalization]. Kekkaku. 2005 Oct;80(10):631-6. Japanese. PubMed PMID:
16296390.

789: SchÃ¶n T, Wolday D, Elias D, Melese E, Moges F, Tessema T, Stendahl O,
Sundqvist T, Britton S. Kinetics of sedimentation rate, viral load and TNF-alpha
in relation to HIV co-infection in tuberculosis. Trans R Soc Trop Med Hyg. 2006
May;100(5):483-8. Epub 2005 Oct 20. PubMed PMID: 16242741.

790: Fairall LR, Zwarenstein M, Bateman ED, Bachmann M, Lombard C, Majara BP,
Joubert G, English RG, Bheekie A, van Rensburg D, Mayers P, Peters AC, Chapman
RD. Effect of educational outreach to nurses on tuberculosis case detection and
primary care of respiratory illness: pragmatic cluster randomised controlled
trial. BMJ. 2005 Oct 1;331(7519):750-4. Erratum in: BMJ. 2005 Nov
12;331(7525):1120. Myers, Pat [corrected to Mayers, Pat]. PubMed PMID: 16195293;
PubMed Central PMCID: PMC1239979.

791: Range N, Andersen AB, Magnussen P, Mugomela A, Friis H. The effect of
micronutrient supplementation on treatment outcome in patients with pulmonary
tuberculosis: a randomized controlled trial in Mwanza, Tanzania. Trop Med Int
Health. 2005 Sep;10(9):826-32. PubMed PMID: 16135188.

792: Keus K, Houston S, Melaku Y, Burling S. Field research in humanitarian
medical programmes. Treatment of a cohort of tuberculosis patients using the
Manyatta regimen in a conflict zone in South Sudan. Trans R Soc Trop Med Hyg.
2003 Nov-Dec;97(6):614-8. PubMed PMID: 16134258.

793: Ziganshina LE, Vizel AA, Squire SB. Fluoroquinolones for treating
tuberculosis. Cochrane Database Syst Rev. 2005 Jul 20;(3):CD004795. Review.
Update in: Cochrane Database Syst Rev. 2008;(1):CD004795. PubMed PMID: 16034951.

794: Handley DA, Morley J, Vaickus L. Levalbuterol hydrochloride. Expert Opin
Investig Drugs. 1998 Dec;7(12):2027-41. PubMed PMID: 15991945.

795: Deoskar RB, Sengupta B, Rajan KE, Barthwal MS, Falleiro J, Sharma SK. Study
of Drug Resistant Pulmonary Tuberculosis. Med J Armed Forces India. 2005
Jul;61(3):245-8. doi: 10.1016/S0377-1237(05)80164-9. Epub 2011 May 30. PubMed
PMID: 27407770; PubMed Central PMCID: PMC4925639.

796: Chambers HF, Turner J, Schecter GF, Kawamura M, Hopewell PC. Imipenem for
treatment of tuberculosis in mice and humans. Antimicrob Agents Chemother. 2005
Jul;49(7):2816-21. PubMed PMID: 15980354; PubMed Central PMCID: PMC1168716.

797: Sherwood JT, Mitchell JD, Pomerantz M. Completion pneumonectomy for chronic
mycobacterial disease. J Thorac Cardiovasc Surg. 2005 Jun;129(6):1258-65. PubMed
PMID: 15942565.

798: LoBue PA, Moser KS. Isoniazid- and rifampin-resistant tuberculosis in San
Diego County, California, United States, 1993-2002. Int J Tuberc Lung Dis. 2005
May;9(5):501-6. PubMed PMID: 15875920.

799: Nagai H, Inagaki T, Toyoda E, Kawabe Y, Fujiwara K, Masuyama H, Takahashi S.
[Tuberculosis and human rights]. Kekkaku. 2005 Jan;80(1):31-45. Japanese. PubMed
PMID: 15839061.

800: Taga S, Ogawa K, Nakagawa T, Tano M. [The clinical study on efficacy of
clarithromycin, levofloxacin, and streptomycin for pulmonary Mycobacterium
avium-intracellulare complex infection]. Kekkaku. 2005 Jan;80(1):1-7. Japanese.
PubMed PMID: 15839056.

801: Ige OM, Sogaolu OM, Odaibo GN, Olaleye OD. Evaluation of modified short
course chemotherapy in active pulmonary tuberculosis patients with human
immunodeficiency virus infection in University College Hospital, Ibadan,
Nigeria--a preliminary report. Afr J Med Med Sci. 2004 Sep;33(3):259-62. PubMed
PMID: 15819474.

802: Takahara M. [Clinical evaluation of causes of death in patients with
pulmonary tuberculosis]. Kekkaku. 2004 Dec;79(12):711-6. Japanese. PubMed PMID:
15782616.

803: Abal AT, Jayakrishnan B, Parwer S, El Shamy A, Abahussain E, Sharma PN.
Effect of cigarette smoking on sputum smear conversion in adults with active
pulmonary tuberculosis. Respir Med. 2005 Apr;99(4):415-20. PubMed PMID: 15763447.

804: Niazi AD, Al-Delaimi AM. Impact of community participation on treatment
outcomes and compliance of DOTS patients in Iraq. East Mediterr Health J. 2003
Jul;9(4):709-17. PubMed PMID: 15748068.

805: Mohan A, Nassir H, Niazi A. Does routine home visiting improve the return
rate and outcome of DOTS patients who delay treatment? East Mediterr Health J.
2003 Jul;9(4):702-8. PubMed PMID: 15748067.

806: Mayanja-Kizza H, Jones-Lopez E, Okwera A, Wallis RS, Ellner JJ, Mugerwa RD,
Whalen CC; Uganda-Case Western Research Collaboration. Immunoadjuvant
prednisolone therapy for HIV-associated tuberculosis: a phase 2 clinical trial in
Uganda. J Infect Dis. 2005 Mar 15;191(6):856-65. Epub 2005 Feb 8. PubMed PMID:
15717259; PubMed Central PMCID: PMC4515766.

807: Marras TK. Dexamethasone for tuberculous meningitis. N Engl J Med. 2005 Feb
10;352(6):628-30; author reply 628-30. PubMed PMID: 15706646.

808: Singla R, Singla N, Sarin R, Arora VK. Influence of pre-treatment bacillary
load on treatment outcome of pulmonary tuberculosis patients receiving DOTS under
revised national tuberculosis control programme. Indian J Chest Dis Allied Sci.
2005 Jan-Mar;47(1):19-23. PubMed PMID: 15704711.

809: Murhekar MV, Kolappan C, Gopi PG, Chakraborty AK, Sehgal SC. Tuberculosis
situation among tribal population of Car Nicobar, India, 15 years after intensive
tuberculosis control project and implementation of a national tuberculosis
programme. Bull World Health Organ. 2004 Nov;82(11):836-43. Epub 2004 Dec 14.
PubMed PMID: 15640919; PubMed Central PMCID: PMC2623056.

810: Shirai M, Hayakawa H, Nakano Y, Kuroishi S, Nakano Y, Todate A, Suda T,
Chida K. [Outcome of pulmonary Mycobacterium avium complex (MAC) disease treated
with clarithromycin (CAM)-containing regimens]. Nihon Kokyuki Gakkai Zasshi. 2004
Oct;42(10):875-9. Japanese. PubMed PMID: 15566000.

811: Nix DE, Adam RD, Auclair B, Krueger TS, Godo PG, Peloquin CA.
Pharmacokinetics and relative bioavailability of clofazimine in relation to food,
orange juice and antacid. Tuberculosis (Edinb). 2004;84(6):365-73. PubMed PMID:
15525560.

812: Daniel OJ, Salako AA, Oluwole FA, Alausa OK, Oladapo OT. HIV sero-prevalence
among newly diagnosed adult pulmonary tuberculosis patients in Sagamu. Niger J
Med. 2004 Oct-Dec;13(4):393-7. PubMed PMID: 15523868.

813: Thwaites GE, Nguyen DB, Nguyen HD, Hoang TQ, Do TT, Nguyen TC, Nguyen QH,
Nguyen TT, Nguyen NH, Nguyen TN, Nguyen NL, Nguyen HD, Vu NT, Cao HH, Tran TH,
Pham PM, Nguyen TD, Stepniewska K, White NJ, Tran TH, Farrar JJ. Dexamethasone
for the treatment of tuberculous meningitis in adolescents and adults. N Engl J
Med. 2004 Oct 21;351(17):1741-51. PubMed PMID: 15496623.

814: TobÃ³n A, Paniagua L, Henao M, Arcila V, Maya J, Bedoya F, MuÃ±oz B. [Surgical
treatment of multiresistant lung tuberculosis]. Biomedica. 2004 Jun;24 Supp
1:65-72. Spanish. PubMed PMID: 15495573.

815: Roth DE, Soto G, Arenas F, Bautista CT, Ortiz J, Rodriguez R, Cabrera L,
Gilman RH. Association between vitamin D receptor gene polymorphisms and response
to treatment of pulmonary tuberculosis. J Infect Dis. 2004 Sep 1;190(5):920-7.
Epub 2004 Jul 29. PubMed PMID: 15295697.

816: Elliott AM, Luzze H, Quigley MA, Nakiyingi JS, Kyaligonza S, Namujju PB,

Ducar C, Ellner JJ, Whitworth JA, Mugerwa R, Johnson JL, Okwera A. A randomized,
double-blind, placebo-controlled trial of the use of prednisolone as an adjunct
to treatment in HIV-1-associated pleural tuberculosis. J Infect Dis. 2004 Sep
1;190(5):869-78. Epub 2004 Jul 29. PubMed PMID: 15295690.

817: Ito K, Takahashi M, Yoshiyama T, Wada M, Ogata H. [Multi-drug resistant lung
tuberculosis due to double infection of MDR strain]. Kekkaku. 2004
Jun;79(6):387-90. Japanese. PubMed PMID: 15293752.

818: Strang JI, Nunn AJ, Johnson DA, Casbard A, Gibson DG, Girling DJ. Management
of tuberculous constrictive pericarditis and tuberculous pericardial effusion in
Transkei: results at 10 years follow-up. QJM. 2004 Aug;97(8):525-35. PubMed PMID:
15256610.

819: Moriwaki A, Hagimoto N, Wataya H, Yoshimi M, Nakashima N, Maeyama T, Kuwano
K, Nakanishi Y. [A case of rapidly deteriorated pulmonary aspergillosis with
various clinical manifestations]. Nihon Kokyuki Gakkai Zasshi. 2004
Jun;42(6):491-5. Japanese. PubMed PMID: 15228135.

820: Patel N, Trapathi SB. Improved cure rates in pulmonary tuberculosis category
II (retreatment) with mycobacterium w. J Indian Med Assoc. 2003 Nov;101(11):680,
682. PubMed PMID: 15198421.

821: Condos R, Hull FP, Schluger NW, Rom WN, Smaldone GC. Regional deposition of
aerosolized interferon-gamma in pulmonary tuberculosis. Chest. 2004
Jun;125(6):2146-55. PubMed PMID: 15189935.

822: Schoeman JF, Springer P, van Rensburg AJ, Swanevelder S, Hanekom WA, Haslett
PA, Kaplan G. Adjunctive thalidomide therapy for childhood tuberculous
meningitis: results of a randomized study. J Child Neurol. 2004 Apr;19(4):250-7.
PubMed PMID: 15163089.

823: Ziboh VA, Naguwa S, Vang K, Wineinger J, Morrissey BM, Watnik M, Gershwin

ME. Suppression of leukotriene B4 generation by ex-vivo neutrophils isolated from
asthma patients on dietary supplementation with gammalinolenic acid-containing
borage oil: possible implication in asthma. Clin Dev Immunol. 2004
Mar;11(1):13-21. PubMed PMID: 15154607; PubMed Central PMCID:
PMC2275406.

824: Zheng XM, Li SM, Xing BC. [Short-term effect of treatment protocol utilizing
levofloxacin, pasiniazide and M. Vaccae on multi- drug resistant pulmonary
tuberculosis]. Di Yi Jun Yi Da Xue Xue Bao. 2004 May;24(5):574-5, 578. Chinese.
PubMed PMID: 15151838.

825: Arpaz S, Keskin S, Kiter G, Sezgin N, Budin D, Algan A, CaliÅŸir HC.
[Evaluation of Nazilli Tuberculosis Dispensary activities executed between June
1996-May 2000: Pre-DOT situation]. Tuberk Toraks. 2003;51(3):289-97. Turkish.
PubMed PMID: 15143408.

826: Tariq SM, Tariq S. Empirical treatment for tuberculosis: survey of cases
treated over 2 years in a London area. J Pak Med Assoc. 2004 Feb;54(2):88-95.
PubMed PMID: 15134210.

827: Wallis RS, Kyambadde P, Johnson JL, Horter L, Kittle R, Pohle M, Ducar C,
Millard M, Mayanja-Kizza H, Whalen C, Okwera A. A study of the safety,
immunology, virology, and microbiology of adjunctive etanercept in
HIV-1-associated tuberculosis. AIDS. 2004 Jan 23;18(2):257-64. PubMed PMID:
15075543.

828: Kobashi Y, Matsushima T. Comparison of clinical features in patients with
pulmonary Mycobacterium-avium complex (MAC) disease treated before and after
proposal for guidelines. J Infect Chemother. 2004 Feb;10(1):25-30. PubMed PMID:
14991514.

829: Huang XR, Gao WW, Zhang XX, Wang SM, Pan YX, Ma Y. [A preliminary study on

the definition of resistant breakpoints of ofloxacin and levofloxacin for
Mycobacterium tuberculosis]. Zhonghua Jie He He Hu Xi Za Zhi. 2004 Feb;27(2):84-8. Chinese. PubMed PMID: 14990180.

830: Visweswaraiah NK, Telles S. Randomized trial of yoga as a complementary
therapy for pulmonary tuberculosis. Respirology. 2004 Mar;9(1):96-101. PubMed
PMID: 14982609.

831: Stanford J, Stanford C, Grange J. Immunotherapy with Mycobacterium vaccae in
the treatment of tuberculosis. Front Biosci. 2004 May 1;9:1701-19. Review. PubMed
PMID: 14977580.

832: Goerke C, Matias y Papenberg S, Dasbach S, Dietz K, Ziebach R, Kahl BC, Wolz
C. Increased frequency of genomic alterations in Staphylococcus aureus during
chronic infection is in part due to phage mobilization. J Infect Dis. 2004 Feb
15;189(4):724-34. Epub 2004 Jan 29. PubMed PMID: 14767828.

833: Richardus JH, Withington SG, Anderson AM, Croft RP, Nicholls PG, Van Brakel
WH, Smith WC. Adverse events of standardized regimens of corticosteroids for
prophylaxis and treatment of nerve function impairment in leprosy: results from
the 'TRIPOD' trials. Lepr Rev. 2003 Dec;74(4):319-27. PubMed PMID: 14750577.

834: Sasaki Y, Yamagishi F, Yagi T, Itakura M, Fujikawa A, Kuga M, Ishimaru T. [A
study on pulmonary tuberculosis retreatment cases]. Kekkaku. 2003
Dec;78(12):723-32. Japanese. Erratum in: Kekkaku. 2004 Apr;79(4):43. PubMed PMID:
14733112.

835: Hoheisel G, Vogtmann M, Chan KS, Luk WK, Chan CH. [Pleuritis tuberculosa -
therapeutic value of repeated chest tapping]. Pneumologie. 2004 Jan;58(1):23-7.
German. PubMed PMID: 14732896.

836: [Pneumoconiosis and mycobacterial infection]. Kekkaku. 2003
Nov;78(11):711-5. Japanese. PubMed PMID: 14672049.

837: Long R, Bochar K, Chomyc S, Talbot J, Barrie J, Kunimoto D, Tilley P.
Relative versus absolute noncontagiousness of respiratory tuberculosis on
treatment. Infect Control Hosp Epidemiol. 2003 Nov;24(11):831-8. PubMed PMID:
14649771.

838: Saito W, Nagayama N, Miyamoto M, Hara H, Suzuki J, Masuda K, Baba M, Tamura
A, Nagai H, Akagawa S, Kawabe Y, Machida K, Kurashima A, Yotsumoto H.
[Characteristics and treatment outcomes of INH-resistant or RFP-resistant
tuberculosis]. Kekkaku. 2003 Oct;78(10):611-7. Japanese. PubMed PMID: 14621568.

839: Salihu HM, Aliyu MH, Ratard R, Pierre-Louis BJ. Characteristics associated
with reported sputum culture conversion in the era of re-emergent Mycobacterium
tuberculosis in the State of North Carolina, 1993-1998. Int J Tuberc Lung Dis.
2003 Nov;7(11):1070-6. PubMed PMID: 14598967.

840: Griffith DE, Brown-Elliott BA, Wallace RJ Jr. Thrice-weekly
clarithromycin-containing regimen for treatment of Mycobacterium kansasii lung
disease: results of a preliminary study. Clin Infect Dis. 2003 Nov
1;37(9):1178-82. Epub 2003 Oct 1. PubMed PMID: 14557961.

841: Field SK, Cowie RL. Treatment of Mycobacterium avium-intracellulare complex
lung disease with a macrolide, ethambutol, and clofazimine. Chest. 2003
Oct;124(4):1482-6. PubMed PMID: 14555583.

842: Yew WW, Chan CK, Leung CC, Chau CH, Tam CM, Wong PC, Lee J. Comparative
roles of levofloxacin and ofloxacin in the treatment of multidrug-resistant
tuberculosis: preliminary results of a retrospective study from Hong Kong. Chest.
2003 Oct;124(4):1476-81. PubMed PMID: 14555582.

843: Yang H, Guo K, Shen W, Li Y, Dai M. [Clinical observation of the treatment

of 654-2 injection and "ganxian tui huang recipe" on liver cirrhosis with
intractable jaundice]. Zhong Yao Cai. 2003 May;26(5):385-7. Chinese. PubMed PMID:
14535021.

844: Pedral-Sampaio DB, Netto EM, Brites C, Bandeira AC, Guerra C, Barberin MG,
BadarÃ³ R. Use of Rhu-GM-CSF in pulmonary tuberculosis patients: results of a
randomized clinical trial. Braz J Infect Dis. 2003 Aug;7(4):245-52. PubMed PMID:
14533985.

845: Chu NH, Zhu LZ, Yie ZZ, Yuan SL, Wang JY, Xu JL, Ma LP. [A controlled
clinical study on the efficacy of recombinant human interleukin-2 in the
treatment of pulmonary tuberculosis]. Zhonghua Jie He He Hu Xi Za Zhi. 2003
Sep;26(9):548-51. Chinese. PubMed PMID: 14521759.

846: Chen QL, Chen L, Yin JJ. [A study on the clinical efficacy of a combination
regimen with levofloxacin and capreomycin in the treatment of multi-drug
resistant pulmonary tuberculosis]. Zhonghua Jie He He Hu Xi Za Zhi. 2003
Aug;26(8):454-7. Chinese. PubMed PMID: 14505519.

847: Frieden TR, Khatri GR. Impact of national consultants on successful
expansion of effective tuberculosis control in India. Int J Tuberc Lung Dis. 2003
Sep;7(9):837-41. PubMed PMID: 12971666.

848: Miti S, Mfungwe V, Reijer P, Maher D. Integration of tuberculosis treatment
in a community-based home care programme for persons living with HIV/AIDS in
Ndola, Zambia. Int J Tuberc Lung Dis. 2003 Sep;7(9 Suppl 1):S92-8. PubMed PMID:
12971660.

849: Salaniponi FM, Gausi F, Mphasa N, Nyirenda TE, Kwanjana JH, Harries AD.
Decentralisation of treatment for patients with tuberculosis in Malawi: moving
from research to policy and practice. Int J Tuberc Lung Dis. 2003 Sep;7(9 Suppl

1):S38-47. PubMed PMID: 12971653.

850: Arora VK, Singla N, Sarin R. Profile of geriatric patients under DOTS in
Revised National Tuberculosis Control Programme. Indian J Chest Dis Allied Sci.
2003 Oct-Dec;45(4):231-5. PubMed PMID: 12962456.

851: Yokoyama T, Rikimaru T, Gohara R, Sueyasu Y, Aizawa H. [Tuberculosis in
elderly]. Kekkaku. 2003 Jul;78(7):479-82. Japanese. PubMed PMID: 12931644.

852: Kobashi Y, Matsushima T. The effect of combined therapy according to the
guidelines for the treatment of Mycobacterium avium complex pulmonary disease.
Intern Med. 2003 Aug;42(8):670-5. PubMed PMID: 12924489.

853: AlsedÃ M, Godoy P. [Study investigating infection in contacts of
tuberculosis patients in a semi-urban area]. Enferm Infecc Microbiol Clin. 2003
Jun-Jul;21(6):281-6. Spanish. PubMed PMID: 12809581.

854: Aliyu MH, Salihu HM, Ratard R. HIV infection and sputum-culture conversion
in patients diagnosed with Mycobacterium tuberculosis: a population-based study.
Wien Klin Wochenschr. 2003 May 30;115(10):340-6. PubMed PMID: 12800448.

855: Xu WG, Gao Z, Fan BD. [Follow-up observation on relapse of smear negative
pulmonary tuberculosis after short-course chemotherapy]. Zhonghua Jie He He Hu Xi
Za Zhi. 2003 Feb;26(2):74-6. Chinese. PubMed PMID: 12783655.

856: Gravendeel JM, Asapa AS, Becx-Bleumink M, Vrakking HA. Preliminary results
of an operational field study to compare side-effects, complaints and treatment
results of a single-drug short-course regimen with a four-drug fixed-dose
combination (4FDC) regimen in South Sulawesi, Republic of Indonesia. Tuberculosis
(Edinb). 2003;83(1-3):183-6. PubMed PMID: 12758210.

857: DomÃnguez-Castellano A, Muniain MA, Rodriguez-BaÃ±o J, Garcia M, Rios MJ,
Galvez J, Perez-Cano R. Factors associated with time to sputum smear conversion
in active pulmonary tuberculosis. Int J Tuberc Lung Dis. 2003 May;7(5):432-8.
PubMed PMID: 12757043.

858: Subhash HS, Ashwin I, Jesudason MV, Abharam OC, John G, Cherian AM, Thomas
K. Clinical characteristics and treatment response among patients with
multidrug-resistant tuberculosis: a retrospective study. Indian J Chest Dis
Allied Sci. 2003 Apr-Jun;45(2):97-103. PubMed PMID: 12715931.

859: Johnson JL, Ssekasanvu E, Okwera A, Mayanja H, Hirsch CS, Nakibali JG,
Jankus DD, Eisenach KD, Boom WH, Ellner JJ, Mugerwa RD; Uganda-Case Western
Reserve University Research Collaboration. Randomized trial of adjunctive
interleukin-2 in adults with pulmonary tuberculosis. Am J Respir Crit Care Med.
2003 Jul 15;168(2):185-91. Epub 2003 Apr 17. PubMed PMID: 12702550.

860: Puri MM, Arora VK. Role of gallium arsenide laser irradiation at 890 nm as
an adjunctive to anti-tuberculosis drugs in the treatment of pulmonary
tuberculosis. Indian J Chest Dis Allied Sci. 2003 Jan-Mar;45(1):19-23. PubMed
PMID: 12683708.

861: Kobashi Y, Matsushima T. Clinical analysis of pulmonary Mycobacterium avium
complex disease in association with corticosteroid treatment. J Infect Chemother.
2003 Mar;9(1):68-74. PubMed PMID: 12673411.

862: SchÃ¶n T, Elias D, Moges F, Melese E, Tessema T, Stendahl O, Britton S,
Sundqvist T. Arginine as an adjuvant to chemotherapy improves clinical outcome in
active tuberculosis. Eur Respir J. 2003 Mar;21(3):483-8. PubMed PMID: 12662006.

863: Smego RA, Ahmed N. A systematic review of the adjunctive use of systemic

corticosteroids for pulmonary tuberculosis. Int J Tuberc Lung Dis. 2003
Mar;7(3):208-13. Review. PubMed PMID: 12661833.

864: Beena S, Rao KN, Pai MR. Comparative evaluation of efficacy and safety
profile of three anti-tuberculous regimens in Mangalore. Indian J Med Sci. 2002
Jul;56(7):315-20. PubMed PMID: 12645166.

865: Lwilla F, Schellenberg D, Masanja H, Acosta C, Galindo C, Aponte J, Egwaga
S, Njako B, Ascaso C, Tanner M, Alonso P. Evaluation of efficacy of
community-based vs. institutional-based direct observed short-course treatment
for the control of tuberculosis in Kilombero district, Tanzania. Trop Med Int
Health. 2003 Mar;8(3):204-10. PubMed PMID: 12631309.

866: Tripathy SN, Tripathy SN. Tuberculosis and pregnancy. Int J Gynaecol Obstet.
2003 Mar;80(3):247-53. PubMed PMID: 12628525.

867: Niu HR, Lai ZH, Yuan L. [Observation on effect of supplementary treatment by
Astragalus injection in treating senile pulmonary tuberculosis patients].
Zhongguo Zhong Xi Yi Jie He Za Zhi. 2001 May;21(5):349-50. Chinese. PubMed PMID:
12577419.

868: Hutchison DC, Drobniewski FA, Milburn HJ. Management of multiple
drug-resistant tuberculosis. Respir Med. 2003 Jan;97(1):65-70. PubMed PMID:
12556013.

869: Wallis RS, Vinhas SA, Johnson JL, Ribeiro FC, Palaci M, Peres RL, SÃ¡ RT,
Dietze R, Chiunda A, Eisenach K, Ellner JJ. Whole blood bactericidal activity
during treatment of pulmonary tuberculosis. J Infect Dis. 2003 Jan
15;187(2):270-8. Epub 2003 Jan 6. PubMed PMID: 12552451.

870: Chen CX, Guo SM, Liu B, Yang JH, Xu N, Liu KW. [Glycocorticosteroid
administration prevents fulminant hepatic failure occurrence in patients with

chronic hepatitis B of severe degree]. Zhonghua Gan Zang Bing Za Zhi. 2003
Jan;11(1):37-9. Chinese. PubMed PMID: 12546741.

871: Schillinger JA, Kissinger P, Calvet H, Whittington WL, Ransom RL, Sternberg
MR, Berman SM, Kent CK, Martin DH, Oh MK, Handsfield HH, Bolan G, Markowitz LE,
Fortenberry JD. Patient-delivered partner treatment with azithromycin to prevent
repeated Chlamydia trachomatis infection among women: a randomized, controlled
trial. Sex Transm Dis. 2003 Jan;30(1):49-56. PubMed PMID: 12514443.

872: Noeske J, Nguenko PN. Impact of resistance to anti-tuberculosis drugs on
treatment outcome using World Health Organization standard regimens. Trans R Soc
Trop Med Hyg. 2002 Jul-Aug;96(4):429-33. PubMed PMID: 12497982.

873: Su WJ, Perng RP. Fixed-dose combination chemotherapy (Rifater/Rifinah) for
active pulmonary tuberculosis in Taiwan: a two-year follow-up. Int J Tuberc Lung
Dis. 2002 Nov;6(11):1029-32. PubMed PMID: 12475151.

874: Fraser HS, Jazayeri D, Mitnick CD, Mukherjee JS, Bayona J. Informatics tools
to monitor progress and outcomes of patients with drug resistant tuberculosis in
Peru. Proc AMIA Symp. 2002:270-4. PubMed PMID: 12463829; PubMed Central PMCID:
PMC2244209.

875: Wang L, Cai Y, Cheng Q, Hu Y, Xiao H. [Imbalance of Th1/Th2 cytokines in
patients with pulmonary tuberculosis]. Zhonghua Jie He He Hu Xi Za Zhi. 2002
Sep;25(9):535-7. Chinese. PubMed PMID: 12423561.

876: Patel N, Deshpande MM, Shah M. Effect of an immunomodulator containing
Mycobacterium w on sputum conversion in pulmonary tuberculosis. J Indian Med
Assoc. 2002 Mar;100(3):191-3. PubMed PMID: 12408283.

877: Yamagishi F, Sasaki Y, Kawabe Y, Toyoda E, Inuzuka K, Yamashita T, Mori T.

[The present situation of daily life of tuberculosis patients treated in
hospitals with beds for tuberculosis in Japan]. Kekkaku. 2002 Sep;77(9):609-14.
Japanese. PubMed PMID: 12397709.

878: Aksamit TR. Mycobacterium avium complex pulmonary disease in patients with
pre-existing lung disease. Clin Chest Med. 2002 Sep;23(3):643-53. Review. PubMed
PMID: 12371000.

879: HIV does not increase infectivity of M. tuberculosis. AIDS Wkly. 2000 Feb
14:2-3. PubMed PMID: 12295705.

880: Il'ina TIa, Zhangireev AA, Perzadaeva KA, Sidorenko OA. [Results of
introduction of extended WHO program for detection and treatment of patients with
tuberculosis of the respiratory organs]. Probl Tuberk. 2002;(6):15-8. Russian.
PubMed PMID: 12227042.

881: Kobashi Y, Okimoto N, Matsushima T, Shigetou E, Kuraoka T, Takeyama H, Eda
R, Yano S, Kobayashi K, Ohnishi T, Mori K, Ueda Y, Moritaka T, Nishimura K, Abe
T. [Effect of combined chemotherapy following the guidelines on treatment for
Mycobacterium avium complex pulmonary disease]. Kekkaku. 2002 Jun;77(6):435-41.
Japanese. PubMed PMID: 12136597.

882: Singla R, Al-Sharif N, Al-Sayegh MO, Osman MM, Shaikh MA. Influence of
anti-tuberculosis drug resistance on the treatment outcome of pulmonary
tuberculosis patients receiving DOTS in Riyadh, Saudi Arabia. Int J Tuberc Lung
Dis. 2002 Jul;6(7):585-91. PubMed PMID: 12102297.

883: Ribeiro-Rodrigues R, Resende Co T, Johnson JL, Ribeiro F, Palaci M, SÃ¡ RT,
Maciel EL, Pereira Lima FE, Dettoni V, Toossi Z, Boom WH, Dietze R, Ellner JJ,
Hirsch CS. Sputum cytokine levels in patients with pulmonary tuberculosis as
early markers of mycobacterial clearance. Clin Diagn Lab Immunol. 2002

Jul;9(4):818-23. PubMed PMID: 12093679; PubMed Central PMCID: PMC120011.

884: van Crevel R, Alisjahbana B, de Lange WC, Borst F, Danusantoso H, van der
Meer JW, Burger D, Nelwan RH. Low plasma concentrations of rifampicin in
tuberculosis patients in Indonesia. Int J Tuberc Lung Dis. 2002 Jun;6(6):497-502.
PubMed PMID: 12068982.

885: Sevim T, AtaÃ§ G, GÃ¼ngÃ¶r G, TÃ¶rÃ¼n I, Aksoy E, Gemci, Tahaoglu K. Treatment
outcome of relapse and defaulter pulmonary tuberculosis patients. Int J Tuberc
Lung Dis. 2002 Apr;6(4):320-5. PubMed PMID: 11936741.

886: Sundaram V, Fujiwara PI, Driver CR, Osahan SS, Munsiff SS. Yield of
continued monthly sputum evaluation among tuberculosis patients after culture
conversion. Int J Tuberc Lung Dis. 2002 Mar;6(3):238-45. PubMed PMID: 11934142.

887: Ruohonen RP, Goloubeva TM, Trnka L, Fomin MM, Zhemkova GA, Sinitsyn AV,
Lichachev AA, Koskela KG. Implementation of the DOTS strategy for tuberculosis in
the Leningrad Region, Russian Federation (1998-1999). Int J Tuberc Lung Dis. 2002
Mar;6(3):192-7. PubMed PMID: 11934136.

888: Filho LA, Kritski AL, Salles CL, Sardella IG, Silva MG, Fonseca LS, Saad MH.
Mycobacterium tuberculosis typing: usefulness of DRE-PCR to confirm
cross-contamination in the mycobacteriology laboratory of a general reference
hospital for AIDS. Int J Tuberc Lung Dis. 2002 Feb;6(2):150-4. PubMed PMID:
11931414.

889: Park SK, Lee CM, Heu JP, Song SD. A retrospective study for the outcome of
pulmonary resection in 49 patients with multidrug-resistant tuberculosis. Int J
Tuberc Lung Dis. 2002 Feb;6(2):143-9. PubMed PMID: 11931413.

890: Vranken R, Coulombier D, Kenyon T, Koosimile B, Mavunga T, Coggin W, Binkin

N. Use of a computerized tuberculosis register for automated generation of case
finding, sputum conversion, and treatment outcome reports. Int J Tuberc Lung Dis.
2002 Feb;6(2):111-20. PubMed PMID: 11931409.

891: Sevim T, Aksoy E, AtaÃ§ G, Ozmen I, Kapakli N, Horzum G, Ongel A, TÃ¶rÃ¼n T,
Tahaoglu K. Treatment adherence of 717 patients with tuberculosis in a social
security system hospital in Istanbul, Turkey. Int J Tuberc Lung Dis. 2002
Jan;6(1):25-31. PubMed PMID: 11931397.

892: Karyadi E, West CE, Schultink W, Nelwan RH, Gross R, Amin Z, Dolmans WM,
Schlebusch H, van der Meer JW. A double-blind, placebo-controlled study of
vitamin A and zinc supplementation in persons with tuberculosis in Indonesia:
effects on clinical response and nutritional status. Am J Clin Nutr. 2002
Apr;75(4):720-7. PubMed PMID: 11916759.

893: Pungrassami P, Johnsen SP, Chongsuvivatwong V, Olsen J. Has directly
observed treatment improved outcomes for patients with tuberculosis in southern
Thailand? Trop Med Int Health. 2002 Mar;7(3):271-9. PubMed PMID: 11903990.

894: Chierakul N, Chaiprasert A, Tingtoy N, Arjratanakul W, Pattanakitsakul SN.
Can serial qualitative polymerase chain reaction monitoring predict outcome of
pulmonary tuberculosis treatment? Respirology. 2001 Dec;6(4):305-9. PubMed PMID:
11844121.

895: Shiraishi Y, Nakajima Y, Takasuna K, Hanaoka T, Katsuragi N, Konno H.
Surgery for Mycobacterium avium complex lung disease in the clarithromycin era.
Eur J Cardiothorac Surg. 2002 Feb;21(2):314-8. PubMed PMID: 11825742.

896: Wang W, Jin G, Ye Y, Xia X, Wang A, Zhuang Y, Li G, Sun H, Wang Z, Lin M,
Chen H, Li J, Dan Z, Zhang X. [A clinical study on vaccine of Mycobacterium

vaccae in treating pulmonary tuberculosis]. Zhonghua Jie He He Hu Xi Za Zhi. 1999
Feb;22(2):108-10. Chinese. PubMed PMID: 11820954.

897: Shigetoh E, Murakami I, Yokosaki Y, Kurimoto N. [Treatment outcomes of
multidrug-resistant tuberculosis--comparison between success and failure cases].
Kekkaku. 2001 Dec;76(12):723-8. Japanese. PubMed PMID: 11806128.

898: Luo Y; National Cooperation Group On Clinical Study Of Mycobacterium Vaccae
Vaccine. [The immunotherapeutic effect of Mycobacterium vaccae vaccine on
initially treated pulmonary tuberculosis]. Zhonghua Jie He He Hu Xi Za Zhi. 2001
Jan;24(1):43-7. Chinese. PubMed PMID: 11802939.

899: Feng H, Du B, Dong Z. [Study on pathogenicity of sputum from cavity of
sputum negative patients with pulmonary tuberculosis after short course
chemotherapy]. Zhonghua Jie He He Hu Xi Za Zhi. 2000 Sep;23(9):559-62. Chinese.
PubMed PMID: 11778533.

900: Luo Y, Lu S, Guo S. [Immunotherapeutic effect of Mycobacterium vaccae on
multi-drug resistant pulmonary tuberculosis]. Zhonghua Jie He He Hu Xi Za Zhi.
2000 Feb;23(2):85-8. Chinese. PubMed PMID: 11778496.

901: Lu Y, Zhu L, Duan L. [Antituberculosis effect of levofloxacin]. Zhonghua Jie
He He Hu Xi Za Zhi. 2000 Jan;23(1):50-4. Chinese. PubMed PMID: 11778184.

902: Wei C, Yu H, Zhang R. [Cleaning cavity operation in treating relapse
bacillary cavitary pulmonary tuberculosis]. Zhonghua Jie He He Hu Xi Za Zhi. 1999
Sep;22(9):562-3. Chinese. PubMed PMID: 11776773.

903: Xia X, Gui X. [Clinical analysis of 96 cases with pulmonary disease caused
by nontuberculous mycobacteria]. Zhonghua Jie He He Hu Xi Za Zhi. 1999
Apr;22(4):239-41. Chinese. PubMed PMID: 11775923.

904: Yu D, Wang J, Hu X. [Clinical research of pasinizid on retreated sputum
positive pulmonary tuberculosis in senilities]. Zhonghua Jie He He Hu Xi Za Zhi.
2001 Oct;24(10):608-10. Chinese. PubMed PMID: 11770424.

905: Schoeman JF, Elshof JW, Laubscher JA, Janse van Rensburg A, Donald PR. The
effect of adjuvant steroid treatment on serial cerebrospinal fluid changes in
tuberculous meningitis. Ann Trop Paediatr. 2001 Dec;21(4):299-305. PubMed PMID:
11732147.

906: Nakano N, Hirayama T, Abe M, Nishimura K, Imachi T. [Surgical management of
non-tuberculous mycobacteriosis and tuberculosis of the lung]. Kekkaku. 2001
Oct;76(10):649-56. Japanese. PubMed PMID: 11712385.

907: Tamura M, Shirayama R, Kasahara R, Miyazaki R, Yoshikawa M, Tsukaguchi K,
Yoneda T, Narita N. [A study on relation between active pulmonary tuberculosis
and underlying diseases]. Kekkaku. 2001 Sep;76(9):619-24. Japanese. PubMed PMID:
11676119.

908: Franco J, Camarena JJ, Nogueira JM, Blanquer R, Ruiz MJ, MarÃ-n J.
Serological response (Western blot) to fractions of Mycobacterium tuberculosis
sonicate antigen in tuberculosis patients and contacts. Int J Tuberc Lung Dis.
2001 Oct;5(10):958-62. PubMed PMID: 11605891.

909: Becx-Bleumink M, Wibowo H, Apriani W, Vrakking H. High tuberculosis
notification and treatment success rates through community participation in
central Sulawesi, Republic of Indonesia. Int J Tuberc Lung Dis. 2001
Oct;5(10):920-5. PubMed PMID: 11605885.

910: Cattaneo C. Neltenexine tablets in smoking and non-smoking patients with
COPD. A double-blind, randomised, controlled study versus placebo. Minerva Med.
2001 Aug;92(4):277-84. PubMed PMID: 11535971.

911: Fadda G. Oral neltenexine in patients with obstructive airways diseases: an
open, randomised, controlled comparison versus sobrerol. Minerva Med. 2001
Aug;92(4):269-75. PubMed PMID: 11535970.

912: Mawer C, Ignatenko N, Wares D, Strelis A, Golubchikova V, Yanova G,
Lyagoshina T, Sharaburova O, Banatvala N. Comparison of the effectiveness of WHO
short-course chemotherapy and standard Russian antituberculous regimens in Tomsk,
western Siberia. Lancet. 2001 Aug 11;358(9280):445-9. PubMed PMID: 11513907.

913: Linnane SJ, Thin AG, Keatings VM, Moynihan JB, McLoughlin P, FitzGerald MX.
Glucocorticoid treatment reduces exhaled nitric oxide in cystic fibrosis
patients. Eur Respir J. 2001 Jun;17(6):1267-70. PubMed PMID: 11491175.

914: Jiang W, Chen H, Gao J, Huaibei M. [Efficacy of regimens containing INH, RFP
with varied chemotherapy courses on retreated culture positive
pneumoconio-tuberculosis]. Zhonghua Jie He He Hu Xi Za Zhi. 1998
Nov;21(11):648-50. Chinese. PubMed PMID: 11477888.

915: Zhu L, Yan B, Ma W. [Controlled clinical study on efficacy of fixed-dose
compounds rifater/rifinah in antituberculous chemotherapy]. Zhonghua Jie He He Hu
Xi Za Zhi. 1998 Nov;21(11):645-7. Chinese. PubMed PMID: 11477887.

916: Rutta E, Kipingili R, Lukonge H, Assefa S, Mitsilale E, Rwechungura S.
Treatment outcome among Rwandan and Burundian refugees with sputum smear-positive
tuberculosis in Ngara, Tanzania. Int J Tuberc Lung Dis. 2001 Jul;5(7):628-32.
PubMed PMID: 11467369.

917: Singla R, Gupta S, Gupta R, Arora VK. Efficacy and safety of sparfloxacin in
combination with kanamycin and ethionamide in multidrug-resistant pulmonary
tuberculosis patients: preliminary results. Int J Tuberc Lung Dis. 2001
Jun;5(6):559-63. PubMed PMID: 11409584.

918: Escalante P, Graviss EA, Griffith DE, Musser JM, Awe RJ. Treatment of
isoniazid-resistant tuberculosis in southeastern Texas. Chest. 2001 Jun;119(6):1730-6. PubMed PMID: 11399698.

919: Ige OM, Bakare NA, Onadeko BO. Modified short-course chemotherapy of
pulmonary tuberculosis in Ibadan, Nigeria--a preliminary report. Afr J Med Med
Sci. 2000 Mar;29(1):51-3. PubMed PMID: 11379469.

920: An S, Fan Y. [Polymerase chain reaction technique in monitoring treatment of
bacillary pulmonary tuberculosis]. Zhonghua Jie He He Hu Xi Za Zhi. 1998
Jul;21(7):392-4. Chinese. PubMed PMID: 11326874.

921: Chu N, Yan B, Zhu L. [Controlled clinical trial on efficacy of 5-month
regimens and whole course intermittent 6-month regimens in treating bacillary
pulmonary tuberculosis]. Zhonghua Jie He He Hu Xi Za Zhi. 1998 Jul;21(7):388-91.
Chinese. PubMed PMID: 11326873.

922: Chiang CY, Yu MC, Bai KJ, Suo J, Lin TP, Lee YC. Pulmonary resection in the
treatment of patients with pulmonary multidrug-resistant tuberculosis in Taiwan.
Int J Tuberc Lung Dis. 2001 Mar;5(3):272-7. PubMed PMID: 11326827.

923: Tu D, Zhang L, Su J. [Resistance and efficacy of treatment in relapse
pulmonary tuberculosis]. Zhonghua Jie He He Hu Xi Za Zhi. 2000 Nov;23(11):666-8.
Chinese. PubMed PMID: 11225030.

924: Wada M. [Effectiveness and problems of PZA-containing 6-month regimen for
the treatment of new pulmonary tuberculosis patients]. Kekkaku. 2001
Jan;76(1):33-43. Review. Japanese. PubMed PMID: 11211781.

925: Mayo RE, Stanford JL. Double-blind placebo-controlled trial of Mycobacterium
vaccae immunotherapy for tuberculosis in KwaZulu, South Africa, 1991-97. Trans R
Soc Trop Med Hyg. 2000 Sep-Oct;94(5):563-8. PubMed PMID: 11132390.

926: Desjardin LE, Hehman GL. Quantification of M. tuberculosis DNA in Sputum
During the Treatment of Pulmonary Tuberculosis. Methods Mol Med. 2001;48:121-31.
doi: 10.1385/1-59259-077-2:121. PubMed PMID: 21374412.

927: Sacks LV, Pendle S, Orlovic D, Andre M, Popara M, Moore G, Thonell L,
Hurwitz S. Adjunctive salvage therapy with inhaled aminoglycosides for patients
with persistent smear-positive pulmonary tuberculosis. Clin Infect Dis. 2001
Jan;32(1):44-9. Epub 2000 Dec 12. PubMed PMID: 11118385.

928: AvendaÃ±o M, Goldstein RS. Multidrug-resistant tuberculosis: long term
follow-up of 40 non-HIV-infected patients. Can Respir J. 2000 Sep-Oct;7(5):383-9.
PubMed PMID: 11058206.

929: Hiyama J, Marukawa M, Shiota Y, Ono T, Mashiba H. Factors influencing
response to treatment of pulmonary tuberculosis. Acta Med Okayama. 2000
Aug;54(4):139-45. PubMed PMID: 10985173.

930: Masjedi MR, Jamaati HR, Amin FK, Velayati AA. Detection of Mycobacterium
tuberculosis in bronchial washing of smear-positive patients after sputum
conversion. Monaldi Arch Chest Dis. 2000 Jun;55(3):212-5. PubMed PMID: 10948669.

931: Hakim JG, Ternouth I, Mushangi E, Siziya S, Robertson V, Malin A. Double
blind randomised placebo controlled trial of adjunctive prednisolone in the
treatment of effusive tuberculous pericarditis in HIV seropositive patients.
Heart. 2000 Aug;84(2):183-8. PubMed PMID: 10908256; PubMed Central PMCID:
PMC1760932.

932: Zhangireev AA, Ismailov ShSh, Berikova EM. [New cases of pulmonary
tuberculosis treatment within DOTS strategy]. Probl Tuberk. 2000;(3):23-5.
Russian. PubMed PMID: 10900979.

933: Mishkinis K, KaminskaÄ-te A, Purvanetskene B. [Treatment of multidrug
resistant tuberculosis in Santakiskes tuberculosis hospital]. Probl Tuberk.
2000;(3):9-11. Russian. PubMed PMID: 10900974.

934: Sousa AO, Wargnier A, Poinsignon Y, Simonney N, Gerber F, Lavergne F,
Herrmann JL, Lagrange PH. Kinetics of circulating antibodies, immune complex and
specific antibody-secreting cells in tuberculosis patients during 6 months of
antimicrobial therapy. Tuber Lung Dis. 2000;80(1):27-33. PubMed PMID: 10897381.

935: Lawn SD, Obeng J, Acheampong JW, Griffin GE. Resolution of the acute-phase
response in West African patients receiving treatment for pulmonary tuberculosis.
Int J Tuberc Lung Dis. 2000 Apr;4(4):340-4. PubMed PMID: 10777083.

936: Banerjee A, Harries AD, Mphasa N, Nyirenda TE, Veen J, Ringdal T, Van Gorkom
J, Salaniponi FM. Evaluation of a unified treatment regimen for all new cases of
tuberculosis using guardian-based supervision. Int J Tuberc Lung Dis. 2000
Apr;4(4):333-9. PubMed PMID: 10777082.

937: Johnson JL, Kamya RM, Okwera A, Loughlin AM, Nyole S, Hom DL, Wallis RS,
Hirsch CS, Wolski K, Foulds J, Mugerwa RD, Ellner JJ. Randomized controlled trial
of Mycobacterium vaccae immunotherapy in non-human immunodeficiency
virus-infected ugandan adults with newly diagnosed pulmonary tuberculosis. The
Uganda-Case Western Reserve University Research Collaboration. J Infect Dis. 2000
Apr;181(4):1304-12. Epub 2000 Apr 7. PubMed PMID: 10753731.

938: Bwire R, Borgdorff MW, Sticht-Groh V, Rieder HL, Kawuma HJ, Bretzel G,
RÃ¼sch-Gerdes S. Tuberculosis chemotherapy and sputum conversion among
HIV-seropositive and HIV-seronegative patients in south-eastern Uganda. East Afr
Med J. 1999 Jun;76(6):307-13. PubMed PMID: 10750516.

939: Espinal MA, PerÃ©z EN, BaÃ©z J, HÃ©nriquez L, FernÃ¡ndez K, Lopez M, Olivo P,
Reingold AL. Infectiousness of Mycobacterium tuberculosis in HIV-1-infected
patients with tuberculosis: a prospective study. Lancet. 2000 Jan 22;355(9200):275-80. PubMed PMID: 10675075.

940: Griffith DE, Brown BA, Cegielski P, Murphy DT, Wallace RJ Jr. Early results
(at 6 months) with intermittent clarithromycin-including regimens for lung
disease due to Mycobacterium avium complex. Clin Infect Dis. 2000 Feb;30(2):288-92. PubMed PMID: 10671330.

941: Lobato MN, Mohle-Boetani JC, Royce SE. Missed opportunities for preventing
tuberculosis among children younger than five years of age. Pediatrics. 2000
Dec;106(6):E75. PubMed PMID: 11099618.

942: Mac JT, Doordan A, Carr CA. Evaluation of the effectiveness of a directly
observed therapy program with Vietnamese tuberculosis patients. Public Health
Nurs. 1999 Dec;16(6):426-31. PubMed PMID: 10620253.

943: Kaga K, Park J, Nishiumi N, Iwasaki M, Inoue H. Usefulness of video-assisted
thoracic surgery (Two Windows Method) in the treatment of lung cancer for elderly
patients. J Cardiovasc Surg (Torino). 1999 Oct;40(5):721-3. PubMed PMID:
10597011.

944: Nutini S, Fiorenti F, Codecasa LR, Casali L, Besozzi G, Di Pisa G, Nardini
S, Migliori GB. Hospital admission policy for tuberculosis in pulmonary centres
in Italy: a national survey. AIPO Tuberculosis Study Group. Italian Association
of Hospital Pulmonologists. Int J Tuberc Lung Dis. 1999 Nov;3(11):985-91. PubMed
PMID: 10587320.

945: Aleksandra S, Jacek Z, PaweÅ, R, Ewa AK, Magdalena K. [Results of treating
patients with mycobacterial respiratory infections negative for HIV virus
(retrospective study)]. Pneumonol Alergol Pol. 1999;67(5-6):200-7. Polish. PubMed

PMID: 10570641.

946: Lawn SD, Shattock RJ, Acheampong JW, Lal RB, Folks TM, Griffin GE, Butera
ST. Sustained plasma TNF-alpha and HIV-1 load despite resolution of other
parameters of immune activation during treatment of tuberculosis in Africans.
AIDS. 1999 Nov 12;13(16):2231-7. PubMed PMID: 10563708.

947: DizdareviÄ‡ Z. [Basic characteristics of the National Tuberculosis Program in
Bosnia-Herzegovina]. Med Arh. 1999;53(3 Suppl 1):5-8. Croatian. PubMed PMID:
10546458.

948: Tsogt G, Levy M, Sudre P, Norval PY, Spinaci S. DOTS (Directly Observed
Treatment Strategy) project in Mongolia, 1995. Int J Tuberc Lung Dis. 1999
Oct;3(10):886-90. PubMed PMID: 10524585.

949: Wasiak W, Szmidt M. A six week double blind, placebo controlled, crossover
study of the effect of misoprostol in the treatment of aspirin sensitive asthma.
Thorax. 1999 Oct;54(10):900-4. PubMed PMID: 10491452; PubMed Central PMCID:
PMC1745363.

950: Lao LY, De Guia T. Tuberculin skin testing: determinants and reaction.
Respirology. 1999 Sep;4(3):311-7. PubMed PMID: 10489682.

951: Sung SW, Kang CH, Kim YT, Han SK, Shim YS, Kim JH. Surgery increased the
chance of cure in multi-drug resistant pulmonary tuberculosis. Eur J Cardiothorac
Surg. 1999 Aug;16(2):187-93. PubMed PMID: 10485419.

952: Tanaka E, Kimoto T, Tsuyuguchi K, Watanabe I, Matsumoto H, Niimi A, Suzuki
K, Murayama T, Amitani R, Kuze F. Effect of clarithromycin regimen for
Mycobacterium avium complex pulmonary disease. Am J Respir Crit Care Med. 1999
Sep;160(3):866-72. PubMed PMID: 10471610.

953: Mangunnegoro H, Hudoyo A. Efficacy of low-dose ofloxacin in the treatment of
multidrug-resistant tuberculosis in Indonesia. Chemotherapy. 1999;45 Suppl
2:19-25. PubMed PMID: 10449894.

954: Maranetra KN. Quinolones and multidrug-resistant tuberculosis. Chemotherapy.
1999;45 Suppl 2:12-8. PubMed PMID: 10449893.

955: Dewan RK, Singh S, Kumar A, Meena BK. Thoracoplasty: an obsolete procedure?
Indian J Chest Dis Allied Sci. 1999 Apr-Jun;41(2):83-8. PubMed PMID: 10437320.

956: Carneiro JR, Sato EI. Double blind, randomized, placebo controlled clinical
trial of methotrexate in systemic lupus erythematosus. J Rheumatol. 1999
Jun;26(6):1275-9. PubMed PMID: 10381042.

957: Qin M, Han M, Li S. [Surgical treatment in pulmonary tuberculosis with
sputum long-term Mycobacterium tuberculosis positive]. Zhonghua Jie He He Hu Xi
Za Zhi. 1997 Oct;20(5):311-2. Chinese. PubMed PMID: 10374461.

958: Xiao C, Fu Y, Li Z. [Efficacy of unfixed continuation phase short-course
chemotherapy]. Zhonghua Jie He He Hu Xi Za Zhi. 1997 Dec;20(6):358-60. Chinese.
PubMed PMID: 10374447.

959: Zhang Y, Qian H, Chen M. [Bronchofiberscope and catheter intervention in
treatment of multi-drug resistant pulmonary tuberculosis]. Zhonghua Jie He He Hu
Xi Za Zhi. 1997 Dec;20(6):354-7. Chinese. PubMed PMID: 10374446.

960: Liu Z, Shilkret KL, Ellis HM. Predictors of sputum culture conversion among
patients with tuberculosis in the era of tuberculosis resurgence. Arch Intern
Med. 1999 May 24;159(10):1110-6. PubMed PMID: 10335689.

961: Zalesky R, Abdullajev F, Khechinashvili G, Safarian M, Madaras T, Grzemska

M, Englund E, Dittmann S, Raviglione M. Tuberculosis control in the Caucasus:
successes and constraints in DOTS implementation. Int J Tuberc Lung Dis. 1999
May;3(5):394-401. PubMed PMID: 10331728.

962: Serrate G, Vaqueiro M, NÃºÃ±ez M, Tobar M, Cascales E, Segura F. [Tuberculosis
in a social health care center: epidemiologic study and prevention activities].
Enferm Infecc Microbiol Clin. 1999 Mar;17(3):130-4. Spanish. PubMed PMID:
10217848.

963: BilaÃ§eroÄŸlu S, Perim K, BÃ¼yÃ¼kÅŸirin M, Celikten E. Prednisolone: a beneficial
and safe adjunct to antituberculosis treatment? A randomized controlled trial.
Int J Tuberc Lung Dis. 1999 Jan;3(1):47-54. PubMed PMID: 10094169.

964: Marciniuk DD, McNab BD, Martin WT, Hoeppner VH. Detection of pulmonary
tuberculosis in patients with a normal chest radiograph. Chest. 1999
Feb;115(2):445-52. PubMed PMID: 10027446.

965: Hellyer TJ, DesJardin LE, Teixeira L, Perkins MD, Cave MD, Eisenach KD.
Detection of viable Mycobacterium tuberculosis by reverse transcriptase-strand
displacement amplification of mRNA. J Clin Microbiol. 1999 Mar;37(3):518-23.
PubMed PMID: 9986805; PubMed Central PMCID: PMC84447.

966: LoBue PA, Cass R, Lobo D, Moser K, Catanzaro A. Development of housing
programs to aid in the treatment of tuberculosis in homeless individuals: a pilot
study. Chest. 1999 Jan;115(1):218-23. PubMed PMID: 9925087.

967: Kumaresan JA, Ahsan Ali AK, Parkkali LM. Tuberculosis control in Bangladesh:
success of the DOTS strategy. Int J Tuberc Lung Dis. 1998 Dec;2(12):992-8. PubMed
PMID: 9869115.

968: Jarvis B, Lamb HM. Rifapentine. Drugs. 1998 Oct;56(4):607-16; discussion
617. Review. Erratum in: Drugs 1999 Apr;57(4):506. PubMed PMID: 9806107.

969: Lienhardt C, Manneh K, Bouchier V, Lahai G, Milligan PJ, McAdam KP. Factors
determining the outcome of treatment of adult smear-positive tuberculosis cases
in The Gambia. Int J Tuberc Lung Dis. 1998 Sep;2(9):712-8. PubMed PMID: 9755924.

970: Weir SC, Fischer SH, Stock F, Gill VJ. Detection of Legionella by PCR in
respiratory specimens using a commercially available kit. Am J Clin Pathol. 1998
Sep;110(3):295-300. PubMed PMID: 9728603.

971: Outcome of first-line tuberculosis treatment in migrants from Vietnam.
International Organization for Migration Tuberculosis Working Group. Int J Tuberc
Lung Dis. 1998 Aug;2(8):641-6. PubMed PMID: 9712278.

972: Karak B, Garg RK. Corticosteroids in tuberculous meningitis. Indian Pediatr.
1998 Feb;35(2):193-4. PubMed PMID: 9707871.

973: Morcos MM, Gabr AA, Samuel S, Kamel M, el Baz M, el Beshry M, Michail RR.
Vitamin D administration to tuberculous children and its value. Boll Chim Farm.
1998 May;137(5):157-64. PubMed PMID: 9689902.

974: McKenna MT, McCray E, Jones JL, Onorato IM, Castro KG. The fall after the
rise: Tuberculosis in the United States, 1991 through 1994. Am J Public Health.
1998 Jul;88(7):1059-63. PubMed PMID: 9663154; PubMed Central PMCID: PMC1508272.

975: Griffith DE, Brown BA, Murphy DT, Girard WM, Couch L, Wallace RJ Jr. Initial
(6-month) results of three-times-weekly azithromycin in treatment regimens for
Mycobacterium avium complex lung disease in human immunodeficiency virus-negative
patients. J Infect Dis. 1998 Jul;178(1):121-6. PubMed PMID: 9652431.

976: Iinuma Y, Ichiyama S, Yamori S, Oohama J, Takagi N, Hasegawa Y, Shimokata K,

Nakashima N. Diagnostic value of the Amplicor PCR assay for initial diagnosis and
assessment of treatment response for pulmonary tuberculosis. Microbiol Immunol.
1998;42(4):281-7. PubMed PMID: 9623915.

977: Cao JP, Zhang LY, Zhu JQ, Chin DP. Two-year follow-up of directly-observed
intermittent regimens for smear-positive pulmonary tuberculosis in China. Int J
Tuberc Lung Dis. 1998 May;2(5):360-4. PubMed PMID: 9613630.

978: Jouveshomme S, Dautzenberg B, Bakdach H, Derenne JP. Preliminary results of
collapse therapy with plombage for pulmonary disease caused by
multidrug-resistant mycobacteria. Am J Respir Crit Care Med. 1998 May;157(5 Pt
1):1609-15. PubMed PMID: 9603145.

979: el-Sadr WM, Perlman DC, Matts JP, Nelson ET, Cohn DL, Salomon N, Olibrice M,
Medard F, Chirgwin KD, Mildvan D, Jones BE, Telzak EE, Klein O, Heifets L, Hafner
R. Evaluation of an intensive intermittent-induction regimen and duration of
short-course treatment for human immunodeficiency virus-related pulmonary
tuberculosis. Terry Beirn Community Programs for Clinical Research on AIDS
(CPCRA) and the AIDS Clinical Trials Group (ACTG). Clin Infect Dis. 1998
May;26(5):1148-58. PubMed PMID: 9597244.

980: Asch S, Leake B, Anderson R, Gelberg L. Why do symptomatic patients delay
obtaining care for tuberculosis? Am J Respir Crit Care Med. 1998 Apr;157(4 Pt
1):1244-8. PubMed PMID: 9563746.

981: Park IW, Choi BW, Hue SH. Prospective study of corticosteroid as an adjunct
in the treatment of endobronchial tuberculosis in adults. Respirology. 1997
Dec;2(4):275-81. PubMed PMID: 9525297.

982: Telzak EE, Fazal BA, Turett GS, Justman JE, Blum S. Factors influencing time
to sputum conversion among patients with smear-positive pulmonary tuberculosis.
Clin Infect Dis. 1998 Mar;26(3):775-6. PubMed PMID: 9524873.

983: Wiesemann HG, Steinkamp G, Ratjen F, Bauernfeind A, Przyklenk B, DÃ¶ring G,
von der Hardt H. Placebo-controlled, double-blind, randomized study of
aerosolized tobramycin for early treatment of Pseudomonas aeruginosa colonization
in cystic fibrosis. Pediatr Pulmonol. 1998 Feb;25(2):88-92. PubMed PMID: 9516091.

984: Rajeswari R, Chandrasekaran K, Thiruvalluvan E, Rajaram K, Sudha G,
Sivasubramanian S, Santha T, Prabhakar R. Study of the feasibility of involving
male student volunteers in case holding in an urban tuberculosis programme. Int J
Tuberc Lung Dis. 1997 Dec;1(6):573-5. PubMed PMID: 9487457.

985: Zhao FZ, Levy MH, Wen S. Sputum microscopy results at two and three months
predict outcome of tuberculosis treatment. Int J Tuberc Lung Dis. 1997
Dec;1(6):570-2. PubMed PMID: 9487456.

986: Schwander SK, Dietrich M, Mugyenyi P, Kityo C, Okwera A, Johnson J, Nsubuga
P, Ruesch-Gerdes S, Whalen C. Clinical course of human immunodeficiency virus
type 1 associated pulmonary tuberculosis during short-course antituberculosis
therapy. East Afr Med J. 1997 Sep;74(9):543-8. PubMed PMID: 9487427.

987: Bando T, Fujimura M, Shinagawa S, Mizuhashi K, Noda Y, Ohta G, Hirone T,
Matsuda T. Effect of beclomethasone dipropionate inhalation on eosinophilic
bronchitis in patients with silicosis. Arzneimittelforschung. 1997
Dec;47(12):1370-4. PubMed PMID: 9450166.

988: Telzak EE, Fazal BA, Pollard CL, Turett GS, Justman JE, Blum S. Factors
influencing time to sputum conversion among patients with smear-positive
pulmonary tuberculosis. Clin Infect Dis. 1997 Sep;25(3):666-70. PubMed PMID:
9314458.

989: Ye Z, Pei X, Yang Y. [Analysis of causes of drug resistance and therapeutic

effects on 27 multi-drug resistant pulmonary tuberculosis patients]. Zhonghua Jie
He He Hu Xi Za Zhi. 1997 Aug;20(4):234-6. Chinese. PubMed PMID: 10072811.

990: Hong Z, Li S. [Diagnosis and surgical treatment for tuberculous bronchostenosis]. Zhonghua Jie He He Hu Xi Za Zhi. 1997 Aug;20(4):231-3. Chinese.
PubMed PMID: 10072810.

991: Manresa F, Galarza I, CaÃ±ete C. Using corticosteroids to treat tuberculous
pleurisy. Chest. 1997 Jul;112(1):291-2. PubMed PMID: 9228398.

992: Agutu WO. Short-course tuberculosis chemotherapy in rural Somalia. East Afr
Med J. 1997 Jun;74(6):348-52. PubMed PMID: 9487394.

993: Nakaya M, Yoneda T, Kobayashi A, Onohara Y, Ikoma Y, Fukuoka A, Tomoda K,
Takenaka H, Okamura H, Yamamoto C, Fukuoka K, Tokuyama T, Okamoto Y, Yoshikawa M,
Tsukaguchi K, Narita N. [Cytokine producing ability of peripheral blood
mononuclear cells in the clinical course of pulmonary tuberculosis]. Kekkaku.
1997 Jun;72(6):403-10. Japanese. PubMed PMID: 9248274.

994: Tanaka E, Amitani R, Niimi A, Suzuki K, Murayama T, Kuze F. Yield of
computed tomography and bronchoscopy for the diagnosis of Mycobacterium avium
complex pulmonary disease. Am J Respir Crit Care Med. 1997 Jun;155(6):2041-6.
PubMed PMID: 9196113.

995: Menzies D. Issues in the management of contacts of patients with active
pulmonary tuberculosis. Can J Public Health. 1997 May-Jun;88(3):197-201. Review.
PubMed PMID: 9260361.

996: BozÃ³ky G, Ruby E, GÃ³hÃ©r I, TÃ³th J, Mohos A. [Hematologic abnormalities in
pulmonary tuberculosiss]. Orv Hetil. 1997 Apr 27;138(17):1053-6. Hungarian.
PubMed PMID: 9182273.

997: Hirano K, Saitoh T, Kadono K, Oose H, Watanabe S, Hasegawa S.
[Conversion of
chronic necrotizing pulmonary aspergillosis to invasive pulmonary
aspergillosis,
and successful treatment with fluconazole]. Nihon Kyobu Shikkan
Gakkai Zasshi.
1997 Apr;35(4):408-13. Review. Japanese. PubMed PMID: 9212665.

998: Chaulk CP, Pope DS. The Baltimore City Health Department
program of directly
observed therapy for tuberculosis. Clin Chest Med. 1997
Mar;18(1):149-54. Review.
PubMed PMID: 9098619.

999: TÃ¼mmler B, Bosshammer J, Breitenstein S, Brockhausen I,
Gudowius P, Herrmann
C, Herrmann S, Heuer T, Kubesch P, Mekus F, RÃ¶mling U, Schmidt KD,
Spangenberg C,
Walter S. Infections with Pseudomonas aeruginosa in patients with
cystic
fibrosis. Behring Inst Mitt. 1997 Feb;(98):249-55. Review. PubMed
PMID: 9382747.

1000: Fujita A, Suzuki A, Hamaoka T, Tojima H. [Clinical course of
HIV-infected
tuberculosis patients who admitted to the tuberculosis isolation
ward: current
problems of medical care]. Kekkaku. 1997 Feb;72(2):67-72. Japanese.
PubMed PMID:
9071088.

1001: Schoeman JF, Van Zyl LE, Laubscher JA, Donald PR. Effect of
corticosteroids
on intracranial pressure, computed tomographic findings, and
clinical outcome in
young children with tuberculous meningitis. Pediatrics. 1997
Feb;99(2):226-31.
PubMed PMID: 9024451.

1002: Korzeniewska-KoseÅ‚a M, MichaÅ‚owska-Mitczuk D, Wedzicha S,
Pawlicka L,
Miller M, KuÅ› J. [Reasons for chronic expectoration in patients
with pulmonary
tuberculosis registered in the Central Tuberculosis Registry].
Pneumonol Alergol
Pol. 1997;65(3-4):181-6. Polish. PubMed PMID: 9489413.

1003: MichaÅ‚owska-Mitczuk D, KuÅ› J. [Treatment of patients for
chronic pulmonary

tuberculosis with expectoration--selection and tolerance to drugs]. Pneumonol
Alergol Pol. 1997;65(3-4):172-80. Polish. PubMed PMID: 9489412.

1004: MichaÅ‚owska-Mitczuk D, KuÅ› J. [Treatment of patients with chronic pulmonary
tuberculosis with expectoration--evaluation of effectiveness]. Pneumonol Alergol
Pol. 1997;65(3-4):164-71. Polish. PubMed PMID: 9489411.

1005: Yuen KY, Chan KS, Chan CM, Ho PL, Ng MH. Monitoring the therapy of
pulmonary tuberculosis by nested polymerase chain reaction assay. J Infect. 1997
Jan;34(1):29-33. PubMed PMID: 9120321.

1006: Yano M, Arai T, Inagaki K, Nomura T. [Surgically unsuccessful cases with
pulmonary tuberculosis]. Kekkaku. 1997 Jan;72(1):35-8. Japanese. PubMed PMID:
9038013.

1007: Wen S, Su Y, Zhang F. [Analysis of short-term effects of short-course
intermittent chemotherapies of the World Bank Loaned Project]. Zhonghua Jie He He
Hu Xi Za Zhi. 1996 Dec;19(6):357-9. Chinese. PubMed PMID: 9596819.

1008: Lambregts-van Weezenbeek CS, Keizer ST, Sebek MM, Schepp-Beelen JC, van der
Loo CJ. [Transmission of multiresistant tuberculosis in a Dutch hospital]. Ned
Tijdschr Geneeskd. 1996 Nov 16;140(46):2293-5. Dutch. PubMed PMID: 8984384.

1009: Frieden TR, Sherman LF, Maw KL, Fujiwara PI, Crawford JT, Nivin B, Sharp V,
Hewlett D Jr, Brudney K, Alland D, Kreisworth BN. A multi-institutional outbreak
of highly drug-resistant tuberculosis: epidemiology and clinical outcomes. JAMA.
1996 Oct 16;276(15):1229-35. PubMed PMID: 8849750.

1010: Hursti TJ, Avall-Lundqvist E, BÃ¶rjeson S, Fredrikson M, FÃ¼rst CJ, Steineck
G, Peterson C. Impact of tumour burden on chemotherapy-induced nausea and
vomiting. Br J Cancer. 1996 Oct;74(7):1114-9. PubMed PMID: 8855984; PubMed

Central PMCID: PMC2077107.

1011: Virgo KS, Naunheim KS, McKirgan LW, Kissling ME, Lin JC, Johnson FE. Cost
of patient follow-up after potentially curative lung cancer treatment. J Thorac
Cardiovasc Surg. 1996 Aug;112(2):356-63. PubMed PMID: 8751503.

1012: el-Sadr W, Medard F, Berthaud V. Directly observed therapy for
tuberculosis: the Harlem Hospital experience, 1993. Am J Public Health. 1996
Aug;86(8):1146-9. Erratum in: Am J Public Health 1997 Feb;87(2):168. Barthaud V
[corrected to Berthaud V]. PubMed PMID: 8712276; PubMed Central PMCID:
PMC1380628.

1013: Wyser C, Walzl G, Smedema JP, Swart F, van Schalkwyk EM, van de Wal BW.
Corticosteroids in the treatment of tuberculous pleurisy. A double-blind,
placebo-controlled, randomized study. Chest. 1996 Aug;110(2):333-8. PubMed PMID:
8697829.

1014: Quijada-Carrera J, Valenzuela-CastaÃ±o A, Povedano-GÃ³mez J,
FernÃ¡ndez-Rodriguez A, HernÃ¡nz-Mediano W, Gutierrez-Rubio A, de la
Iglesia-Salgado JL, GarcÃa-LÃ³pez A. Comparison of tenoxicam and bromazepan in the
treatment of fibromyalgia: a randomized, double-blind, placebo-controlled trial.
Pain. 1996 May-Jun;65(2-3):221-5. PubMed PMID: 8826510.

1015: Kennedy N, Berger L, Curram J, Fox R, Gutmann J, Kisyombe GM, Ngowi FI,
Ramsay AR, Saruni AO, Sam N, Tillotson G, Uiso LO, Yates M, Gillespie SH.
Randomized controlled trial of a drug regimen that includes ciprofloxacin for the
treatment of pulmonary tuberculosis. Clin Infect Dis. 1996 May;22(5):827-33.
PubMed PMID: 8722940.

1016: Yan S, Ye H, Lin S. [Effects of intermittent short-course chemotherapy
under full-course supervision on the treatment of smear positive pulmonary
tuberculosis]. Zhonghua Jie He He Hu Xi Za Zhi. 1996 Apr;19(2):104-6. Chinese.
PubMed PMID: 9388851.

1017: Sharma SK, Guleria R, Jain D, Chawla TC, Saha P, Mohan A, Jain NK. Effect
of additional oral ofloxacin administration in the treatment of
multidrug-resistant tuberculosis. Indian J Chest Dis Allied Sci. 1996
Apr-Jun;38(2):73-9. PubMed PMID: 8822640.

1018: Grassi C, Peona V. Use of rifabutin in the treatment of pulmonary
tuberculosis. Clin Infect Dis. 1996 Apr;22 Suppl 1:S50-4. Review. PubMed PMID:
8785257.

1019: Rieder HL. Sputum smear conversion during directly observed treatment for
tuberculosis. Tuber Lung Dis. 1996 Apr;77(2):124-9. PubMed PMID: 8762846.

1020: Lee CN, Lin TP, Chang MF, Jimenez MV, Dolfi L, Olliaro P. Rifabutin as
salvage therapy for cases of chronic multidrug-resistant pulmonary tuberculosis
in Taiwan. J Chemother. 1996 Apr;8(2):137-43. PubMed PMID: 8708745.

1021: McColloster P, Neff NE. Outpatient management of tuberculosis. Am Fam
Physician. 1996 Apr;53(5):1579-94. Review. PubMed PMID: 8623687.

1022: Chotmongkol V, Jitpimolmard S, Thavornpitak Y. Corticosteroid in
tuberculous meningitis. J Med Assoc Thai. 1996 Feb;79(2):83-90. PubMed PMID:
8868018.

1023: Sousa AR, Lane SJ, Atkinson BA, Poston RN, Lee TH. The effects of
prednisolone on the cutaneous tuberculin response in patients with
corticosteroid-resistant bronchial asthma. J Allergy Clin Immunol. 1996
Feb;97(2):698-706. PubMed PMID: 8621857.

1024: Li H, Ling Y. Treating tuberculous pleurisy with effusion by artificial
pneumothorax. Ann Ist Super Sanita. 1996;32(3):399-403. PubMed PMID: 9028062.

1025: Suo J, Yu MC, Lee CN, Chiang CY, Lin TP. Treatment of multidrug-resistant
tuberculosis in Taiwan. Chemotherapy. 1996;42 Suppl 3:20-3; discussion 30-3.
PubMed PMID: 8980864.

1026: Zhang LX. Treatment of multidrug-resistant tuberculosis in China.
Chemotherapy. 1996;42 Suppl 3:16-9; discussion 30-3. Review. PubMed PMID:
8980863.

1027: Maranetra KN. Treatment of multidrug-resistant tuberculosis in Thailand.
Chemotherapy. 1996;42 Suppl 3:10-5; discussion 30-3. Review. PubMed PMID:
8980862.

1028: Hara H. [From the aspects of complicated diseases]. Kekkaku. 1996
Jan;71(1):47-56. Japanese. PubMed PMID: 8808269.

1029: Liss GM, Khan R, Koven E, Simor AE. Tuberculosis infection among staff at a
Canadian community hospital. Infect Control Hosp Epidemiol. 1996 Jan;17(1):29-35.
PubMed PMID: 8789684.

1030: Zhang L, Kan G. [Fifteen years' experience in reformed Beijing tuberculosis
control programme]. Zhonghua Jie He He Hu Xi Za Zhi. 1995 Dec;18(6):337-9, 382.
Chinese. PubMed PMID: 8762490.

1031: Urbanczik R. [Therapeutic approaches in diseases caused by mycobacteria].
Pneumologie. 1995 Dec;49 Suppl 3:649-52. Review. German. PubMed PMID: 8577670.

1032: Fischer B, Ferlinz R. [Therapy and prognosis of tuberculosis].
Versicherungsmedizin. 1995 Dec 1;47(6):212-6. Review. German. PubMed PMID:
8571498.

1033: Galarza I, CaÃ±ete C, Granados A, EstopÃ R, Manresa F. Randomised trial of
corticosteroids in the treatment of tuberculous pleurisy. Thorax. 1995

Dec;50(12):1305-7. PubMed PMID: 8553306; PubMed Central PMCID:
PMC1021356.

1034: Jayaswal R, Arora PN, Panda BN. HIV IN TUBERCULOSIS. Med J
Armed Forces
India. 1995 Oct;51(4):259-263. doi: 10.1016/S0377-1237(17)30987-5.
Epub 2017 Jun
26. PubMed PMID: 28769309; PubMed Central PMCID: PMC5530203.

1035: Chaulk CP, Moore-Rice K, Rizzo R, Chaisson RE. Eleven years of
community-based directly observed therapy for tuberculosis. JAMA.
1995 Sep
27;274(12):945-51. PubMed PMID: 7674524.

1036: Johnson BJ, Ress SR, Willcox P, Pati BP, Lorgat F, Stead P,
Saha R, Lukey
P, Laochumroonvorapong P, Corral L, et al. Clinical and immune
responses of
tuberculosis patients treated with low-dose IL-2 and multidrug
therapy. Cytokines
Mol Ther. 1995 Sep;1(3):185-96. PubMed PMID: 9384675.

1037: Jawad F, Shera AS, Memon R, Ansari G. Glucose intolerance in
pulmonary
tuberculosis. J Pak Med Assoc. 1995 Sep;45(9):237-8. PubMed PMID:
8683828.

1038: Grassi C, Peona V. New drugs for tuberculosis. Eur Respir J
Suppl. 1995
Sep;20:714s-718s. Review. PubMed PMID: 8590571.

1039: Dlugovitzky D, Torres A, Hourquescos MC, Svetaz MJ, Quagliato
N, Valentini
E, Amigot B, Molteni O, Bottasso O. Low occurrence of arthritic
manifestations in
patients with pulmonary tuberculosis. T cell subsets and humoral
studies. Mem
Inst Oswaldo Cruz. 1995 Sep-Oct;90(5):623-8. PubMed PMID: 8569477.

1040: Tian XZ, Wang LX. [A study on the implementation of primary
chemotherapy to
pulmonary tuberculosis with positive bacillus]. Zhonghua Liu Xing
Bing Xue Za
Zhi. 1995 Aug;16(4):220-2. Chinese. PubMed PMID: 7585902.

1041: Bagnato GF, Di Cesare E, Gulli S, Cucinotta D. Long-term
treatment of

pulmonary tuberculosis with ofloxacin in a subject with liver cirrhosis. Monaldi
Arch Chest Dis. 1995 Aug;50(4):279-81. PubMed PMID: 7550207.

1042: Musch E, TÃ¼nnerhoff-MÃ¼cke A. [Tuberculous anal fistula in acquired
immunologic deficiency syndrome]. Z Gastroenterol. 1995 Aug;33(8):440-4. German.
PubMed PMID: 7483736.

1043: McAdams HP, Erasmus J, Winter JA. Radiologic manifestations of pulmonary
tuberculosis. Radiol Clin North Am. 1995 Jul;33(4):655-78. Review. PubMed PMID:
7610237.

1044: Kameda K. [Status quo of pyrazinamide as an antituberculosis drug].
Kekkaku. 1995 Jul;70(7):445-55. Review. Japanese. PubMed PMID: 7564054.

1045: Templeton GL, Illing LA, Young L, Cave D, Stead WW, Bates JH. The risk for
transmission of Mycobacterium tuberculosis at the bedside and during autopsy. Ann
Intern Med. 1995 Jun 15;122(12):922-5. PubMed PMID: 7755227.

1046: Punnotok J, Pumprueg U, Chakorn T. A comparison of two short course
tuberculosis chemotherapy regimens, both using Rifater during an intensive phase,
with a 3 year follow-up. J Med Assoc Thai. 1995 Jun;78(6):298-304. PubMed PMID:
7561554.

1047: Schwander S, RÃ¼sch-Gerdes S, Mateega A, Lutalo T, Tugume S, Kityo C,
Rubaramira R, Mugyenyi P, Okwera A, Mugerwa R. A pilot study of antituberculosis
combinations comparing rifabutin with rifampicin in the treatment of HIV-1
associated tuberculosis. A single-blind randomized evaluation in Ugandan patients
with HIV-1 infection and pulmonary tuberculosis. Tuber Lung Dis. 1995
Jun;76(3):210-8. PubMed PMID: 7548903.

1048: KaustovÃ¡ J, ChmelÃk M, EttlovÃ¡ D, Hudec V, LazarovÃ¡ H, RichtrovÃ¡ S. Disease

due to Mycobacterium kansasii in the Czech Republic: 1984-89. Tuber Lung Dis.
1995 Jun;76(3):205-9. PubMed PMID: 7548902.

1049: Sauret J, HernÃ¡ndez-Flix S, Castro E, HernÃ¡ndez L, Ausina V, Coll P.
Treatment of pulmonary disease caused by Mycobacterium kansasii: results of 18 vs
12 months' chemotherapy. Tuber Lung Dis. 1995 Apr;76(2):104-8. PubMed PMID:
7780090.

1050: Hoogkamp-Korstanje JA, Meis JF, Kissing J, van der Laag J, Melchers WJ.
Risk of cross-colonization and infection by Pseudomonas aeruginosa in a holiday
camp for cystic fibrosis patients. J Clin Microbiol. 1995 Mar;33(3):572-5. PubMed
PMID: 7751359; PubMed Central PMCID: PMC227992.

1051: MÃ¼hlberger F, Nturanye F, Nasbimana J, Portaels F.
[Ofloxacin-cycloserine-protionamide-INH combination against treatment refractory
lung tuberculosis]. Pneumologie. 1995 Feb;49(2):72-6. German. PubMed PMID:
7724506.

1052: Bose M, Gupta A, Banavalikar JN, Saha K. Dysregulation of homeostasis of
blood T-lymphocyte subpopulations persists in chronic multibacillary pulmonary
tuberculosis patients refractory to treatment. Tuber Lung Dis. 1995
Feb;76(1):59-64. PubMed PMID: 7718849.

1053: Qian Y, Zhao D, Liu S. [Implementation of national tuberculosis control
programme--focus on registration, case-management and treatment]. Zhonghua Jie He
He Hu Xi Za Zhi. 1995 Feb;18(1):16-8, 62. Chinese. PubMed PMID: 7600598.

1054: MÃ¼hlberger F, Nturanye F, Ladner J. [Clinical findings and follow-up of 100
each HIV-negative and HIV-positive cases of bacillary pulmonary tuberculosis in
Rwanda]. Pneumologie. 1995 Jan;49(1):27-31. German. PubMed PMID: 7892154.

1055: Wada M, Yosiyama T, Yosikawa M, Ogata H, Sugie T, Nakasono T, Sugita H.
[Six-month short course chemotherapy containing pyrazinamide for initial
treatment of pulmonary tuberculosis]. Kekkaku. 1994 Nov;69(11):671-80. Japanese.
PubMed PMID: 7837720.

1056: Hoheisel G, Teschler H, Chan BK, Chan CH, Luk WK, Sun AJ, Costabel U,
Konietzko N. [Roentgen findings in bronchial tuberculosis]. Pneumologie. 1994
Nov;48(11):788-92. German. PubMed PMID: 7824499.

1057: Gonzalez-Montaner LJ, Natal S, Yongchaiyud P, Olliaro P. Rifabutin for the
treatment of newly-diagnosed pulmonary tuberculosis: a multinational, randomized,
comparative study versus Rifampicin. Rifabutin Study Group. Tuber Lung Dis. 1994
Oct;75(5):341-7. PubMed PMID: 7841427.

1058: Hongthiamthong P, Riantawan P, Subhannachart P, Fuangtong P. Clinical
aspects and treatment outcome in HIV-associated pulmonary tuberculosis: an
experience from a Thai referral centre. J Med Assoc Thai. 1994 Oct;77(10):520-5.
PubMed PMID: 7745373.

1059: Dai YS, Li Q, Duanmu HJ. [The operational control studies of smear positive
tuberculosis cases with short-course chemotherapy under the full-course
management and supervision]. Zhonghua Jie He He Hu Xi Za Zhi. 1994
Oct;17(5):278-80, 318. Chinese. PubMed PMID: 7712567.

1060: Sekhon AS, Stein L, Garg AK, Black WA, Glezos JD, Wong C. Pulmonary
penicillosis marneffei: report of the first imported case in Canada.
Mycopathologia. 1994 Oct;128(1):3-7. PubMed PMID: 7708089.

1061: Toyota E, Suzuki T, Tagawa K, Takahara M, Ito M, Arai T, Kabe J, Baba H.
[Evaluation of the streptomycin twice weekly with INH and RFP for initial therapy
of pulmonary tuberculosis]. Kekkaku. 1994 Sep;69(9):559-63. Japanese. PubMed
PMID: 7967317.

1062: Kitahara Y, Ikeda A, Kajiki A, Maruyama M, Harada S, Harada Y, Takamoto M,
Ishibashi T. [An investigation on risk factors relating to the treatment
difficulty in originally treated pulmonary tuberculosis cases]. Kekkaku. 1994
Aug;69(8):503-11. Japanese. PubMed PMID: 7933776.

1063: Kumarvelu S, Prasad K, Khosla A, Behari M, Ahuja GK. Randomized controlled
trial of dexamethasone in tuberculous meningitis. Tuber Lung Dis. 1994
Jun;75(3):203-7. PubMed PMID: 7919313.

1064: Macnab MF, Bohmer PD, Seager JR. Evaluation of the 3-drug combination,
Rifater, versus 4-drug therapy in the ambulatory treatment of tuberculosis in
Cape Town. S Afr Med J. 1994 Jun;84(6):325-8. PubMed PMID: 7740377.

1065: Wallace RJ Jr, Brown BA, Griffith DE, Girard WM, Murphy DT, Onyi GO,
Steingrube VA, Mazurek GH. Initial clarithromycin monotherapy for Mycobacterium
avium-intracellulare complex lung disease. Am J Respir Crit Care Med. 1994
May;149(5):1335-41. PubMed PMID: 8173775.

1066: Wallace RJ Jr, Dunbar D, Brown BA, Onyi G, Dunlap R, Ahn CH, Murphy DT.
Rifampin-resistant Mycobacterium kansasii. Clin Infect Dis. 1994
May;18(5):736-43. PubMed PMID: 8075262.

1067: Pegues DA, Carson LA, Tablan OC, FitzSimmons SC, Roman SB, Miller JM,
Jarvis WR. Acquisition of Pseudomonas cepacia at summer camps for patients with
cystic fibrosis. Summer Camp Study Group. J Pediatr. 1994 May;124(5 Pt
1):694-702. PubMed PMID: 7513755.

1068: Boersma WG, Puister SM, van Altena R, de Vries-Hospers HG, Molinari M,
KoÃ«ter GH. Clinical and bacteriological efficacy and tolerability of FCE 22891 in
patients with exacerbations of chronic obstructive pulmonary disease. Antimicrob
Agents Chemother. 1994 Apr;38(4):872-5. PubMed PMID: 8031062; PubMed Central

PMCID: PMC284559.

1069: Kawashima T, Kioi S, Arakawa M. [Two cases of lung infection due to
Mycobacterium chelonae subsp. abscessus]. Kansenshogaku Zasshi. 1994
Mar;68(3):416-20. Review. Japanese. PubMed PMID: 8176285.

1070: Pavlova MV, Ivanova lA, Titarenko OT, Perova TL. [Comparative effectiveness
of etiopathogenetic therapy in adolescents with destructive pulmonary
tuberculosis]. Probl Tuberk. 1994;(2):45-6. Russian. PubMed PMID: 8036221.

1071: Pavlova MV. [Pathogenetic therapy of pulmonary tuberculosis in
adolescents]. Probl Tuberk. 1994;(3):19-21. Russian. PubMed PMID: 7937663.

1072: Valentini G, Forlani S, Zompatori M, Longi R. [Computerized tomography in
the study of tuberculosis]. Radiol Med. 1993 Dec;86(6):820-5. Italian. PubMed
PMID: 8296002.

1073: Kennedy N, Fox R, Kisyombe GM, Saruni AO, Uiso LO, Ramsay AR, Ngowi FI,
Gillespie SH. Early bactericidal and sterilizing activities of ciprofloxacin in
pulmonary tuberculosis. Am Rev Respir Dis. 1993 Dec;148(6 Pt 1):1547-51. PubMed
PMID: 8256898.

1074: Sandin RL, Isada CM, Hall GS, Tomford JW, Rutherford I, Rogers AL,
Washington JA. Aberrant Histoplasma capsulatum. Confirmation of identity by a
chemiluminescence-labeled DNA probe. Diagn Microbiol Infect Dis. 1993
Oct;17(3):235-8. PubMed PMID: 8112034.

1075: Ray D, Saha K, Krishna KS. Serum immunoglobulin E response in sputum
positive patients with pulmonary tuberculosis. Indian J Med Res. 1993
Jul;97:151-3. PubMed PMID: 8406639.

1076: Wada M, Seita A, Mori T, Ogata H, Sugie T, Sugita H. [Retreatment of

pulmonary tuberculosis--duration of chemotherapy]. Kekkaku. 1993
Jul;68(7):469-78. Japanese. PubMed PMID: 8361115.

1077: Zych D, Pawlicka L, Zielinski J. Inhaled budesonide vs
prednisone in the
maintenance treatment of pulmonary sarcoidosis. Sarcoidosis. 1993
Mar;10(1):56-61. PubMed PMID: 8134718.

1078: He GJ. [A comparative study of rifapentine treatment and three
years
follow-up on initial pulmonary tuberculous]. Zhonghua Jie He He Hu
Xi Za Zhi.
1993 Feb;16(2):73-6, 122. Chinese. PubMed PMID: 8251027.

1079: Peng DP. [Assessment on the epidemiological efficiency and
treatment of
smear positive pulmonary tuberculosis]. Zhonghua Jie He He Hu Xi Za
Zhi. 1993
Feb;16(2):85-7, 123. Chinese. PubMed PMID: 8221958.

1080: Liu XR. [A study on the relapse and its potential causes in
pulmonary
tuberculosis]. Zhonghua Jie He He Hu Xi Za Zhi. 1993 Feb;16(2):69-
72, 122.
Chinese. PubMed PMID: 8221953.

1081: Minnikin DE, Bolton RC, Hartmann S, Besra GS, Jenkins PA,
Mallet AI,
Wilkins E, Lawson AM, Ridell M. An integrated procedure for the
direct detection
of characteristic lipids in tuberculosis patients. Ann Soc Belg Med
Trop. 1993;73
Suppl 1:13-24. PubMed PMID: 8129475.

1082: Grosset JH. Treatment of tuberculosis in HIV infection. Tuber
Lung Dis.
1992 Dec;73(6):378-83. Review. PubMed PMID: 1337993.

1083: Migliori GB, Borghesi A, Adriko C, Manfrin V, Okware S,
Naamara W,
Bartoloni A, Neri M, Acocella G. Tuberculosis and HIV infection
association in a
rural district of northern Uganda: epidemiological and clinical
considerations.
Tuber Lung Dis. 1992 Oct;73(5):285-90. PubMed PMID: 1493236.

1084: Beck-SaguÃ© C, Dooley SW, Hutton MD, Otten J, Breeden A,
Crawford JT,

Pitchenik AE, Woodley C, Cauthen G, Jarvis WR. Hospital outbreak of multidrug-resistant Mycobacterium tuberculosis infections. Factors in
transmission to staff and HIV-infected patients. JAMA. 1992 Sep 9;268(10):1280-6.
PubMed PMID: 1507374.

1085: Behera D, Dash RJ. Adreno-cortical reserve in pulmonary tuberculosis. J
Assoc Physicians India. 1992 Aug;40(8):520-1. PubMed PMID: 1339211.

1086: O'Donnell LJ, Arvind AS, Hoang P, Cameron D, Talbot IC, Jewell DP,
Lennard-Jones JE, Farthing MJ. Double blind, controlled trial of 4-aminosalicylic
acid and prednisolone enemas in distal ulcerative colitis. Gut. 1992
Jul;33(7):947-9. PubMed PMID: 1644335; PubMed Central PMCID: PMC1379410.

1087: Pretet S, Lebeaut A, Parrot R, Truffot C, Grosset J, Dinh-Xuan AT. Combined
chemotherapy including rifabutin for rifampicin and isoniazid resistant pulmonary
tuberculosis. G.E.T.I.M. (Group for the Study and Treatment of Resistant
Mycobacterial Infection). Eur Respir J. 1992 Jun;5(6):680-4. PubMed PMID:
1321054.

1088: Wolde K, Lema E, Roscigno G, Abdi A. Fixed dose combination short course
chemotherapy in the treatment of pulmonary tuberculosis. Ethiop Med J. 1992
Apr;30(2):63-8. PubMed PMID: 1345030.

1089: Murate T, Shimokata K, Watanabe A, Ichiyama S, Saito H, Yamori S, Nomura F,
Iwahara T, Sakai S, Nakanishi K, et al. Chest roentgenogram classification and
clinical parameters in patients with active pulmonary tuberculosis. Intern Med.
1992 Feb;31(2):185-8. PubMed PMID: 1600264.

1090: Giwercman B, Meyer C, Lambert PA, Reinert C, HÃ¸iby N. High-level
beta-lactamase activity in sputum samples from cystic fibrosis patients during
antipseudomonal treatment. Antimicrob Agents Chemother. 1992 Jan;36(1):71-6.
PubMed PMID: 1590704; PubMed Central PMCID: PMC189229.

1091: MihÄƒilescu P, Hartia V, Didilescu C, Ibraim E, Lugoji D, Chiotan D, Sasu
AM, Evian N. [The exclusively outpatient treatment of pulmonary tuberculosis
under the conditions in Romania]. Pneumoftiziologia. 1992 Jan-Mar;41(1):5-8.
Romanian. PubMed PMID: 1299402.

1092: Ojeniyi B, Birch-Andersen A, Mansa B, Rosdahl VT, HÃ¸iby N. Morphology of
Pseudomonas aeruginosa phages from the sputum of cystic fibrosis patients and
from the phage typing set. An electron microscopy study. APMIS. 1991
Oct;99(10):925-30. PubMed PMID: 1930965.

1093: Nowak D, Radenbach D, Kirsten D, Magnussen H. [Tuberculosis treatment
today]. Z Gesamte Inn Med. 1991 Aug;46(10-11):404-9. German. PubMed PMID:
1926948.

1094: Al-Hajjaj MS, Pandya L, Marie AA, Madani AA, Al-Sharif N, Al-Majed S.
Pulmonary tuberculosis in Saudi Arabia: A retrospective study of 1566 patients.
Ann Saudi Med. 1991 Jul;11(4):443-7. PubMed PMID: 17590764.

1095: Levine B, Chaisson RE. Mycobacterium kansasii: a cause of treatable
pulmonary disease associated with advanced human immunodeficiency virus (HIV)
infection. Ann Intern Med. 1991 May 15;114(10):861-8. PubMed PMID: 1673053.

1096: Pandya L, Al-Sharif N, Maraey A, Al-Majed S, El-Sakka M. Pulmonary
tuberculosis in diabetic patients. Ann Saudi Med. 1991 May;11(3):293-6. PubMed
PMID: 17588106.

1097: Yamashita M, Morikawa K, Kuroda M. [Clinical implications of the O-antigen
serovar E and drug resistance in persistent Pseudomonas infection]. Rinsho Byori.
1991 May;39(5):541-7. Japanese. PubMed PMID: 1906555.

1098: Tsukamura M. [Chemotherapeutic regimens that were considered effective to
cure pulmonary infection caused by Mycobacterium avium-Mycobacterium
intracellulare complex]. Kekkaku. 1991 May;66(5):375-80. Japanese. PubMed PMID:
1875580.

1099: Kino C, Sato M, Iwasaki T, Takase A, Kinoshita T, Yamaguchi T, Kinjo T, Odo
A, Hara M, Hirata H. [Nine month chemotherapy with INH and rifampicin for
non-cavitary pulmonary tuberculosis]. Kekkaku. 1991 Apr;66(4):291-7. Japanese.
PubMed PMID: 2051705.

1100: Nakae I, Nakatani K, Inoue S, Takahasi K, Ikeda N, Matsumoto T, Ozawa S,
Sakatani M, Kita N, Tanaka S. [Therapeutic effect of ofloxacin on intractable
pulmonary tuberculosis and ofloxacin resistance of tubercle bacilli isolated from
the patients. Chest DIsease Cooperative Study Unit of National Sanatoriums in
Kinki District]. Kekkaku. 1991 Apr;66(4):299-307. Japanese. PubMed PMID: 1904960.

1101: Wang CL. [Evaluation of administrative practices for the registration of
outpatients with pulmonary tuberculosis]. Zhonghua Jie He He Hu Xi Za Zhi. 1991
Apr;14(2):71-3, 125. Chinese. PubMed PMID: 1879018.

1102: Girgis NI, Farid Z, Kilpatrick ME, Sultan Y, Mikhail IA. Dexamethasone
adjunctive treatment for tuberculous meningitis. Pediatr Infect Dis J. 1991
Mar;10(3):179-83. PubMed PMID: 2041662.

1103: Kilbourn JP. Phenotypic conversion of Pseudomonas aeruginosa. J Clin
Microbiol. 1991 Feb;29(2):416-7. PubMed PMID: 1901068; PubMed Central PMCID:
PMC269783.

1104: MoÅ›-Antkowiak R. [Tuberculosis in patients with alcoholism, peptic ulcer,
diabetes mellitus or mental disorders]. Pneumonol Alergol Pol. 1991;59(1-2):43-7.
Polish. PubMed PMID: 1843886.

1105: Taylor LQ, Williams AJ, Santiago S. Pulmonary disease caused
by
Mycobacterium asiaticum. Tubercle. 1990 Dec;71(4):303-5. PubMed
PMID: 2267684.

1106: Kameda K, Kawabata S, Masuda N. [Follow-up study of short
course
chemotherapy of pulmonary tuberculosis complicated with diabetes
mellitus].
Kekkaku. 1990 Dec;65(12):791-803. Japanese. PubMed PMID: 2077255.

1107: Toppet M, Malfroot A, Derde MP, Toppet V, Spehl M, Dab I.
Corticosteroids
in primary tuberculosis with bronchial obstruction. Arch Dis Child.
1990
Nov;65(11):1222-6. PubMed PMID: 2248533; PubMed Central PMCID:
PMC1792610.

1108: Yew WW, Kwan SY, Ma WK, Khin MA, Chau PY. In-vitro activity of
ofloxacin
against Mycobacterium tuberculosis and its clinical efficacy in
multiply
resistant pulmonary tuberculosis. J Antimicrob Chemother. 1990
Aug;26(2):227-36.
PubMed PMID: 2120177.

1109: Chen QY, Liu TY. [An approach to the mode of case findings of
pulmonary
tuberculosis in countryside]. Zhonghua Jie He He Hu Xi Za Zhi. 1990
Aug;13(4):207-8, 253-4. Chinese. PubMed PMID: 2090348.

1110: Thomas A, Balakrishnan A, Nagarajan M, Prabhakar R, Tripathy
SP, Christian
M, Somasundaram PR. Controlled clinical trial of two multidrug
regimens with and
without rifampin in highly bacilliferous BL/LL south Indian
patients: a five-year
report. Int J Lepr Other Mycobact Dis. 1990 Jun;58(2):273-80. PubMed
PMID:
2198314.

1111: Fegan M, Francis P, Hayward AC, Davis GH, Fuerst JA.
Phenotypic conversion
of Pseudomonas aeruginosa in cystic fibrosis. J Clin Microbiol. 1990
Jun;28(6):1143-6. PubMed PMID: 2116444; PubMed Central PMCID:
PMC267893.

1112: Cowie RL, Brink BA. Short-course chemotherapy for pulmonary tuberculosis
with a rifampicin-isoniazid-pyrazinamide combination tablet. S Afr Med J. 1990
Apr 21;77(8):390-1. PubMed PMID: 2184527.

1113: O'Brien RJ, Geiter LJ, Lyle MA. Rifabutin (ansamycin LM427) for the
treatment of pulmonary Mycobacterium avium complex. Am Rev Respir Dis. 1990
Apr;141(4 Pt 1):821-6. PubMed PMID: 2158257.

1114: Nowak D, Radenbach D, Magnussen H. [In 76% of patients with active
tuberculosis treated with triple therapy (isoniazid-rifampicin-pyrazinamide)
cultural conversion precedes microscopic conversion]. Pneumologie. 1990 Feb;44
Suppl 1:497-8. German. PubMed PMID: 2114634.

1115: Breyer O, Neher A, Feldmann K, Shrestha HB. [Control of tuberculosis in
Nepal. A project. Initial results, experiences]. Pneumologie. 1990 Feb;44 Suppl
1:475-6. German. PubMed PMID: 2114632.

1116: Kharakter ZhZ, Mazhak KD, Pavlenko AV. [The role of genetically determined
haptoglobin phenotypes in patients with destructive pulmonary tuberculosis].
Probl Tuberk. 1990;(7):50-2. Russian. PubMed PMID: 2235952.

1117: MezhebovskiÄ- VR. [Effectiveness of the treatment of patients with pulmonary
tuberculosis using a liver protector zixoryn]. Probl Tuberk. 1990;(10):32-5.
Russian. PubMed PMID: 2080156.

1118: Palmero DJ, Teres RI, Eiguchi K. Pulmonary disease due to Mycobacterium
terrae. Tubercle. 1989 Dec;70(4):301-3. PubMed PMID: 2626806.

1119: Hiro Y. [Pulmonary tuberculosis and diabetes mellitus--report of the 29th B
series of controlled trials of chemotherapy. Cooperative Study Unit of
Chemotherapy of Tuberculosis of National Sanatoria in Japan (CSUCTNS)]. Kekkaku.
1989 Nov;64(11):699-705. Japanese. PubMed PMID: 2593461.

1120: Harada S, Takamoto M, Harada Y, Ninomiya H, Maruyama M, Ishibashi T,
Shinoda A. [Clinico-immunological studies of pulmonary tuberculosis in the
elderly]. Kekkaku. 1989 Aug;64(8):529-36. Japanese. PubMed PMID: 2811010.

1121: [A short course of chemotherapy in newly diagnosed and retreatment of
smear-positive pulmonary tuberculosis in a rural area]. Zhonghua Jie He He Hu Xi
Za Zhi. 1989 Aug;12(4):220-2, 254-5. Chinese. PubMed PMID: 2636947.

1122: Tsukamura M, Ichiyama S, Miyachi T. Superiority of enviomycin or
streptomycin over ethambutol in initial treatment of lung disease caused by
Mycobacterium avium complex. Chest. 1989 May;95(5):1056-8. PubMed PMID: 2539957.

1123: Johnson PC, Sarosi GA. Community-acquired fungal pneumonias. Semin Respir
Infect. 1989 Mar;4(1):56-63. Review. PubMed PMID: 2652235.

1124: Fortic B. [Clinical study of a 6-month chemotherapy regimen in the
treatment of pulmonary tuberculosis. Results approximately 2 years after
completion of chemotherapy]. Plucne Bolesti. 1989 Jan-Jun;41(1-2):46-57.
Croatian. PubMed PMID: 2798572.

1125: Blaha H, Heilig B, Schreiber MA, Styblo K. Surveillance of diagnostic and
treatment measures in Bavaria, 1974-1976. Results 2 and 5 years after the start
of chemotherapy. Tubercle. 1988 Dec;69(4):255-65. PubMed PMID: 3257004.

1126: Strang JI, Kakaza HH, Gibson DG, Allen BW, Mitchison DA, Evans DJ, Girling
DJ, Nunn AJ, Fox W. Controlled clinical trial of complete open surgical drainage
and of prednisolone in treatment of tuberculous pericardial effusion in Transkei.
Lancet. 1988 Oct 1;2(8614):759-64. PubMed PMID: 2901610.

1127: Onwubalili JK, Scott GM. Immune status in tuberculosis and response to
treatment. Tubercle. 1988 Jun;69(2):81-94. PubMed PMID: 3188236.

1128: Tsukamura M, Ichiyama S. Comparison of antituberculosis drug regimens for
lung disease caused by Mycobacterium avium complex. Chest. 1988 Apr;93(4):821-3.
PubMed PMID: 3349841.

1129: Baba H, Shinkai A, Izuchi R, Azuma Y. [Long-term results of short-course
chemotherapy in pulmonary tuberculosis (final report). The third study:
comparative study of two regimens, 6RHZ and -6RHS (treatment was continued for 6
months after negative conversion of sputum by culture)]. Kekkaku. 1988
Apr;63(4):239-46. Japanese. PubMed PMID: 3136270.

1130: Hornick DB, Dayton CS, Bedell GN, Fick RB Jr. Nontuberculous mycobacterial
lung disease. Substantiation of a less aggressive approach. Chest. 1988
Mar;93(3):550-5. PubMed PMID: 3342664.

1131: Tsukamura M. Evidence that antituberculosis drugs are really effective in
the treatment of pulmonary infection caused by Mycobacterium avium complex. Am
Rev Respir Dis. 1988 Jan;137(1):144-8. PubMed PMID: 3337455.

1132: Strang JI, Kakaza HH, Gibson DG, Girling DJ, Nunn AJ, Fox W. Controlled
trial of prednisolone as adjuvant in treatment of tuberculous constrictive
pericarditis in Transkei. Lancet. 1987 Dec 19;2(8573):1418-22. PubMed PMID:
2891992.

1133: Ichiyama S, Tsukamura M. Ofloxacin and the treatment of pulmonary disease
due to Mycobacterium fortuitum. Chest. 1987 Dec;92(6):1110-2. PubMed PMID:
3479304.

1134: Aitken ML, Anderson KM, Albert RK. Is the tuberculosis screening program of

hospital employees still required? Am Rev Respir Dis. 1987
Oct;136(4):805-7.
PubMed PMID: 3116895.

1135: Nowak D, Radenbach D, Magnussen H. [Chemotherapy of pulmonary tuberculosis.
Sputum culture conversion in 8 weeks in 84% of patients]. Dtsch Med Wochenschr.
1987 Sep 4;112(36):1367-70. German. PubMed PMID: 3622281.

1136: Laursen LC, Faurschou P, Pals H, Svendsen UG, Weeke B. Intramuscular
betamethasone dipropionate vs. oral prednisolone in hay fever patients. Allergy.
1987 Apr;42(3):168-72. PubMed PMID: 3592139.

1137: Etzkorn ET, Aldarondo S, McAllister CK, Matthews J, Ognibene AJ. Medical
therapy of Mycobacterium avium-intracellulare pulmonary disease. Am Rev Respir
Dis. 1986 Sep;134(3):442-5. PubMed PMID: 3752699.

1138: Onadeko BO, Awotedu AA, Ogunbanjo BO, Aderinto EB. Controlled clinical
trial of three short-course regimens of chemotherapy for pulmonary tuberculosis
in Nigeria--a preliminary report. Tubercle. 1986 Sep;67(3):189-95. PubMed PMID:
3535187.

1139: Ahn CH, Ahn SS, Anderson RA, Murphy DT, Mammo A. A four-drug regimen for
initial treatment of cavitary disease caused by Mycobacterium avium complex. Am
Rev Respir Dis. 1986 Sep;134(3):438-41. PubMed PMID: 3530069.

1140: Mitchison DA, Nunn AJ. Influence of initial drug resistance on the response
to short-course chemotherapy of pulmonary tuberculosis. Am Rev Respir Dis. 1986
Mar;133(3):423-30. PubMed PMID: 2420242.

1141: Huang M. [Studies on sputum conversion of smear-positive, previously
untreated pulmonary tuberculosis: a comparison of short-course and standard
chemotherapy regimens]. Zhonghua Jie He He Hu Xi Xi Ji Bing Za Zhi. 1986
Feb;9(1):1-5, 61. Chinese. PubMed PMID: 3743274.

1142: Leads from the MMWR. Bacteriologic conversion of sputum among tuberculosis
patients--United States. JAMA. 1986 Jan 24-31;255(4):451, 455-6. PubMed PMID:
3941524.

1143: Centers for Disease Control (CDC). Bacteriologic conversion of sputum among
tuberculosis patients--United States. MMWR Morb Mortal Wkly Rep. 1985 Dec
13;34(49):747-50. PubMed PMID: 3934517.

1144: Bezel R, Salfinger M, BrÃ¤ndli O. [The transmission of mycobacteria through
the fiberoptic bronchoscope]. Schweiz Med Wochenschr. 1985 Sep 28;115(39):1360-5.
German. PubMed PMID: 3934749.

1145: Kan GQ, Zhang LX, Wu JC, Ma ZI, Liu CW, Sun FZ. Supervised intermittent
chemotherapy for pulmonary tuberculosis in a rural area of China. Tubercle. 1985
Mar;66(1):1-7. PubMed PMID: 3984034.

1146: Tsukamura M, Nakamura E, Yoshii S, Amano H. Therapeutic effect of a new
antibacterial substance ofloxacin (DL8280) on pulmonary tuberculosis. Am Rev
Respir Dis. 1985 Mar;131(3):352-6. PubMed PMID: 3856412.

1147: Snider DE Jr, Cohn DL, Davidson PT, Hershfield ES, Smith MH, Sutton FD Jr.
Standard therapy for tuberculosis 1985. Chest. 1985 Feb;87(2 Suppl):117S-124S.
Review. PubMed PMID: 3881230.

1148: Cavallo G. [The etiological agent of tuberculosis]. Minerva Med. 1984 Mar
17;75(11):531-4. Italian. PubMed PMID: 6424053.

1149: Kim TC, Blackman RS, Heatwole KM, Kim T, Rochester DF. Acid-fast bacilli in
sputum smears of patients with pulmonary tuberculosis. Prevalence and
significance of negative smears pretreatment and positive smears post-treatment.
Am Rev Respir Dis. 1984 Feb;129(2):264-8. PubMed PMID: 6421211.

1150: [A controlled trial of 6-month and 12-month regimens after negative
conversion of sputum in the original treatment of pulmonary tuberculosis. Report
of the 21st series of controlled trials of chemotherapy. Cooperative Study Unit
of Chemotherapy of Tuberculosis of National Sanatoria in Japan (CSUCTNS)].
Kekkaku. 1984 Feb;59(2):81-9. Japanese. PubMed PMID: 6379258.

1151: Fischer P, Schilling W. [Therapeutic results in lung diseases caused by
atypical mycobacteria]. Z Erkr Atmungsorgane. 1984;162(1):26-9. German. PubMed
PMID: 6202070.

1152: Randhawa HS, Khan ZU, Gaur SN. Blastomyces dermatitidis in India: first
report of its isolation from clinical material. Sabouraudia. 1983
Sep;21(3):215-21. PubMed PMID: 6415825.

1153: Powell-Jackson PR, Gray BJ, Heaton RW, Costello JF, Williams R, English J.
Adverse effect of rifampicin administration on steroid-dependent asthma. Am Rev
Respir Dis. 1983 Aug;128(2):307-10. PubMed PMID: 6349444.

1154: BÃ¶lcskei P. [Pulmonary tuberculosis. 2. Therapy]. Fortschr Med. 1983 Jun
16;101(23):1075-80, 1090. German. PubMed PMID: 6411560.

1155: Nagasawa S. [Evaluation of short-term chemotherapy of pulmonary
tuberculosis (2). (a trial of 6-month chemotherapy following negative conversion
of the sputum)--a report on the 19th and 20th controlled trials series A of
chemotherapy by the Cooperative Study Unit, Japan National Sanatoria)]. Kekkaku.
1983 Jun;58(6):347-53. Japanese. PubMed PMID: 6353032.

1156: Study of chemotherapy regimens of 5 and 7 months' duration and the role of
corticosteroids in the treatment of sputum-positive patients with pulmonary
tuberculosis in South India. Tubercle. 1983 Jun;64(2):73-91. PubMed PMID:
6351390.

1157: Miller SD. A comparative evaluation of drug combinations used in the
treatment of pulmonary tuberculosis. S Afr Med J. 1983 May 14;63(20):764-6.
PubMed PMID: 6845095.

1158: Banks J, Hunter AM, Campbell IA, Jenkins PA, Smith AP. Pulmonary infection
with Mycobacterium kansasii in Wales, 1970-9: review of treatment and response.
Thorax. 1983 Apr;38(4):271-4. PubMed PMID: 6867979; PubMed Central PMCID:
PMC459534.

1159: Favez G, Leuenberger P. [Priorities among antitubercular measures:
epidemiologic arguments]. Schweiz Med Wochenschr. 1983 Jan 22;113(3):96-9.
French. PubMed PMID: 6828845.

1160: Robertson CE, Ford MJ, Munro JF, Gould JC, Langford DT, Bernstein LS. The
efficacy of a new formulation of trimethoprim and sulphadiazine in acute
exacerbations of chronic bronchitis. Methods Find Exp Clin Pharmacol.
1983;5(2):127-9. PubMed PMID: 6876943.

1161: Law MR, Holt HA, Reeves DS, Hodson ME. Cefaclor and amoxycillin in the
treatment of infective exacerbations of chronic bronchitis. J Antimicrob
Chemother. 1983 Jan;11(1):83-8. PubMed PMID: 6337988.

1162: Cassels A, Heineman E, LeClerq S, Gurung PK, Rahut CB. Tuberculosis
case-finding in Eastern Nepal. Tubercle. 1982 Sep;63(3):175-85. PubMed PMID:
7179484.

1163: Centers for Disease Control (CDC). Bacteriologic conversion of sputum among
tuberculosis patients. MMWR Morb Mortal Wkly Rep. 1982 Aug 27;31(33):454, 459-62.
PubMed PMID: 6815461.

1164: Dornetzhuber V, Martis R, Burjanova B, Pavukova K, Turzova M, Vincurova M.
Pulmonary mycobacteriosis caused by Mycobacterium xenopi. Report of a case. Eur J
Respir Dis. 1982 Jul;63(4):293-7. PubMed PMID: 7117426.

1165: Catanzaro A. Nosocomial tuberculosis. Am Rev Respir Dis. 1982 May;125(5):559-62. PubMed PMID: 7081816.

1166: Ross JB, Levine B, Catanzaro A, Einstein H, Schillaci R, Friedman PJ.
Ketoconazole for treatment of chronic pulmonary coccidioidomycosis. Ann Intern
Med. 1982 Apr;96(4):440-3. PubMed PMID: 6279005.

1167: Tabachnik NF, Blackburn P, Peterson CM, Cerami A. Protein binding of
N-2-mercaptoethyl-1,3-diaminopropane via mixed disulfide formation after oral
administration of WR 2721. J Pharmacol Exp Ther. 1982 Feb;220(2):243-6. PubMed
PMID: 6276531.

1168: Detre KM, Ware J, Mantel N. Are clinical trials in coronary heart disease
oversold or undersold? Circulation. 1981 Oct;64(4):667-8. PubMed PMID: 7023742.

1169: Ahn CH, Lowell JR, Ahn SS, Ahn S, Hurst GA. Chemotherapy for pulmonary
disease due to Mycobacterium kansasii: efficacies of some individual drugs. Rev
Infect Dis. 1981 Sep-Oct;3(5):1028-34. PubMed PMID: 7339800.

1170: Good JT Jr, Iseman MD, Davidson PT, Lakshminarayan S, Sahn SA. Tuberculosis
in association with pregnancy. Am J Obstet Gynecol. 1981 Jul 1;140(5):492-8.
PubMed PMID: 7246682.

1171: Gwinup G, Randazzo G, Elias A. The influence of vitamin D intake on serum
calcium in tuberculosis. Acta Endocrinol (Copenh). 1981 May;97(1):114-7. PubMed
PMID: 6971551.

1172: A controlled trial of six months chemotherapy in pulmonary tuberculosis.

First Report: results during chemotherapy. British Thoracic Association. Br J Dis
Chest. 1981 Apr;75(2):141-53. PubMed PMID: 7023526.

1173: Zierski M, Bek E, Long MW, Snider DE Jr. Short-course (6 month) cooperative
tuberculosis study in Poland: results 18 months after completion of treatment. Am
Rev Respir Dis. 1980 Dec;122(6):879-89. PubMed PMID: 7006476.

1174: Pruzzo C, Debbia EA, Satta G. Identification of the major adherence ligand
of Klebsiella pneumoniae in the receptor for coliphage T7 and alteration of
Klebsiella adherence properties by lysogenic conversion. Infect Immun. 1980
Nov;30(2):562-71. PubMed PMID: 7002803; PubMed Central PMCID: PMC551348.

1175: Graybill JR, Lundberg D, Donovan W, Levine HB, Rodriguez MD, Drutz DJ.
Treatment of coccidioidomycosis with ketoconazole: clinical and laboratory
studies of 18 patients. Rev Infect Dis. 1980 Jul-Aug;2(4):661-73. PubMed PMID:
6255548.

1176: Bobrowitz ID. Reversible roentgenographic progression in the initial
treatment of pulmonary tuberculosis. Am Rev Respir Dis. 1980 Apr;121(4):735-42.
PubMed PMID: 7386981.

1177: Tousek J, ZÃtkovÃ¡ L, Trnka L, StaflovÃ¡ S, PapezovÃ¡ E, DrÃ¡pela J. Factors
influencing the effects of the initial phase of tuberculosis chemotherapy. Czech
Med. 1980;3(2):114-22. PubMed PMID: 7418568.

1178: Sieler R, Reech R. [Problems concerning old-aged patients with pulmonary
tuberculosis from the clinical point of view (author's transl)]. Z Erkr
Atmungsorgane. 1980;155(1):114-9. German. PubMed PMID: 7210735.

1179: Ahn CH, Lowell JR, Onstad GD, Ahn SS, Hurst GA. Elimination of
Mycobacterium intracellulare from sputum after bronchial hygiene. Chest. 1979
Oct;76(4):480-2. PubMed PMID: 477441.

1180: Long MW, Snider DE Jr, Farer LS. U.S. Public Health Service Cooperative
trial of three rifampin-isoniazid regimens in treatment of pulmonary
tuberculosis. Am Rev Respir Dis. 1979 Jun;119(6):879-94. PubMed
PMID: 110184.

1181: Horsfall PA, Plummer J, Allan WG, Girling DJ, Nunn AJ, Fox W. Double blind
controlled comparison of aspirin, allopurinol and placebo in the management of
arthralgia during pyrazinamide administration. Tubercle. 1979 Mar;60(1):13-24.
PubMed PMID: 377739.

1182: Rosenzweig DY. Pulmonary mycobacterial infections due to Mycobacterium
intracellulare-avium complex. Clinical features and course in 100 consecutive
cases. Chest. 1979 Feb;75(2):115-9. PubMed PMID: 421545.

1183: Quinteros CM, ChehÃ-n E, Wayar E, Ratner M, Amadio G. [Isoprodiane therapy
for previously untreated cavitating pulmonary tuberculosis (author's transl)].
Prax Klin Pneumol. 1979 Jan;33(1):45-7. German. PubMed PMID: 760101.

1184: Wernstedt L, Berntsson E, Thiringer G. Cefaclor therapy in acute
exacerbations of chronic bronchitis. Postgrad Med J. 1979;55 Suppl 4:56-8. PubMed
PMID: 548943.

1185: [Short course chemotherapy for pulmonary tuberculosis with duration for 6
months after sputum negative conversion. (Report 1)--Report of the 19--20th A
series of controlled trial of chemotherapy--(author's transl)]. Kekkaku. 1979
Jan;54(1):51-61. Japanese. PubMed PMID: 107362.

1186: Ito F, Okochi T, Yamazaki M, Yamamoto Y, Kondo K, Konishiike J, Asahi T.
[Sputum positive conversion among retreatment cases with pulmonary tuberculosis
converted to negative by the use of rifampicin (author's transl)]. Kekkaku. 1979
Jan;54(1):37-42. Japanese. PubMed PMID: 107360.

1187: Mok CK, Nandi P, Ong GB. Coexistent bronchogenic carcinoma and active
pulmonary tuberculosis. J Thorac Cardiovasc Surg. 1978 Oct;76(4):469-72. PubMed
PMID: 212648.

1188: Onadeko BO, Sofowora EO. Daily short course (6 months) chemotherapy for
treatment of pulmonary tuberculosis in Nigerians: a preliminary report. Afr J Med
Med Sci. 1978 Sep;7(3):175-81. PubMed PMID: 108938.

1189: Xalabarder E. The conversion of sputum in tuberculosis. Scand J Respir Dis
Suppl. 1978;102:68-9. PubMed PMID: 98840.

1190: Pines A, Nandi AR, Raafat H, Rahman M. Pivmecillinam and amoxycillin as
combined treatment in purulent exacerbations of chronic bronchitis. J Antimicrob
Chemother. 1977 Jul;3 Suppl B:141-8. PubMed PMID: 330483.

1191: Albu A, Hartia V, Esrig E, Galbenu M, Marcovici M, GÄƒlculescu A, Golub M.
[Clinical, radiological and bacteriological study of pulmonary tuberculosis with
bacteria showing drug resistance]. Rev Ig Bacteriol Virusol Parazitol Epidemiol
Pneumoftiziol Pneumoftiziol. 1977 Jul-Sep;26(3):151-60. Romanian. PubMed PMID:
201007.

1192: Dubois P, Gyselen A, Prignot J. Rifampim-combined chemotherapy in coal
worker's pneumoconio-tuberculosis. Am Rev Respir Dis. 1977 Feb;115(2):221-8.
PubMed PMID: 842936.

1193: Pines A, Nandi AR, Raafat H, Rahman M. Amoxycillin and co-trimoxazole in
acute purulent exacerbations of chronic bronchitis. Chemotherapy.
1977;23(1):58-64. PubMed PMID: 830516.

1194: Pines A, Nandi AR, Raafat H, Rahman M. Amoxycillin and co-trimoxazole in
acute purulent exacerbations of chronic bronchitis. Chemotherapy.
1977;23(1):58-64. PubMed PMID: 318978.

1195: Rouillon A, Perdrizet S, Parrot R. Transmission of tubercle bacilli: The
effects of chemotherapy. Tubercle. 1976 Dec;57(4):275-99. Review. PubMed PMID:
827837.

1196: Elkadi A, Salas R, Almond CH. Surgical treatment of atypical pulmonary
tuberculosis. J Thorac Cardiovasc Surg. 1976 Sep;72(3):435-40. PubMed PMID:
986519.

1197: PÅŸegalinschi N, Moscalu G. [Cured chronic pulmonary tuberculosis]. Rev Ig
Bacteriol Virusol Parazitol Epidemiol Pneumoftiziol Pneumoftiziol. 1976
Jul-Sep;25(3):179-82. Romanian. PubMed PMID: 188102.

1198: Sahn SA, Lakshminarayan S. Tuberculosis after corticosteroid therapy. Br J
Dis Chest. 1976 Jul;70(3):195-205. PubMed PMID: 136264.

1199: Ingold A. Conversion of benzylpenicillin to penicilloic acid in patients
with chronic bronchial infections. Chemotherapy. 1976;22(2):88-96. PubMed PMID:
1253635.

1200: Dandoy S, Hansen R. Tuberculosis care in general hospitals: Arizona's
experience. Am Rev Respir Dis. 1975 Dec;112(6):757-63. PubMed PMID: 1202996.

1201: Weiss T. [Sputum conversion and behavior of cavitations in the years
1967-1973]. Schweiz Med Wochenschr. 1975 Nov 29;105(48):1626-7. German. PubMed
PMID: 1215950.

1202: Inhaled corticosteroids compared with oral prednisone in patients starting
long-term corticosteroid therapy for asthma. A controlled trial by the British
Thoracic and Tuberculosis Association. Lancet. 1975 Sep 13;2(7933):469-73. PubMed
PMID: 51284.

1203: Onadeko BO, Sofowora EO. Comparative trial of thiacetazone with isoniazid
and paraaminosalicyclic acid (PAS) with isoniazid in the treatment of pulmonary
tuberculosis in Nigerians. J Trop Med Hyg. 1975 Sep;78(9):201-5. PubMed PMID:
57244.

1204: Snider DE Jr. Reactivation of tuberculosis in Oklahoma: 1970-1973. Chest.
1975 Jul;68(1):36-40. PubMed PMID: 1149527.

1205: Fairshter RD, Randazzo GP, Garlin J, Wilson AF. Failure of isoniazid
prophylaxis after exposure to isoniazid-resistant tuberculosis. Am Rev Respir
Dis. 1975 Jul;112(1):37-42. PubMed PMID: 807140.

1206: Short-course chemotherapy in pulmonary tuberculosis. A controlled trial by
the British Thoracic and Tuberculosis Association. Lancet. 1975 Jan
18;1(7899):119-24. PubMed PMID: 46047.

1207: Jessamine AG, Gale GL, Eidus L. Therapeutic efficacy of rifampin in newly
detected pulmonary tuberculosis. Can Med Assoc J. 1974 May 4;110(9):1033-7.
PubMed PMID: 4207234; PubMed Central PMCID: PMC1947749.

1208: Juchniewicz M, Radecki A, Kowalczyk H, Grzywacz W. [Effect of ethambutol
and rifampicin on sputum conversion in chronically infectious patients].
Gruzlica. 1974 May;42(5):397-400. Polish. PubMed PMID: 4464226.

1209: Sutalo I. [Speed of conversion sputum in the culture of sensitive and
resistant organisms]. Plucne Bolesti Tuberk. 1974;26 Suppl 2:56-9. Croatian.
PubMed PMID: 4216922.

1210: Tamura M, Yamazaki A, Tamura T, Takano S, Yamazaki M. [The relation between
bacilli negative conversion rate and the number of combined sensitive
antituberculous drugs in intermittent administration of rifampicin for severe
pulmonary tuberculosis]. Kekkaku. 1973 Jul;48(7):297-302. Japanese. PubMed PMID:

4200058.

1211: Doggett RG, Harrison GM. Pseudomonas aeruginosa: immune status in patients
with cystic fibrosis. Infect Immun. 1972 Oct;6(4):628-35. PubMed PMID: 4628901;
PubMed Central PMCID: PMC422584.

1212: Winter G, Viereck HJ. [Conversion of the sputum--only a relative success
measurement in the treatment of tuberculosis. Bacteriological study of 614
resection preparations]. Med Welt. 1972 Sep 16;23(38):1294-7. German. PubMed
PMID: 5078662.

1213: Jeanes CW, Jessamine AG, Eidus L. Treatment of chronic drug-resistant
pulmonary tuberculosis with rifampin and ethambutol. Can Med Assoc J. 1972 Apr
22;106(8):884-8. PubMed PMID: 4623743; PubMed Central PMCID: PMC1940582.

1214: Schonell M, Dorken E, Grzybowski S. Rifampin. Can Med Assoc J. 1972 Apr
8;106(7):783-6. Review. PubMed PMID: 4622757; PubMed Central PMCID: PMC1940515.

1215: Baba H, Takahashi R, Azuma Y. Rifampicin in the retreatment of severe
cavitary pulmonary tuberculosis. I. Some considerations on the sputum conversion
rate and the emergence of drug resistance. Kekkaku. 1971 Nov;46(11):429-35.
Japanese. PubMed PMID: 5004056.

1216: Freiman I, Geefhuysen J. Evaluation of intrathecal therapy with
streptomycin and hydrocortisone in tuberculous meningitis. J Pediatr. 1970
Jun;76(6):895-901. PubMed PMID: 5467641.

1217: Sehm G. [Sputum conversion and cavity closure]. Prax Pneumol. 1970
Jun;24(6):360-8. German. PubMed PMID: 5432551.

1218: KrzyÅ›ko R, Marciniak J, Zielniewicz B. [Value of controlled ambulatory and

clinical treatment in just discovered,TbB-positive patients during the first
treatment year, as the possibilities of sputum conversion]. Z Erkr Atmungsorgane
Folia Bronchol. 1970;132(1):49-51. German. PubMed PMID: 5203108.

1219: Poole G, Stradling P. Intermittent chemotherapy for tuberculosis in an
urban community. Br Med J. 1969 Jan 11;1(5636):82-4. PubMed PMID: 5761830; PubMed
Central PMCID: PMC1981997.

1220: VadÃ¡sz I. Effect of nandrolone decanoate on some forms of pulmonary
tuberculosis. Arzneimittelforschung. 1969 Jan;19(1):100-3. PubMed PMID: 5819351.

1221: Kent DC, Reid D, Sokolowski JW, Houk VN. Tuberculin conversion. The iceberg
of tuberculous pathogenesis. Arch Environ Health. 1967 Apr;14(4):580-4. PubMed
PMID: 4960792.

1222: Eule, Ewert. [The significance of persistent cavities after achieved sputum
conversion]. Z Tuberk Erkr Thoraxorg. 1966;125(3):168-71. German. PubMed PMID:
6014517.

1223: Eule H, Ewert EG. [Significance of rest caverns after sputum conversion].
Beitr Klin Erforsch Tuberk Lungenkr. 1966;133(5):323-35. German. PubMed PMID:
5973714.

1224: HSU KH, JEU F, JENKINS DE. STUDIES ON THE SPECIFIC TUBERCULIN REACTION.
TUBERCULIN CONVERSION IN TUBERCULOSIS CONTACTS. Am Rev Respir Dis. 1964
Jul;90:36-47. PubMed PMID: 14178625.

1225: NOVAK M, JANCIK E. SPUTUM CONVERSION IN CHRONIC TUBERCULOSIS WITH
POLYRESISTANT BACILLI. Acta Tuberc Pneumol Scand. 1964;45:301-19. PubMed PMID:
14234473.

1226: SULA L, LANGEROVA M. DRUG SENSITIVITY-RESISTANCE DETERMINATION AND SIMPLE
ENZYMATIC TESTS FOR THE DIFFERENTIATION OF MYCOBACTERIA. Bull World Health Organ.
1963;29:579-88. PubMed PMID: 14102035; PubMed Central PMCID: PMC2555064.

1227: STRANDGAARD E. Sputum conversion on chemotherapy in fresh cases of
pulmonary tuberculosis. Acta Tuberc Pneumol Scand. 1962;41:187-91. PubMed PMID:
13917617.

1228: SELKON JB, FOX W, GANGADHARAM PR, RAMACHANDRAN K, RAMAKRISHNAN CV, VELU S.
Rate of inactivation of isoniazid in South Indian patients with pulmonary
tuberculosis. 2. Clinical implications in the treatment of pulmonary tuberculosis
with isoniazid either alone or in combination with PAS. Bull World Health Organ.
1961;25:779-92. PubMed PMID: 13910443; PubMed Central PMCID: PMC2555638.

1229: BARBER LM, SAMSON PC, DUGAN D. Primary pulmonary resection for
tuberculosis; medical and economic aspects in a small sanatorium. Calif Med. 1953
Apr;78(4):282-4. PubMed PMID: 13042660; PubMed Central PMCID: PMC1521819.
