# Supplementary material for: The effects of anti-inflammatory agents as host-directed adjunct treatment of tuberculosis in humans: a systematic review and meta-analysis
Source: Respir Res. 2020 Aug 26;21:223. doi: 10.1186/s12931-020-01488-9 (PMC7448999; doi:10.1186/s12931-020-01488-9)
Supplement: Supplementary file 2 — Additional file 2: Supplementary Table 1. Quality assessment of included studies according to the Jadad scale. Supplementary Table 2. Risk of bias assessment of individual studies included. Supplementary Table 3. Frequency of outcome assessment and follow-up duration. Supplementary Table 4a. Outcome measurement at end point by allocation. Supplementary Table 4b. Outcome measurement at end point by allocation. Supplementary Table 4c. Outcome measurement at end point by allocation. Supplementary Figure 1. Funnel plot for aggregate patient data meta-analysis of sputum smear conversion rate conversion in vitamin D supplemented randomized controlled trials. Supplementary Figure 2. Funnel plot for aggregate patient data meta-analysis of sputum smear conversion rate in other anti-inflammatory HDT agents supplemented randomized controlled trials. [file 12931_2020_1488_MOESM2_ESM.zip › Additional File 2_ Suppl Tables & Figures.docx]

**The effects of anti-inflammatory agents as host-directed adjunct treatment of Tuberculosis in humans: A systematic review and meta-analysis**

Frank EA Hayford^1, 2*^, Robin C Dolman^1^, Renee Blaauw^3^, Arista Nienaber^1^, Cornelius M Smuts^1^, Linda Malan^1^, Cristian Ricci^1, 4^

**TABLES**

**Supplementary Table 1: Quality assessment of included studies according to the Jadad scale**

| **NO.** | **Included studies** | **Study design** | **Randomization criteria score (0/1/2)** | **Blinding**  **score (0/1/2)** | **Withdrawals /dropouts described score (0/1)** | **Total Jadad score** |
| --- | --- | --- | --- | --- | --- | --- |
| 1 | **Daley 2015 [16]** | RCT | 2 | 2 | 1 | 5 |
| 2 | **Farazi et al, 2017 [23]** | RCT | 2 | 1 | 1 | 5 |
| 3 | **Martineau 2017 [21]** | RCT | 2 | 2 | 1 | 5 |
| 4 | **Mily 2015 [18]** | RCT | 2 | 2 | 1 | 5 |
| 5 | **Nursyam 2006 [44]** | RCT | 1 | 2 | 0 | 3 |
| 6 | **Ralph 2013 [20]** | RCT | 2 | 2 | 1 | 5 |
| 7 | **Salahuddin 2013 [14]** | RCT | 2 | 2 | 1 | 5 |
| 8 | **Tukvadze 2015 [46]** | RCT | 2 | 2 | 1 | 5 |
| 9 | **Wejse 2009 [45]** | RCT | 2 | 2 | 1 | 5 |
| 10 | **Ganmaa 2017 [15]** | RCT | 2 | 2 | 1 | 5 |
| 11 | **Johnson 2003 [25]** | RCT | 2 | 2 | 1 | 5 |
| 12 | **Mayanja-Kizza 2005 [47]** | RCT | 2 | 2 | 1 | 5 |
| 13 | **Pedral-Sampaio 2003 [50]** | RCT | 1 | 2 | 1 | 4 |
| 14 | **Wallis 1996 [48]** | RCT | 2 | 2 | 1 | 5 |
| 15 | **Mahakalkar 2017 [49]** | RCT | 2 | 2 | 1 | 5 |

RCT: randomized control trial; Jadad scale score range (0-5); high-quality Jadad scale score (3−5); low-quality Jadad scale score (1−2)

**Supplementary Table 2 Risk of bias assessment of individual studies included**

| **NO.** | **Included studies** | **Adequate sequence generation** | **Allocation concealment** | **Blinding** | **Incomplete outcome**  **data addressed** | **Selective reporting (reporting bias)** | | | **Other bias** | |
| --- | --- | --- | --- | --- | --- | --- | --- | --- | --- | --- |
| 1 | **Daley 2015 [16]** | ✓ | ✓ | ✓ | U^?^ | | ✓ | ✓ | |  |
| 2 | **Farazi et al, 2017 [23]** | ✓ | U | ✓ | ✓ | | ✓ | ✓ | |  |
| 3 | **Martineau 2017 [21]** | ✓ | ✓ | ✓ | ✓ | | ✓ | ✓ | |  |
| 4 | **Mily 2015 [18]** | ✓ | ✓ | ✓ | U^?^ | | ✓ | ✓ | |  |
| 5 | **Nursyam 2006 [44]** | U | ✓ | U | ✓ | | ✓ | ✓ | |  |
| 6 | **Ralph 2013 [20]** | ✓ | ✓ | ✓ | U^?^ | | ✓ | ✓ | |  |
| 7 | **Salahuddin 2013 [14]** | ✓ | ✓ | ✓ | ✓ | | ✓ | ✓ | |  |
| 8 | **Tukvadze 2015 [46]** | ✓ | ✓ | ✓ | ✓ | | ✓ | ✓ | |  |
| 9 | **Wejse 2009 [45]** | ✓ | ✓ | ✓ | U^?^ | | ✓ | ✓ | |  |
| 10 | **Ganmaa 2017 [15]** | ✓ | ✓ | ✓ | ✓ | | ✓ | ✓ | |  |
| 11 | **Johnson 2003 [25]** | ✓ | ✓ | ✓ | ✓ | | ✓ | ✓ | |  |
| 12 | **Mayanja-Kizza 2005 [47]** | ✓ | ✓ | ✓ | ✓ | | ✓ | ✓ | |  |
| 13 | **Pedral-Sampaio 2003 [50]** | U | ✓ | ✓ | ✓ | | ✓ | ✓ | |  |
| 14 | **Wallis 1996 [48]** | ✓ | ✓ | ✓ | ✓ | | ✓ | ✓ | |  |
| 15 | **Mahakalkar 2017 [49]** | ✓ | ✓ | ✓ | U^?^ | | ✓ | ✓ | |  |

✓denotes low risk of bias; U denotes unclear risk of bias; ? denotes risk of bias due to incomplete outcome data assessed as ‘unclear’ due to relatively high rates of loss to follow-up (>20%)

**Supplementary Table 3:** **Frequency of outcome assessment and follow-up duration**

| **Frequency of assessing outcomes and follow-up duration/weeks** | | | | | | | | | |
| --- | --- | --- | --- | --- | --- | --- | --- | --- | --- |
| **Author (year)** | **Sputum culture** | **Sputum smear** | **Chest radiograph** | **BMI** | **CRP** | **ESR** | **TB score** | **Weight** | **MUAC** |
| **Martineau 2011 [21]** | 0, 2, 4, 6, 8 | 0, 2, 4, 6, 8 | 0,8 | 0,2, 4, 6, 8 | 0,2, 4, 6, 8 | 0, 2, 4, 6,8 | ND | ND | ND |
| **Daley 2015 [16]** | 0, 2, 4, 6, 8 | 0, 2, 4, 6, 8 | ND | 0,8 | ND | ND | ND | 0,8 | ND |
| **Farazi 2017 [23]** | ND | 0,4,8,12 | ND | 0,4,8,12 | 0,4,8,12 | 0,4,8,12 | 0,4,8,12 | ND | 0 |
| **Mily 2015 [18]** | 0,4,8 | 0-4, 6, 8, 10, 12, 24 | 0, 8, 12,24 | ND | 0, 4, 8, 12 ,24 | 0, 4, 8, 12 ,24 | 0-4, 6, 8, 10, 12,24 | 0-4, 6, 8, 10, 12,24 | ND |
| **Nursyam 2006 [44]** | ND | 0,6,8 | 0,24 | 0,1,6 | ND | ND | ND | ND | ND |
| **Ralph 2013 [20]** | 0,4,8 | 0-8, 20, 24 | 0, 8, 24 | 0 | ND | ND | ND | 0,8 | ND |
| **Salahuddin 2013 [14]** | ND | 0,4,8,12 | 0,12 | 0,4,8,12 | ND | ND | 0,4,8,12 | 0,4,8,12 | 0,4,8,12 |
| **Tukvadze 2015 [46]** | 0,2,4,6,8,12,16 | 0,8 | 0,4,8 | 0,4,8 | ND | ND | ND | NM | ND |
| **Ganmaa 2017 [15]** | 0, 2, 4, 6, 8 | 0, 2, 4, 6, 8 | 0,8 | 0,2, 4, 6,8 | 0,2, 4, 6,8 | 0,2, 4, 6,8 | ND | NM | 0,2, 4,6,8 |
| **Wejse 2009 [45]** | ND | 0,2,4,6,8, 20,32 | ND | 0 | ND | ND | 0,8,20,32 | 0,32 | 0 |
| **Johnson 2003 [25]** | 0,1-4,6 | 0,1-4,6 | 0,4,8,24,48 | ND | ND | ND | ND | 0,4 | ND |
| **Mayanja-Kizza 2005 [47]** | 0,4,8 | 0,4,8,16,24 | NM | 0 | ND | ND | ND | 0 | ND |
| **Pedral-Sampaio 2003 [50]** | 1,2,4,8 | 1,2,4,8 | ND | ND | ND | ND | ND | 0 | ND |
| **Wallis 1996 [48]** | 1-16 | ND | 1-4,24,36,60 | ND | ND | ND | ND | 0-16 | ND |
| **Mahakalkar 2017 [49]** | ND | 1-4,12,16,20,24 | 0,8,24 | ND | ND | ND | ND | 0,8,24 | ND |

ND: not determined; BMI:body mass index; MUAC: mid-upper arm circumference; TB:tuberculosis; CRP:C-reactive protein; ESR: erythrocyte sedimentation rate

**Supplementary Table 4a: Outcome measurement at end point by allocation**

| **Author (year)** | **Reported outcome measurements in treatment vs placebo groups respectively** | | | | |
| --- | --- | --- | --- | --- | --- |
|  | ***Sputum smear**  **n/N(%)** | ****Sputum culture**  **n/N(%)** | **^#^Sputum smear**  **(95% CI)** | **^@^Sputum culture**  **(95% CI)** | **Chest radiograph**  **(95% CI)** |
| **Martineau 2011 [21]** | 45/52 (86.5) vs 40/56 (71.4) | 41/52 (78.8) vs 45/56 (80.4); p=0.85 | ^ϕ^1.26 ( 0.83–1.93); p=0.28 | 36 (31.8–40.2) vs 43.5 (36.5–50.5); p=0.41 | ^Ǫ^2.30 ±1.29 vs 2.28 ±1.18 (–0.45-0.27) ; p=0.6 |
| **Daley 2015 [16]** | 85/101 (84) vs 88/110 (80); p=0·95 | 59/73 (80.8) vs 68/82 (82.9) | 43·0 (33·3–52·7) vs 43·0 (36·1–49·9); p=0·95 | 43·0 (33·2–52·8) vs 42·0 (33·9–50·1); p=0·95 | NM |
| **Farazi 2017 [23]** | 28/34 (93.3) vs 22/34 (73.3); p= 0.04 | NM | ^α^0.20 (0.04–1.02); p= 0.037 | NM | NM |
| **Mily 2015 [18]** | NA | 61/62(98.4) vs 57/64 (89.1); p = 0.032 | ^β^3.37 (1.0–10.8); p=0.041 | ^α^2.20 (1.07–4.51)  p = 0.032 | ^γ^0.87(0.35–2.18) |
| **Nursyam 2006 [44]** | 34/34 (100) vs 25/33 (76.7) ; p = 0.002 | NA | NA | NM | ^ῼ^ 30/34(87.5) vs 22/33(65.0) |
| **Ralph 2013 [20]** | NA | 44/75 (58.7) vs 52/80 (65.0); p=0.42 | ^µ^0.84(0.62-1.15) | ^ῼ^24/37 (64.9) vs 45/54 (83); p=0.05 | ^Ƶ^-20.5 vs -23.0; p=0.87 |
| **Salahuddin 2013 [14]** | 108/132 (81.8) vs 103/127 (81.1);p=0.39 | NM | NA | NM | ^к^50%↓; p= 0.035 |
| **Tukvadze 2015 [46]** | 85/97 (87.6) vs 84/95 (88.4) ; p=0.83 | 75/83 (90.4) vs 73/86 (84.9); p=0.28 | NA | 29 (24-36) vs 27(23-36); p = 0.99 | NA |
| **Ganmaa 2017 [15]** | 131/190 (68.9) vs 115/200 (57.5) | 150/190 (78.9) vs. 148/200 (74.0); p= 0.14 | ^ϕ^1.47(1.09–1.98); p = 0.01 | 1.11(0.88–1.39); p= 0.39 | ^Ǫ^5.48 vs. 5.69 (-0.06–0.77) ; p = 0.02 |
| **Wejse 2009 [45]** | 92/100 (92) v 93/100 (93) | NM | NM | NM | NM |
| **Johnson 2003 [25]** | ^ᵟ^48/48 (100) vs 44/47 (93.6); p=0.03 | 36/48 (77) vs 40/47 (85) ; p=0.29 | NA | ^ρ^Wilcoxon,p=0.05 | NA |
| **Mayanja-Kizza 2005 [47]** | 61/93 (65) vs 36/94 (38); p=0.002 | 58/93(62.0) vs. 35/94(37.0); p=0.001 | NM | NM | NM |
| **Pedral-Sampaio 2003 [50]** | 10/14 (70) vs 8/14 (55); p=0.07 | 7/14 (50.0) vs 5/14 (35.7); p= 0.44 | ^&^P=0.07 | ^&^P=0.08 | NM |
| **Wallis 1996 [48]** | NM | 49/52(95) vs 51/55(92) | NM | NM | NA |
| **Mahakalkar 2017 [49]** | 23/24 (95.83) vs 14/24 (58.35); p= 0.002 | NM | NM | NM | ^ῼ^ 21/24 (87.5) vs 8/24 (33.33) ; p= 0.05 |

NA: Not assessed or available; NM: not measured; *: proportion of subject with negative sputum smear ; **: proportion of subject with negative sputum culture; #: time to sputum smear conversion in days; @: time to sputum culture conversion in days; N: total number of subjects allocated to either treatment or placebo group; n: number (proportion) of subjects with either sputum smear or culture negative; TB:tuberculosis; HR: hazard ratio; CI: confidence interval; vs: verses; α: odds in favour of vitamin D treatment group;ϕ: adjusted hazard ratio; β: log rank test favours vitamin D treatment group after 8 weeks of supplentation; γ: adjusted odds ratio in favour of placebo group;µ: hazard ratio in favore of placebo group though not significant; к: reduction in TB cavity size in favour of treatment group; ρ: time until sputum culture conversion longer among subjects in the IL-2 arm; ᵟ:sputum bacillary load higher in IL-2 group compared with placebo; &: a trend toward faster sputum smear and culture conversion time to negative in rhu-GM-CSF group; ῼ: proportion, n/N(%); Ƶ: median; Ǫ:mean; p-value significant at p<0.05

**Supplementary Table 4b: Outcome measurement at end point by allocation**

| **Author (year)** | **Reported outcome measurements in treatment vs placebo groups respectively** | | | | |
| --- | --- | --- | --- | --- | --- |
|  | **Weight gain**  **Mean SD (95% CI)/kg** | **BMI**  **Mean SD (95% CI)/kg/m²** | **CRP**  **Mean SD (95% CI)** | **ESR**  **Mean SD (95% CI)** | **MUAC**  **Mean SD (95% CI)** |
| **Martineau 2011 [21]** | NM | 21.29 ±2.72 vs 21.18 ±2.75  (-0.28 – 0.46); p=0.63 | 19·59 ±25·23 vs 17·82 ±22·73(-7·14–9·71); p=0·76 | 29·90 ±25·91 vs 30·11 ±25·22 (-9·27– 9·76); p=0·96 | NM |
| **Daley 2015 [16]** | ^µ^↑3·1; p<0·0001 | **↑0·087; p=0·59 | NM | NM | NM |
| **Farazi 2017 [23]** | NM | 24.66±4.3 vs 22.31±3.9  (0.23,-4.47); p=0.03 | *****2.98 (1.04–8.52); p=0.04 | 15±50 vs 17±56.7 (0.28–2.11); p= 0.60 | NM |
| **Mily 2015 [18]** | 46.3±9.5 vs 44.9±7.6 | NM | 5.68±0.60 vs 5.80±0.60 | ^ß^33.5±22.0 vs 36.3±22.2 | NM |
| **Nursyam 2006 [44]** | NM | 17.88±1.79 vs 18.77±2.47 | NM | NM | NM |
| **Ralph 2013 [20]** | ^ῼ^30/101 (29.7) vs 31/99 (31.3); p=0.80 | NM | NM | NM | NM |
| **Salahuddin 2013 [14]** | + 3.75 (3.16 – 4.34) vs  + 2.61(1.99 –3.23) ; p=0.009 | + 1.39 (1.15 – 1.63) vs + 0.95 (0.71 –1.19) ; p=0.01 | NM | NM | + 1.34, (0.74 – 1.64) vs + 0.97, (0.68 – 1.26); p= 0.079 |
| **Tukvadze 2015 [46]** | NM | NA | NM | NM | NM |
| **Ganmaa 2017 [15]** | NM | 20.7 ±3.0 vs 21.2 ±3.3 :p=0.59 | 20.2 ±24.6 vs 25.2 ± 28.2; p= 0.06 | 12.6 ±10.5 vs 11.7 ±9.8; p=0.84 | ^@^0.25 (0.08-- 0.43); p=0.04 |
| **Wejse 2009 [45]** | ^Ƶ^5.9 vs 5.7 ; p=0.9 | NM | NM | NM | NM |
| **Johnson 2003 [25]** | NA | NM | NM | NM | NM |
| **Mayanja-Kizza 2005 [47]** | NM | NM | NM | NM | NM |
| **Pedral-Sampaio 2003 [50]** | NM | NM | NM | NM | NM |
| **Wallis 1996 [48]** | ^ψ^1.03 vs. 0.975 | NM | NM | NM | NM |
| **Mahakalkar 2017 [49]** | 2.66±0.18 vs −0.02±0.03; p<0.001 | NM | NM | NM | NM |

NM:Not measured ;NA:Not available; BMI:body mass index; MUAC: mid-upper arm circumference; CRP:C-reactive protein; ESR: erythrocyte sedimentation rate; CI: confidence interval; vs: verses; *: odds ratio, increase CRP in placebo group; ** increament in favour of placeboo group; ῼ: proportion, n/N(%); Ƶ: median weight gain; ψ:kg/month; µ:increase in favour of treatment group; ß: higher in the placebo group; @: adjusted mean difference in favour of treatment; p-value significant at p<0.05

**Supplementary Table 4c: Outcome measurement at end point by allocation**

| **Author (year)** | **Reported outcome measurements in treatment vs placebo groups respectively** | | | |
| --- | --- | --- | --- | --- |
|  | **Adverse event** | **Mortality** | **TB score**  **Mean SD (95% CI)** | **Other blood indice^$^**  **Mean SD (95% CI)** |
| **Martineau 2011 [21]** | NDf | NRS | NA | ^#^3.52 *vs* 2.70(0.16–1.11); p=0.009 |
| **Daley 2015 [16]** | NDf | NRS | NA | NM |
| **Farazi 2017 [23]** | NA | NA | 2.4 ±1.5 vs 3.7±1.7(-2.13 -- 0.47); p=0.003 | NM |
| **Mily 2015 [18]** | NDf | NA | NM | ^a^30.92±9.17 vs 28.73±9.16  ^b^4.32±2.54 vs 3.52±2.36 |
| **Nursyam 2006 [44]** | NA | NA | NA | ^c^2671.27±826.06 vs 959.82±957.62; p=0.278 |
| **Ralph 2013 [20]** | NDf | NRS | *6.9±2.0 vs 6.8±1.9 ;p= 0.68 | NM |
| **Salahuddin 2013 [14]** | NM | NM | - 3.19 ± 2.37(−3.61, -2.75) vs -2.79 ± 2.44(−3.23,2.34); p=0.198 | ^d^3092 ±1363 vs 2987 ±1510; p=0.497 |
| **Tukvadze 2015 [46]** | NDf | NRS | NA | ^ϓ^35 vs.6; p< 0.001 |
| **Ganmaa 2017 [15]** | NDf | NRS | NA | ^#^0.4 (0.2--0.6); p=0.001 |
| **Wejse 2009 [45]** | NDf | NRS | 2.4±2.0 vs 2.3±2.0; p= 0.35 | NM |
| **Johnson 2003 [25]** | ^ῼ^55/55(100) vs 1/55(2); p> 0.0001 | None | NA | NM |
| **Mayanja-Kizza 2005 [47]** | NDf | ^β^17/93(18) vs 14/94 (15) ; p=0.28 | NA | ^e^61.9 (37.4–89.2) vs 25.3 (17.1–38.1) ; p<0.001 |
| **Pedral-Sampaio 2003 [50]** | NDf | NRS | NA | ^f^8,425 ±3,374 vs 10,693 ±3,610 |
| **Wallis 1996 [48]** | NDf | NM | NA | ^χ^0.4 vs 0.6; p=0.27 |
| **Mahakalkar 2017 [49]** | NA | NM | NA | NM |

NA: not assessed;NM: not measured; $:other blood indices associated with infectivity and inflammation such as total white cells, lymphocytes, neutrophils, monocytes, Interferon-gamma and tumor necrosis factor-alpha; CI: confidence interval; vs: verses;*mean(SD); IFN-g:Interferon-gamma; NDf: No significant differences in frequencyof occurance between groups; NRS: Not related to study intervention; # in favour of treatment gorup; β: proportion of mortality recorded in the study. one was classified as probably related and two were classified as possibly related to the study intervention out of 17 death that occured in treatment arm (n/N(%)); χ:trend to reduced TNF-α in treatment group, log_10_(g/ml); ῼ: higher proportion of occurance (n/N(%)) in treatment group; #: lymphocyte to monocyte ratio; Other blood indice: (a) lymphocyte, (b) monocyte,(c) total lymphocyte, (d) *Mtbs*-stimulated IFN-g, (e) TNF-α and (f) total leukocytes ; ϓ: Higer proportion(%) of detectable serum anti–IL-2 antibodies in treatment group than placebo group; p-value significant at p<0.05

**FIGURES**


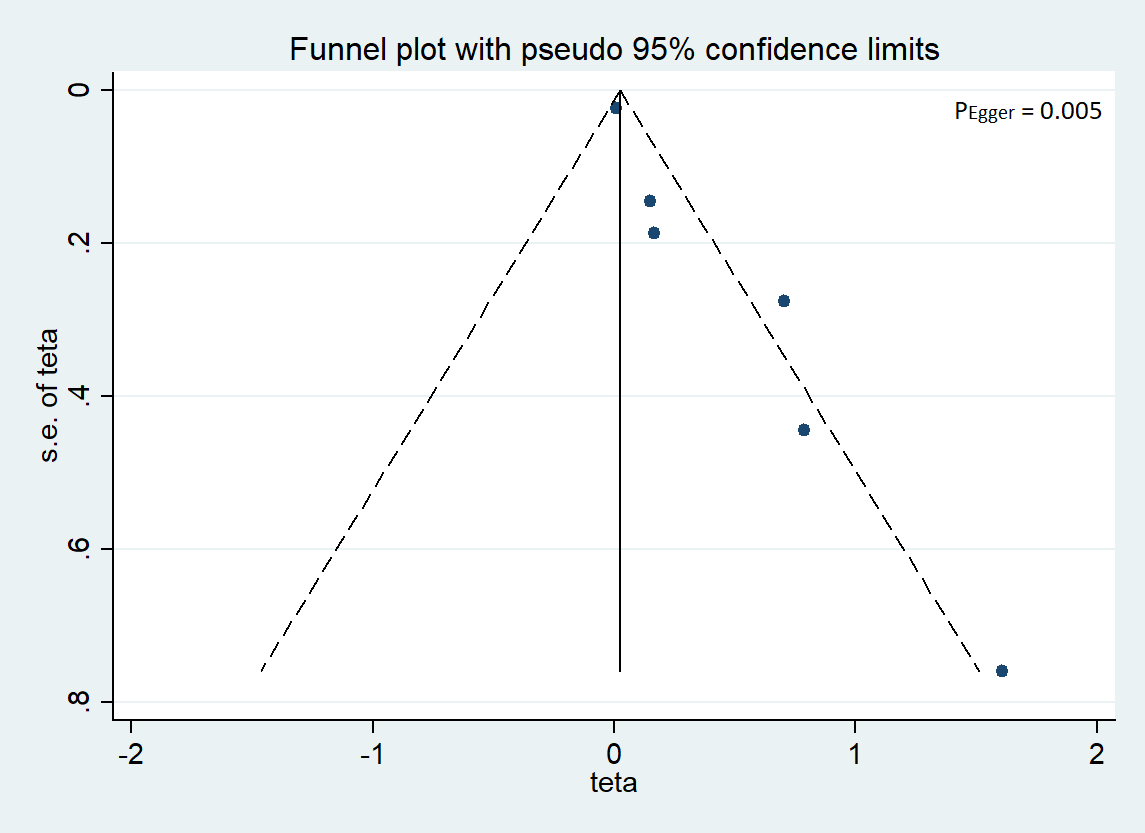


Supplementary figure 1: Funnel plot for aggregate patient data meta-analysis of sputum smear conversion rate conversion in vitamin D supplemented randomized controlled trials.


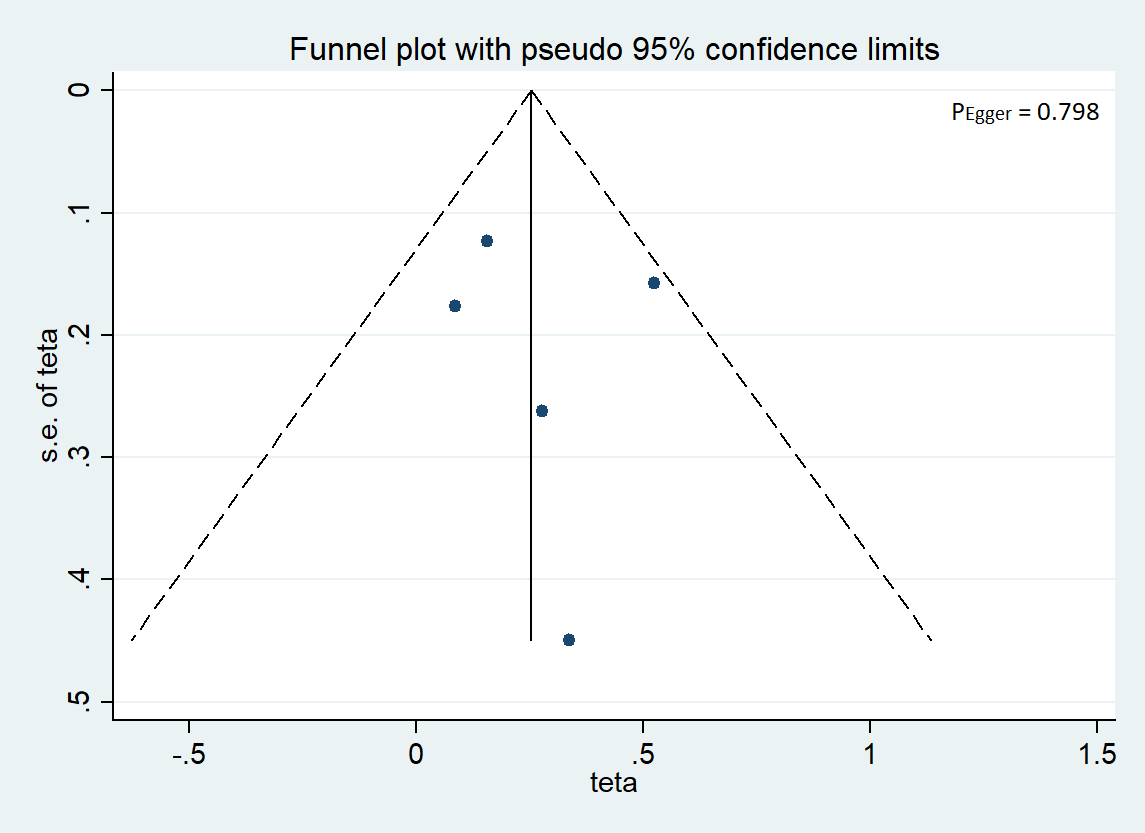


Supplementary figure 2: Funnel plot for aggregate patient data meta-analysis of sputum smear conversion rate conversion in other anti-inflammatory agents supplemented randomized controlled trials.
