# Supplementary figures and images for: The effects of anti-inflammatory agents as host-directed adjunct treatment of tuberculosis in humans: a systematic review and meta-analysis
Source: Respir Res. 2020 Aug 26;21:223. doi: 10.1186/s12931-020-01488-9 (PMC7448999; doi:10.1186/s12931-020-01488-9)

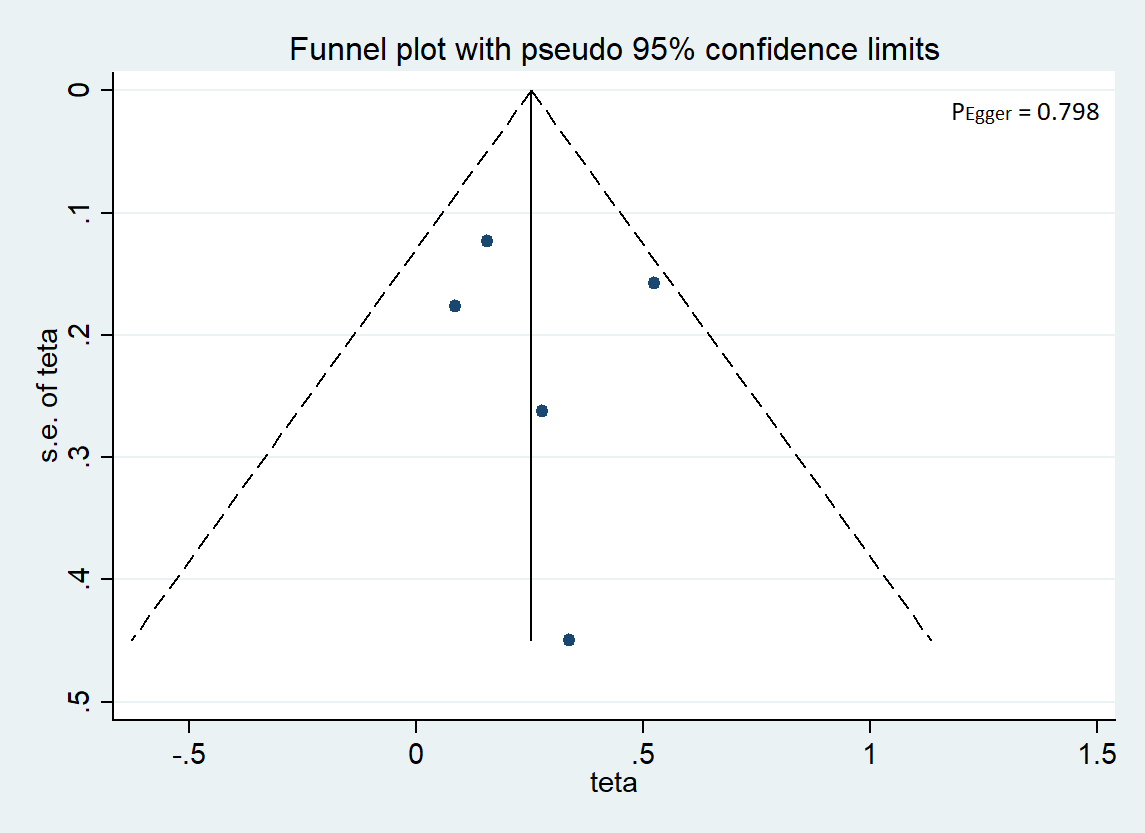

Supplement: Supplementary file 2 — Additional file 2: Supplementary Table 1. Quality assessment of included studies according to the Jadad scale. Supplementary Table 2. Risk of bias assessment of individual studies included. Supplementary Table 3. Frequency of outcome assessment and follow-up duration. Supplementary Table 4a. Outcome measurement at end point by allocation. Supplementary Table 4b. Outcome measurement at end point by allocation. Supplementary Table 4c. Outcome measurement at end point by allocation. Supplementary Figure 1. Funnel plot for aggregate patient data meta-analysis of sputum smear conversion rate conversion in vitamin D supplemented randomized controlled trials. Supplementary Figure 2. Funnel plot for aggregate patient data meta-analysis of sputum smear conversion rate in other anti-inflammatory HDT agents supplemented randomized controlled trials. [file 12931_2020_1488_MOESM2_ESM.zip › Supplementary figure 2_funnel plot other anti inf HDT group.tif]

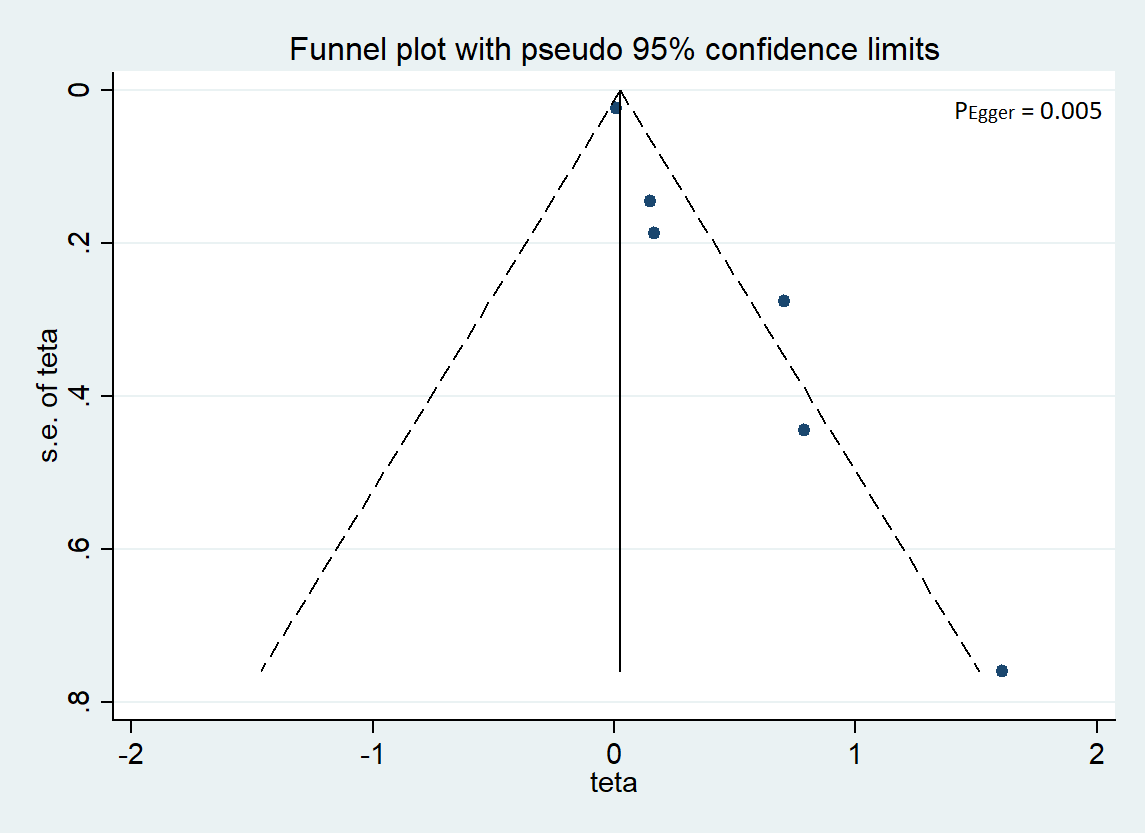

Supplement: Supplementary file 2 — Additional file 2: Supplementary Table 1. Quality assessment of included studies according to the Jadad scale. Supplementary Table 2. Risk of bias assessment of individual studies included. Supplementary Table 3. Frequency of outcome assessment and follow-up duration. Supplementary Table 4a. Outcome measurement at end point by allocation. Supplementary Table 4b. Outcome measurement at end point by allocation. Supplementary Table 4c. Outcome measurement at end point by allocation. Supplementary Figure 1. Funnel plot for aggregate patient data meta-analysis of sputum smear conversion rate conversion in vitamin D supplemented randomized controlled trials. Supplementary Figure 2. Funnel plot for aggregate patient data meta-analysis of sputum smear conversion rate in other anti-inflammatory HDT agents supplemented randomized controlled trials. [file 12931_2020_1488_MOESM2_ESM.zip › Supplemetary figure 1_funnel plot vitamin D group.tif]
